# Undescribed Phyllocladane-Type Diterpenoids from *Callicarpa giraldii* Hesse ex Rehd. and Their Anti-Neuroinflammatory Activity

**DOI:** 10.3390/molecules30071553

**Published:** 2025-03-31

**Authors:** Xu Liang, Qi Gong, Yuting Xu, Jiaxing Mu, Chunping Tang, Bintao Hu, Changqiang Ke, Sheng Yao, Haiyan Zhang, Yang Ye

**Affiliations:** 1 School of Chinese Materia Medica, Nanjing University of Chinese Medicine, Nanjing 210023, China; liangxu@simm.ac.cn; 2 State Key Laboratory of Drug Research, Natural Products Chemistry Department, Shanghai Institute of Materia Medica, Chinese Academy of Sciences, Shanghai 201203, China; mujiaxing@simm.ac.cn (J.M.); tangcp@simm.ac.cn (C.T.); 201728012342006@simm.ac.cn (B.H.); kechangqiang@simm.ac.cn (C.K.); 3 State Key Laboratory of Drug Research, Center for Neurological and Psychiatric Research, Shanghai Institute of Materia Medica, Chinese Academy of Sciences, Shanghai 201203, China; gq1021@simm.ac.cn (Q.G.); hzhang@simm.ac.cn (H.Z.); 4 School of Pharmaceutical Sciences, Southern Medical University, Guangzhou 510515, China; xuyuting551@zidd.ac.cn; 5 Zhongshan Institute for Drug Discovery, Shanghai Institute of Materia Medica, Chinese Academy of Sciences, Zhongshan 528400, China; 6 University of Chinese Academy of Sciences, Beijing 100049, China; 7 China-Serbia “Belt and Road” Joint Laboratory for Natural Products and Drug Discovery, Shanghai Institute of Materia Medica, Chinese Academy of Sciences, Shanghai 201203, China

**Keywords:** *Callicarpa giraldii*, phyllocladane-type diterpenoids, anti-neuroinflammatory activity, calligirlins A-R

## Abstract

*Callicarpa giraldii* Hesse ex Rehd. is an endemic plant in China and has long been used as a traditional medicine in several provinces. Although the plant has been reported to contain flavonoids, triterpenes, and alkaloids, this study represents the first report of the isolation of phyllocladane-type diterpenoids, a relatively rare class of compounds. In this study, 18 new phyllocladane-type diterpenoids (**7**–**24**) were isolated and structurally elucidated, including eight uncommon 3,4-*seco* phyllocladane-type diterpenoids (**15**–**22**) and two unusual phyllocladane-type diterpene dimers (**23**–**24**), along with six known analogues (**1**–**6**). Their structures were elucidated by a comprehensive analysis of 1D and 2D NMR, IR, and HRESIMS data. The absolute configurations were determined by single crystal X-ray diffraction experiments, DFT NMR calculations, and TDDFT ECD calculations. Based on the obtained and reported spectroscopic data, we refined a rule to distinguish phyllocladane-type diterpenoids from their diastereomeric *ent*-kaurane-type compounds. Additionally, the isolated compounds were evaluated for their in vitro anti-neuroinflammatory activity against lipopolysaccharide (LPS)-induced inflammation in BV-2 microglial cells. Compounds **5**, **10**, **13**, **18**, **19**, and **20** showed moderate inhibitory activity at the concentration of 20 μM, with compounds **5** and **13** markedly reducing the mRNA levels of the pro-inflammatory cytokines IL-1*β*, IL-6, and TNF-*α* at this concentration.

## 1. Introduction

Phyllocladane-type diterpenoids, a rare class of tetracyclic diterpenes, have been scarcely found in nature. To date, fewer than 50 diterpenes with this unique skeletal structure have been reported, primarily distributed across the genera *Callicarpa* [1], *Plectranthus* [2], *Anisomeles* [3], and *Cryptomeria* [4]. The pharmacological activities of these diterpenoids mainly include promoting plant growth [5], antibacterial properties [3,4], and cytotoxicity [1,6]. It should be noted that phyllocladane-type diterpenoids are easily misidentified as the more common *ent*-kaurane due to their diastereomeric relationship [7].

The genus *Callicarpa* (Lamiaceae) consists of approximately 190 species worldwide, with 46 species found in China, predominantly distributed in regions south of the Yangtze River [8]. *Callicarpa* plants are rich in diterpenes, which have been widely reported from this genus in recent years, including labdane- [9,10], abietane- [11,12,13], clerodane- [14], and phyllocladane-type diterpenes [1]. In the previous study in our lab, 3,4-*seco*-isopimarane and 3,4-*seco*-pimarane diterpenoids were isolated from the whole plant of *Callicarpa nudiflora* [15].

*Callicarpa giraldii* Hesse ex Rehd. (Lamiaceae) is an endemic plant in China, widely distributed in Gansu, Hubei, Fujian, Guangdong, Guangxi, Sichuan, Guizhou, and Yunnan provinces. Traditionally, it has been recorded for its effects in dispelling wind, removing dampness, dispersing blood stasis, and detoxifying. *C. giraldii* has been reported to contain a diverse array of secondary metabolites, including flavonoids, triterpenes, and alkaloids [16]. However, no reports have previously documented the isolation of diterpenes from this plant.

**Figure 1:**
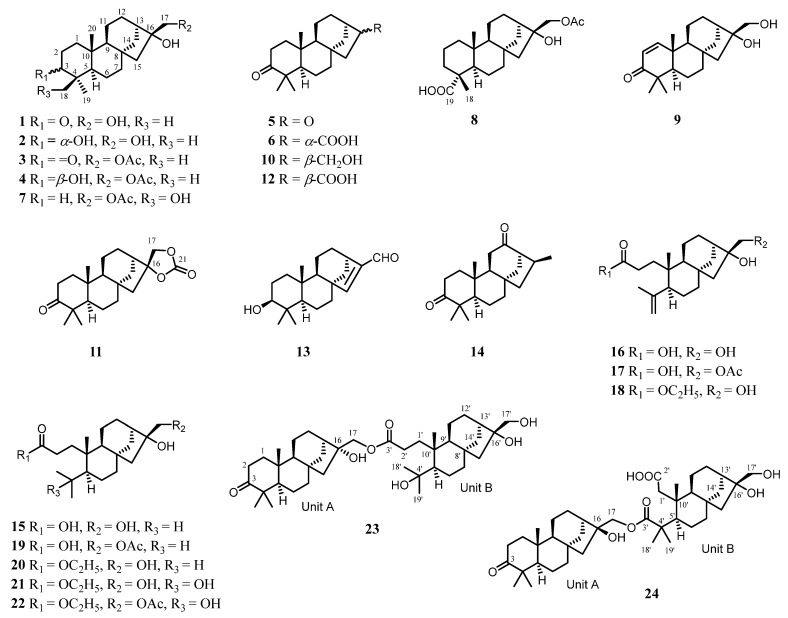
Structures of compounds **1**–**24** from *C. giraldii*.

In this study, a systematic investigation of the branches and leaves of *C. giraldii* was conducted, aiming to achieve an in-depth understanding of the chemical diversity of this plant, especially its diterpenoids (Figure 1), and the possible bioactivities relevant to its traditional usages. Herein, we describe the isolation and structural elucidation of these diterpenoids, as well as their anti-neuroinflammatory properties against lipopolysaccharide (LPS)-induced inflammation in BV-2 microglial cells.

## 2. Results

### 2.1. Spectroscopic Differentiation Between Phyllocladane- and Ent-Kaurane-Type Diterpenoids

Phyllocladane-type diterpenoids are a relatively rare class of compounds and are often misidentified as *ent*-kaurane type diterpenoids due to their diastereoisomeric relationship. It is necessary to refine simple spectroscopic rules to quickly distinguish these two types of diterpenes rather than relying mostly on single crystal X-ray diffraction experiments.

In 2000, Liu found that some of the reported NMR rules were not reliable for the determination of phyllocladane-type diterpenoids [17], based on the isolation and synthesis of several phyllocladane-type diterpenes. Dong further summarized that “the carbon spectra of the two diterpenes show obvious differences, particularly in C-14, C-15, and the methyl group at C-20” [7]. However, due to the limited variety of phyllocladane-type diterpenoids, the rule based on specific NMR data ranges for distinguishing these two diterpenes was not substantially refined.

**Figure 2:**
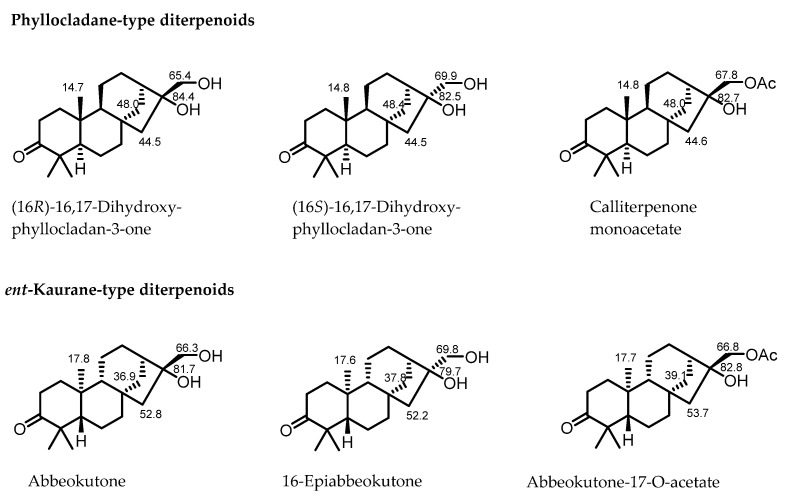
^13^C NMR data in CDCl_3_ of some known Phyllocladane- and *ent*-Kaurane-type diterpenoids [17,18,20].

In this study, six known phyllocladane-type diterpenes (**1**–**6**) were separated from *C. giraldii* and identified as calliterpenone (**1**) [18], (3*β*,16*α*)-phyllocladane-3,16,17-triol (**2**) [19], calliterpenone monoacetate (**3**) [1], (3*β*,16*α*)-phyllocladane-3,16,17-triol17-acetate (**4**) [19], 17-norphyllocladane-3,16-dion (**5**) [19], and (16*R*)-3-oxophyllocladan-17-oic acid (**6**) [17], respectively, by comparison of their spectroscopic data with those in literature. Fortunately, suitable single crystals of compounds **1**, **2**, and **5** were successfully obtained, which fully confirmed their absolute configurations. These data, in combination with the concrete data reported in the previous literature (Figure 2), provided more comprehensive ^13^C NMR rules to rapidly determine phyllocladane- or *ent*-kaurane-type diterpenoids (Table 1).

The chemical shift of C-14 exceeds 40 ppm in phyllocladane-type diterpenoids, while it is below 40 ppm in *ent*-kaurane-type diterpenoids.

The chemical shift of C-15 ranges from 40 to 50 ppm in phyllocladane-type diterpenoids, while it exceeds 50 ppm in *ent*-kaurane-type diterpenoids.

The chemical shift of Me-20 ranges from 14 to 15 ppm in phyllocladane-type diterpenoids, while it is around 17 ppm in *ent*-kaurane-type diterpenoids.

For phyllocladane-type diterpenoids substituted by a hydroxyl or an acetoxy group at C-17, the chemical shift of C-17 ranged from 65 to 68 ppm in compounds with *rel*-16*R*, while it exceeded 69 ppm in compounds with *rel*-16*S*.

### 2.2. Structural Elucidation of New Compounds ***7***–***24***

Compound **7** was isolated as colorless needle crystals. Its molecular formula was determined as C_22_H_36_O_4_ by HRESIMS data (*m*/*z* 387.2498 [M + Na]^+^, calcd for C_22_H_36_O_4_Na, 387.2511), indicative of five degrees of unsaturation. The IR spectrum (Figure S2) showed absorption bands for hydroxy (3428 cm^−1^) and carbonyl groups (1713 cm^−1^). The ^1^H NMR data (Table 2) showed three methyl groups (*δ*_H_ 2.10, 0.96, 0.85) and two oxygenated methylenes (*δ*_H_ 4.24, 4.16; *δ*_H_ 3.74, 3.41). The ^13^C NMR and DEPT data (Table 3) revealed 22 carbon atom signals, including three methyls, 11 methylenes, three methines, and five quaternary carbons (one ester carbonyl). These observations accounted for one degree of unsaturation, and the remaining four required a tetracyclic scaffold for compound **7**.

**Figure 3:**
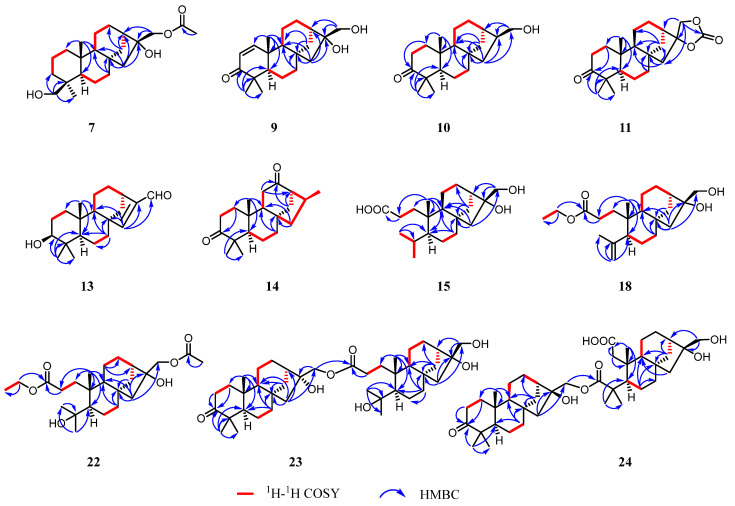
Key ^1^H-^1^H COSY and HMBC correlations of compounds **7**, **9**–**11**, **13**–**15**, **18**, and **22**–**24**.

The ^1^H–^1^H COSY spectrum revealed three spin-coupling systems of H_2_-1/H_2_-2/H_2_-3, H-5/H_2_-6/H_2_-7, and H-9/H_2_-11/H_2_-12 (Figure 3 and Figure S5). The HMBC spectrum (Figure 3 and Figure S7) exhibited key correlations from H_3_-20 to C-1/C-5/C-9/C-10, from H_3_-18 to C-3/C-4/C-5/C-19, and from H_2_-7 to C-8/C-9, which indicated the existence of two fused six-membered carbon rings, where Me-20 was located at C-10 while Me-18 and a hydroxymethyl (-CH_2_OH) were located at C-4. HMBC correlations from H_2_-14 to C-8/C-9/C-12/C-13 constructed another six-membered ring, which fused to ring C through C-8 and C-9. In addition, HMBC correlations from H_2_-15 to C-8/C-9/C-13/C-14/C-16 constructed a five-membered ring. Moreover, an acetoxy group connected to C-17 and a hydroxyl group located at C-16 were deduced from the HMBC correlations from H_2_-17 and the methyl of the acetoxy group to the carbonyl carbon (*δ*_C_ 171.4). Thus, the planer structure of compound **7** was established.

**Figure 4:**
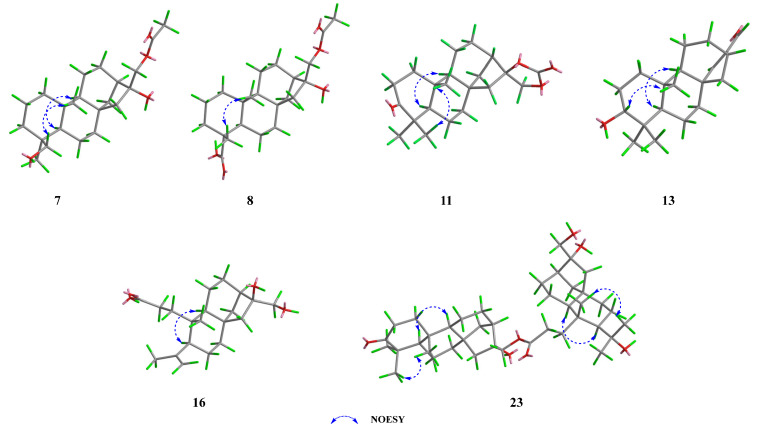
Key NOESY correlations of compounds **7**, **8**, **11**, **13**, **16**, and **23**. The 3D structure modeling was produced by PerkinElmer Chem3D by using MM2 minimization calculation.

**Figure 5:**
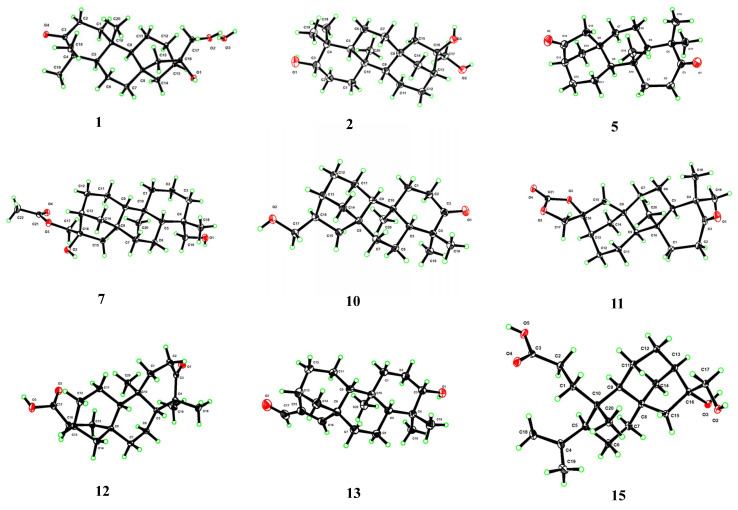
Perspective ORTEP drawings for compounds **1**, **2**, **5**, **7**, **10**, **11**, **12**, **13**, and **15**.

The NOESY correlations (Figure 4 and Figure S8) of H-5/H_3_-19 suggested that these protons were co-facial and *α*-oriented, while the cross-peak of H_2_-18/H_3_-20 indicated that they were on the opposite face and *β*-oriented. To figure out the orientation of C-16, DFT NMR calculation was carried out on two possible conformations of **7a** (16*R*) and **7b** (16*S*). The Boltzmann-weighted average NMR data of **7a** and **7b** were compared with the experimental data using the improved statistical method DP4+ probability [19]. Compound **7** gave 100% possibilities (H data, C data, and all data) for **7a**. The absolute configuration was further proved by a single-crystal X-ray diffraction experiment with Cu K*α* radiation (CCDC 2389914, Figure 5). Therefore, the structure of compound **7** was fully defined and named calligirlin A.

Compound **8** was isolated as a white solid. The HRESIMS and ^13^C NMR data gave a molecular formula of C_22_H_34_O_5_ with six degrees of unsaturation. The ^13^C and ^1^H NMR data of **8** (Table 2 and Table 3) also suggested a phyllocladane-type diterpene. Compared with compound **7**, a carboxyl group instead of a hydroxymethyl group was located at C-19, which was supported by the HMBC correlation (Figure 3 and Figure S17) from H_3_-18 to C-19 (*δ*_C_ 181.9). The NOESY correlation (Figure 4 and Figure S18) of H_3_-18/H_3_-20 suggested that these two methyls were on the same face and *β*-oriented, while the carboxyl group was on the other face and *α*-oriented. According to the spectroscopic rule (Table 2), the relative configuration of C-16 was designated as *R* by the chemical shift of C-17 (*δ*_C_ 67.9)*.* Therefore, given the biosynthetic consideration, the full structure of compound **8** was defined and named calligirlin B.

**Figure 6:**
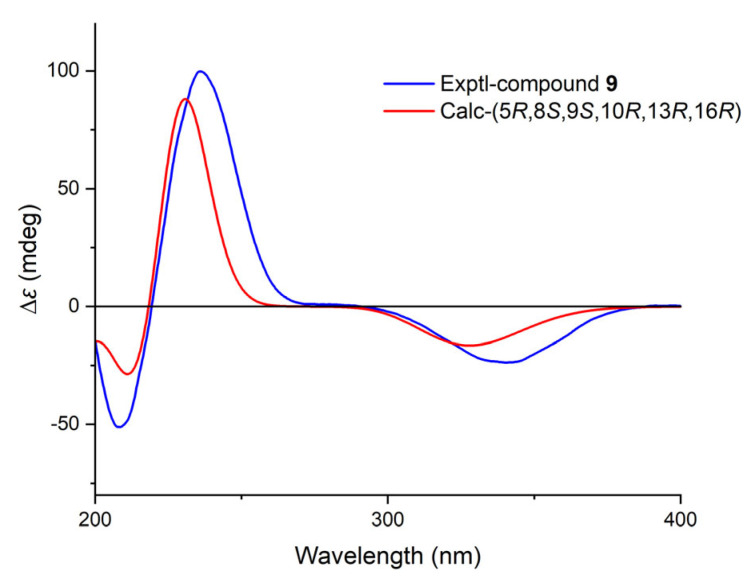
Comparison of experimental and calculated ECD spectra for compound **9** in methanol.

The molecular formula of **9** was established as C_20_H_30_O_3_ by HRESIMS. The NMR data comparison of **9** and **1** (Table 2 and Table 3) suggested that they might share the same skeleton [21]. The carbonyl carbon (*δ*_C_ 205.3) and two typical olefinic carbons (*δ*_C_ 158.2, 125.8; *δ*_H_ 6.99, 5.83) revealed the presence of an *α*, *β-*unsaturated *δ*-ketone moiety located at C-1/C-2/C-3, which was evidenced by the HMBC correlations from H-1 to C-3/C-5/C-10, from H-2 to C-4/C-10 (Figure 3 and Figure S25). The NOESY correlation of H-5/H-9 suggested that these protons were co-facial and *α*-oriented (Figure S26). The chemical shift of C-17 (*δ*_C_ 65.6) suggested a *rel*-16*R* for C-16. To further determine the absolute configurations, TDDFT ECD calculation was performed on the structure of **9** with assigned relative configurations. The non-redundant advantageous conformers were re-optimized at the M062X/6-31G(d) level using the SMD solvent model for methanol. Then, ECD calculations were performed at the CAM-B3LYP/TZVP level on the same solvent model for methanol. The results showed that the experimental ECD curve of compound **9** matched well with its calculated one (Figure 6). Therefore, the structure of **9** was fully established and named calligirlin C.

Compound **10** was isolated as colorless orthorhombic crystals and had a molecular formula of C_20_H_32_O_2_ based on its HRESIMS data, one less oxygen atom than that of **1**. Its NMR data (Table 2 and Table 3) were highly similar to those of **1**, except for some signals of ring D. The absence of OH-16 was evidenced by the upfield shift of C-16 (*δ*_C_ 36.9) (Table 3). The NOESY correlation of H-5/H-9 suggested that these protons were co-facial and *α*-oriented (Figure S34). The chemical shift of C-17 (*δ*_C_ 67.6) suggested that the relative configuration of C-16 was *R*. The full structure, including the absolute configuration of compound **10**, was confirmed by a single-crystal X-ray diffraction experiment with Cu K*α* radiation (CCDC 2389915, Figure 5). Therefore, the structure of compound **10** was proposed and named calligirlin D.

Compound **11** was isolated as light-yellow needle crystals. Its molecular formula was determined as C_21_H_30_O_4_ by HRESIMS, indicative of seven degrees of unsaturation. Its NMR data (Table 3 and Table 4) were similar to those of a known compound 16*α*, 17-isopropylideno-3-oxo-phyllocladane [22], suggesting it might also possess an additional 1,3-dioxolane located at C-16. When comparing their NMR data, a quaternary carbon (*δ*_C_ 154.7) in **11**, instead of a quaternary carbon (*δ*_C_ 108.7) and two methyl groups (*δ*_C_ 26.9, 26.9) in the known compound, was observed, implying the additional five-membered ring in **11** was a 1, 3-dioxolane-2-one. HMBC correlations from H_2_-17 to C-13/C-15/C-16/C-21 (Figure 3 and Figure S41) further supported such elucidation. The NOESY correlation of H_3_-18/H_3_-20, H-5/H_3_-19, and H-5/H-9 inferred the relative configurations (Figure 4 and Figure S42). The absolute configuration was finally established by a single-crystal X-ray diffraction experiment with Cu K*α* radiation (CCDC 2390159, Figure 5). Therefore, the full structure of compound **11** was defined and named calligirlin E.

The molecular formula of **12** was established as C_20_H_30_O_3_ by its HRESIMS data, the same as that of the known compound **6**. The ^1^H and ^13^C NMR data (Table 3 and Table 4) of **12** were almost identical to **6**, except for the differences observed for the chemical shifts of the carboxylic acid group. A suitable single crystal of compound **12** was obtained, and the single-crystal X-ray diffraction experiment with Cu K*α* radiation finally confirmed the full structure of **12** (CCDC 2389922, Figure 5). Accordingly, compound **12** was ultimately identified as an isomer of **6**, with the carboxyl group at the C-16 having the opposite configuration compared to that in **6**, and named calligirlin F.

Compound **13** was isolated as a white solid. The HRESIMS and ^13^C NMR data gave a molecular formula of C_20_H_30_O_2_ with six degrees of unsaturation. Its NMR data (Table 3 and Table 4) were indicative of a phyllocladane-type diterpene skeleton. Detailed analysis revealed the presence of an *α*, *β-*unsaturated *δ*-aldehyde (*δ*_C_ 156.9, 147.6, 190.0) and an oxygenated methine (*δ*_C_ 79.0). HMBC correlations (Figure 3 and Figure S57) from H-17 to C-13/C-16 and from H-15 to C-8/C-13/C-14/C-16/C-17 indicated the existence of the 15,16-ene-17-aldehyde moiety. HMBC correlations from H-3 to C-2 and C-4 suggested a hydroxy group attached to C-3. The NOESY correlation of H-3/H-5 suggested that the hydroxy group was *β*-oriented (Figure 4 and Figure S58). Fortunately, a suitable single crystal of compound **13** was obtained, and the single-crystal X-ray diffraction experiment with Cu K*α* radiation finally confirmed the full structure of **13** (CCDC 2389916, Figure 5). The structure of compound **13** was then established and named calligirlin G.

Compound **14** was isolated as a white solid. Its molecular formula was determined as C_20_H_30_O_2_ by HRESIMS. A detailed NMR data analysis (Table 3 and Table 4) suggested that **14** was an analogue of **5** but with an additional methyl group. Further analysis of its NMR data revealed the existence of Me-17 and 12-ketone, which could be supported by the HMBC correlations (Figure 3 and Figure S65) from H_3_-17 to C-13/C-15/C-16, from H-13 to C-12/C-15/C-14, from H_2_-11/to C-9/C-12/C-13, and from H_2_-14 to C-8/C-9/C-12/C-13. To figure out the orientation of C-16, DFT calculation of ^1^H and ^13^C NMR chemical shifts was carried out on two possible conformations of **14a** (16*S*) and **14b** (16*R*). The Boltzmann-weighted average NMR data of **14a** and **14b** were compared with the experimental data using the improved statistical method DP4+ probability. The result gave 100% possibilities (H data, C data, and all data) for **14a**. Therefore, given the biosynthetic consideration, the structure and absolute configuration of compound **14** were proposed and named calligirlin H.

Compound **15** was isolated as colorless needle crystals. Its molecular formula was determined as C_20_H_34_O_4_ by HRESIMS, corresponding to four degrees of unsaturation. A detailed analysis of its NMR data (Table 5 and Table 6) was conducted, and the ^13^C NMR and DEPT data (Table 6) only revealed 19 carbon atom signals, including three methyls, nine methylenes, four methines, and three quaternary carbons. The missing carbon was identified as C-3 by the HMBC correlations from H_2_-1 and H_2_-2 to the carbonyl carbon at *δ*_C_ 178.6. As one degree of unsaturation was occupied by the carboxyl group, the remaining three suggested a three-ring skeleton. The planer structure of **15** was established by 2D NMR data. The ^1^H–^1^H COSY spectrum (Figure 3 and Figure S73) revealed three spin-coupling systems of H_2_-1/H_2_-2, H-5/H_2_-6/H_2_-7 and H-9/H_2_-11/H_2_-12, H-13/H_2_-14. HMBC correlations (Figure 3 and Figure S75) from H_3_-20 to C-1/C-2/C-5/C-9/C-10, and from H_2_-7 to C-5/C-6/C-8/C-9/C-10 indicated a six-membered ring. HMBC correlations from H-9 to C-8/C-11/C-12/C-13 indicated another six-membered ring, which was fused to the former one through C-8 and C-9. HMBC correlations from H_2_-15 to C-8/C-9/C-12/C-14/C-16, and from H_2_-17 to C-13/C-16, constructed a five-membered ring with a hydroxymethyl (C-17) and a hydroxyl group both located at C-16. In addition, an isopropyl was located at C-5, which was deduced from the ^1^H-^1^H COSY correlations of H_3_-18/H-4/H_3_-19 and the HMBC correlations from the H_3_-18 to C-5/C-19. Besides Me-20, a propanoic acid group was attached to C-10, which was established by the HMBC correlations from H_2_-2 to C-1/C-3 and from Me-20 to C-1. Thus, the structure of **15** was constructed as a 3,4-*seco* phyllocladane diterpene, a rare type of phyllocladane derivative. The NOESY correlation (Figure S76) of H-5/H-9 suggested that these protons were co-facial and *α*-oriented. The absolute configuration was established by a single-crystal X-ray diffraction experiment with Cu K*α* radiation (CCDC 2389933, Figure 5). Therefore, the structure of compound **15** was fully defined and named calligirlin I.

Compound **16** was obtained as a white solid. Its molecular formula was determined to be C_20_H_32_O_4_ with five degrees of unsaturation by HRESIMS. The ^1^H and ^13^C NMR data of **16** (Table 5 and Table 6) showed high similarities to those of **15**, suggesting **16** was also a 3,4-*seco* phyllocladane-type diterpene. Comparison of their NMR data revealed the presence of a terminal double bond (*δ*_C_ 114.2, 148.8, *δ*_H_ 4.67, 4.88) and a singlet methyl (*δ*_C_ 24.3, *δ*_H_ 1.75) in **15**, replacing a methine (*δ*_C_ 26.7, *δ*_H_ 1.91) and two doublet methyl (*δ*_C_ 25.2, 19.5, *δ*_H_ 0.93, 0.87) as found in **16**. HMBC correlations (Figure 3 and Figure S83) from the olefinic proton (*δ*_H_ 4.88, 4.67) to C-19/C-4/C-5 indicated an isopropenyl attached to C-5. The NOESY correlation (Figure 4 and Figure S84) of H-5/H-9 suggested that these protons were *α*-oriented. According to the above-mentioned spectroscopic rules, the chemical shift of C-17 (*δ*_C_ 66.2) implied a *rel*-*R*-configuration for C-16. Therefore, the whole structure of **16** was proposed and named calligirlin J.

Comprehensive analysis of NMR data of compound **17** (Table 5 and Table 6) revealed that **17** was an acetylated derivative of **16**. The acetoxy group was connected to C-17 owing to the HMBC correlations (Figure 3 and Figure S91) from the methyl (*δ*_H_ 2.10) and H_2_-17 to the carbonyl carbon (*δ*_C_ 171.4). The chemical shift of C-17 (*δ*_C_ 67.9) suggested that the relative configuration of C-16 was *R* (Figure 4). Therefore, the whole structure of compound **17** was proposed and named calligirlin K.

Compound **18** was obtained as a white solid. The ^1^H and ^13^C NMR data (Table 5 and Table 6) of **18** showed high similarities to those of **16**, suggesting that they might possess the same skeleton. The HMBC correlations (Figure 3 and Figure S99) from the quartet oxygenated methylene (*δ*_H_ 4.10) to the carbonyl carbon (*δ*_C_ 113.9, C-3), and from the triplet methyl (*δ*_H_ 1.24) to the oxymethylene carbon (*δ*_C_ 60.5), indicated that **18** was an ethyl ester derivative of **16**, and the esterification happened at C-3. The NOESY correlation (Figure S100) of H-5/H-9 was observed, suggesting *α*-orientations for both protons. The chemical shift of C-17 (*δ*_C_ 65.8) suggested the typical *rel*-16*R* in a phyllocladane-type diterpene. Therefore, the structure of compound **18** was proposed and named calligirlin L.

Compound **19** was obtained as a white solid. Its molecular formula was determined to be C_22_H_36_O_5_ with five degrees of unsaturation by HRESIMS. The ^1^H and ^13^C NMR data (Table 6 and Table 7) of **19** showed high similarities to those of compound **15**, except for the presence of an additional acetyl group (*δ*_H_ 2.11, s; *δ*_C_ 171.4, 21.1) in **19**. HMBC correlations (Figure 3 and Figure S107) from H_2_-17 to the carbonyl carbon (*δ*_C_ 171.4, C) suggested that an acetoxyl rather than a hydroxy was connected to C-17. The NOESY correlation (Figure S108) of H-5/H-9 and the chemical shift of C-17 (*δ*_C_ 67.9) suggested the same relative configurations with compound **5**. Therefore, given the biosynthetic consideration, the whole structure of compound **19** was established and named calligirlin M.

Compound **20** was obtained as a white solid. Its molecular formula was determined to be C_22_H_38_O_4_ with four degrees of unsaturation by HRESIMS. The ^1^H and ^13^C NMR data analysis (Table 6 and Table 7) revealed that **20** was also an ethyl ester derivative of compound **15**, similar to the above-mentioned compounds **16** and **18**. Such elucidation was supported by the HMBC correlations (Figure 3 and Figure S115) from the oxygenated methylene (*δ*_H_ 4.14) to the carbonyl carbon (*δ*_C_ 174.5, C-3) and from the triplet methyl (*δ*_H_ 1.28) to the oxymethylene carbon (*δ*_C_ 60.5). Therefore, the whole structure of compound **20** was proposed and named calligirlin N.

The molecular formula of compound **21**, determined by HRESIMS, was established as C_22_H_38_O_5_, one more oxygen atom than that of **20**. Its ^1^H and ^13^C NMR data (Table 6 and Table 7) showed high similarities to those of **20**, expect that two doublet methyls (*δ*_C_ 25.0, 19.1, *δ*_H_ 0.92, 0.80) and one methine (*δ*_C_ 25.2, *δ*_H_ 1.88) were observed in **21**, replacing two singlet methyl (*δ*_C_ 34.2, 27.8, *δ*_H_ 1.28, 1.22) and one oxygenated quaternary carbon (*δ*_C_ 75.9) compared to **20**. HMBC correlations (Figure 3 and Figure S123) from H-5 to C-4/C-10/C-6/C-18/C-19/C-20 and from H_3_-18 to C-4/C-5 indicated a hydroxyl group located at C-4. Accordingly, the structure of compound **21** was fully established and named calligirlin O.

The molecular formula of compound **22** was determined to be C_24_H_40_O_6_ with five degrees of unsaturation by HRESIMS. The detailed analysis of the ^1^H and ^13^C NMR data of **22** (Table 6 and Table 7) revealed that **22** possessed a similar skeleton to that of **21**, except for an additional acetoxy group observed in **22**. HMBC correlations (Figure 3 and Figure S131) from the methyl (*δ*_H_ 2.10) and H_2_-17 to the carbonyl carbon (*δ*_C_ 171,4) supported that the acetoxyl group was attached to C-17. Therefore, the structure of compound **22** was proposed and named calligirlin P.

Compound **23** was isolated as a white solid. Its molecular formula was determined as C_40_H_64_O_7_ by the pseudo molecular ion peak (*m*/*z* 679.4544 [M + Na]^+^, calcd for C_40_H_64_O_7_Na 679.4550) in the HRESIMS, corresponding to nine degrees of unsaturation. The IR spectrum showed absorption bands for hydroxy (3440 cm^−1^) and carbonyl groups (1704 cm^−1^). The ^1^H NMR data (Table 8) showed the signals of six methyl groups. The ^13^C NMR and DEPT data (Table 8) revealed the resonances of 40 carbon signals ascribed to six methyls, 18 methylenes, six methines, and 10 quaternary carbons (a carbonyl and an ester carbonyl carbon). A detailed analysis of these NMR data revealed that compound **23** might be a compound dimerized from two different monomeric units of phyllocladane-type diterpene.

The ^1^H–^1^H COSY cross-peaks revealed four spin systems of H_2_-1/H_2_-2, H_2_-6/H_2_-7, H-9/H_2_-11/H_2_-12, H-13/H_2_-14 (Figure 3 and Figure S137). HMBC correlations from H_3_-20 to C-1/C-9/C-10, from H_3_-18 to C-3/C-4/C-5/C-19, from H_2_-7 to C-6/C-8/C-9, from H_2_-14 to C-8/C-11/C-12/C-13/C-16, and from H_2_-15 to C-8/C-9/C-14/C-16/C-17 were observed (Figure 3 and S139). These data constructed a half fragment of Unit A, as shown in Figure 1. Similarly, the spin systems of H_2_-1’/H_2_-2’, H-9’/H_2_-11’/H_2_-12’ and H-13’/H_2_-14’ deduced by ^1^H–^1^H COSY correlations, along with the HMBC correlations from H_3_-20’ to C-1’/C-5’/C-9’/C-10’, from H_2_-7’ to C-5’/C-6’/C-8’/C-9’, from H-9’ to C-8’/C-11’/C-13’, from H_2_-15’ to C-8’/C-9’/C-13’/C-14’/C-16’, from H_2_-17’ to C-16’/C-13’, from H_3_-18’ to C19’/C-4’/C-5’, and from H_2_-2’ to C-1’/C-3’ manifested the other fragment of Unit B (Figure 1). Furthermore, the HMBC correlations from H_2_-17 (*δ*_H_ 4.19) to C-3’ provided strong evidence for the connection of two fragments via an ester carbonyl group. Thereby determining the planar structure of **23**.

The NOESY correlations (Figure 4 and Figure S140) of H-5/H-9 and H-5’/H-9’ suggested that these protons were co-facial and *α*-oriented. The chemical shift of C-17 (*δ*_C_ 67.6) and C-17’ (*δ*_C_ 65.7) suggested the *β*-orientation for both C-17 and C-17’. Therefore, given the biosynthetic consideration, the full structure of compound **23** was proposed and named calligirlin Q.

Compound **24** was isolated as a colorless oil. Its molecular formula was determined as C_40_H_62_O_8_ by HRESIMS, indicating 10 degrees of unsaturation. A detailed analysis of its ^1^H and ^13^C NMR spectra (Table 8), with the aid of HSQC data, revealed 40 carbon resonances, including six methyls, 17 methylenes, six methines, and 11 quaternary carbons. The above characteristic resonances suggested that **24** could also be a phyllocladane-type diterpenoid dimer. The same Unit A (Figure 1) was constructed by the ^1^H–^1^H COSY correlations (Figure 3 and Figure S145), indicating four spin systems of H_2_-1/H_2_-2, H_2_-6/H_2_-7, H-9/H_2_-11, and H_2_-12/H_2_-13, and the HMBC correlations from H_3_-20 to C-1/C-9/C-10, from H_3_-18 to C-3/C-5/C-19, from H_2_-7 to C-5/C-8/C-9, from H_2_-14 to C-8/C-9/C-12/, and from H_2_-15 to C-8/C-9/C-14/C-16/C-17 (Figure 3 and Figure S147). The Unit B of **24** (Figure 1) was established by three spin-coupling systems of H-5’/H_2_-6’, H-9’/H_2_-11’, and H-13’/H-14’ (Figure 3 and Figure S147), and the HMBC correlations from H_2_-1’ to C-2’/C-5/C-9’/C-10’/C-20’ and from H_3_-20 to C-2’/C-5/C-9’/C-10’ (Figure 3 and Figure S147). HMBC correlations from H_3_-18’ to C-3’/C-5/C-19’ suggested that one carbonyl group was located at C-3’ (*δ*_C_ 180.7). The other one (*δ*_C_ 175.8) was placed at C-2’ by H_3_-20 to C-2’/C-5/C-9’/C-10’. Thus, the moiety of Unit B was a 2,3-*seco* phyllocladane diterpene. HMBC correlation from H_2_-17 to C-3’ further confirmed that these two units were also connected through the ester carbonyl group. The NOESY correlations (Figure 4 and Figure S148) of H-5/H-9 and H-5’/H-9’ suggested that these protons were co-facial and *α*-oriented. The chemical shift of C-17 (*δ*_C_ 69.0) suggested that C-17 was *β*-oriented. Therefore, given the biosynthetic consideration, the full structure of compound **24** was proposed and named calligirlin R.

### 2.3. Anti-neuroinflammatory Activity Evaluation

**Figure 7:**
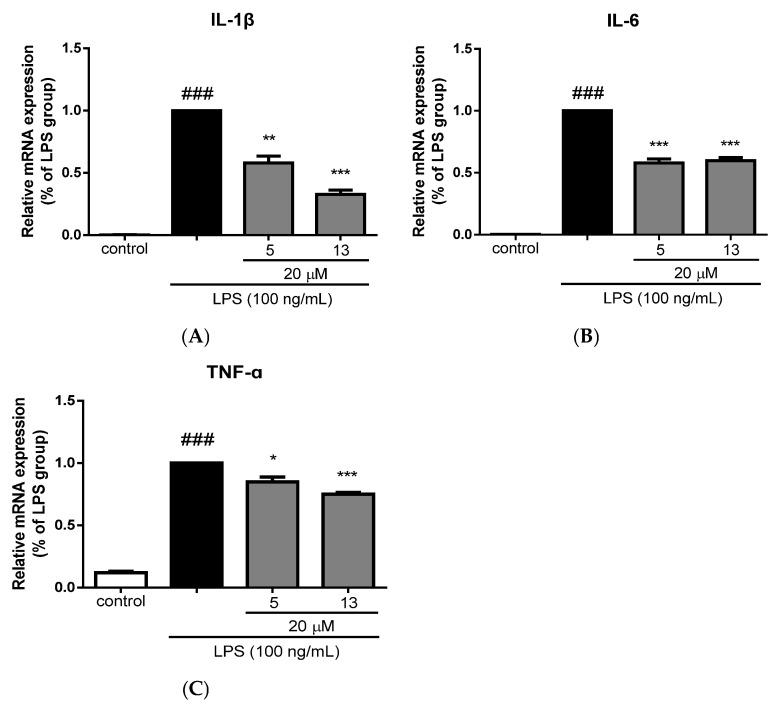
Inhibitory effects of compounds **5** and **13** on mRNA levels of IL-1*β* (**A**), IL-6 (**B**), and TNF-α (**C**) in BV-2 cells stimulated by LPS. Data were normalized by the LPS group and presented as means ± SEM, *n* = 3. ^###^ *p* < 0.001 vs. the control group; * *p* < 0.05, ** *p* < 0.01, *** *p* < 0.001 compared to the LPS group.

The anti-neuroinflammatory activity of compounds **1**–**7**, **10**–**13**, **15**, **18**–**20**, and **22**–**24** was evaluated in vitro against lipopolysaccharide (LPS)-induced inflammation-related BV-2 microglial cells. Compounds **5**, **10**, **13**, **18**, **19**, and **20** at 20 μM exhibited obvious inhibitory activity on NO production against LPS-induced inflammation-related BV-2 microglial cells (Table 9). In addition, compounds **5** and **13** at 20 μM markedly reduced the mRNA levels of the pro-inflammatory cytokines IL-1*β*, IL-6, and TNF-α in LPS-stimulated BV-2 microglial cells (Figure 7), which further demonstrated their inhibitory activities against microglial inflammation.

## 3. Discussion

In summary, the systematic investigation of *C. giraldii* led to the first report about the isolation of 24 phyllocladane-type diterpenoids, including 18 new derivatives, from the title plant. Among the new compounds, **15**–**22** were unusual 3,4-*seco* phyllocladane-type diterpenoids, while **23** and **24** were identified as two new phyllocladane-type diterpenoids dimers. Notably, unit B of **24** possessed the first identified 2,3-*seco* phyllocladane fragment.

By analyzing NMR data of the isolated compounds in this study and those reported in previous literature, spectroscopic rules were refined for distinguishing the phyllocladane- from the *ent*-kaurane type diterpenoids based on the chemical shifts of C-14, C-15, and C-20. The chemical shift of C-17 could be used to identify the stereochemistry of C-16. These rules were also applicable to 3,4-*seco* phyllocladane-type diterpenoids. The reliability of these rules was further confirmed by the single-crystal X-ray diffraction experiment results of new compounds **7**, **10**, **11**, **12**, **13**, and **15** in this study.

In comparing carbon NMR data of 3,4-*seco* and typical phyllocladane-type diterpenoids, it was found that the C-20 chemical shift in 3,4-*seco* phyllocladane-type diterpenoids (with unchanged H-5 relative configuration after ring opening) was between 18 and 20 ppm. However, for (5*β*,16*α*)-4,16,17-trihydroxy-3,4-seco-phyllocladan-3-oic acid, where the H-5 relative configuration is reversed, the C-20 chemical shift was 17.4 ppm.

Compounds **5**, **10**, **13**, **18**, **19**, and **20** exhibited anti-neuroinflammatory activity, as evidenced by their downregulation of the pro-inflammatory cytokines IL-1*β*, IL-6, and TNF-*α* in LPS-stimulated murine microglial BV-2 cells at the tested concentrations.

## 4. Materials and Methods

### 4.1. General Experimental Procedures

Optical rotations were measured with a Rudolph Research Analytical Autopol VI automatic polarimeter (Hackettstown, NJ, USA). IR spectra were recorded with a Thermo Nicolet FTIR IS5 spectrometer (Thermo Fisher, Waltham, MA, USA). NMR spectra were recorded on Bruker Avance III-500, Bruker Avance III-600, or III-800 spectrometers. (BRUKER BIOSPIN AG, Fällanden, Switzerland). Chemical shifts were reported in ppm (*δ*) with coupling constants in hertz. HRESIMS data were acquired on a Waters Synapt G2 Si Q-TOf mass detector. Single-crystal X-ray diffraction measurements were conducted on a Bruker Smart Apex II diffractometer with a graphite monochromator. LCESIMS analysis was performed on a Waters 2695 system equipped with a 2998 PDA detector, a Waters 2424 ELSD, and Waters 3100 MS detectors. Preparative HPLC was run on a Waters system equipped with a Waters 2767 autosampler, a Waters 2545 pump, and a Waters 2489 PDA, using a Waters Sunfire RP C18 column (5 μm, 30 × 150 mm, flow rate 30 mL/min). AB-8 macroporous resin (Shandong Lu Kang Chemical Co., Ltd., Jining, China). MCI gel CHP20P (75–150 μm, Mitsubishi Chemical Industries, Tokyo, Japan). ODS gel AAG12S50 (12 nm, s-50 μm, YMC Co., Ltd., Kyoto, Japan). Sephadex LH-20 (Pharmacia Biotech AB, Uppsala, Sweden), Toyopearl HW-40F (30–60 2 μm, TOSOH corporation, Tokyo, Japan). Silica gel (200–300 meshe, and 300–400 mesh, Qingdao Marine Chemical Inc. Qingdao, China). All solvents used for CC were of analytical grade (Shanghai Chemical Reagents Co., Ltd., Shanghai, China), and solvents used for HPLC were of HPLC grade (Merck KGaA, Darmstadt, Germany).

### 4.2. Plant Material

The branches and leaves of *C. giraldii* were collected from Jinghong City in Xishuangbanna Prefecture, Yunnan Province, China, in January 2020 and identified by Jun Zhang, an associate researcher from Kunming Zhifen Biotechnology Co., Ltd. (Kunming, China) A voucher (No. 20200115001) was deposited at the Herbarium of the Shanghai Institute of Materia Medica, Chinese Academy of Sciences.

### 4.3. Extraction and Isolation

The dried branches and leaves of *C. giraldii* (30 kg) were extracted with 95% ethanol (3 × 60 L, 7 days each) at room temperature. The ethanol extracts were concentrated under reduced pressure to obtain 831.5 g of extract. The extract was suspended in water and partitioned successively with petroleum ether (PE), dichloromethane (DCM), and ethyl acetate, yielding a PE extract (228.9 g), a DCM extract (260.6 g), an EA extract (68.5 g), and a water-soluble fraction. Then, the PE fraction was partitioned with 70% aqueous methanol to afford a 70% methanol fraction (53.2 g). LC-MS and TLC analyses indicated that the DCM extract and the 70% methanol extract showed high similarity in the chemical compositions, so these two extracts were combined to give a new fraction (313.8 g). The new fraction was subject to column chromatography (CC) over AB-8 macroporous resin, eluted with aqueous EtOH (20%, 50%, 75%, 95%) in a stepwise manner, then with acetone, finally yielding five subfractions Frs. A-E.

Fr. C (110.7 g) was applied to an MCI gel column, eluted with a gradient system of aqueous EtOH from 25% to 75%, resulting in subfractions Fr. C1–Fr. C14. Combined Fr. C6 and Fr. C7 (total 2.7 g) were subjected to CC over Sephadex LH-20 gel eluting with MeOH, and subsequently to CC over HW-40F gel eluting with MeOH, then purified by preparative HPLC to give compounds **15** (6.0 mg) and **16** (3.3 mg) (MeCN/H_2_O with 0.1% formic acid from 30 to 50%, 25 min).

Fr. C8 (9.4 g) was fractionated using an ODS gel column eluted with a gradient elution of aqueous MeCN (32–95%) to afford subfractions Fr. C8A–Fr. C8H. Fr. C8H (1.2 g) was subsequently separated by chromatography on Sephadex LH-20 gel eluted with MeOH to afford Fr. C8H1–Fr. C8H7. Fr. C8H3 (286.7 mg) was purified by preparative HPLC to afford compound **21** (10.0 mg, MeCN/H_2_O with 0.1% formic acid, from 23 to 43%, 25 min). Fr. C8H4 (759.7 mg) was separated by using a HW-40F column (eluted with MeOH) and further purified by preparative HPLC to give compound **2** (2.3 mg, MeCN/H_2_O with 0.1% formic acid, from 21 to 41%, 25 min). Fr. C8H4E (190.3 mg) was subjected to CC over silica gel (200–300 mesh, CH_2_Cl_2_/MeOH, gradient from 200:1 to 30:1) and then purified by preparative HPLC (MeCN/H_2_O with 0.1% formic acid, from 25% to 45%, 25 min) to give compound **9** (2.3 mg).

Fr. C10 (6.0 g) was applied for CC over Sephadex LH-20 gel eluted with CHCl_3_: MeOH = 1:1, yielding seven subfractions Fr. C10A- Fr. C10G. Fr. C10B (596.9 mg) was separated by using a HW-40F gel column eluted with MeOH and finally preparative HPLC (MeCN/H_2_O with 0.1% formic acid, from 34 to 59%, 25 min) to afford compound **22** (9.9 mg). Fr. C10C (773.8 mg) was also separated by using a HW-40F gel column (MeOH), and then purified by silica gel CC (200–300 mesh), with isocratic elution of CH_2_Cl_2_/MeOH (180:1–50:1) and then purified by preparative HPLC to give compounds **4** (22.1 mg) and **7** (2.9 mg) (MeCN/H_2_O with 0.1% formic acid, from 39 to 64%, 25 min).

Fr. C10D (3.3 g) was subjected to CC over HW-40F gel eluted with MeOH to afford Fr. C10D6 (678.0 mg), and then was treated with CC over HW-40F eluted with MeOH again to yield Fr. C10D6A–Fr. C10D6H. Fr. C10D6C (44.0 mg) was purified by preparative HPLC to give compound **19** (5.4 mg, MeCN/H_2_O with 0.1% formic acid, from 38 to 63%, 25 min). Fr. C10D6D (381.0 mg) was subjected to silica gel CC (200–300 mesh) with isocratic elution of CH_2_Cl_2_/acetone (150:1) and then purified by preparative HPLC to afford compounds **8** (1.4 mg) and **17** (2.2 mg) (MeCN/H_2_O with 0.1% formic acid, from 30 to 50%, 46–66%, 25 min, respectively).

Fr. C11 (13.3 g) was treated with CC over Sephadex LH-20 gel eluted with CHCl_3_: MeOH = 1:1 to yield Fr. C11A- Fr. C11H, which was further separated by CC over ODS gel (MeCN/H_2_O with 0.1% formic acid, from 30% to 95%) to give a subfraction of Fr. C11C2 (245.9 mg). Compounds **1** (44.3 mg) and **3** (9.8 mg) were obtained from Fr. C11C2 by preparative HPLC (MeCN/H_2_O with 0.1% formic acid, from 32 to 52%, 44 to 64%, 25 min, respectively). Fr. C11C7 (2.8 g) was subjected to a CC over silica gel (200–300 mesh, CH_2_Cl_2_/acetone, from 30:1 to 1:1) and separated by CC over silica gel (200–300 mesh, CH_2_Cl_2_/MeOH, from 150:1 to 30:1) and then purified by preparative HPLC (MeCN/H_2_O with 0.1% formic acid, from 42 to 62%, 25 min) to afford compound **20** (5.3 mg).

Fr. C12 (25.1 g) was separated by CC over ODS gel (MeCN/H_2_O with 0.1% formic acid, from 20% to 95%) to give 18 subfractions of Fr. C12A-Fr. C12R. Compounds **5** (37.3 mg), **6** (8.3 mg), **18** (5.4 mg), **23** (22.8 mg), and **24** (6.6 mg) were obtained by CC over a HW-40F gel (MeOH) and then preparative HPLC (MeCN/H_2_O with 0.1% formic acid, from 47 to 67%, 47–67%, 45–65%, 46–66%, 46–66%, 25 min, respectively) from Fr. C12J (2.2 g). Fr. C12L (1.1 g) was further purified by CC over Sephadex LH-20 eluted with MeOH, resulting in subfractions Frs. C12L1-7. Fr. C12L5 (281.7 mg) was subjected to separation by chromatography on HW-40F gel eluted with MeOH to afford Fr. C12L5F and Fr. C12L5J. Fr. C12L5F (66.1 mg) was purified by a silica gel column (200–300 mesh) using a gradient solvent system of CH_2_Cl_2_/MeOH (200:1–30:1) and then purified by preparative HPLC to afford compound **12** (3.1 mg) (MeCN/H_2_O with 0.1% formic acid, from 51 to 71%, 25 min). Compound **11** (1.5 mg) was isolated from Fr. C12L5J (25.5 mg) by preparative HPLC (MeCN/H_2_O with 0.1% formic acid, from 49 to 69%, 25 min). Fr. C12M (2.2 g) was separated by chromatography on Sephadex LH-20 gel eluted with MeOH to afford nine subfractions of Fr. C12M1-Fr. C12M9. Compounds **10** (65.1 mg) and **13** (7.3 mg) were isolated from Fr. C12M6 (665.7 mg) by repeated CC over silica gel (200–300 mesh, PE/acetone, 30:1–1:1) and then preparative HPLC (MeCN/H_2_O with 0.1% formic acid, from 50 to 70%, 51 to 71%, 25 min, respectively). In a similar way, compound **14** (1.0 mg) was isolated from Fr. C12M6E (104.0 mg) by repeated CC over silica gel (200–300 mesh, PE/acetone, from 30:1 to 1:1) and finally preparative HPLC (MeCN/H_2_O with 0.1% formic acid, from 52 to 72%, 25 min).

#### 4.3.1. Calligirlin A (**7**)

Colorless needles crystal from acetone; m.p. 159–161 °C; [α${]}_{D}^{20}$+ 5 (c 0.1, MeOH); IR (KBr) v_max_ 3428, 2929, 2848, 1713, 1456, 1373, 1278, 1260, 1225, 1080, 1038 cm^−1^; ^1^H and ^13^C NMR, see Table 2 and Table 3; HRESIMS *m*/*z* 387.2498 ([M + Na]^+^, calcd for C_22_H_36_O_4_Na, 387.2511).

#### 4.3.2. Calligirlin B (**8**)

White solid; [*α*${]}_{D}^{20}$ + 24 (*c* 0.1, MeOH); IR (KBr) *v*_max_ 3387, 2935, 2849, 1722, 1698, 1455, 1385, 1244, 1183, 1156, 1095, 1077, 1038 cm^−1^; ^1^H and ^13^C NMR, see Table 2 and Table 3; HRESIMS *m*/*z* 401.2286 ([M + Na]^+^, calcd for C_22_H_34_O_5_Na, 401.2304).

#### 4.3.3. Calligirlin C (**9**)

White amorphous powder; [*α*${]}_{D}^{20}$ + 17 (*c* 0.1, MeOH); IR (KBr) *v*_max_ 3416, 2931, 2862, 1668, 1456, 1383, 1261, 1106, 1073, 1042 cm^−1^; ^1^H and ^13^C NMR, see Table 2 and Table 3; HRESIMS *m*/*z* 319.2276 ([M + H]^+^, calcd for C_20_H_31_O_3_, 319.2273).

#### 4.3.4. Calligirlin D (**10**)

Colorless orthorhombic crystals from methanol; m.p. 134–136 °C; [*α*${]}_{D}^{20}$ + 50 (*c* 0.1, MeOH); IR (KBr) *v*_max_ 3443, 2928, 2852, 1702, 1457, 1384, 1115, 1041 cm^−1^; ^1^H and ^13^C NMR, see Table 2 and Table 3; HRESIMS *m*/*z* 305.2484 ([M + H]^+^, calcd for C_20_H_33_O_2_, 305.2481).

#### 4.3.5. Calligirlin E (**11**)

Light yellow needles crystal from acetone; m.p. 211–213 °C; [*α*${]}_{D}^{20}$ + 50 (*c* 0.1, MeOH); IR (KBr) *v*_max_ 2932, 2865, 1798, 1703, 1459, 1385, 1173, 1155, 1123, 1056 cm^−1^; ^1^H and ^13^C NMR, see Table 3 and Table 4; HRESIMS *m*/*z* 369.2033 ([M + Na]^+^, calcd for C_21_H_30_O_4_Na, 369.2042).

#### 4.3.6. Calligirlin F (**12**)

Colorless needles crystal from acetone; m.p. 183–185 °C; [*α*${]}_{D}^{20}$ + 24 (*c* 0.1, MeOH); IR (KBr) *v*_max_ 2929, 2854, 1073, 1458, 1385, 1260, 1234, 1182, 1150, 1115, 1039 cm^−1^; ^1^H and ^13^C NMR, see Table 3 and Table 4; HRESIMS *m*/*z* 319.2280 ([M + H]^+^, calcd for C_20_H_31_O_3_, 319.2273).

#### 4.3.7. Calligirlin G (**13**)

Colorless needles crystal from acetone; mp 174–176 °C; [*α*${]}_{D}^{20}$ − 84 (*c* 0.1, MeOH); IR (KBr) *v*_max_ 3355, 2937, 2858, 1671, 1384, 1045, 1017 cm^−1^; ^1^H and ^13^C NMR, see Table 3 and Table 4; HRESIMS *m*/*z* 303.2336 ([M + H]^+^, calcd for C_20_H_31_O_2_, 303.2324).

#### 4.3.8. Calligirlin H (**14**)

White solid; [*α*${]}_{D}^{20}$ + 47 (*c* 0.1, MeOH); IR (KBr) *v*_max_ 2927, 2869, 1703, 1456, 1384, 1261, 1102, 1023 cm^−1^; ^1^H and ^13^C NMR, see Table 3 and Table 4; HRESIMS *m*/*z* 319.2277 ([M + H]^+^, calcd for C_20_H_31_O_3_, 319.2273).

#### 4.3.9. Calligirlin I (**15**)

Colorless needles crystal from methanol; m.p. 107–109 °C; [*α*${]}_{D}^{20}$ + 41 (*c* 0.1, MeOH); IR (KBr) *v*_max_ 3419, 2924, 2863, 1707, 1457, 1384, 1303, 1262, 1029 cm^−1^; ^1^H and ^13^C NMR, see Table 5 and Table 6; HRESIMS *m*/*z* 361.2355 ([M + Na]^+^, calcd for C_20_H_34_O_4_Na, 361.2355).

#### 4.3.10. Calligirlin J (**16**)

White solid; [*α*${]}_{D}^{20}$ + 40 (*c* 0.1, MeOH); IR (KBr) *v*_max_ 3442, 2925, 2856, 1707, 1637, 1454, 1383, 1261, 1181, 1071, 1037, 893, 803 cm^−1^; ^1^H and ^13^C NMR, see Table 5 and Table 6; HRESIMS *m*/*z* 359.2187 ([M + Na]^+^, calcd for C_20_H_32_O_4_Na, 359.2198).

#### 4.3.11. Calligirlin K (**17**)

White solid; [*α*${]}_{D}^{20}$ + 17 (*c* 0.1, MeOH); IR (KBr) *v*_max_ 3461, 2930, 2863, 1730, 1637, 1454, 1384, 1242, 1102, 1039, 925, 894, 737 cm^−1^; ^1^H and ^13^C NMR, see Table 5 and Table 6; HRESIMS *m*/*z* 401.2293 ([M + Na]^+^, calcd for C_22_H_34_O_5_Na, 401.2304).

#### 4.3.12. Calligirlin L (**18**)

White solid; [*α*${]}_{D}^{20}$ + 39 (*c* 0.1, MeOH); IR (KBr) *v*_max_ 3417, 2930, 2864, 1734, 1453, 1382, 1301, 1260, 1207, 1179, 1118, 1072, 1033 cm^−1^; ^1^H and ^13^C NMR, see Table 5 and Table 6; HRESIMS *m*/*z* 387.2503 ([M + Na]^+^, calcd for C_22_H_36_O_4_Na, 387.2511).

#### 4.3.13. Calligirlin M (**19**)

White solid; [*α*${]}_{D}^{20}$ + 21 (*c* 0.1, MeOH); IR (KBr) *v*_max_ 3443, 2930, 2865, 1714, 1456, 1384, 1259, 1039 cm^−1^; ^1^H and ^13^C NMR, see Table 6 and Table 7; HRESIMS *m*/*z* 403.2452 ([M + Na]^+^, calcd for C_22_H_36_O_5_Na, 403.2460).

#### 4.3.14. Calligirlin N (**20**)

White solid; [*α*${]}_{D}^{20}$ + 46 (*c* 0.1, MeOH); IR (KBr) *v*_max_ 3406, 2931, 2865, 1736, 1458, 1383, 1304, 1279, 1259, 1181, 1156, 1125, 1032 cm^−1^; ^1^H and ^13^C NMR, see Table 6 and Table 7; HRESIMS *m*/*z* 389.2657 ([M + Na]^+^, calcd for C_22_H_38_O_4_Na, 389.2668).

#### 4.3.15. Calligirlin O (**21**)

White solid; [*α*${]}_{D}^{20}$ + 22 (*c* 0.1, MeOH); IR (KBr) *v*_max_ 3442, 2931, 2862, 1714, 1455, 1383, 1301, 1261, 1181, 1151, 1098, 1035 cm^−1^; ^1^H and ^13^C NMR, see Table 6 and Table 7; HRESIMS *m*/*z* 405.2615 ([M + Na]^+^, calcd for C_22_H_38_O_5_Na, 405.2617).

#### 4.3.16. Calligirlin P (**22**)

White solid; [*α*${]}_{D}^{20}$ + 34 (*c* 0.1, MeOH); IR (KBr) *v*_max_ 3479, 2935, 2864, 1732, 1455, 1385, 1259, 1150, 1102, 1037 cm^−1^; ^1^H and ^13^C NMR, see Table 6 and Table 7; HRESIMS *m*/*z* 447.2723 ([M + Na]^+^, calcd for C_24_H_40_O_6_Na, 447.2723).

#### 4.3.17. Calligirlin Q (**23**)

White solid; [*α*${]}_{D}^{20}$ + 36 (*c* 0.1, MeOH); IR (KBr) *v*_max_ 3440, 2930, 2860, 1704, 1457, 1384, 1301, 1263, 1181, 1150, 1102, 1073, 1036, 737 cm^−1^; ^1^H and ^13^C NMR, see Table 8; HRESIMS *m*/*z* 679.4544 ([M + Na]^+^, calcd for C_40_H_64_O_7_Na, 679.4450).

#### 4.3.18. Calligirlin R (**24**)

White solid; [*α*${]}_{D}^{20}$ + 22 (*c* 0.1, MeOH); IR (KBr) *v*_max_ 3459, 2928, 2861, 1713, 1457, 1387, 1301, 1262, 1192, 1146, 1102, 1072, 1037, 737 cm^−1^; ^1^H and ^13^C NMR, see Table 8; HRESIMS *m*/*z* 693.4337 ([M + Na]^+^, calcd for C_40_H_62_O_8_Na, 693.4342).

### 4.4. Computational Section

The DFT NMR calculations and TDDFT ECD calculations were performed with the Gaussian 16 program [23]. The conformational searching was conducted by the Conflex 8.0 software using the MMFF (Merck Molecular Force Field) force field within an energy window of 5.0 kcal/mol [24]. The conformers with the Boltzmann population above 1.0% were selected for re-optimization at the B3LYP/6-31G(d) level in vacuo. DFT NMR calculations were run at the level of mPW1PW91/6-311G (d,p) with the PCM solvent mode for chloroform. A possible configuration was specified using DP4+ probability analysis [21]. TDDFT ECD calculations were run at the CAM-B3LYP/TZVP level after re-optimization at the M06-2X/6-31G(d) level, both using the SMD solvent model for methanol. Calculated ECD spectra were generated using the SpecDis Version 17.1 [22,25].

### 4.5. X-Ray Crystallographic Analyses

Crystals of Compounds **1**, **2**, **5**, **7**, **10**, **11**, **12**, **13**, and **15** were obtained from their methanol or acetone solutions, respectively. Suitable crystals were selected for the X-ray crystallographic analysis. With the use of the Bruker SHELXTL (2014) software package, the structure was settled and refined. Copies of crystallographic data of every crystal can be obtained free of charge via the Internet at www.ccdc.cam.ac.uk/conts/retrieving.html (accessed on 9–11 August 2025) or on application to the CCDC, 12 Union Road, Cambridge CB2 1EZ, UK [tel: (+44) 1223-336-408; fax: (+44) 1223-336-033; e-mail: deposit@ccdc.cam.ac.uk].

#### 4.5.1. Crystal Data for Compound **1**

C_40_H_66_O_7_ (*M* = 658.92 g/mol): monoclinic, space group C2 (no. 5), *a* = 13.3789(4) Å, *b* = 6.2918(2) Å, *c* = 20.9753(7) Å, *β* = 101.6040(10)°, *V* = 1729.56(10) Å3, *Z* = 2, *T* = 100 K, *μ*(Cu K*α*) = 0.667 mm^−1^, *D_calc_* = 1.265 g/cm^3^, 13502 reflections measured (4.3° ≤ 2Θ ≤ 148.814°), 3462 unique (*R_int_* = 0.0793, *R_sigma_* = 0.0635) which were used in all calculations. The final *R*_1_ was 0.0452 (I > 2σ(I)) and *wR*_2_ was 0.1185. Flack parameter: 0.08(11). Crystallographic data for **1** have been deposited at the Cambridge Crystallographic Data Centre with deposit no. CCDC 2389726.

#### 4.5.2. Crystal Data for Compound **2**

C_40_H_70_O_7_ (*M* = 662.96 g/mol): monoclinic, space group C2 (no. 5), *a* = 12.9964(8) Å, *b* = 6.2092(4) Å, *c* = 25.0541(15) Å, *β* = 96.763(3)°, *V* = 2007.7(2) Å^3^, *Z* = 2, *T* = 170 K, *μ*(Cu K*α*) = 0.575 mm^−1^, *D_calc_* = 1.097 g/cm^3^, 17974 reflections measured (3.552° ≤ 2Θ ≤ 136.474°), 3673 unique (*R*_int_ = 0.0905, *R_sigma_* = 0.0680) which were used in all calculations. The final *R*_1_ was 0.0635 (I > 2σ(I)) and *wR*_2_ was 0.1774. Flack parameter:0.16. Crystallographic data for **2** have been deposited at the Cambridge Crystallographic Data Centre with deposit no. CCDC 2389913.

#### 4.5.3. Crystal Data for Compound **5**

C_19_H_28_O_2_ (*M* = 288.41 g/mol): orthorhombic, space group *P*2_1_2_1_2_1_ (no. 19), *a* = 7.4821(2) Å, *b* = 10.3278(3) Å, *c* = 20.0847 [18] Å, *V* = 1552.02(8) Å^3^, *Z* = 4, *T* = 170 K, *μ*(Cu K*α*) = 0.602 mm^−1^, *D_calc_* = 1.234 g/cm^3^, 19921 reflections measured (8.806° ≤ 2Θ ≤ 140.104°), 2942 unique (*R*_int_ = 0.0673, *R_sigma_* = 0.0390) which were used in all calculations. The final *R*_1_ was 0.0404 (I > 2σ(I)) and *wR*_2_ was 0.1038. Flack parameter: 0.04(13). Crystallographic data for **5** have been deposited at the Cambridge Crystallographic Data Centre with deposit no. CCDC 2389917.

#### 4.5.4. Crystal Data for Compound **7**

C_22_H_36_O_4_ (*M* = 364.51 g/mol): monoclinic, space group *P*2_1_ (no. 4), *a* = 7.3566(3) Å, *b* = 19.9404(8) Å, *c* = 7.4739(3) Å, *β* = 116.2340(10)°, *V* = 983.44(7) Å^3^, *Z* = 2, *T* = 150 K, *μ*(Cu K*α*) = 0.654 mm^−1^, *D_calc_* = 1.231 g/cm^3^, 18434 reflections measured (13.206° ≤ 2Θ ≤ 149.058°), 3975 unique (*R*_int_ = 0.0681, R_sigma_ = 0.0487) which were used in all calculations. The final *R*_1_ was 0.0444 (I > 2σ(I)) and *wR*_2_ was 0.1148. Flack parameter:0.03(10). Crystallographic data for **7** have been deposited at the Cambridge Crystallographic Data Centre with deposit no. CCDC 2389914.

#### 4.5.5. Crystal Data for Compound **10**

C_20_H_32_O_2_ (*M* = 304.45 g/mol): triclinic, space group *P*1 (no. 1), *a* = 6.4804(2) Å, *b* = 7.3872(3) Å, *c* = 10.3910(4) Å, *α* = 103.846(2)°, *β* = 96.885(2)°, *γ* = 115.760(2)°, *V* = 420.49(3) Å3, *Z* = 1, *T* = 150 K, *μ*(Cu K*α*) = 0.577 mm^−1^, *D_calc_* = 1.202 g/cm^3^, 21250 reflections measured (9.068° ≤ 2Θ ≤ 149.562°), 3284 unique (*R_int_* = 0.0689, *R_sigma_* = 0.0478) which were used in all calculations. The final *R*_1_ was 0.0407 (I > 2σ(I)) and *wR*_2_ was 0.1080. Flack parameter:0.05(13). Crystallographic data for **10** have been deposited at the Cambridge Crystallographic Data Centre with deposit no. CCDC 2389915.

#### 4.5.6. Crystal Data for Compound **11**

C_21_H_30_O_4_ (*M* = 346.45 g/mol): orthorhombic, space group *P*212121 (no. 19), *a* = 6.16210(10) Å, *b* = 9.2762(2) Å, *c* = 30.6471(7) Å, *V* = 1751.81 [18] Å3, *Z* = 4, *T* = 150 K, *μ*(Cu K*α*) = 0.713 mm^−1^, *D_calc_* = 1.314 g/cm^3^, 35528 reflections measured (5.768° ≤ 2Θ ≤ 149.2°), 3583 unique (*R_int_* = 0.0922, *R_sigma_* = 0.0445) which were used in all calculations. The final *R*_1_ was 0.0404 (I > 2σ(I)) and *wR*_2_ was 0.0942. Flack parameter: 0.12(12). Crystallographic data for **11** have been deposited at the Cambridge Crystallographic Data Centre with deposit no. CCDC 2390159.

#### 4.5.7. Crystal Data for Compound **12**

C_20_H_30_O_3_ (*M* = 318.44 g/mol): monoclinic, space group *P*21 (no. 4), *a* = 6.41320(10) Å, *b* = 19.0135(4) Å, *c* = 7.2649(2) Å, *β* = 105.8560(10)°, *V* = 852.16(3) Å3, *Z* = 2, *T* = 150.00 K, *μ*(Cu K*α*) = 0.641 mm^−1^, *D_calc_* = 1.241 g/cm^3^, 20082 reflections measured (9.302° ≤ 2Θ ≤ 149.252°), 3438 unique (*R_int_* = 0.0682, *R_sigma_* = 0.0459) which were used in all calculations. The final *R*_1_ was 0.0360 (I > 2σ(I)), and *wR*_2_ was 0.0943 (all data). Flack parameter: 0.08(10). Crystallographic data for **12** have been deposited at the Cambridge Crystallographic Data Centre with deposit no. CCDC 2389922.

#### 4.5.8. Crystal Data for Compound **13**

C_20_H_30_O_2_ (*M* = 302.44 g/mol): monoclinic, space group *P*21 (no. 4), *a* = 6.99180(10) Å, *b* = 23.8335(4) Å, *c* = 15.1339(3) Å, *β* = 91.7220(10)°, *V* = 2520.76(7) Å3, *Z* = 6, *T* = 170 K, *μ*(Cu K*α*) = 0.577 mm^−1^, *D_calc_* = 1.195 g/cm^3^, 49969 reflections measured (5.842° ≤ 2Θ ≤ 149.176°), 10263 unique (*R_int_* = 0.0869, *R_sigma_* = 0.0585) which were used in all calculations. The final *R*_1_ was 0.0424 (I > 2σ(I)), and *wR*_2_ was 0.1090 (all data). Flack parameter: −0.13(10). Crystallographic data for **13** have been deposited at the Cambridge Crystallographic Data Centre with deposit no. CCDC 2389916.

#### 4.5.9. Crystal Data for Compound **15**

C_21_H_38_O_5_ (*M* = 370.51 g/mol): triclinic, space group *P*1 (no. 1), *a* = 6.5343(2) Å, *b* = 7.1372(2) Å, *c* = 11.2987(3) Å, *α* = 84.2530(10)°, *β* = 82.0570(10)°, *γ* = 75.3600(10)°, *V* = 503.76(2) Å3, *Z* = 1, *T* = 100 K, *μ*(Cu Kα) = 0.681 mm^−1^, *D_calc_* = 1.221 g/cm^3^, 17254 reflections measured (7.918° ≤ 2Θ ≤ 148.822°), 3710 unique (*R_int_* = 0.0493, *R_sigma_* = 0.0445) which were used in all calculations. The final *R*_1_ was 0.0401 (I > 2σ(I)) and *wR*_2_ was 0.1058. Flack parameter: 0.05(8). Crystallographic data for **15** have been deposited at the Cambridge Crystallographic Data Centre with deposit no. CCDC 2389933.

### 4.6. Pharmacological Activity Assessment

The BV-2 cells (identifier: CSTR:19375.09.3101MOUGNM45) were a gift from Prof. L. Feng (Shanghai Institute of Materia Medica, Chinese Academy of Sciences), which was purchased from the National Collection of Authenticated Cell Cultures (Shanghai, China) and cultured in Dulbecco’s modified Eagle’s medium (DMEM, Life Technologies, Grand Island, NY, USA) supplemented with 10% fetal bovine serum (FBS, Gibco, Grand Island, NY, USA), 60 mg/L ampicillin sodium, and 50 mg/L streptomycin sulfate at 37 °C in a humidified incubator with 5% CO_2_.

The NO production was tested by the Griess reaction. Briefly, a density of 2 × 10 ^5^ cells/mL BV-2 cells was seeded per well in 96-well plates with a volume of 100 μL/well and cultured in a 37 °C constant-temperature incubator containing 5% CO_2_. After culturing for 24 h, the culture medium of BV-2 cells was replaced with fresh DMEM high-glucose medium containing 10% fetal bovine serum. The BV-2 cells were pretreated with test compounds (10 or 20 μM final concentration) or vehicle for 2 h, followed by LPS (100 ng/mL final concentration) exposure for 24 h. For the measurement of nitrite, 50 μL of culture medium taken from each well and 50 μL of Greiss buffer were mixed and incubated at room temperature for 15 min. The absorbance of each well was measured at 540 nm using a FlexStation 3 Microplate Reader, and the level of nitrite was calculated from a standard curve of sodium nitrite. The NO production in LPS was taken as 100%.

Quantitative real-time PCR (qRT–PCR) was employed to evaluate the mRNA levels of pro-inflammatory cytokines IL-1*β*, IL-6, and TNF-α. BV-2 microglial cells were seeded in a 12-well plate at a density of 2 × 10^5^ cells/mL and cultured overnight. The cells were then pretreated with 20 μM of compound **5** or compound **13** for 2 h and exposed to 100 ng/mL of LPS for 6 h. Total RNAs were extracted with Trizol reagent and reverse-transcribed with a PrimeScript RT Reagent Kit (Vazyme, Nanjing, China) to convert to cDNA. qRT-PCR assays were carried out with a real-time PCR detection system (Thermo Fisher Waltham, MA, USA) using a SYBR green kit (Vazyme, Nanjing, China). The gene expression values were normalized to those of GAPDH.

## 5. Conclusions

A systematic phytochemical investigation of *C. giraldii* led to the isolation and characterization of 18 new diterpenoids, including eight phyllocladane-type (**7**–**14**), eight 3,4-*seco* phyllocladane-type (**15**–**22**), and two unusual phyllocladane-type diterpene dimers (**23**, **24**), along with six known analogues. Through detailed analysis, empirical rules were further improved for distinguishing the phyllocladane-type from *ent*-kaurane type diterpenoids. Furthermore, anti-inflammatory evaluations revealed that compounds **5** and **13** exhibited significant anti-neuroinflammatory activity by reducing the expression levels of pro-inflammatory cytokines IL-1*β*, IL-6, and TNF-*α* in LPS-stimulated BV-2 microglial cells. These findings not only expand the chemical diversity of the genus *Callicarpa* but also provide potential lead compounds for further development of anti-neuroinflammatory agents.

## Figures and Tables

**Table 1:** Summary of the ^13^C NMR data differences of phyllocladane- and *ent*-kaurane-type diterpenoids.

	C-14	C-15	C-20	C-17
Phyllocladane-type	>40 ppm	<50 ppm	About 14–15 ppm	About 65–68 ppm, *rel*-16R; >69 ppm, *rel*-16S
*ent*-Kaurane-type	<40 ppm	>50 ppm	About 17 ppm	About 65–68 ppm, *rel*-16R; >69 ppm, *rel*-16S

**Table 2:** ^1^H NMR data of compounds **7**–**10** in CDCl_3_ (*δ* in ppm, *J* in Hz).

No.	7 ^a^	8 ^b^	9 ^a^	10 ^a^
1	0.81 (dd, 4.0, 13.1) 1.60 ^c^	0.86 (m) 1.69 ^c^	6.99 (d, 10.2)	1.37 (m) 1.90 (m)
2	1.38 (m, 2H)	1.40 (m) 1.82 (m)	5.83 (d, 10.2)	2.37 (m) 2.49 (m)
3	0.92 (m) 1.78 (m)	1.01 (td, 13.5, 4.2) 2.15 (d, 13.5)		
5	1.10 (m)	1.08 (m)	1.62 (m)	1.35 (m)
6	1.63 ^c^ (2H)	1.65 (m) 1.82 (m)	1.40 (m) 1.58 (m)	1.37 (m) 1.44 ^c^
7	1.49 (m) 1.67 (dt, 13.2, 3.2)	1.46 (m) 1.69 ^c^	1.59 (m) 1.74 ^c^	1.51 (m, 2H)
9	0.98 (m)	1.08 (m)	1.36 ^c^	1.09 (m)
11	1.20 (m) 1.56 ^c^	1.19 (m) 1.57 (m)	1.36 ^c^ 1.78 (m)	1.51 ^c^
12	1.43 (m) 1.59 ^c^	1.44 (m) 1.59 (m)	1.51 (m) 1.74 ^c^	1.44 ^c^ 1.55 (m)
13	1.90 (q, 3.9)	1.92 (m)	1.98 (q, 3,9)	1.94 (m)
14	1.07 (m) 2.09 (m)	1.07 (m) 2.09 (m)	1.16 (m) 2.14 (ddd, 11.5, 4.8, 2.5)	0.99 (m) 1.44 ^c^
15	1.23 (m) 2.07 (m)	1.29 (d, 14.6) 2.05 (m)	1.30 (d, 14.7) 2.05 (dd, 14.7, 2.0)	0.71 (dd, 13.5, 5.3) 2.20 (m)
16				1.94 (m)
17	4.16 (d, 11.3) 4.24 (d, 11.3)	4.16 (d, 11.3) 4.23 (d, 11.3)	3.65 (d, 10.9) 3.79 (d, 10.9)	3.39 (m, 2H)
18	3.41 (d, 10.9) 3.74 (d, 10.9)	1.23 (s, 3H)	1.13 (s, 3H)	1.06 (s, 3H)
19	0.96 (s, 3H)		1.08 (s, 3H)	1.01 (s, 3H)
20	0.85 (s, 3H)	0.80 (s, 3H)	1.09 (s, 3H)	1.01 (s, 3H)
OAc	2.10 (s, 3H)	2.10 (s, 3H)		

^a^ At 500 MHz, ^b^ At 600 MHz, ^c^ Overlapped.

**Table 3:** ^13^C NMR data of compounds **7**–**14** in CDCl_3_ (125 MHz, *δ* in ppm).

No.	7	8	9	10	11	12	13	14
1	39.5	39.8	158.2	38.6	38.3	38.4	36.9	38.1
2	18.1	19.0	125.8	34.2	34.0	34.2	27.4	34.0
3	35.6	38.0	205.3	218.2	217.2	218.1	79.0	216.5
4	38.6	44.0	44.7	47.5	47.5	47.6	39.0	47.7
5	57.0	57.1	53.5	55.6	55.2	55.4	55.1	55.6
6	20.5	22.0	20.7	21.5	20.3	21.4	19.8	19.7
7	41.8	41.5	40.9	40.6	39.8	40.5	36.1	37.9
8	43.9	43.7	44.2	44.4	44.3	43.6	49.7	36.4
9	56.9	56.2	50.8	56.0	55.2	56.7	53.9	50.5
10	37.8	38.4	39.9	37.3	37.1	37.3	37.3	37.3
11	19.5	19.6	19.7	20.0	21.3	19.7	19.0	25.2
12	26.9	26.9	26.7	32.6	26.5	28.8	24.7	216.5
13	44.7	44.6	44.1	46.2	43.8	47.9	36.4	50.3
14	48.4	48.1	48.1	47.8	48.4	51.0	54.0	55.9
15	44.8	44.6	45.2	36.7	47.0	33.4	156.9	37.8
16	82.8	82.8	84.5	36.9	93.7	38.5	147.6	31.3
17	68.0	67.9	65.6	67.6	70.7	179.3	190.0	22.9
18	65.8	29.2	28.4	26.9	26.9	26.9	28.4	26.4
19	27.3	181.9	21.9	21.6	21.7	21.7	15.8	21.7
20	15.5	13.5	18.2	15.2	15.0	15.0	15.6	13.7
OAc	171.4, C	171.5, C						
	21.1, CH_3_	21.1, CH_3_						
C=O					154.7, C			

**Table 4:** ^1^H NMR data of compounds **11**–**14** in CDCl_3_ (*δ* in ppm, *J* in Hz).

No.	11 ^a^	12 ^b^	13 ^b^	14 ^b^
1	1.42 (m), 1.87 (m)	1.89 (m) 2.58 ^c^	1.02 (m) 1.61 ^c^	1.36 ^c^ 1.81 (m)
2	2.40 (ddd, 15.9, 7.1, 4.1) 2.51 (m)	2.37 (ddd, 15.8,7.0, 4.0) 2.53 ^c^	1.51 (m) 1.62 ^c^	2.36 (m) 2.57 (ddd, 16.1, 12.2, 6.9)
3			3.21 (dd, 11.7, 4.1)	
5	1.39 (m)	1.38 ^c^	0.88 (dd, 11.6, 2.2)	1.36 ^c^
6	0.96 (m) 1.67 (m)	1.48 ^c^(2H)	1.47 ^c^, 1.64 (m)	1.51 (m, 2H)
7	1.59 (m) 1.72 (m)	1.50 ^c^ 1.63 (m)	1.42 (dd, 13.2, 4.2) 1.77 (dt, 13.4, 2.9)	1.25 ^c^ 1.60 (m)
9	1.19 (m)	1.14 (m)	1.18 ^c^	1.25 ^c^
11	1.36 (m) 1.56 (m)	1.49 ^c^(2H)	1.56 ^c^ 1.15 ^c^	1.64 (m) 1.84 ^c^
12	1.56 (m) 1.77 (m)	1.47 ^c^ 1.82 (m)	1.46 ^c^ 1.58 ^c^	
13	2.23 (m)	3.08 (dt, 12.1, 6.1)	2.88 (p, 2.8)	2.16 (m)
14	1.29 (dd, 11.7, 2.4) 1.98 (ddd, 11.7, 4.9, 2.4)	1.26 (dd, 11.1, 2.0) 1.60 (m)	1.27 (d, 10.3) 1.85 (ddd, 10.3, 5.4, 2.3)	1.84 ^c^ 1.90 (m)
15	1.90 (m) 2.28 (dd, 15.2, 2.4)	1.38 ^c^ (2H)	6.85 (s)	0.69 (dd, 14.0, 6.6) 2.36 (m)
16		2.47 (m)		2.00 (m)
17	4.35 (d, 8.8) 4.52 (d, 8.8)		9.73 (s)	0.90 (d, 7.0)
18	1.09 (s, 3H)	1.08 (s, 3H)	1.00 (s, 3H)	1.08 (s, 3H)
19	1.03 (s, 3H)	1.04 (s, 3H)	0.81 (s, 3H)	1.05 (s, 3H)
20	0.97 (s, 3H)	1.08 (s, 3H)	0.75 (s, 3H)	1.14 (s, 3H)

^a^ At 600 MHz, ^b^ At 500 MHz, ^c^ Overlapped.

**Table 5:** ^1^H NMR data of compounds **15**–**18** (*δ* in ppm, *J* in Hz).

No.	15 ^a^	16 ^a^	17 ^b^	18 ^c^
1	1.64 ^d^ (2H)	1.53 (m, 2H)	1.56 (m, 2H)	1.52 ^d^
2	2.15 (m, 2H)	2.19 (m) 2.33 (m)	2.22 (m) 2.37 (m)	2.18 (m) 2.32 (m)
4	1.91 ^d^			
5	1.10 (m)	2.06 (m)	1.97 (m)	1.98 (dd, 12.3, 2.8)
6	1.28 ^d^ 1.41 (m)	1.39 (m) 1.70 (m)	1.39 (m) 1.62 (m)	1.38 (m) 1.60 (m)
7	1.44 ^d^ 1.64 ^d^	1.55 (m, 2H)	1.50 (m) 1.59 (m)	1.52 ^d^ 1.58 (m)
9	1.28 ^d^	1.31 (m)	1.23 (m)	1.20 (m)
11	1.35 (m) 1.54 (m)	1.37 (m) 1.50 (m)	1.29 (m) 1.48 ^d^	1.27 (m) 1.49 ^d^
12	1.47 ^d^ 1.73 (m)	1.47 (m) 1.72 (m)	1.48 ^d^	1.44 (m) 1.68 (m)
13	1.89 (m)	1.91 (m)	1.92 (m)	1.92 (m)
14	1.10 ^d^ 2.10 (ddd, 11.1, 4.7, 2.4)	1.11 (d, 11.1) 2.10 (m)	1.10 (d, 11.1) 2.12 (m)	1.10 (dd, 11.5, 1.9), 2.06 ^d^
15	1.20 (d, 14.5) 2.00 (d, 14.5)	1.22 (d, 14.5) 2.05 (m)	1.31 (m) 2.07 (d, 15.7)	1.25 ^d^ 2.06 ^d^
17	3.57 (d, 11.3) 3.68 (d, 11.3)	3.58 (d, 11.2) 3.70 (d, 11.2)	4.18 (d, 11.2) 4.23 (d, 11.2)	3.62 (d, 11.0) 3.77 (d, 11.0)
18	0.93 (d, 6.9, 3H)	4.67 (s) 4.88 (s)	4.64 (s) 4.87 (s)	4.63 (s) 4.87 (s)
19	0.81 (d, 6.9, 3H)	1.75 (s, 3H)	1.73 (s, 3H)	1.72 (s, 3H)
20	0.87 (s, 3H)	0.90 (s, 3H)	0.87 (s, 3H)	0.86 (s, 3H)
OAc			2.10 (s, 3H)	
C_2_H_5_				4.10 (q, 7.1, 2H)
				1.24 (t, 7.1, 3H)

^a^ Measured in CD_3_OD, at 600 MHz. ^b^ Measured in CDCl_3,_ at 600 MHz. ^c^ Measured in CDCl_3_, at 500 MHz. ^d^ Overlapped.

**Table 6:** ^13^C NMR data of compounds **15**–**22** (*δ* in ppm).

No.	15 ^a^	16 ^a^	17 ^b^	18 ^b^	19 ^b^	20 ^b^	21 ^b^	22 ^c^
1	33.5	34.3	32.8	33.0	31.9	32.1	33.5	33.4
2	29.3	29.3	28.0	28.6	28.0	28.5	29.2	29.2
3	178.6	178.2	178.3	174.3	178.8	174.5	175.4	175.3
4	26.7	148.8	147.2	147.3	25.2	25.2	75.9	75.8
5	49.3	51.9	50.8	50.7	48.1	47.6	51.8	51.8
6	20.9	27.5	26.2	26.2	19.9	19.9	24.6	24.6
7	41.7	41.3	39.9	40.1	40.4	40.6	40.8	40.6
8	44.8	44.6	43.7	43.6	43.9	43.8	43.9	44.0
9	48.9	48.7,	47.6	47.5	47.6	47.6	48.4	48.4
10	41.1	40.6	39.6	39.7	40.4	40.3	41.8	41.8
11	21.0	20.9	19.8	19.9	19.9	19.9	19.2	19.1
12	27.9	27.8	26.9	26.9	27.0	27.0	27.1	27.1
13	44.9	44.8	44.6	44.0	44.6	44.0	43.9	44.5
14	49.5	49.2	48.2	48.4	48.3	48.5	48.2	48.1
15	45.2	45.1	44.5	44.7	44.6	44.7	44.5	44.5
16	85.6	85.6	82.7	84.5,	82.6	84.5	84.4	82.6
17	66.2	66.2	67.9	65.8	67.9	65.8	65.8	67.9
18	25.2	114.2	114.0	113.9	25.0	25.0	34.2	34.2
19	19.5	19.8	23.8	23.9	19.1	19.1	27.8	27.2
20	19.3	19.4	18.8	18.9	18.7	18.8	19.4	19.3
OAc			171.4, C		171.4, C			171.4, C
			21.1, CH_3_		21.1, CH_3_			21.1, CH_3_
C_2_H_5_				60.5, CH_2_		60.5, CH_2_	60.6, CH_2_	60.9, CH_2_
				14.4, CH_3_		14.4, CH_3_	14.4, CH_3_	14.4, CH_3_

^a^ Measured in CD_3_OD, at 125 MHz. ^b^ Measured in CDCl_3_, at 125 MHz. ^c^ Measured in CDCl_3_, at 200 MHz.

**Table 7:** ^1^H NMR data of compounds **19**–**22** in CDCl_3_ (*δ* in ppm, *J* in Hz).

No.	19 ^a^	20 ^a^	21 ^a^	22 ^b^
1	1.66 (m, 2H)	1.66 (m, 2H)	1.64 (m) 2.34 (ddd, 14.9, 10.7, 4.8)	1.61 ^c^ 2.33 (ddd, 15.2, 10.7, 4.8)
2	2.21 (m, 2H)	2.18 (m, 2H)	2.19 (ddd, 15.0, 10.7, 5.8) 2.45 (ddd, 15.3, 10.6, 4.7)	2.18 (ddd, 15.0, 10.7, 5.8) 2.45 (ddd, 15.3, 10.7, 4.8)
4	1.86 (p, 6.8)	1.88 (m)		
5	1.02 (m)	1.23 ^c^	1.36 ^c^	1.35 ^c^
6	1.20 ^c^ 1.42 (m)	1.23 ^c^	1.36 ^c^ 1.44 ^c^	1.35 ^c^ 1.44 (m)
7	1.42 (m) 1.63 (m)	1.42 ^c^ 1.63 (m)	1.44 ^c^ 1.59 (m)	1.41 (m) 1.60 ^c^
9	1.20 ^c^	1.23 ^c^	1.23 (m)	1.22 ^c^
11	1.25 ^c^ 1.51 (m)	1.42 ^c^ 1.54 (m)	1.22 ^c^ 1.56 (m)	1.22 ^c^ 1.55 ^c^
12	1.45 (m) 1.59 (m)	1.48 (m) 1.70 (m)	1.67 (m, 2H)	1.62 ^c^ (2H)
13	1.92 (m)	1.93 (m)	1.91 (q, 3.9)	1.91 (q, 3.8)
14	1.08 (d, 14.5) 2.09 (m)	1.10 (d, 11.2) 2.08 (m)	1.07 (m) 2.05 (m)	1.06 (m) 2.08 ^c^
15	1.28 (d, 11.2) 2.02 (m)	1.26 (m) 2.03 (m)	1.22 ^c^ 2.00 (m)	1.26 ^c^ 2.02 (m)
17	4.16 (d, 11.3) 4.23 (d, 11.3)	3.64 (m) 3.78 (m)	3.62 (d, 10.9) 3.76 (d, 10.9)	4.16 (d, 11.3) 4.22 (d, 11.3)
18	0.90 (d, 6.8, 3H)	0.92 (d, 6.8, 3H)	1.28 (s, 3H)	1.27 (s, 3H)
19	0.78 (d, 6.8, 3H)	0.80 (d, 6.8, 3H)	1.22 (s, 3H)	1.22 (s, 3H)
20	0.84 (s, 3H)	0.86 (s, 3H)	1.04 (s, 3H)	1.03 (s, 3H)
OAc	2.11 (s, 3H)			2.10 (s, 3H)
C_2_H_5_		4.14 (q, 7.1, 2H)	4.11 (q, 7.1, 2H)	4.11 (q, 7.1, 2H)
		1.28 (t, 7.1, 3H)	1.25 (t, 7.1, 3H)	1.25 (t, 7.1, 3H)

^a^ At 500 MHz, ^b^ At 800 MHz, ^c^ Overlapped.

**Table 8:** ^1^H and ^13^C NMR data of compounds **23** and **24** (*δ* in ppm, *J* in Hz).

No.	23		24	
	*δ*_C_ ^a^	*δ*_H_ mult (*J* in Hz) ^b^	* δ_C_* ^c^	*δ*_H_ mult (*J* in Hz) ^d^
1	38.3	1.37 * 1.85 (m)	39.2	1.45 * 1.92 *
2	34.1	2.36 * 2.50 *	34.9	2.40 (m) 2.55 (m)
3	217.9		220.4	
4	47.5		48.5	
5	55.4	1.36 *	56.4	1.47 *
6	21.4	1.35 * 1.49 (m)	22.4	1.43 * 1.54 *
7	40.6	1.54 (m) 1.70 (m)	41.7	1.71 *
8	43.7		44.9	
9	55.9	1.13 (dd, 12.3, 4.4)	57.1	1.23 (dd, 12.2, 4.7)
10	37.4		38.3	
11	19.7	1.28 * 1.55 *	20.8	1.41 * 1.61 *
12	26.9	1.45 * 1.61 *	27.8	1.51 * 1.68 *
13	44.6	1.94 (m)	44.7	1.90 (m)
14	48.0	1.09 (m) 2.12 (m)	49.1	1.13 (d, 11.1) 2.18 (m)
15	44.5	1.28 * 2.05 *	45.8	1.33 * 2.15 (m)
16	82.8		83.9	
17	67.6	4.19 (s, 2H)	69.0	4.02 (d, 11.3) 4.37 (d, 11.3)
18	26.9	1.06 (s, 3H)	27.3	1.07 (s, 3H)
19	21.7	1.01 (s, 3H)	22.1	1.04 (s, 3H)
20	14.8	0.97 (s, 3H)	15.3	1.02 (s, 3H)
1’	33.8	1.66 * 2.36 *	43.0	2.51 (m) 2.44 (m)
2’	29.2	2.20 * 2.51 *	175.8	
3’	175.5		180.7	
4’	76.1		miss	
5’	51.9	1.38 *	49.0	2.70 (m)
6’	24.6	1.45 * 1.35 *	24.8	1.44 * 1.56 *
7’	40.7	1.45 * 1.58 *	41.8	1.58 *
8’	43.8		44.9	
9’	48.5	1.19 *	50.4	2.00 *
10’	41.9		43.3	
11’	19.1	1.23 (m) 1.55 *	20.9	1.34 * 1.64 *
12’	27.0	1.45 * 1.69 *	21.9	1.43 * 1.75 *
13’	43.8	1.91 (m)	44.8	1.19 (d, 14.5) 1.99 *
14’	48.2	1.04 * 2.05 (m)	49.2	1.06 (d, 11.7) 2.07 (m)
15’	44.4	1.20 * 2.00 (m)	45.4	1.97 *
16’	84.4		85.4	
17’	65.7	3.61 (d, 10.9) 3.75 (d, 10.9)	66.2	3.57 (d, 11.3) 3.69 (d, 11.3)
18’	34.4	1.27 (s, 3H)	26.1	1.37 (s, 3H)
19’	27.6	1.21 (s, 3H)	25.7	1.27 (s, 3H)
20’	19.4	1.03 (s, 3H)	19.4	1.03 (s, 3H)

^a^ In CDCl_3_, at 125 MHz, ^b^ In CDCl_3_, at 500 MHz, ^c^ In CD_3_OD, at 150 MHz, ^d^ In CD_3_OD, at 600 MHz, * Overlapped.

**Table 9:** Anti-inflammatory effect of compounds **5**, **10**, **13**, **18**, **19**, and **20** in LPS-induced murine microglial BV-2 Cells ^a^.

No.	NO Production ± SEM (% of LPS Group)	
	10 μM	20 μM
**5**	95.70 ± 2.77	74.52 ± 4.25 **
**10**	96.95 ± 3.06	79.30 ± 0.74 ***
**13**	89.71 ± 0.89	73.11 ± 6.20 **
**18**	93.87 ± 2.71	79.84 ± 4.71 **
**19**	98.12 ± 1.15	88.82 ± 4.18 *
**20**	95.15 ± 2.71	80.13 ± 3.51 **

^a^ The NO production of the LPS alone group was set as 100%, and data are shown as mean ± SEM (*n* = 3). The NO production of the control group (without LPS exposure) was 1.40 ± 1.56% (*n* = 3). * *p* < 0.05, ** *p* < 0.01, *** *p* < 0.001 vs. LPS alone group.

## Data Availability

The original contributions presented in this study are included in the article/Supplementary Material. Further inquiries can be directed to the corresponding authors.

## Supplementary Materials

The following supporting information can be downloaded at https://www.mdpi.com/article/10.3390/molecules30071553/s1, Figure S1. The HRESIMS spectrum of compound **7**; Figure S2. IR spectrum of compound **7**; Figure S3. 1H NMR Spectrum of compound **7** in CDCl_3_; Figure S4. ^13^C NMR and DEPT 135 spectrum of compound **7** in CDCl_3_; Figure S5. ^1^H-^1^H COSY Spectrum of compound **7** in CDCl_3_; Figure S6. HSQC Spectrum of compound **7** in CDCl_3_; Figure S7. HMBC spectrum of compound **7** in CDCl3; Figure S8. NOESY Spectrum of compound **7** in CDCl_3_; Figure S9. Two possible conformations of compound **7**; Figure S10. DP4+ probability analysis results for compound **7** (7a: *rel*- 4*S*, 5*R*, 8*S*, 9*S*, 10*R*, 13*R*, 16*R*). Figure S11. HRESIMS Spectrum of compound **8**; Figure S12. IR spectrum of compound **8**; Figure S13. ^1^H NMR Spectrum of compound **8** in CDCl_3_; Figure S14. ^13^C NMR and DEPT 135 spectrum of compound **8** in CDCl_3_; Figure S15. ^1^H-^1^H COSY Spectrum of compound **8** in CDCl_3_; Figure S16. HSQC Spectrum of compound **8** in CDCl_3_; Figure S17. HMBC Spectrum of compound **8** in CDCl_3_; Figure S18. NOESY Spectrum of compound **8** in CDCl_3_; Figure S19. HRESIMS Spectrum of compound **9**; Figure S20. IR spectrum of compound **9**; Figure S21. ^1^H NMR Spectrum of compound **9** in CDCl_3_; Figure S22. ^13^C NMR and DEPT 135 spectrum of compound **9** in CDCl_3_; Figure S23. ^1^H-^1^H COSY Spectrum of compound **9** in CDCl_3_; Figure S24. HSQC Spectrum of compound **9** in CDCl_3_; Figure S25. HMBC Spectrum of compound **9** in CDCl_3_; Figure S26. NOESY Spectrum of compound **9** in CDCl_3_; Figure S27. HRESIMS Spectrum of compound **10**; Figure S28. IR spectrum of compound **10**; Figure S29. ^1^H NMR Spectrum of compound **10** in CDCl_3_; Figure S30. ^13^C NMR and DEPT 135 spectrum of compound **10** in CDCl_3_; Figure S31. ^1^H-^1^H COSY Spectrum of compound **10** in CDCl_3_; Figure S32. HSQC Spectrum of compound **10** in CDCl_3_; Figure S33. HMBC Spectrum of compound **10** in CDCl_3_; Figure S34. NOESY Spectrum of compound **10** in CDCl_3_; Figure S35. HRESIMS Spectrum of compound **11**; Figure S36. IR spectrum of compound **11**; Figure S37. ^1^H NMR Spectrum of compound **11** in CDCl_3_; Figure S38. ^13^C NMR and DEPT 135 spectrum of compound **11** in CDCl_3_; Figure S39. ^1^H-^1^H COSY Spectrum of compound **11** in CDCl_3_; Figure S40. HSQC Spectrum of compound **11** in CDCl_3_; Figure S41. HMBC Spectrum of compound **11** in CDCl_3_; Figure S42. NOESY Spectrum of compound **11** in CDCl_3_; Figure S43. HRESIMS Spectrum of compound **12**; Figure S44. IR spectrum of compound **12**; Figure S45. ^1^H NMR Spectrum of compound **12** in CDCl_3_; Figure S46. ^13^C NMR and DEPT 135 spectrum of compound **12** in CDCl_3_; Figure S47. ^1^H-^1^H COSY Spectrum of compound **12** in CDCl_3_; Figure S48. HSQC Spectrum of compound **12** in CDCl_3_; Figure S49. HMBC Spectrum of compound **12** in CDCl_3_; Figure S50. NOESY Spectrum of compound **12** in CDCl_3_; Figure S51. HRESIMS Spectrum of compound **13**; Figure S52. IR spectrum of compound **13**; Figure S53. ^1^H NMR Spectrum of compound **13** in CDCl_3_; Figure S54. ^13^C NMR and DEPT 135 spectrum of compound **13** in CDCl_3_; Figure S55. ^1^H-^1^H COSY Spectrum of compound **13** in CDCl_3_; Figure S56. HSQC Spectrum of compound **13** in CDCl_3_; Figure S57. HMBC Spectrum of compound **13** in CDCl_3_; Figure S58. NOESY Spectrum of compound **13** in CDCl_3_; Figure S59. HRESIMS Spectrum of compound **14**; Figure S60. IR spectrum of compound **14**; Figure S61. ^1^H NMR Spectrum of compound **14** in CDCl_3_; Figure S62. ^13^C NMR and DEPT 135 spectrum of compound **14** in CDCl_3_; Figure S63. ^1^H-^1^H COSY Spectrum of compound **14** in CDCl_3_; Figure S64. HSQC Spectrum of compound **14** in CDCl_3_; Figure S65. HMBC Spectrum of compound **14** in CDCl_3_; Figure S66. NOESY Spectrum of compound **14** in CDCl_3_; Figure S67. Two possible conformations of compound **14**; Figure S68. DP4+ probability analysis result for compound **14** (14a: *rel*- 5*R*, 8*R*, 9*S*, 10*R*, 13*R*, 16*S*); Figure S69. HRESIMS Spectrum of compound **15**; Figure S70. IR spectrum of compound **15**; Figure S71. ^1^H NMR Spectrum of compound **15** in CD_3_OD; Figure S72. ^13^C NMR and DEPT 135 spectrum of compound **15** in CD_3_OD; Figure S73. ^1^H-^1^H COSY Spectrum of compound **15**in CD_3_OD; Figure S74. HSQC Spectrum of compound **15** in CD_3_OD; Figure S75. HMBC Spectrum of compound **15** in CD_3_OD; Figure S76. NOESY Spectrum of compound **15** in CD_3_OD; Figure S77. HRESIMS Spectrum of compound **16**; Figure S78. IR spectrum of compound **16**; Figure S79. ^1^H NMR Spectrum of compound **16** in CD_3_OD; Figure S80. ^13^C NMR and DEPT 135 spectrum of compound **16** in CD_3_OD; Figure S81. ^1^H-^1^H COSY Spectrum of compound **16** in CD_3_OD; Figure S82. HSQC Spectrum of compound **16** in CD_3_OD; Figure S83. HMBC Spectrum of compound **16** in CD_3_OD; Figure S84. NOESY Spectrum of compound **16** in CD_3_OD; Figure S85. HRESIMS Spectrum of compound **17**; Figure S86. IR spectrum of compound **17**; Figure S87. ^1^H NMR Spectrum of compound **17** in CDCl_3_; Figure S88. ^13^C NMR and DEPT 135 spectrum of compound **17** in CDCl_3_; Figure S89. ^1^H-^1^H COSY Spectrum of compound **17** in CDCl_3_; Figure S90. HSQC Spectrum of compound **17** in CDCl_3_; Figure S91. HMBC Spectrum of compound **17** in CDCl_3_; Figure S92. NOESY Spectrum of compound **17** in CDCl_3_; Figure S93. HRESIMS Spectrum of compound **18**; Figure S94. IR spectrum of compound **18**; Figure S95. ^1^H NMR Spectrum of compound **18** in CDCl_3_; Figure S96. ^13^C NMR and DEPT 135 spectrum of compound **18** in CDCl_3_; Figure S97. ^1^H-^1^H COSY Spectrum of compound **18** in CDCl_3_; Figure S98. HSQC Spectrum of compound **18** in CDCl_3_; Figure S99. HMBC Spectrum of compound **18** in CDCl_3_; Figure S100. NOESY Spectrum of compound **18** in CDCl_3_; Figure S101. HRESIMS Spectrum of compound **19**; Figure S102. IR spectrum of compound **19**; Figure S103. ^1^H NMR Spectrum of compound **19** in CDCl_3_; Figure S104. ^13^C NMR and DEPT 135 spectrum of compound **19** in CDCl_3_; Figure S105. ^1^H-^1^H COSY Spectrum of compound **19** in CDCl_3_; Figure S106. HSQC Spectrum of compound **19** in CDCl_3_; Figure S107. HMBC Spectrum of compound **19** in CDCl_3_; Figure S108. NOESY Spectrum of compound **19** in CDCl_3_; Figure S109. HRESIMS Spectrum of compound **20**; Figure S110. IR spectrum of compound **20**; Figure S111. ^1^H NMR Spectrum of compound **20** in CDCl_3_; Figure S112. ^13^C NMR and DEPT 135 spectrum of compound **20** in CDCl_3_; Figure S113. ^1^H-^1^H COSY Spectrum of compound **20** in CDCl_3_; Figure S114. HSQC Spectrum of compound **20** in CDCl_3_; Figure S115. HMBC Spectrum of compound **20** in CDCl_3_; Figure S116. NOESY Spectrum of compound **20** in CDCl_3_; Figure S117. HRESIMS Spectrum of compound **21**; Figure S118. IR spectrum of compound **21**; Figure S119. ^1^H NMR Spectrum of compound **21** in CDCl_3_; Figure S120. ^13^C NMR and DEPT 135 spectrum of compound **21** in CDCl_3_; Figure S121. ^1^H-^1^H COSY Spectrum of compound **21** in CDCl_3_; Figure S122. HSQC Spectrum of compound **21** in CDCl_3_; Figure S123. HMBC Spectrum of compound **21** in CDCl_3_; Figure S124. NOESY Spectrum of compound **21** in CDCl_3_; Figure S125. HRESIMS Spectrum of compound **22**; Figure S126. IR spectrum of compound **22**; Figure S127. ^1^H NMR Spectrum of compound **22** in CDCl_3_; Figure S128. ^13^C NMR and DEPT 135 spectrum of compound **22** in CDCl_3_; Figure S129. ^1^H-^1^H COSY Spectrum of compound **22** in CDCl_3_; Figure S130. HSQC Spectrum of compound **22** in CDCl_3_; Figure S131. HMBC Spectrum of compound **22** in CDCl_3_; Figure S132. NOESY Spectrum of compound **22** in CDCl_3_; Figure S133. HRESIMS Spectrum of compound **23**; Figure S134. IR spectrum of compound **23**; Figure S135. ^1^H NMR Spectrum of compound **23** in CDCl_3_; Figure S136. ^13^C NMR and DEPT 135 spectrum of compound **23** in CDCl_3_; Figure S137. ^1^H-^1^H COSY Spectrum of compound **23** in CDCl_3_; Figure S138. HSQC Spectrum of compound **23** in CDCl_3_; Figure S139. HMBC Spectrum of compound **23** in CDCl_3_; Figure S140. NOESY Spectrum of compound **23** in CDCl_3_; Figure S141. HRESIMS Spectrum of compound **24**; Figure S142. IR spectrum of compound **24**; Figure S143. ^1^H NMR Spectrum of compound **24** in CD_3_OD; Figure S144. ^13^C NMR and DEPT 135 spectrum of compound **24** in CD_3_OD; Figure S145. ^1^H-^1^H COSY Spectrum of compound **24** in CD_3_OD; Figure S146. HSQC Spectrum of compound **24** in CD_3_OD. Figure S147. HMBC Spectrum of compound 24 in CD_3_OD. Figure S148. NOESY Spectrum of compound 24 in CD_3_OD.
