# Supplementary material for: Undescribed Phyllocladane-Type Diterpenoids from Callicarpa giraldii Hesse ex Rehd. and Their Anti-Neuroinflammatory Activity
Source: Molecules. 2025 Mar 31;30(7):1553. doi: 10.3390/molecules30071553 (PMC11990348; doi:10.3390/molecules30071553)
Supplement: Supplementary file 1 [file molecules-30-01553-s001.zip › molecules-3516771-supplementary.pdf]

**Supporting Information for**

# Undescribed Phyllocladane-Type Diterpenoids from *Callicarpa giraldii* Hesse ex Rehd. and Their Anti-Neuroinflammatory Activity

**Xu Liang [1,2,†], Qi Gong [3,†], Yuting Xu [4,5], Jiaxing Mu [2,6], Chunping Tang [2,7], Bintao Hu [2], Changqiang Ke [2],**
**Sheng Yao [2,3,5,7,*], Haiyan Zhang [3] and Yang Ye [1,2,6,7,*]**

[1] School of Chinese Materia Medica, Nanjing University of Chinese Medicine, Nanjing 210023, China; liangxu@simm.ac.cn

2 State Key Labo ratory of Drug Research, Natural Products Chemistry Department, Shanghai Institute of Materia Medica, Chinese Academy of Sciences, Shanghai 201203, China; mujiaxing@simm.ac.cn (J.M.); tangcp@simm.ac.cn (C.T.); 201728012342006@simm.ac.cn (B.H.); kechangqiang@simm.ac.cn (C.K.)

[3] State Key Laboratory of Drug Research, Center for Neurological and Psychiatric Research, Shanghai Institute of Materia Medica, Chinese Academy of Sciences, Shanghai 201203, China; gq1021@simm.ac.cn (Q.G.); hzhang@simm.ac.cn (H.Z.)

[4] School of Pharmaceutical Sciences, Southern Medical University, Guangzhou 510515, China; xuyuting551@zidd.ac.cn

[5] Zhongshan Institute for Drug Discovery, Shanghai Institute of Materia Medica, Chinese Academy of Sciences, Zhongshan 528400, China

[6] University of Chinese Academy of Sciences, Beijing 100049, China

[7] China-Serbia "Belt and Road" Joint Laboratory for Natural Products and Drug Discovery, Shanghai Institute of Materia Medica, Chinese Academy of Sciences, Shanghai 201203, China

* Correspondence: yaosheng@simm.ac.cn (S.Y.); yye@simm.ac.cn (Y.Y.)

† These authors contributed equally to this work.

# Table of Content


**Figure S1.** The HRESIMS spectrum of compound **7**. ........ 6

**Figure S2.** IR spectrum of compound **7**. ........ 6

**Figure S3a.** $^{1}H$ NMR spectrum of compound **7** in $CDCl_3$ ........ 7

**Figure S3b.** Partial $^{1}H$ NMR spectrum ($\delta$ 0.7-4.3 ppm) of compound **7** in $CDCl_3$ ........ 7

**Figure S3c.** Partial $^{1}H$ NMR spectrum ($\delta$ 0.8-2.2 ppm) of compound **7** in $CDCl_3$ ........ 8

**Figure S4.** $^{13}C$ NMR and DEPT 135 spectrum of compound **7** in $CDCl_3$ ........ 8

**Figure S5.** $^{1}H$-$^{1}H$ COSY spectrum of compound **7** in $CDCl_3$ ........ 9

**Figure S6**. HSQC spectrum of compound **7** in $CDCl_3$ ........ 9

**Figure S7.** HMBC spectrum of compound **7** in $CDCl_3$ ........ 10

**Figure S8.** NOESY spectrum of compound **7** in $CDCl_3$ ........ 10

**Figure S9.** Two possible conformations of compound **7** ........ 11

**Fig. S10** DP4+ probability analysis results for compound **7** (7a: *rel*- 4*S*, 5*R*, 8*S*, 9*S*, 10*R*, 13*R*, 16*R*) ........ 11

**Figure S11.** HRESIMS spectrum of compound **8** ........ 11

**Figure S12.** IR spectrum of compound **8** ........ 12

**Figure S13a.** $^{1}H$ NMR spectrum of compound **8** in $CDCl_3$ ........ 12

**Figure S13b.** Partial $^{1}H$ NMR spectrum ($\delta$ 0.0-4.4 ppm) of compound **8** in $CDCl_3$ ........ 13

**Figure S13c.** Partial $^{1}H$ NMR spectrum ($\delta$ 0.7-2.2 ppm) of compound **8** in $CDCl_3$ ........ 13

**Figure S14.** $^{13}C$ NMR and DEPT 135 spectrum of compound **8** in $CDCl_3$ ........ 14

**Figure S15.** $^{1}H$-$^{1}H$ COSY spectrum of compound **8** in $CDCl_3$ ........ 14

**Figure S16.** HSQC spectrum of compound **8** in $CDCl_3$ ........ 15

**Figure S17.** HMBC spectrum of compound **8** in $CDCl_3$ ........ 15

**Figure S18.** NOESY spectrum of compound **8** in $CDCl_3$ ........ 16

**Figure S19.** HRESIMS spectrum of compound **9** ........ 16

**Figure S20.** IR spectrum of compound **9** ........ 17

**Figure S21a.** $^{1}H$ NMR spectrum of compound **9** in $CDCl_3$ ........ 17

**Figure S21b.** Partial $^{1}H$ NMR spectrum ($\delta$ 0.7-7.2 ppm) of compound **9** in $CDCl_3$ ........ 18

**Figure S21c.** Partial $^{1}H$ NMR spectrum ($\delta$ 1.0-2.2 ppm) of compound **9** in $CDCl_3$ ........ 18

**Figure S22.** $^{13}C$ NMR and DEPT 135 spectrum of compound **9** in $CDCl_3$ ........ 19

**Figure S23.** $^{1}H$-$^{1}H$ COSY spectrum of compound **9** in $CDCl_3$ ........ 19

**Figure S24.** HSQC spectrum of compound **9** in $CDCl_3$ ........ 20

**Figure S25.** HMBC spectrum of compound **9** in $CDCl_3$ ........ 20

**Figure S26.** NOESY spectrum of compound **9** in $CDCl_3$ ........ 21

**Figure S27.** HRESIMS spectrum of compound **10** ........ 21

**Figure S28.** IR spectrum of compound **10** ........ 22

**Figure S29a.** $^1$H NMR spectrum of compound **10** in $CDCl_3$ ........................................................22
**Figure S29b.** Partial $^1$H NMR spectrum ($\delta$ 0.6-3.5 ppm) of compound **10** in $CDCl_3$...................23
**Figure S30.** $^{13}$C NMR and DEPT 135 spectrum of compound **10** in $CDCl_3$.................................23
**Figure S31.** $^1$H-$^1$H COSY spectrum of compound **10** in $CDCl_3$.....................................................24
**Figure S32.** HSQC spectrum of compound **10** in $CDCl_3$ .............................................................24
**Figure S33.** HMBC spectrum of compound **10** in $CDCl_3$ .............................................................25
**Figure S34.** NOESY spectrum of compound **10** in $CDCl_3$.............................................................25
**Figure S35.** HRESIMS spectrum of compound **11** .......................................................................26
**Figure S36.** IR spectrum of compound **11** .....................................................................................26
**Figure S37a.** $^1$H NMR spectrum of compound **11** in $CDCl_3$ ........................................................27
**Figure S37b.** Partial $^1$H NMR spectrum ($\delta$ 0.8-4.6 ppm) of compound **11** in $CDCl_3$...................27
**Figure S38.** $^{13}$C NMR and DEPT 135 spectrum of compound **11** in $CDCl_3$.................................28
**Figure S39.** $^1$H-$^1$H COSY spectrum of compound **11** in $CDCl_3$.....................................................29
**Figure S40.** HSQC spectrum of compound **11** in $CDCl_3$ .............................................................29
**Figure S41.** HMBC spectrum of compound **11** in $CDCl_3$ .............................................................30
**Figure S42.** NOESY spectrum of compound **11** in $CDCl_3$.............................................................30
**Figure S43.** HRESIMS spectrum of compound **12** .......................................................................31
**Figure S44.** IR spectrum of compound **12** .....................................................................................31
**Figure S45a.** $^1$H NMR spectrum of compound **12** in $CDCl_3$ ........................................................32
**Figure S45b.** Partial $^1$H NMR spectrum ($\delta$ 1.0-3.2 ppm) of compound **12** in $CDCl_3$...................32
**Figure S46.** $^{13}$C NMR and DEPT 135 spectrum of compound **12** in $CDCl_3$.................................33
**Figure S47.** $^1$H-$^1$H COSY spectrum of compound **12** in $CDCl_3$.....................................................33
**Figure S48.** HSQC spectrum of compound **12** in $CDCl_3$ .............................................................34
**Figure S49.** HMBC spectrum of compound **12** in $CDCl_3$ .............................................................34
**Figure S50.** NOESY spectrum of compound **12** in $CDCl_3$.............................................................35
**Figure S51.** HRESIMS spectrum of compound **13** .......................................................................35
**Figure S52.** IR spectrum of compound **13** .....................................................................................36
**Figure S53a.** $^1$H NMR spectrum of compound **13** in $CDCl_3$ ........................................................36
**Figure S54.** $^{13}$C NMR and DEPT 135 spectrum of compound **13** in $CDCl_3$.................................37
**Figure S55.** $^1$H-$^1$H COSY spectrum of compound **13** in $CDCl_3$.....................................................38
**Figure S56.** HSQC spectrum of compound **13** in $CDCl_3$ .............................................................38
**Figure S57.** HMBC spectrum of compound **13** in $CDCl_3$ .............................................................39
**Figure S58.** NOESY spectrum of compound **13** in $CDCl_3$.............................................................39
**Figure S59.** HRESIMS spectrum of compound **14** .......................................................................40
**Figure S60.** IR spectrum of compound **14** .....................................................................................40
**Figure S61a.** $^1$H NMR spectrum of compound **14** in $CDCl_3$ ........................................................41
..........................................................................................................................................41

**Figure S61b.** Partial $^1$H NMR spectrum ($\delta$ 0.6-2.7 ppm) of compound **14** in $CDCl_3$....................41
**Figure S62.** $^{13}$C NMR and DEPT 135 spectrum of compound **14** in $CDCl_3$....................42
**Figure S63.** $^1$H-$^1$H COSY spectrum of compound **14** in $CDCl_3$....................42
**Figure S64.** HSQC spectrum of compound **14** in $CDCl_3$....................43
**Figure S65.** HMBC spectrum of compound **14** in $CDCl_3$....................43
**Figure S66.** NOESY spectrum of compound **14** in $CDCl_3$....................44
**Figure S67.** Two possible conformations of compound **14**....................44
**Figure S68.** DP4+ probability analysis result for compound **14** (14a : *rel*- 5*R*, 8*R*, 9*S*, 10*R*, 13*R*, 16*S*)....................45
**Figure S69.** HRESIMS spectrum of compound **15**....................45
**Figure S70.** IR spectrum of compound **15**....................45
**Figure S71a.** $^1$H NMR spectrum of compound **15** in $CD_3OD$....................46
**Figure S71b.** Partial $^1$H NMR spectrum ($\delta$ 0.7-3.8 ppm) of compound **15** in $CD_3OD$....................46
**Figure S71c.** Partial $^1$H NMR spectrum ($\delta$ 0.7-2.3 ppm) of compound **15** in $CD_3OD$....................47
**Figure S72.** $^{13}$C NMR and DEPT 135 spectrum of compound **15** in $CD_3OD$....................47
**Figure S73.** $^1$H-$^1$H COSY spectrum of compound **15**in $CD_3OD$....................48
**Figure S74.** HSQC spectrum of compound **15** in $CD_3OD$....................48
**Figure S75.** HMBC spectrum of compound **15** in $CD_3OD$....................49
**Figure S76.** NOESY spectrum of compound **15** in $CD_3OD$....................49
**Figure S77.** HRESIMS spectrum of compound **16**....................50
**Figure S78.** IR spectrum of compound **16**....................50
**Figure S79a.** $^1$H NMR spectrum of compound **16** in $CD_3OD$....................51
**Figure S79b.** Partial $^1$H NMR spectrum ($\delta$ 0.8-4.9 ppm) of compound **16** in $CD_3OD$....................51
**Figure S79c.** Partial $^1$H NMR spectrum ($\delta$ 0.8-2.4 ppm) of compound **16** in $CD_3OD$....................52
**Figure S80.** $^{13}$C NMR and DEPT 135 spectrum of compound **16** in $CD_3OD$....................52
**Figure S81.** $^1$H-$^1$H COSY spectrum of compound **16** in $CD_3OD$....................53
**Figure S82.** HSQC spectrum of compound **16** in $CD_3OD$....................53
**Figure S83.** HMBC spectrum of compound **16** in $CD_3OD$....................54
**Figure S84.** NOESY spectrum of compound **16** in $CD_3OD$....................54
**Figure S85.** HRESIMS spectrum of compound **17**....................55
**Figure S86.** IR spectrum of compound **17**....................55
**Figure S87a.** $^1$H NMR spectrum ($\delta$ 0.7-4.3 ppm)of compound **17** in $CDCl_3$....................56
**Figure S87b.** Partial $^1$H NMR spectrum ($\delta$ 0.7-4.9 ppm)of compound **17** in $CDCl_3$....................56
**Figure S87b.** Partial $^1$H NMR spectrum ($\delta$ 0.8-2.5 ppm) of compound **17** in $CDCl_3$....................57
**Figure S88.** $^{13}$C NMR and DEPT 135 spectrum of compound **17** in $CDCl_3$....................57
**Figure S89.** $^1$H-$^1$H COSY spectrum of compound **17** in $CDCl_3$....................58
**Figure S90.** HSQC spectrum of compound **17** in $CDCl_3$....................58

**Figure S91.** HMBC spectrum of compound **17** in $CDCl_3$ ........ 59
**Figure S92.** NOESY spectrum of compound **17** in $CDCl_3$ ........ 59
**Figure S93.** HRESIMS spectrum of compound **18** ........ 60
**Figure S94.** IR spectrum of compound **18** ........ 60
**Figure S95a.** $^1$H NMR spectrum of compound **18** in $CDCl_3$ ........ 61
**Figure S95b.** Partial $^1$H NMR spectrum ($\delta$ 0.7-5.0 ppm) of compound **18** in $CDCl_3$ ........ 61
**Figure S95c.** Partial $^1$H NMR spectrum ($\delta$ 0.8-2.4 ppm) of compound **18** in $CDCl_3$ ........ 62
**Figure S96.** $^{13}$C NMR and DEPT 135 spectrum of compound **18** in $CDCl_3$ ........ 62
**Figure S97.** $^1$H-$^1$H COSY spectrum of compound **18** in $CDCl_3$ ........ 63
**Figure S98.** HSQC spectrum of compound **18** in $CDCl_3$ ........ 63
**Figure S99.** HMBC spectrum of compound **18** in $CDCl_3$ ........ 64
**Figure S100.** NOESY spectrum of compound **18** in $CDCl_3$ ........ 64
**Figure S101.** HRESIMS spectrum of compound **19** ........ 65
**Figure S102.** IR spectrum of compound **19** ........ 65
**Figure S103a.** $^1$H NMR spectrum of compound **19** in $CDCl_3$ ........ 66
**Figure S103b.** Partial $^1$H NMR spectrum ($\delta$ 0.6-4.4 ppm) of compound **19** in $CDCl_3$ ........ 66
**Figure S103c.** Partial $^1$H NMR spectrum ($\delta$ 0.7-2.3 ppm) of compound **19** in $CDCl_3$ ........ 67
**Figure S104.** $^{13}$C NMR and DEPT 135 spectrum of compound **19** in $CDCl_3$ ........ 67
**Figure S105.** $^1$H-$^1$H COSY spectrum of compound **19** in $CDCl_3$ ........ 68
**Figure S106.** HSQC spectrum of compound **19** in $CDCl_3$ ........ 68
**Figure S107.** HMBC spectrum of compound **19** in $CDCl_3$ ........ 69
**Figure S108.** NOESY spectrum of compound **19** in $CDCl_3$ ........ 69
**Figure S109.** HRESIMS spectrum of compound **20** ........ 70
**Figure S110.** IR spectrum of compound **20** ........ 70
**Figure S111a.** $^1$H NMR spectrum of compound **20** in $CDCl_3$ ........ 71
**Figure S111b.** Partial $^1$H NMR spectrum ($\delta$ 0.6-4.3 ppm) of compound **20** in $CDCl_3$ ........ 71
**Figure S111c.** Partial $^1$H NMR spectrum ($\delta$ 0.7-2.3 ppm) of compound **20** in $CDCl_3$ ........ 72
**Figure S112** $^{13}$C NMR and DEPT 135 spectrum of compound **20** in $CDCl_3$ ........ 72
**Figure S113.** $^1$H-$^1$H COSY spectrum of compound **20** in $CDCl_3$ ........ 73
**Figure S114.** HSQC spectrum of compound **20** in $CDCl_3$ ........ 73
**Figure S115.** HMBC spectrum of compound **20** in $CDCl_3$ ........ 74
**Figure S116.** NOESY spectrum of compound **20** in $CDCl_3$ ........ 74
**Figure S117.** HRESIMS spectrum of compound **21** ........ 75
**Figure S118.** IR spectrum of compound **21** ........ 75
**Figure S119a.** $^1$H NMR spectrum of compound **21** in $CDCl_3$ ........ 76
**Figure S119b.** Partial $^1$H NMR spectrum ($\delta$ 0.8-4.3 ppm) of compound **21** in $CDCl_3$ ........ 76
**Figure S119c.** Partial $^1$H NMR spectrum ($\delta$ 1.0-2.5 ppm) of compound **21** in $CDCl_3$ ........ 77

**Figure S120** $^{13}C$ NMR and DEPT 135 spectrum of compound **21** in $CDCl_3$ .......... 77
**Figure S121.** $^{1}H$-$^{1}H$ COSY spectrum of compound **21** in $CDCl_3$ .......... 78
**Figure S122.** HSQC spectrum of compound **21** in $CDCl_3$ .......... 78
**Figure S123.** HMBC spectrum of compound **21** in $CDCl_3$ .......... 79
**Figure S124.** NOESY spectrum of compound **21** in $CDCl_3$ .......... 79
**Figure S125.** HRESIMS spectrum of compound **22** .......... 80
**Figure S126.** IR spectrum of compound **22** .......... 80
**Figure S127a.** $^{1}H$ NMR spectrum of compound **22** in $CDCl_3$ .......... 81
**Figure S127b.** Partial $^{1}H$ NMR spectrum ($\delta$ 0.9-4.3 ppm) of compound **22** in $CDCl_3$ .......... 81
**Figure S127c.** Partial $^{1}H$ NMR spectrum($\delta$ 1.0-2.5 ppm) of compound **22** in $CDCl_3$ .......... 82
**Figure S128** $^{13}C$ NMR and DEPT 135 spectrum of compound **22** in $CDCl_3$ .......... 82
**Figure S129.** $^{1}H$-$^{1}H$ COSY spectrum of compound **22** in $CDCl_3$ .......... 83
**Figure S130.** HSQC spectrum of compound **22** in $CDCl_3$ .......... 83
**Figure S131.** HMBC spectrum of compound **22** in $CDCl_3$ .......... 84
**Figure S132.** NOESY spectrum of compound **22** in $CDCl_3$ .......... 84
**Figure S133.** HRESIMS spectrum of compound **23** .......... 85
**Figure S134.** IR spectrum of compound **23** .......... 85
**Figure S135a.** $^{1}H$ NMR spectrum of compound **23** in $CDCl_3$ .......... 86
**Figure S135b.** Partial $^{1}H$ NMR spectrum ($\delta$ 0.8-4.3 ppm) of compound **23** in $CDCl_3$ .......... 86
**Figure S135c.** Partial $^{1}H$ NMR spectrum ($\delta$ 0.9-2.6 ppm) of compound **23** in $CDCl_3$ .......... 87
**Figure S136.** $^{13}C$ NMR and DEPT 135 spectrum of compound **23** in $CDCl_3$ .......... 87
**Figure S137.** $^{1}H$-$^{1}H$ COSY spectrum of compound **23** in $CDCl_3$ .......... 88
**Figure S138.** HSQC spectrum of compound **23** in $CDCl_3$ .......... 88
**Figure S139.** HMBC spectrum of compound **23** in $CDCl_3$ .......... 89
**Figure S140.** NOESY spectrum of compound **23** in $CDCl_3$ .......... 89
**Figure S141.** HRESIMS spectrum of compound **24** .......... 90
**Figure S142.** IR spectrum of compound **24** .......... 90
**Figure S143a.** $^{1}H$ NMR spectrum of compound **24** in $CD_3OD$ .......... 91
**Figure S143b.** Partial $^{1}H$ NMR spectrum ($\delta$ 0.8-4.5 ppm) of compound **24** in $CD_3OD$ .......... 91
**Figure S143c.** Partial $^{1}H$ NMR spectrum ($\delta$ 1.0-2.8 ppm) of compound **24** in $CD_3OD$ .......... 92
**Figure S144.** $^{13}C$ NMR and DEPT 135 spectrum of compound **24** in $CD_3OD$ .......... 92
**Figure S145.** $^{1}H$-$^{1}H$ COSY spectrum of compound **24** in $CD_3OD$ .......... 93
**Figure S146.** HSQC spectrum of compound **24** in $CD_3OD$ .......... 93
**Figure S147.** HMBC spectrum of compound **24** in $CD_3OD$ .......... 94
**Figure S148.** NOESY spectrum of compound **24** in $CD_3OD$ .......... 94

Tolerance = 5.0 PPM / DBE: min = -1.5, max = 50.0
Element prediction: Off
Number of isotope peaks used for i-FIT = 3

Monoisotopic Mass, Even Electron Ions
151 formula(e) evaluated with 1 results within limits (up to 50 closest results for each mass)
Elements Used:
C: 0-100 H: 0-200 O: 0-20 Na: 0-1
LX
20240422-LX-S004 988 (7.216)

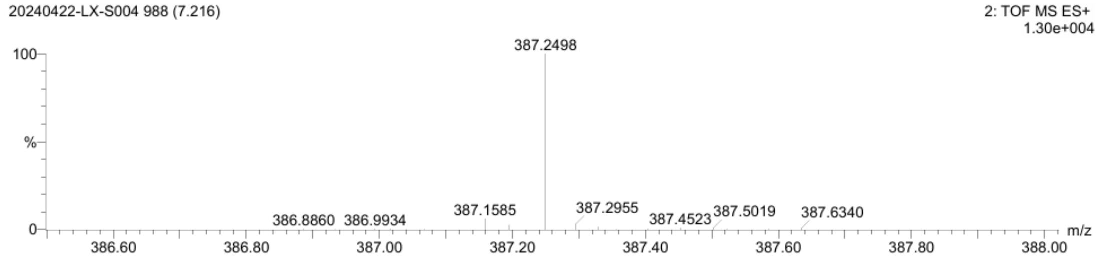


| | | | | | | | | | |
|---|---|---|---|---|---|---|---|---|---|
| Minimum: | 80.00 | | | | -1.5 | | | | |
| Maximum: | 100.00 | | 5.0 | 5.0 | 50.0 | | | | |
| Mass | RA | Calc. Mass | mDa | PPM | DBE | i-FIT | Norm | Conf(%) | Formula |
| 387.2498 | 100.00 | 387.2511 | -1.3 | -3.4 | 4.5 | 110.6 | n/a | n/a | C22 H36 O4 Na |

**Figure S1.** The HRESIMS spectrum of compound **7**.

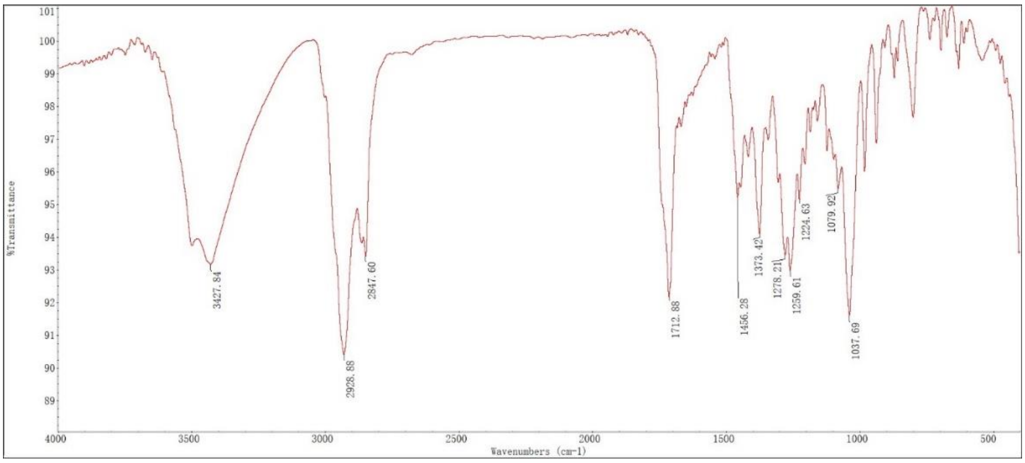


**Figure S2.** IR spectrum of compound **7**.

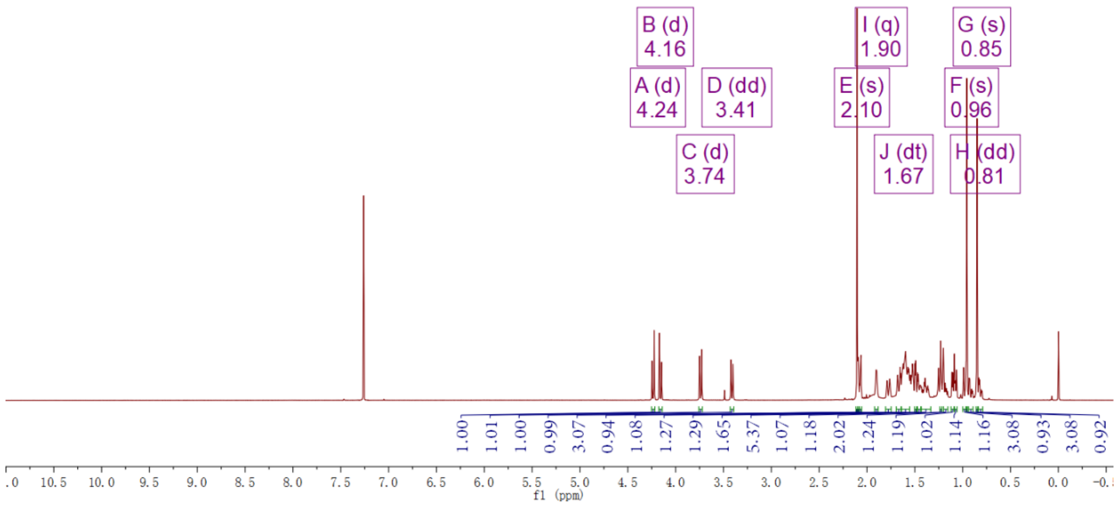


**Figure S3a.** $^{1}$H NMR spectrum of compound **7** in $CDCl_3$

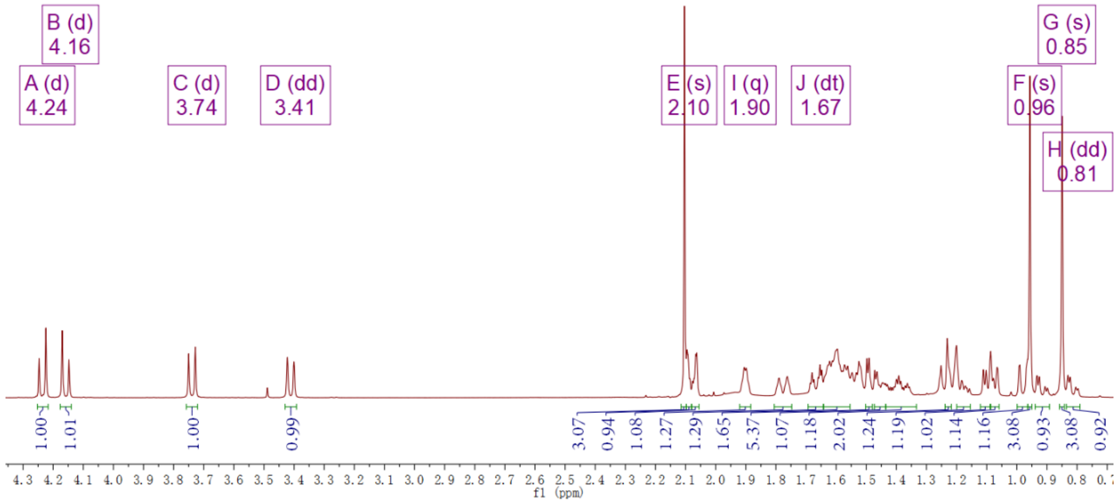


**Figure S3b.** Partial $^{1}$H NMR spectrum ($\delta$ 0.7-4.3 ppm) of compound **7** in $CDCl_3$

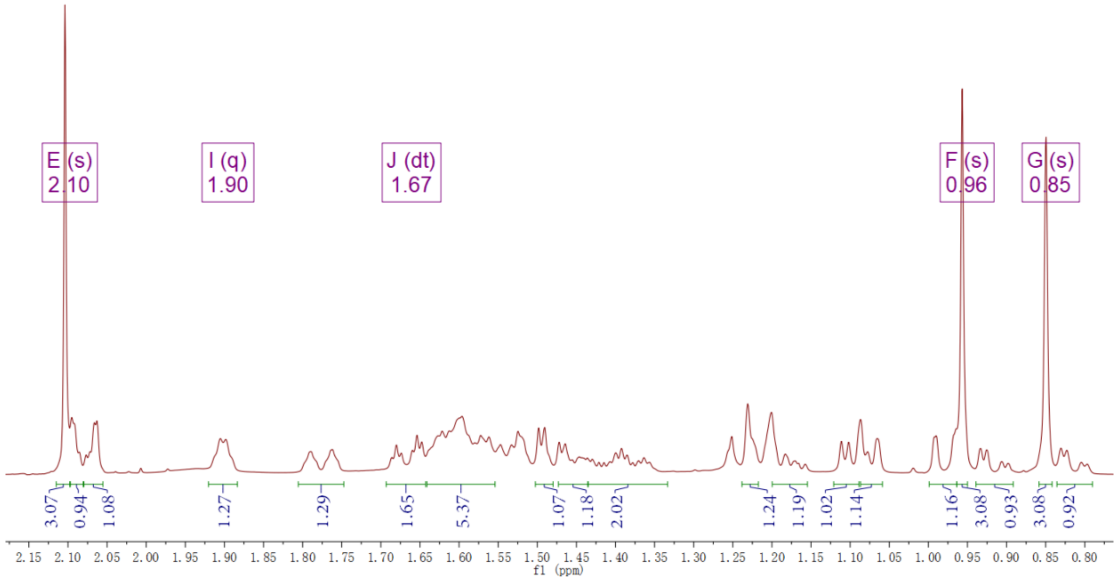


**Figure S3c.** Partial $^{1}$H NMR spectrum ($\delta$ 0.8-2.2 ppm) of compound **7** in $CDCl_3$

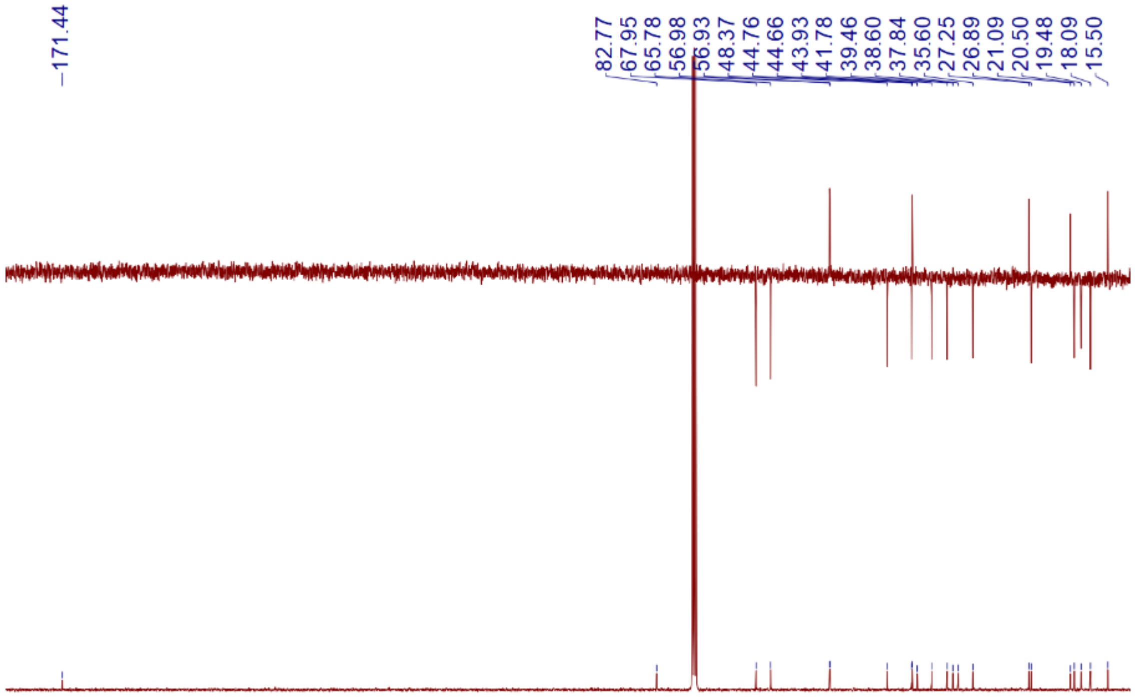


**Figure S4.** $^{13}$C NMR and DEPT 135 spectrum of compound **7** in $CDCl_3$

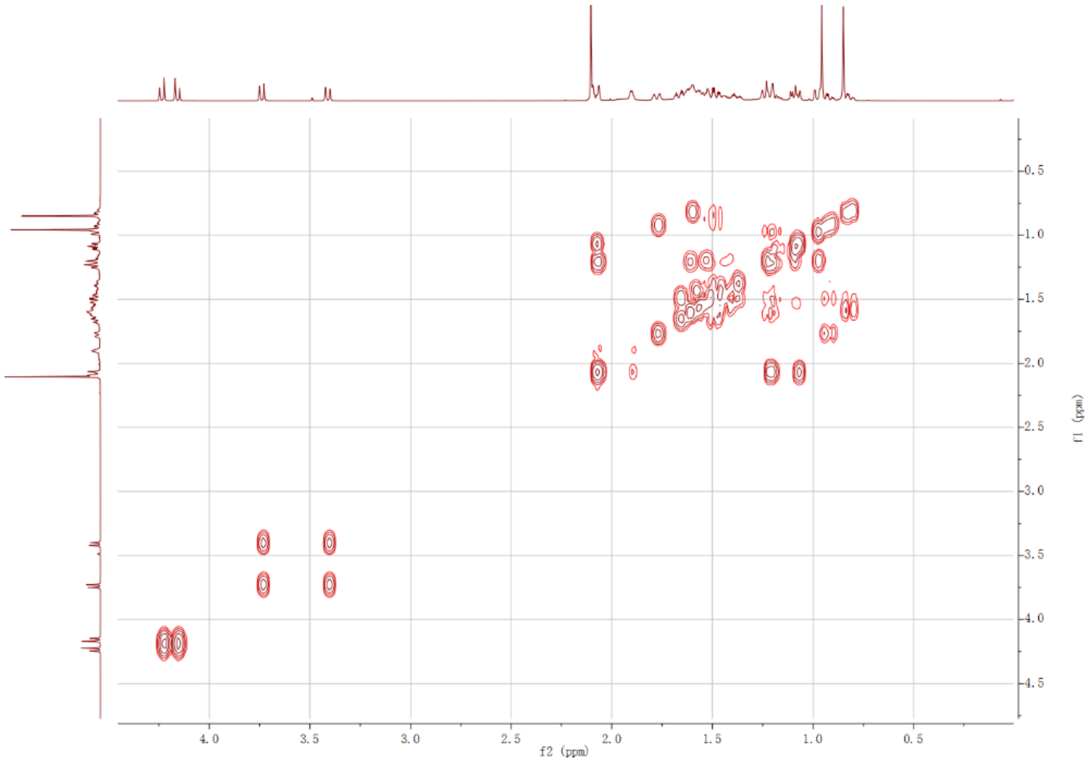


**Figure S5.** $^{1}H$-$^{1}H$ COSY spectrum of compound **7** in $CDCl_3$

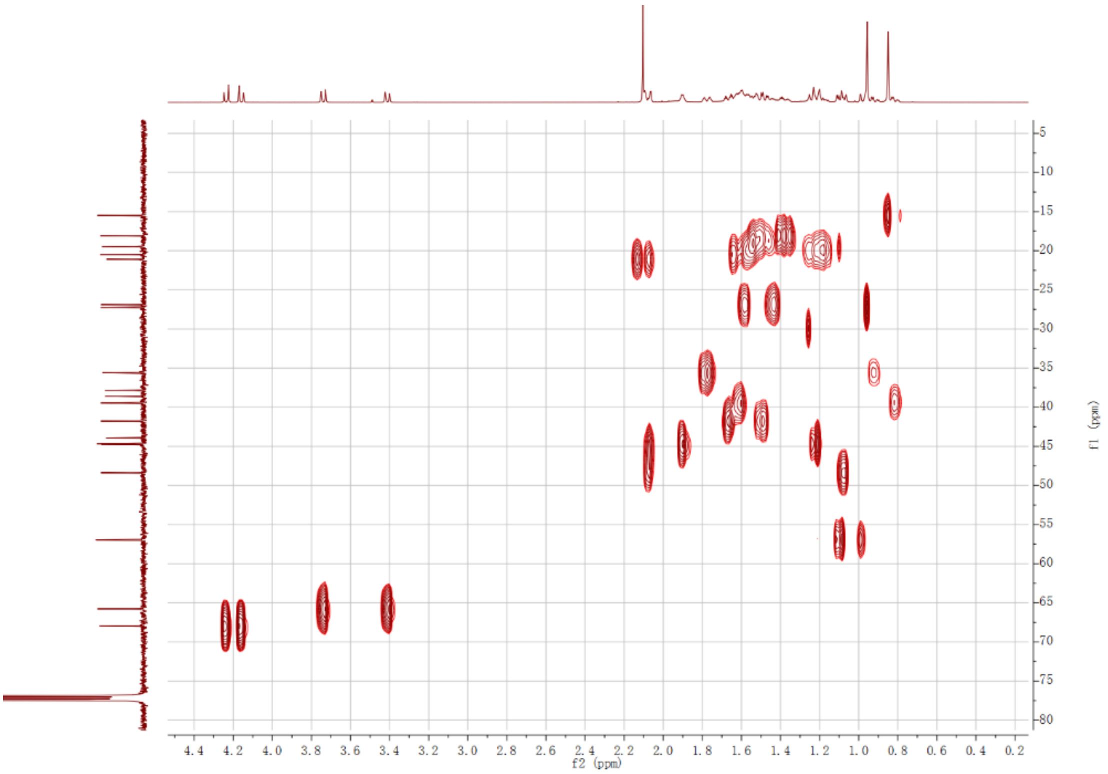


**Figure S6**. HSQC spectrum of compound **7** in $CDCl_3$

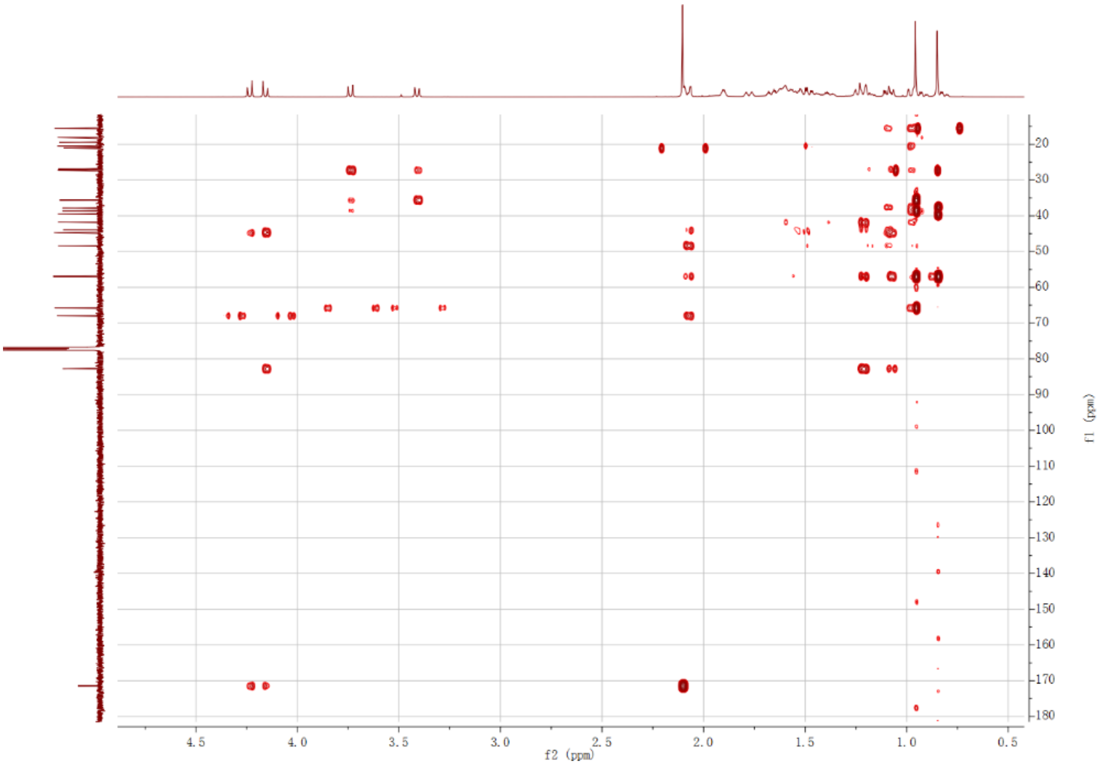


**Figure S7.** HMBC spectrum of compound **7** in $CDCl_3$

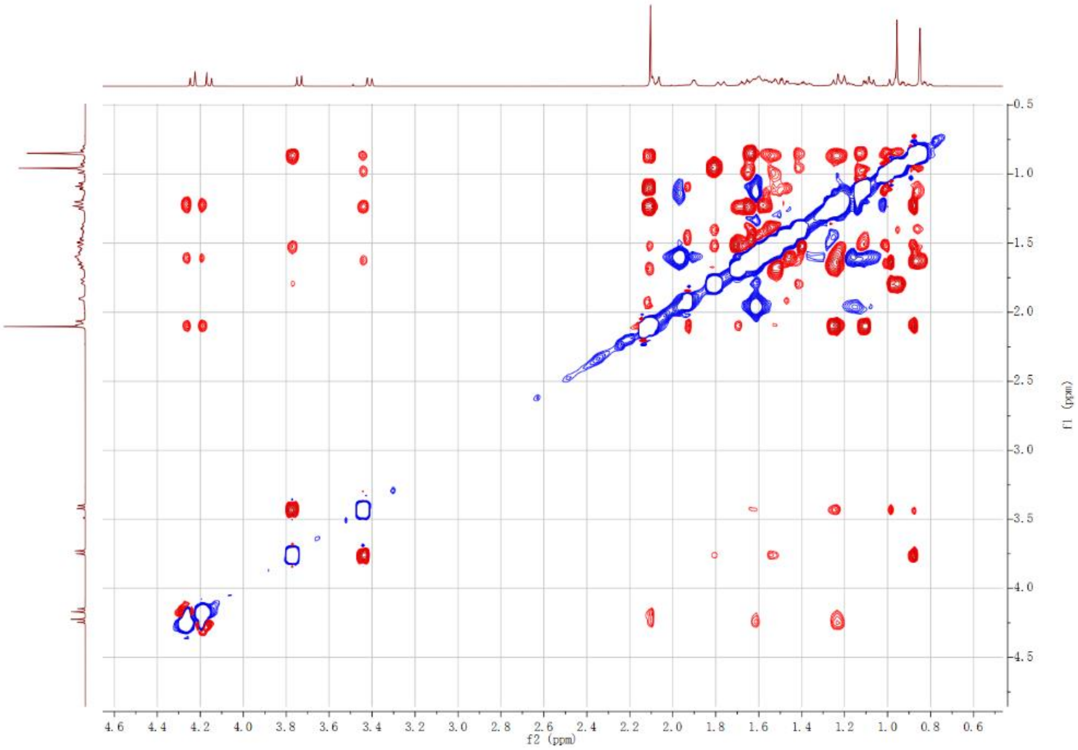


**Figure S8.** NOESY spectrum of compound **7** in $CDCl_3$

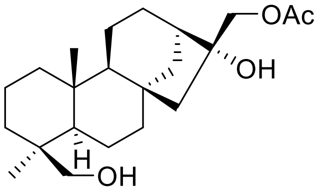


(4*S*, 5*R*, 8*S*, 9*S*, 10*R*, 13*R*, 16*R**)

7a

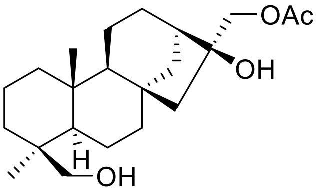


(4*S*, 5*R*, 8*S*, 9*S*, 10*R*, 13*R*, 16*S**)

7b

**Figure S9.** Two possible conformations of compound **7**

| Functional | Solvent? | Basis Set | Type of Data | | | |
|---|---|---|---|---|---|---|
| mPW1PW91 | PCM | 6-311G(d, p) | Shielding Tensors | | | |
| | Isomer 1 | Isomer 2 | Isomer 3 | Isomer 4 | Isomer 5 | Isomer 6 |
| sDP4+ (H data) | 99.98% | 0.02% | – | – | – | – |
| sDP4+ (C data) | 89.28% | 10.72% | – | – | – | – |
| sDP4+ (all data) | 100.00% | 0.00% | – | – | – | – |
| uDP4+ (H data) | 99.99% | 0.01% | – | – | – | – |
| uDP4+ (C data) | 93.30% | 6.70% | – | – | – | – |
| uDP4+ (all data) | 100.00% | 0.00% | – | – | – | – |
| DP4+ (H data) | 100.00% | 0.00% | – | – | – | – |
| DP4+ (C data) | 99.15% | 0.85% | – | – | – | – |
| DP4+ (all data) | 100.00% | 0.00% | – | – | – | – |

**Fig. S10** DP4+ probability analysis results for compound **7** (7a: *rel*- 4*S*, 5*R*, 8*S*, 9*S*, 10*R*, 13*R*, 16*R*)

Tolerance = 5.0 PPM / DBE: min = -1.5, max = 50.0
Element prediction: Off
Number of isotope peaks used for i-FIT = 3

Monoisotopic Mass, Even Electron Ions
151 formula(e) evaluated with 2 results within limits (up to 50 closest results for each mass)
Elements Used:
C: 0-100 H: 0-200 O: 0-20 Na: 0-1
LX
20240422-LX-S003 1008 (7.359)

2: TOF MS ES+
1.99e+004

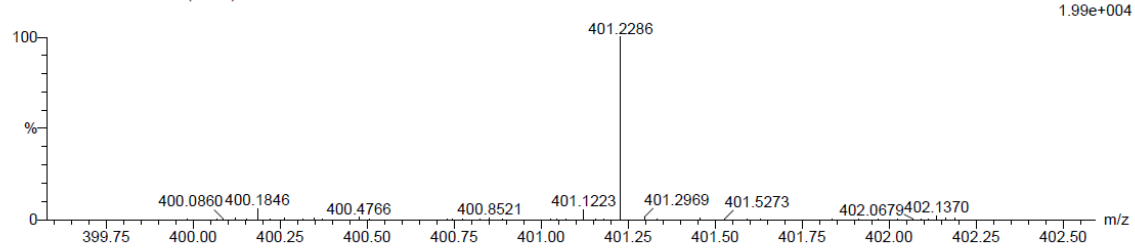


| | | | | | | | | | |
|---|---|---|---|---|---|---|---|---|---|
| Minimum: | 80.00 | | | | -1.5 | | | | |
| Maximum: | 100.00 | | 5.0 | 5.0 | 50.0 | | | | |
| Mass | RA | Calc. Mass | mDa | PPM | DBE | i-FIT | Norm | Conf(%) | Formula |
| 401.2286 | 100.00 | 401.2269 | 1.7 | 4.2 | 17.5 | 156.2 | 1.075 | 34.11 | C31 H29 |
| | | 401.2304 | -1.8 | -4.5 | 5.5 | 155.5 | 0.417 | 65.89 | C22 H34 O5 Na |

**Figure S11.** HRESIMS spectrum of compound **8**

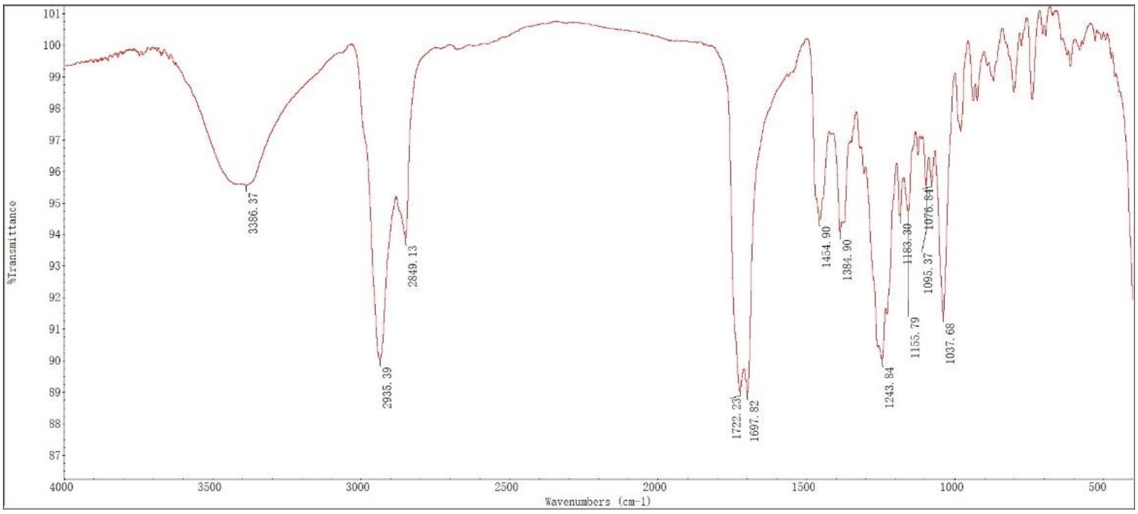


**Figure S12.** IR spectrum of compound **8**

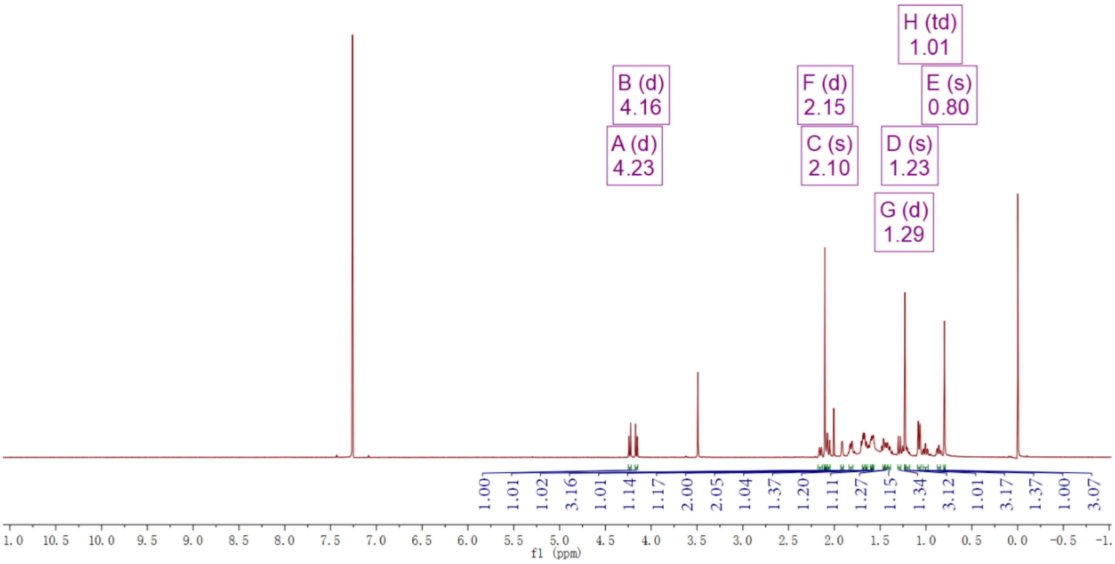


**Figure S13a.** $^1$H NMR spectrum of compound **8** in $CDCl_3$

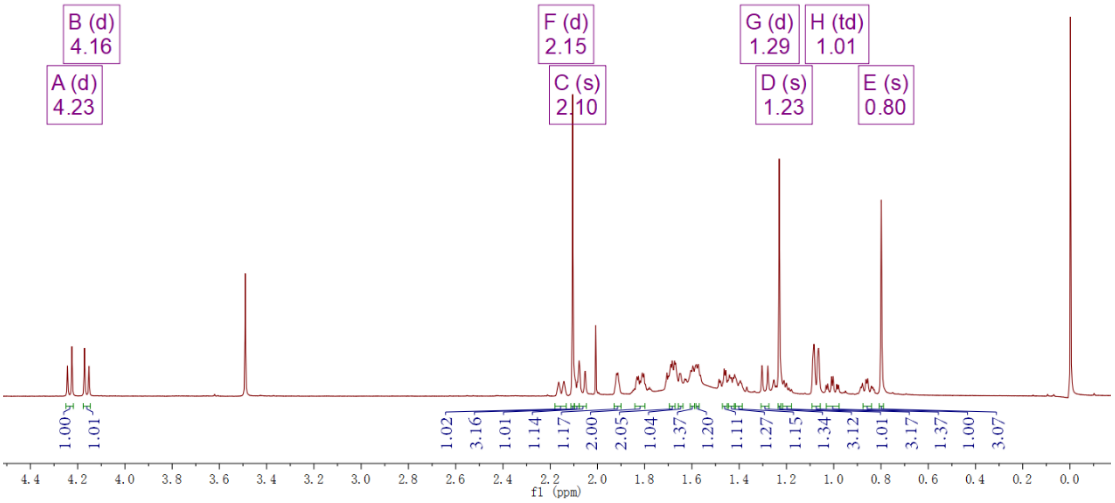


**Figure S13b.** Partial $^{1}$H NMR spectrum ($\delta$ 0.0-4.4 ppm) of compound **8** in $CDCl_3$

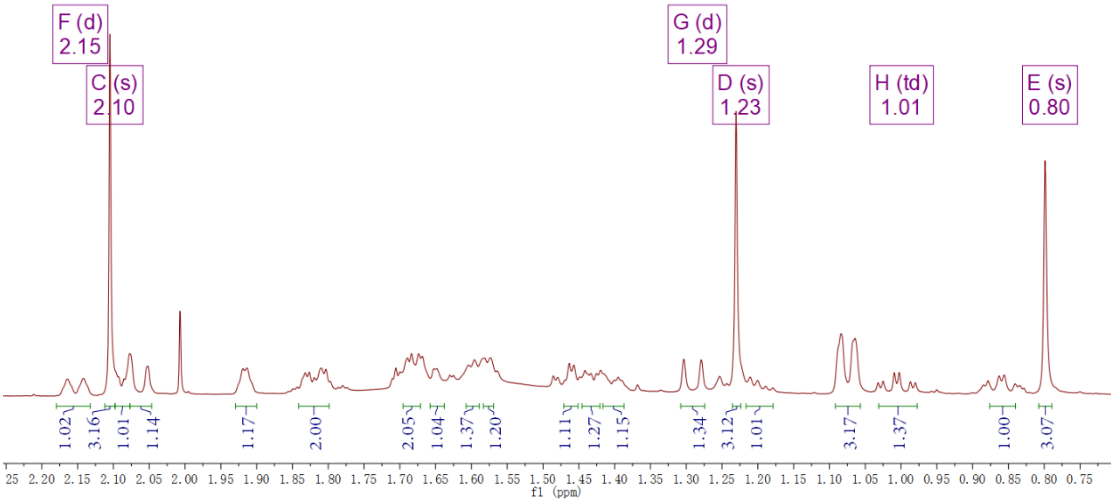


**Figure S13c.** Partial $^{1}$H NMR spectrum ($\delta$ 0.7-2.2 ppm) of compound **8** in $CDCl_3$

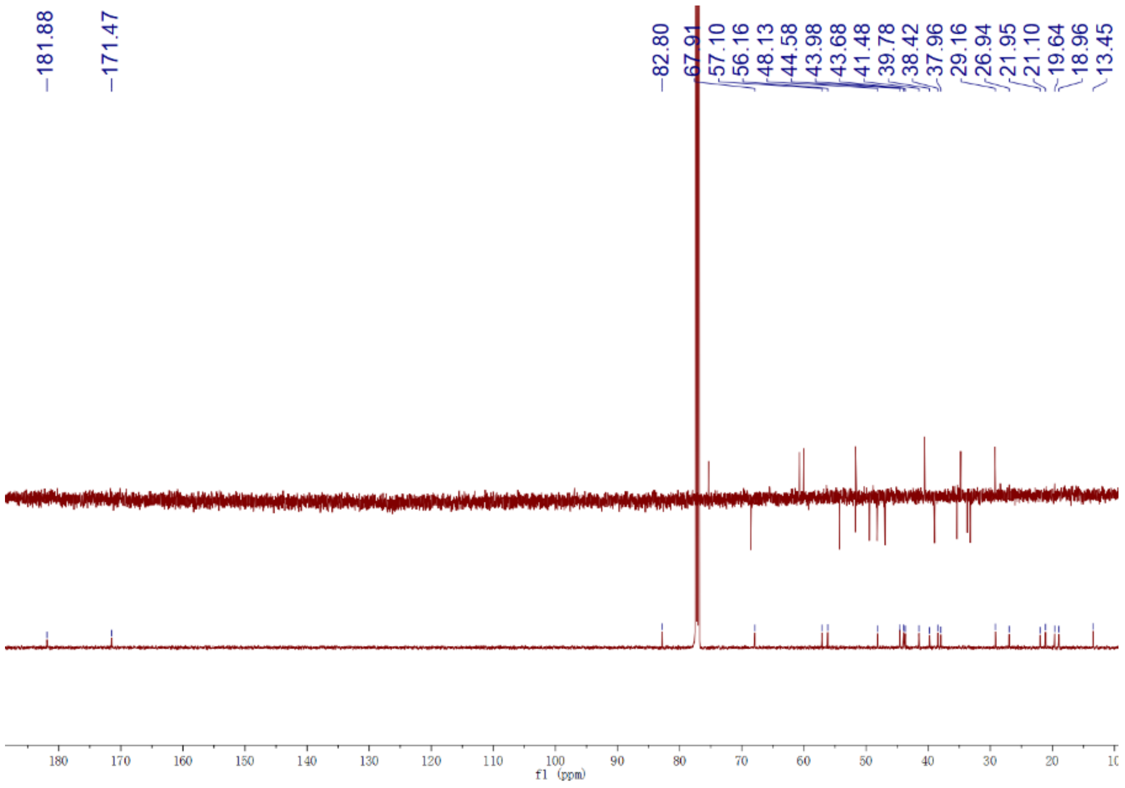


**Figure S14.** $^{13}$C NMR and DEPT 135 spectrum of compound **8** in $CDCl_3$

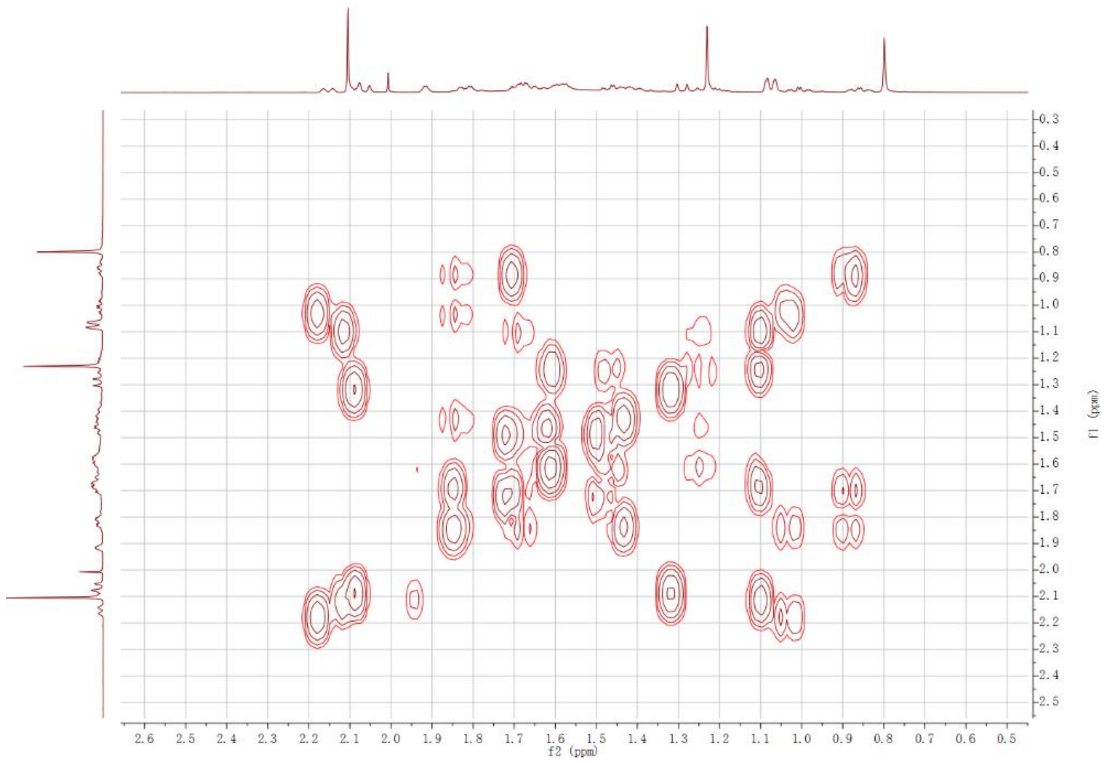


**Figure S15.** $^{1}$H-$^{1}$H COSY spectrum of compound **8** in $CDCl_3$

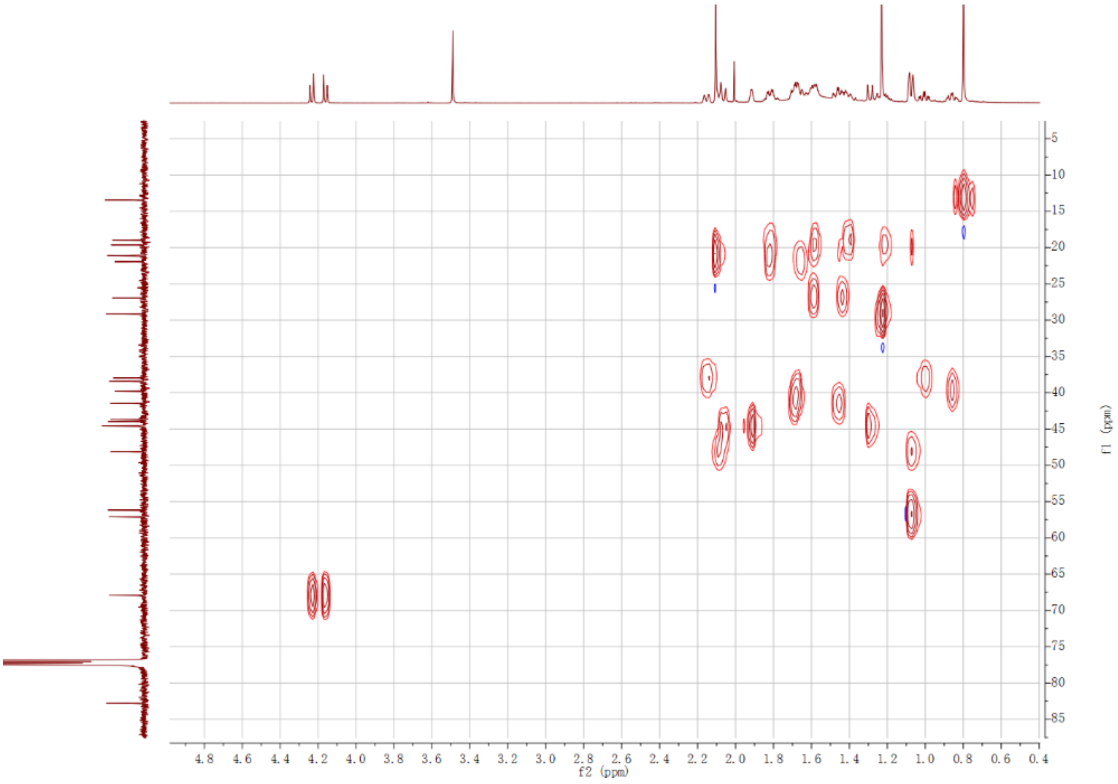


**Figure S16.** HSQC spectrum of compound **8** in $CDCl_3$

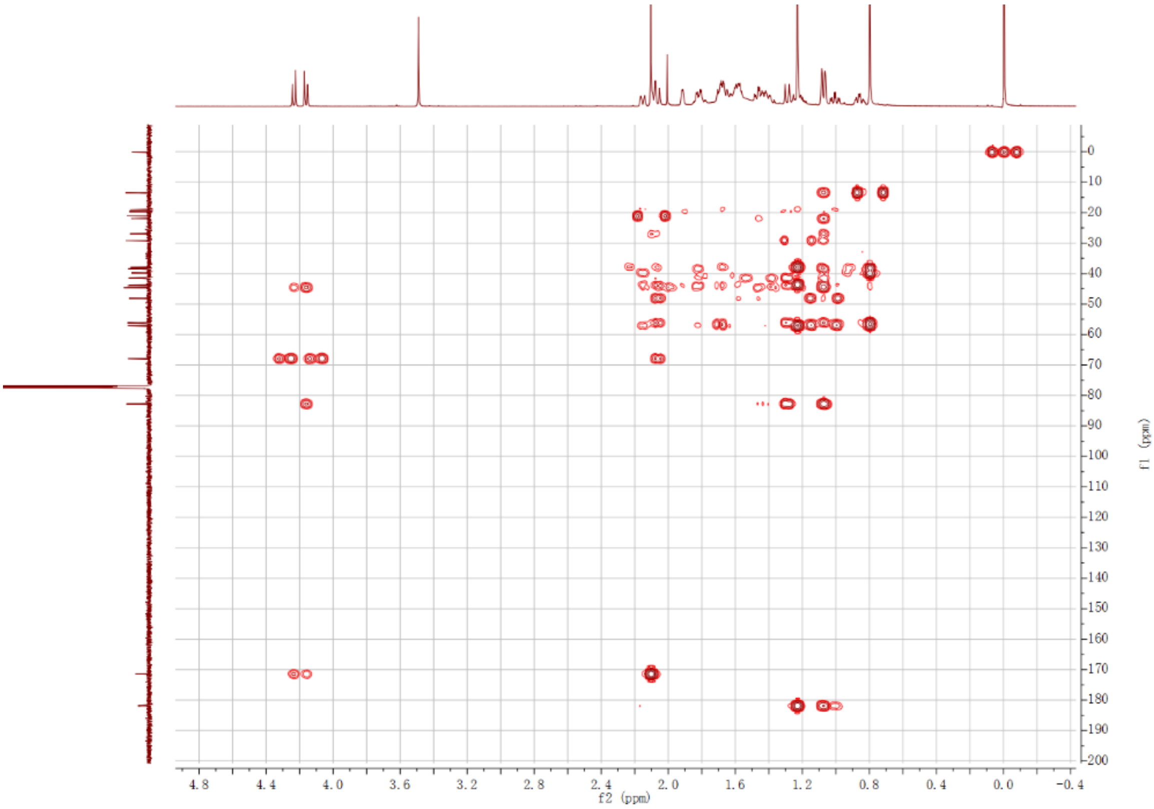


**Figure S17.** HMBC spectrum of compound **8** in $CDCl_3$

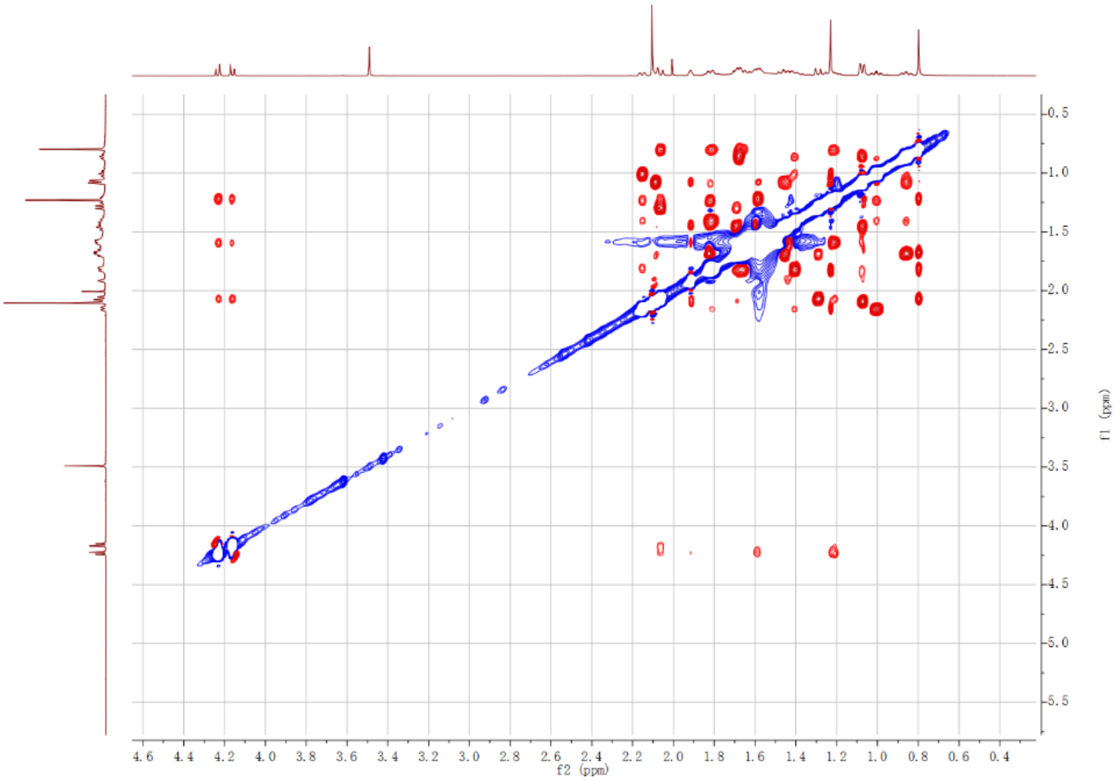


**Figure S18.** NOESY spectrum of compound **8** in $CDCl_3$

Tolerance = 5.0 PPM / DBE: min = -1.5, max = 50.0
Element prediction: Off
Number of isotope peaks used for i-FIT = 3

Monoisotopic Mass, Even Electron Ions
114 formula(e) evaluated with 1 results within limits (up to 50 closest results for each mass)
Elements Used:
C: 0-100 H: 0-200 O: 0-20 Na: 0-1
LX
20240422-LX-S002 781 (5.703)

1: TOF MS ES+
1.43e+005

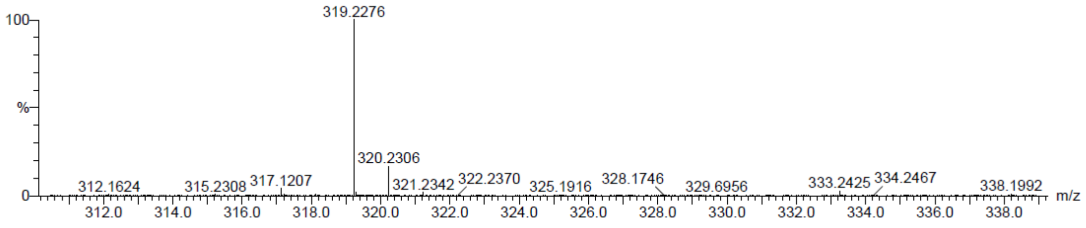


| | | | | | | | | | |
|---|---|---|---|---|---|---|---|---|---|
| Minimum: | 80.00 | | | | -1.5 | | | | |
| Maximum: | 100.00 | | 5.0 | 5.0 | 50.0 | | | | |
| Mass | RA | Calc. Mass | mDa | PPM | DBE | i-FIT | Norm | Conf(%) | Formula |
| 319.2276 | 100.00 | 319.2273 | 0.3 | 0.9 | 5.5 | 542.6 | n/a | n/a | C20 H31 O3 |

**Figure S19.** HRESIMS spectrum of compound **9**

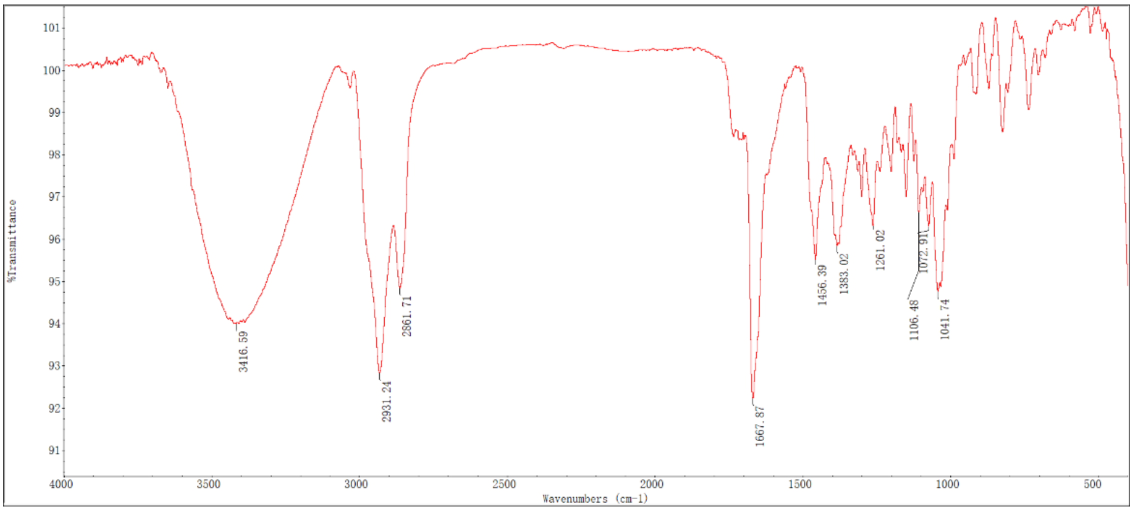


**Figure S20.** IR spectrum of compound **9**

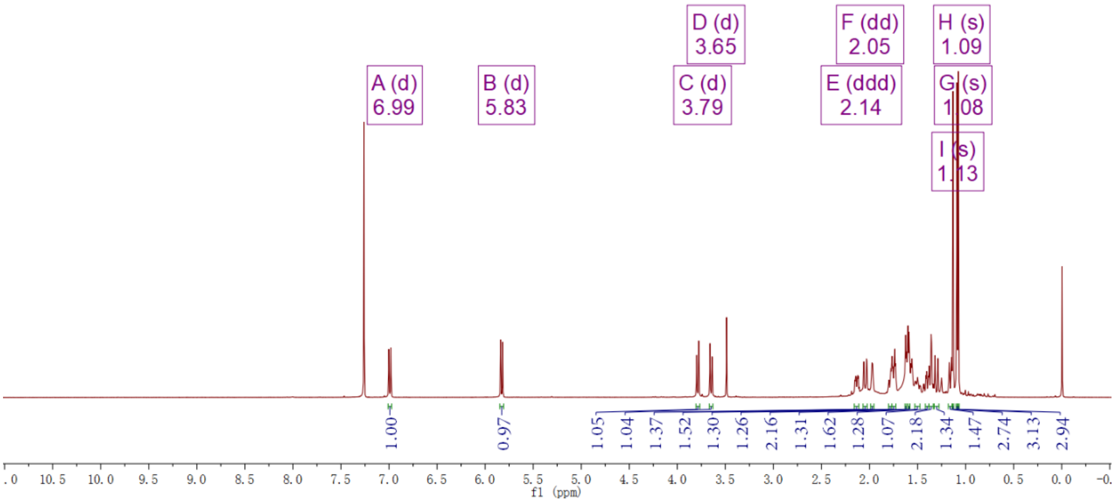


**Figure S21a.** $^1$H NMR spectrum of compound **9** in $CDCl_3$

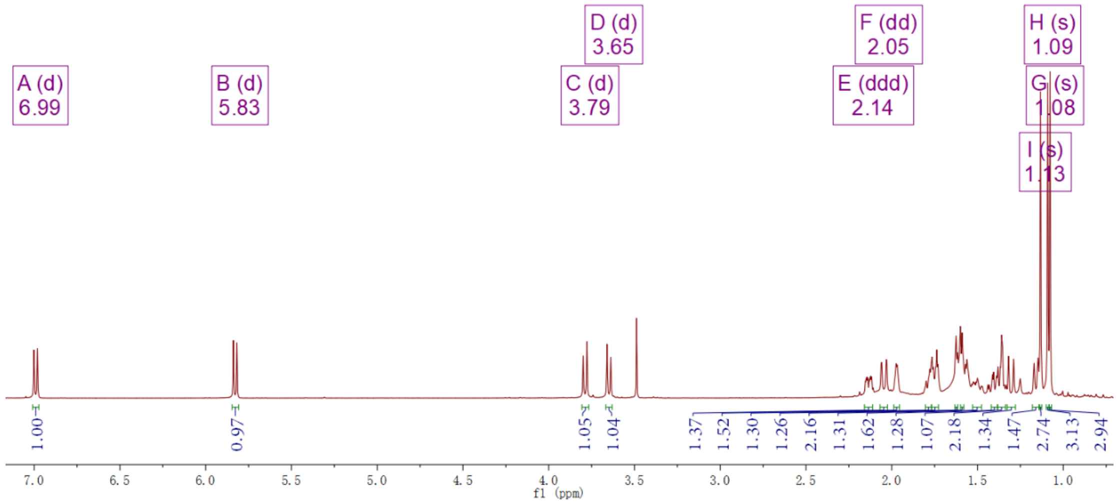


**Figure S21b.** Partial $^{1}$H NMR spectrum ($\delta$ 0.7-7.2 ppm) of compound **9** in $CDCl_3$

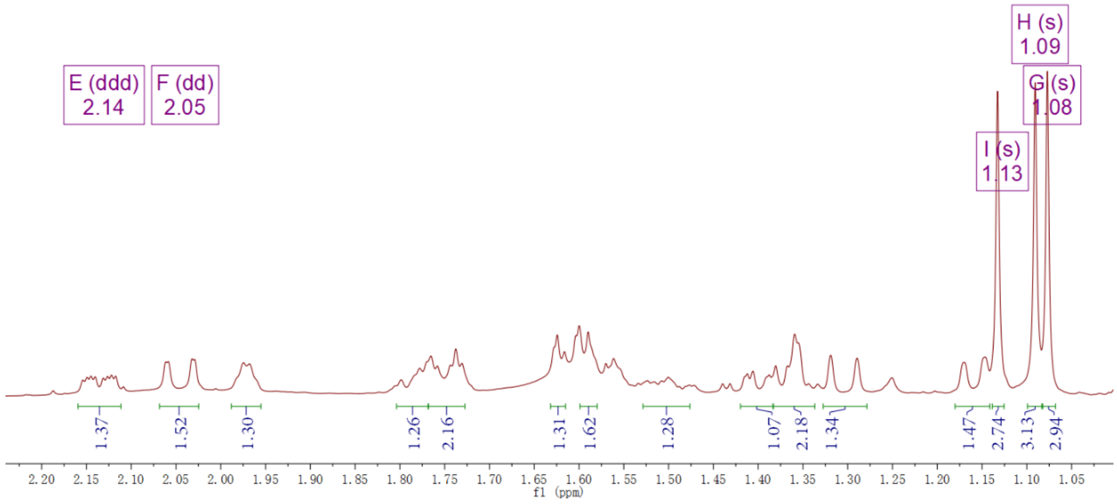


**Figure S21c.** Partial $^{1}$H NMR spectrum ($\delta$ 1.0-2.2 ppm) of compound **9** in $CDCl_3$

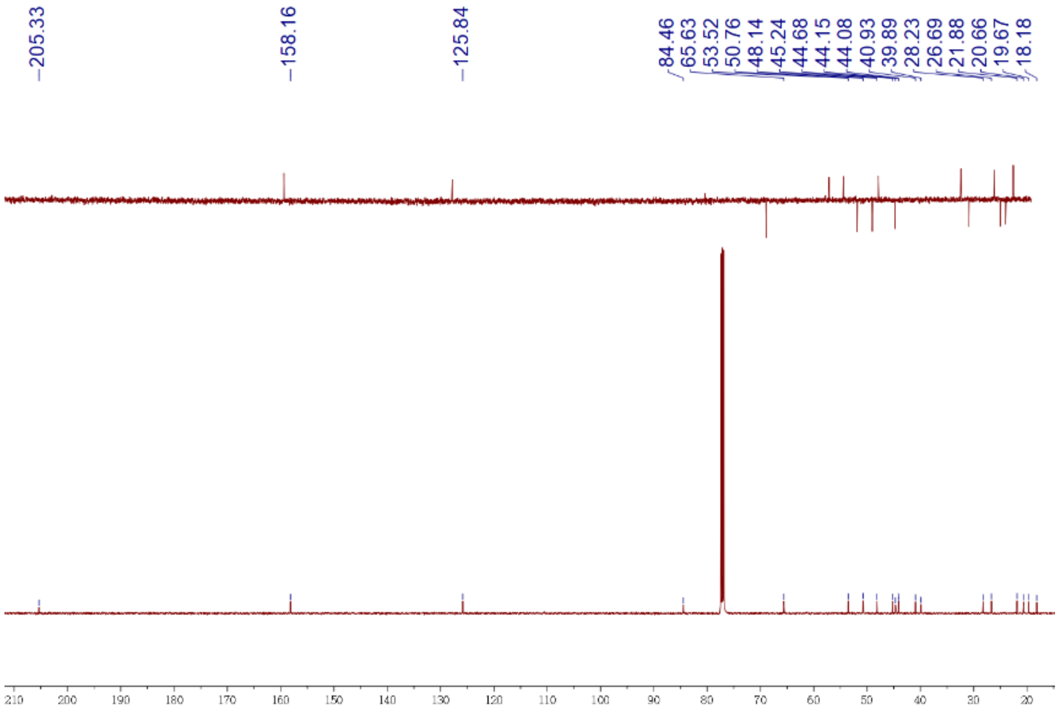


**Figure S22.** $^{13}C$ NMR and DEPT 135 spectrum of compound **9** in $CDCl_3$

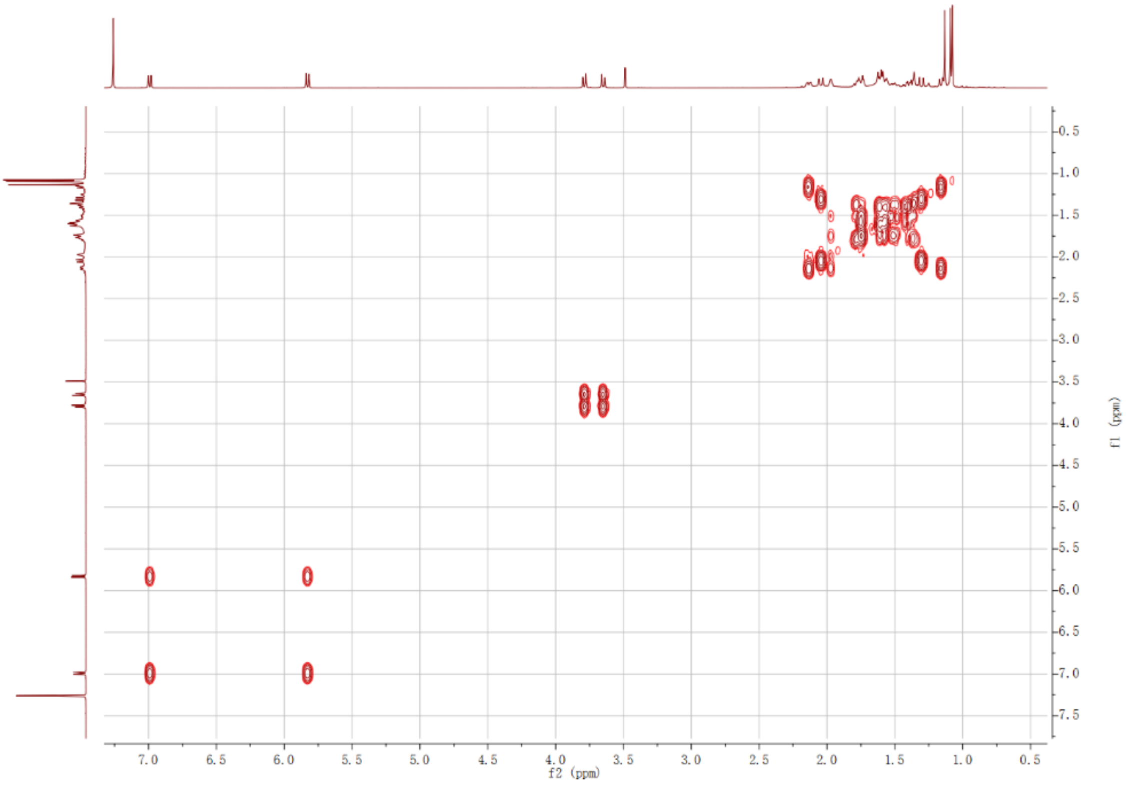


**Figure S23.** $^{1}H$-$^{1}H$ COSY spectrum of compound **9** in $CDCl_3$

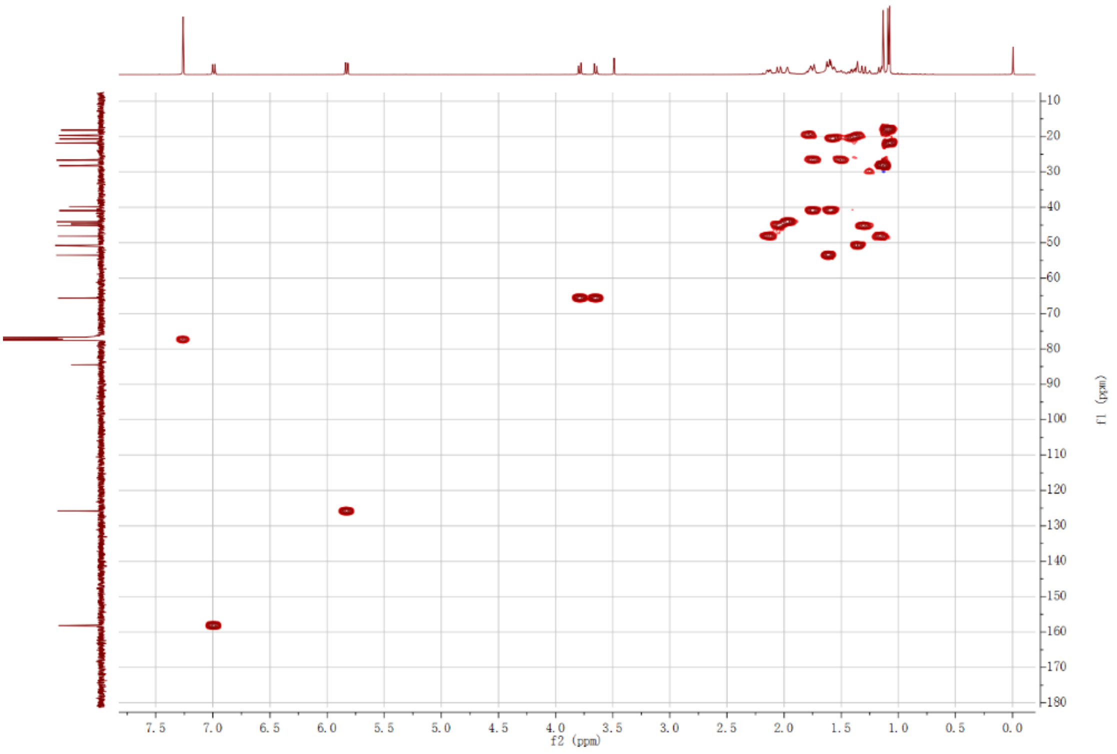


**Figure S24.** HSQC spectrum of compound **9** in $CDCl_3$

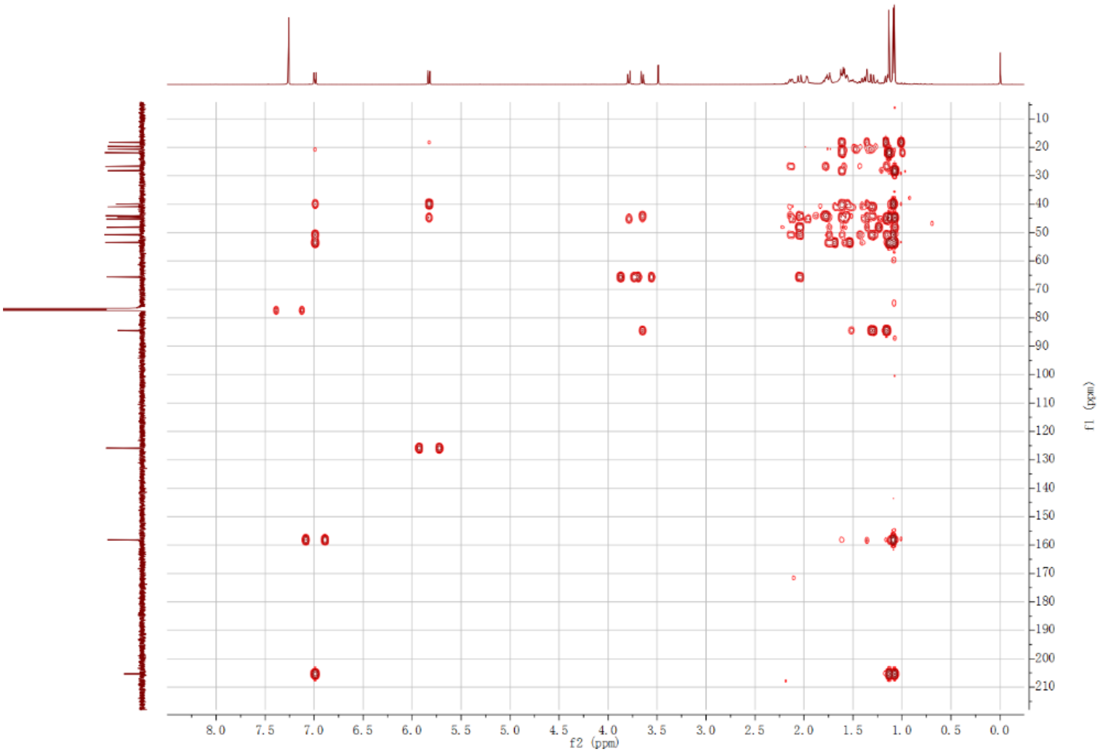


**Figure S25.** HMBC spectrum of compound **9** in $CDCl_3$

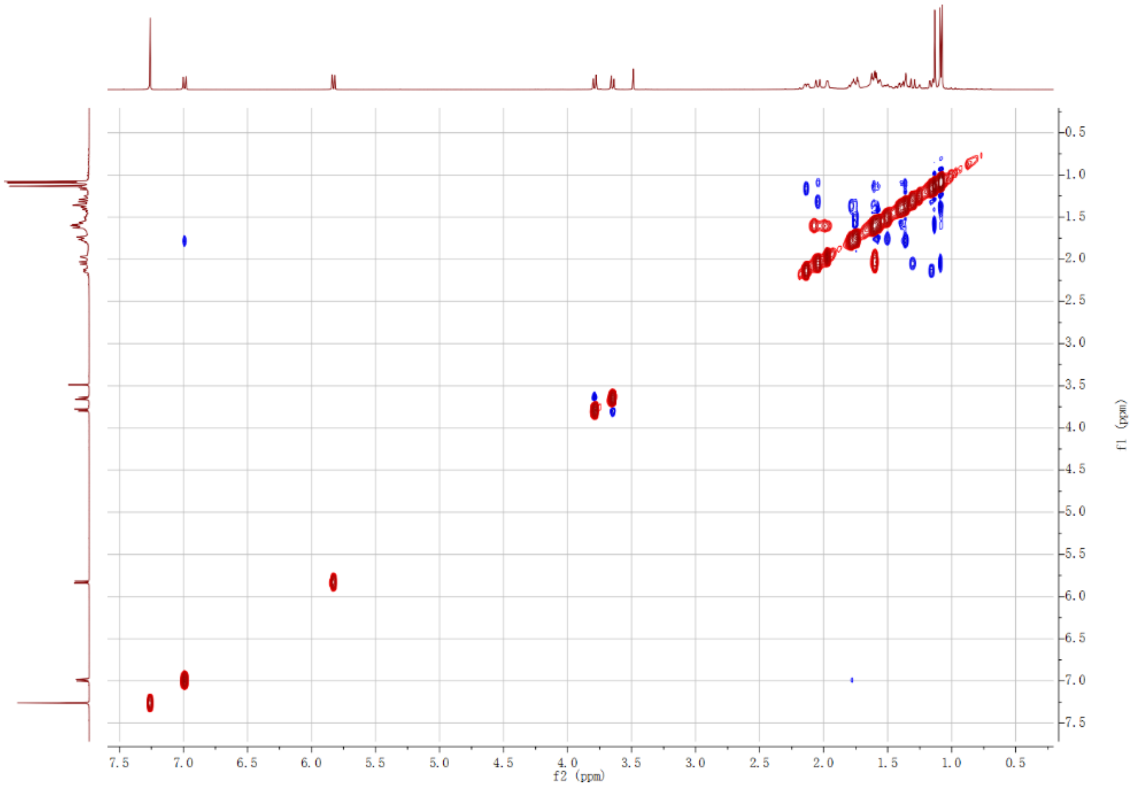


**Figure S26.** NOESY spectrum of compound **9** in $CDCl_3$

Tolerance = 5.0 PPM / DBE: min = -1.5, max = 50.0
Element prediction: Off
Number of isotope peaks used for i-FIT = 3

Monoisotopic Mass, Even Electron Ions
101 formula(e) evaluated with 1 results within limits (up to 50 closest results for each mass)
Elements Used:
C: 0-100 H: 0-200 O: 0-20 Na: 0-1
LX
20240422-LX-S013 1053 (7.685)

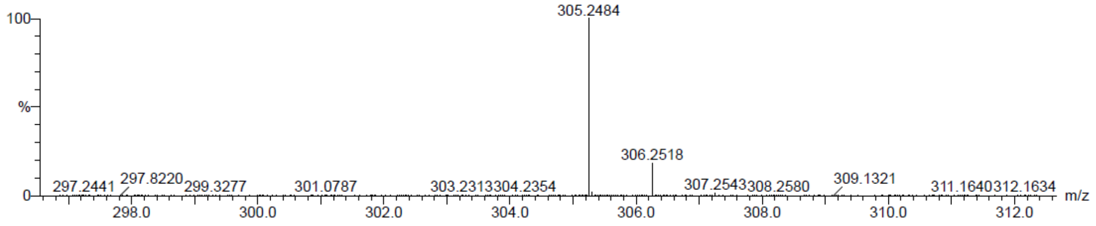


| | Mass | RA | Calc. Mass | mDa | PPM | DBE | i-FIT | Norm | Conf(%) | Formula |
|---|---|---|---|---|---|---|---|---|---|---|
| Minimum: | | 80.00 | | | | -1.5 | | | | |
| Maximum: | | 100.00 | | 5.0 | 5.0 | 50.0 | | | | |
| | 305.2484 | 100.00 | 305.2481 | 0.3 | 1.0 | 4.5 | 500.7 | n/a | n/a | C20 H33 O2 |

**Figure S27.** HRESIMS spectrum of compound **10**

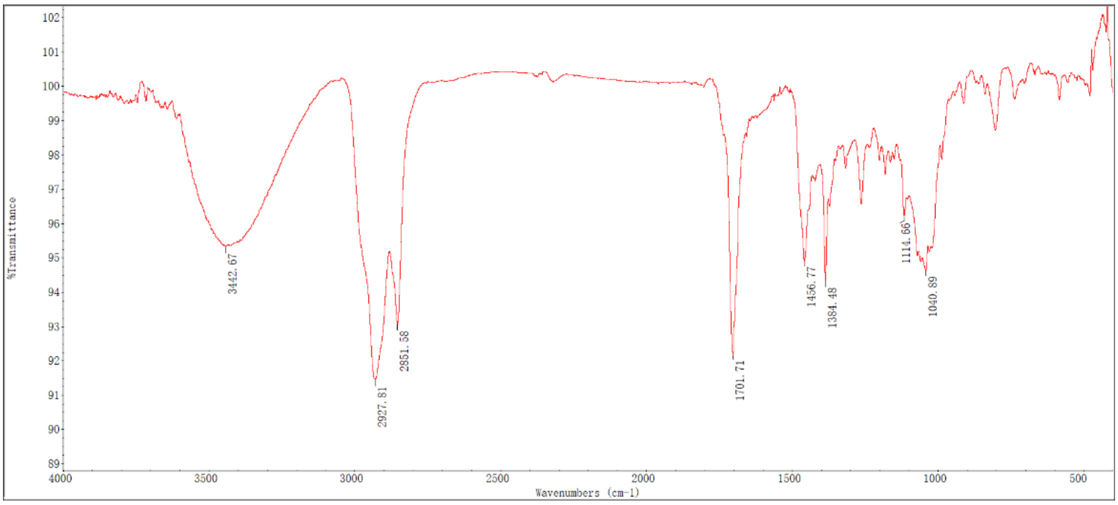


**Figure S28.** IR spectrum of compound **10**

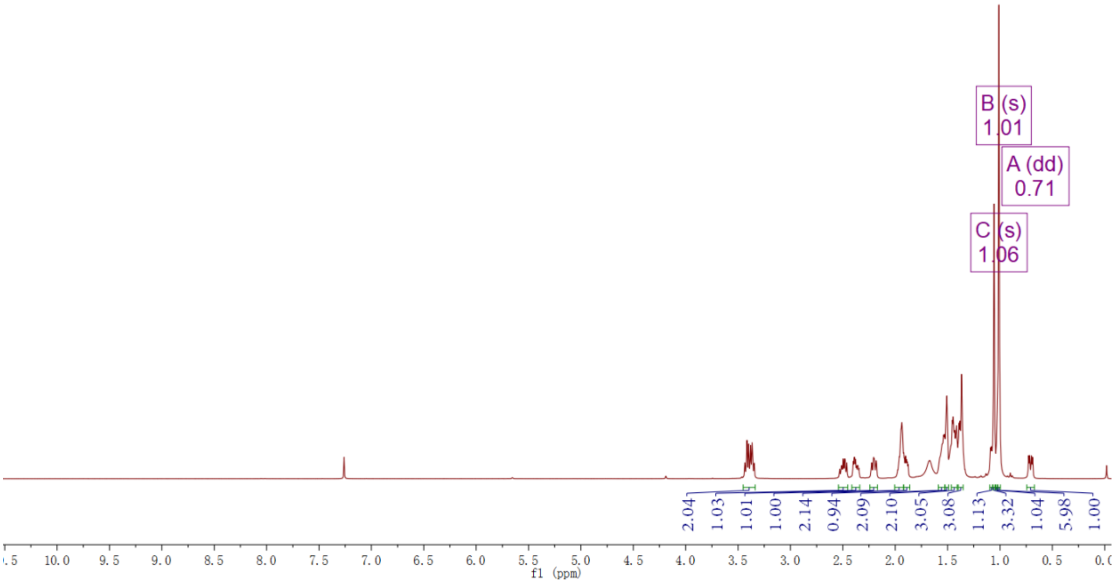


**Figure S29a.** $^{1}H$ NMR spectrum of compound **10** in $CDCl_3$

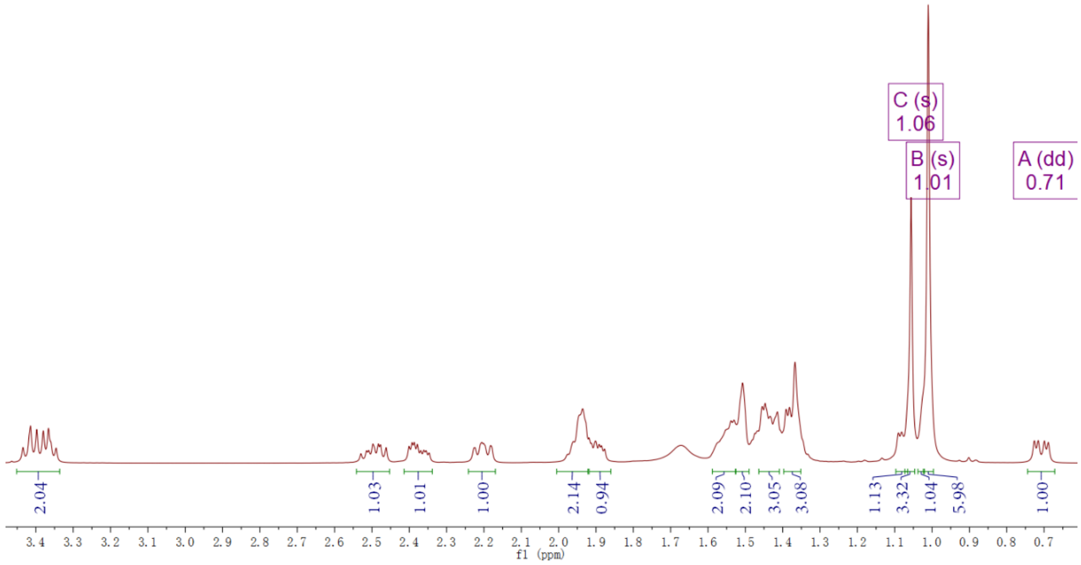


**Figure S29b.** Partial $^{1}$H NMR spectrum ($\delta$ 0.6-3.5 ppm) of compound **10** in $CDCl_3$

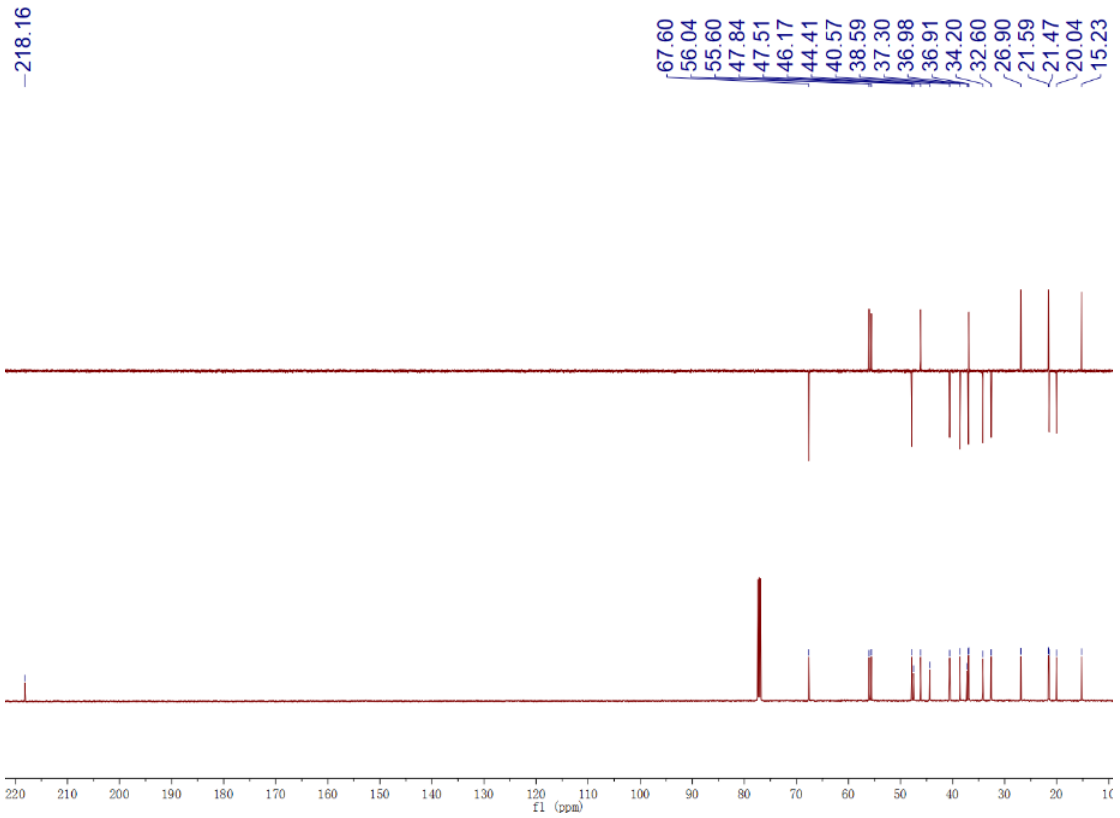


**Figure S30.** $^{13}$C NMR and DEPT 135 spectrum of compound **10** in $CDCl_3$

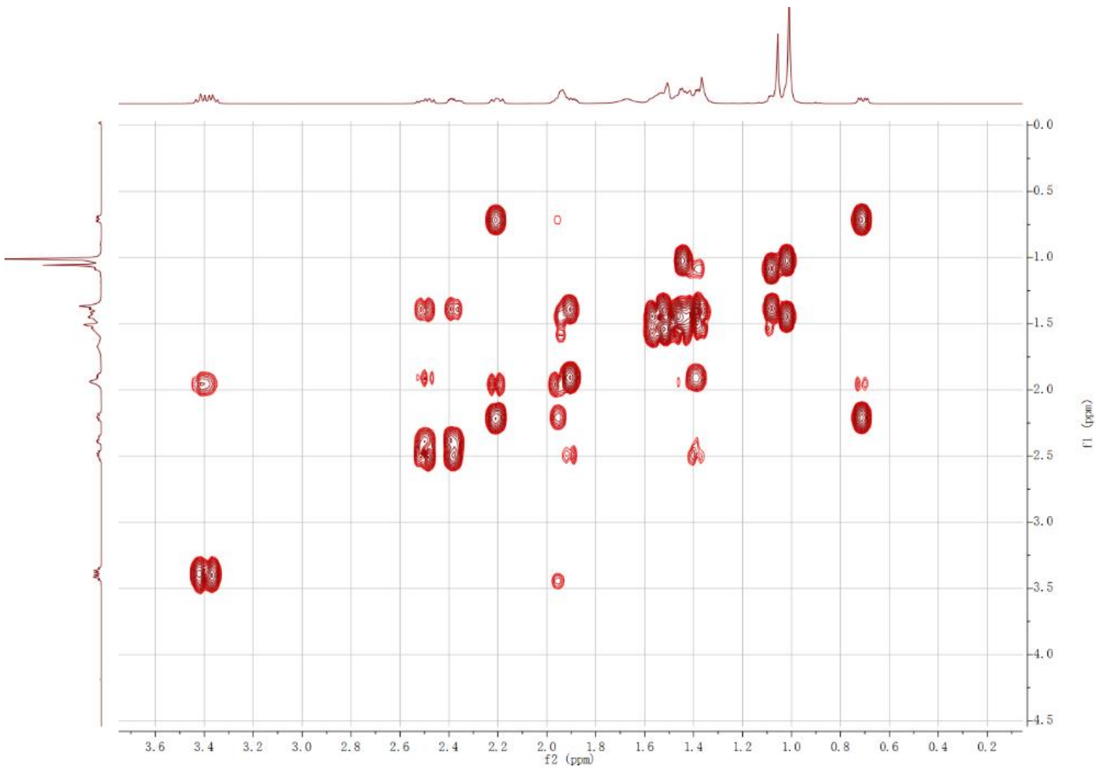


**Figure S31.** $^{1}H$-$^{1}H$ COSY spectrum of compound **10** in $CDCl_3$

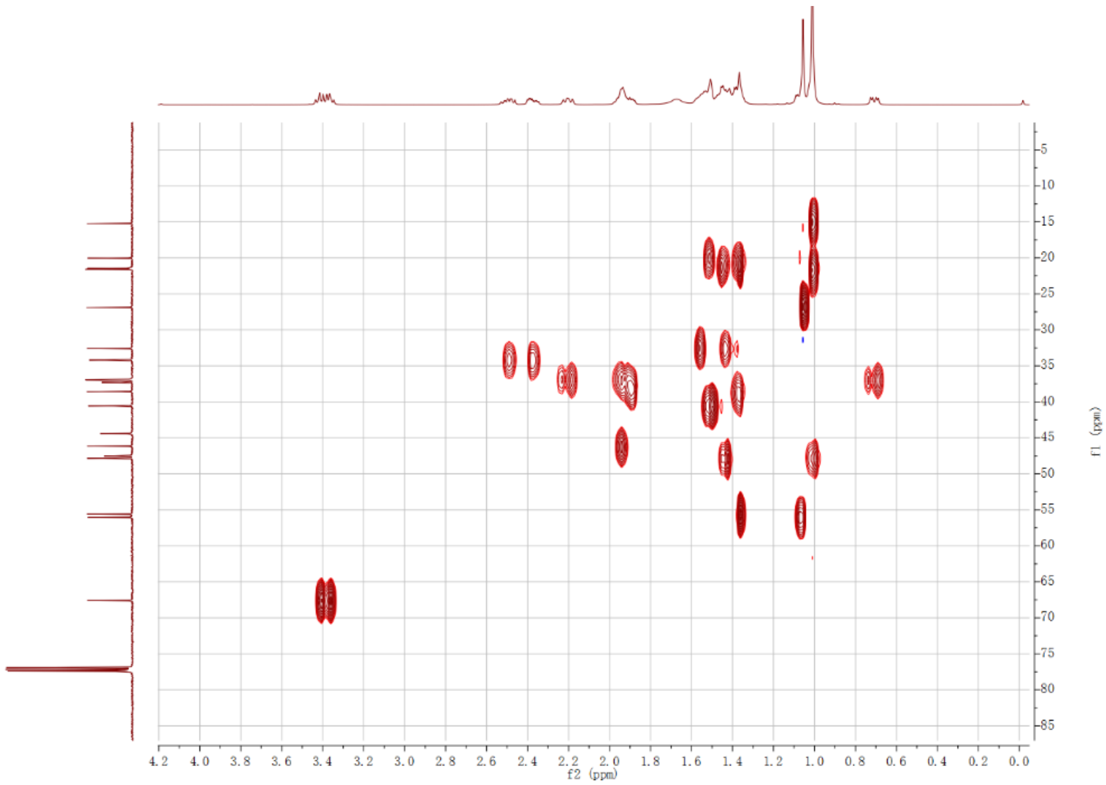


**Figure S32.** HSQC spectrum of compound **10** in $CDCl_3$

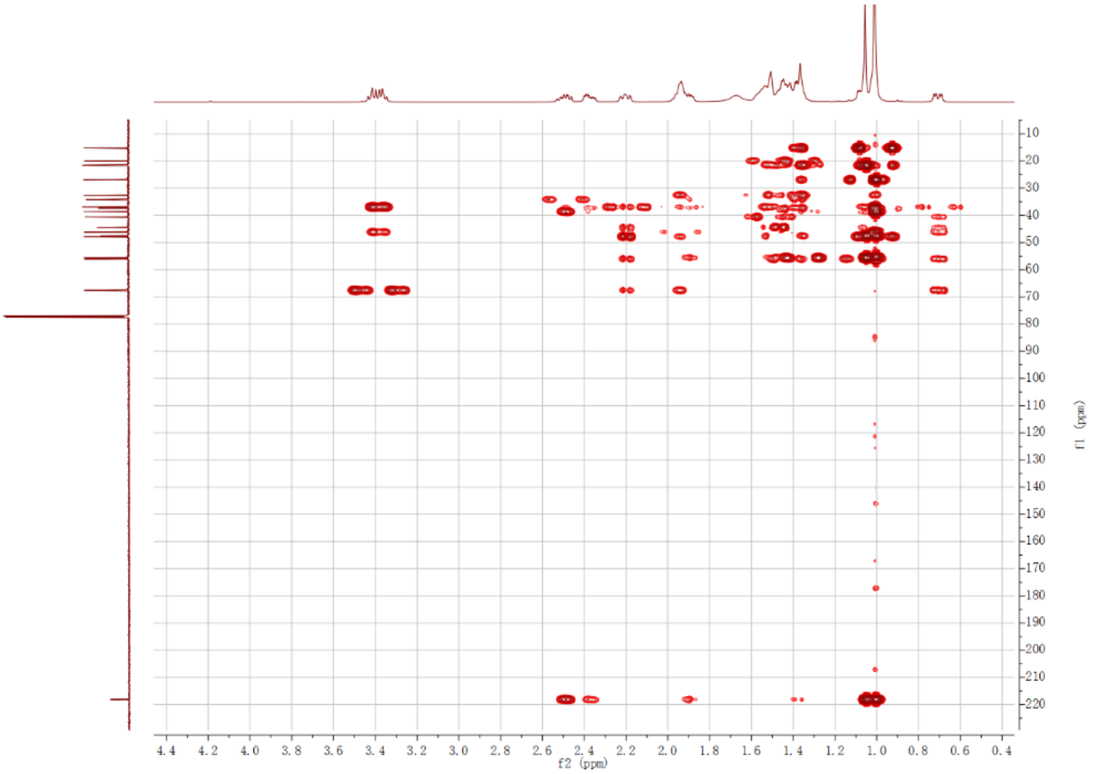


**Figure S33.** HMBC spectrum of compound **10** in $CDCl_3$

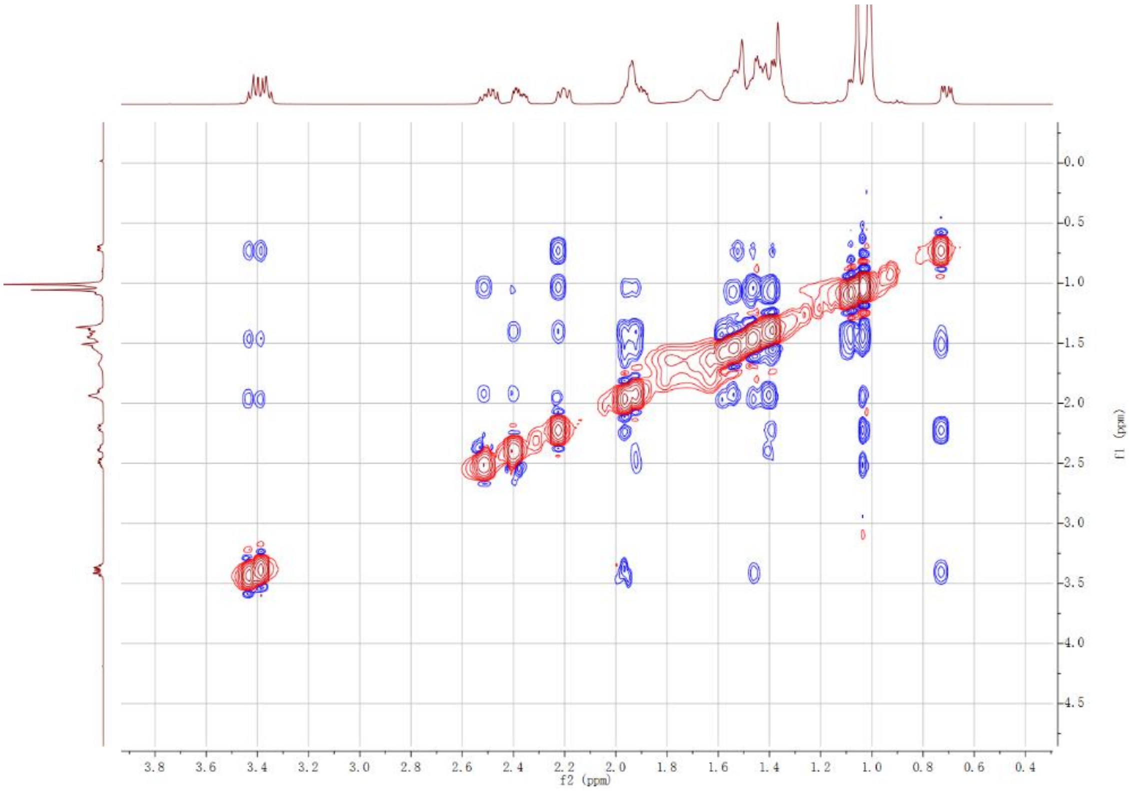


**Figure S34.** NOESY spectrum of compound **10** in $CDCl_3$

Tolerance = 5.0 PPM / DBE: min = -1.5, max = 50.0
Element prediction: Off
Number of isotope peaks used for i-FIT = 3

Monoisotopic Mass, Even Electron Ions
135 formula(e) evaluated with 1 results within limits (up to 50 closest results for each mass)
Elements Used:
C: 0-100 H: 0-200 O: 0-20 Na: 0-1
LX
20240422-LX-S015 1059 (7.738) 2: TOF MS ES+ 9.85e+003

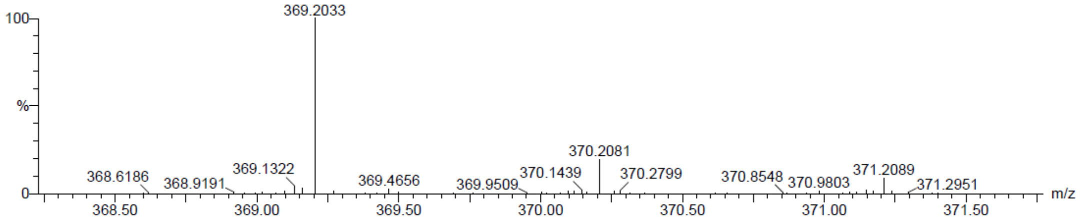


| | | | | | | | | | |
|---|---|---|---|---|---|---|---|---|---|
| Minimum: | 80.00 | | | | -1.5 | | | | |
| Maximum: | 100.00 | | 5.0 | 5.0 | 50.0 | | | | |
| Mass | RA | Calc. Mass | mDa | PPM | DBE | i-FIT | Norm | Conf(%) | Formula |
| 369.2033 | 100.00 | 369.2042 | -0.9 | -2.4 | 6.5 | 282.2 | n/a | n/a | C21 H30 O4 Na |

**Figure S35.** HRESIMS spectrum of compound **11**

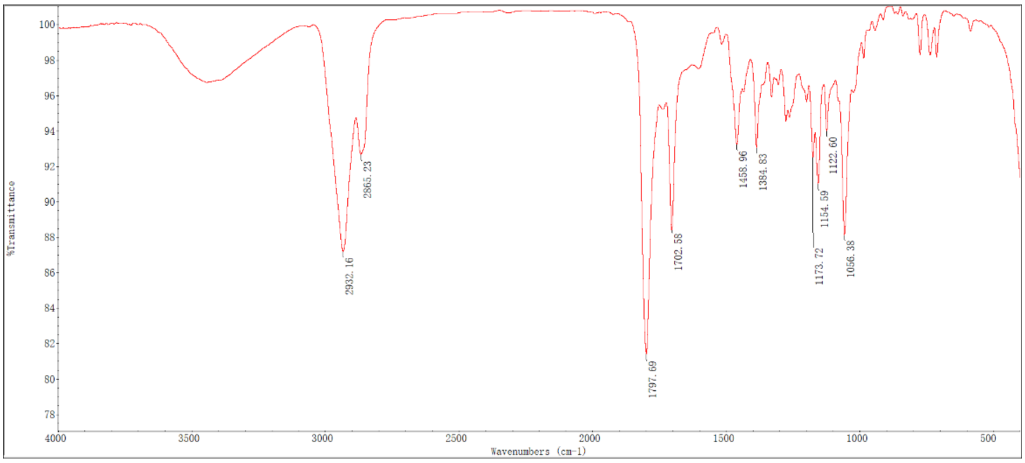


**Figure S36.** IR spectrum of compound **11**

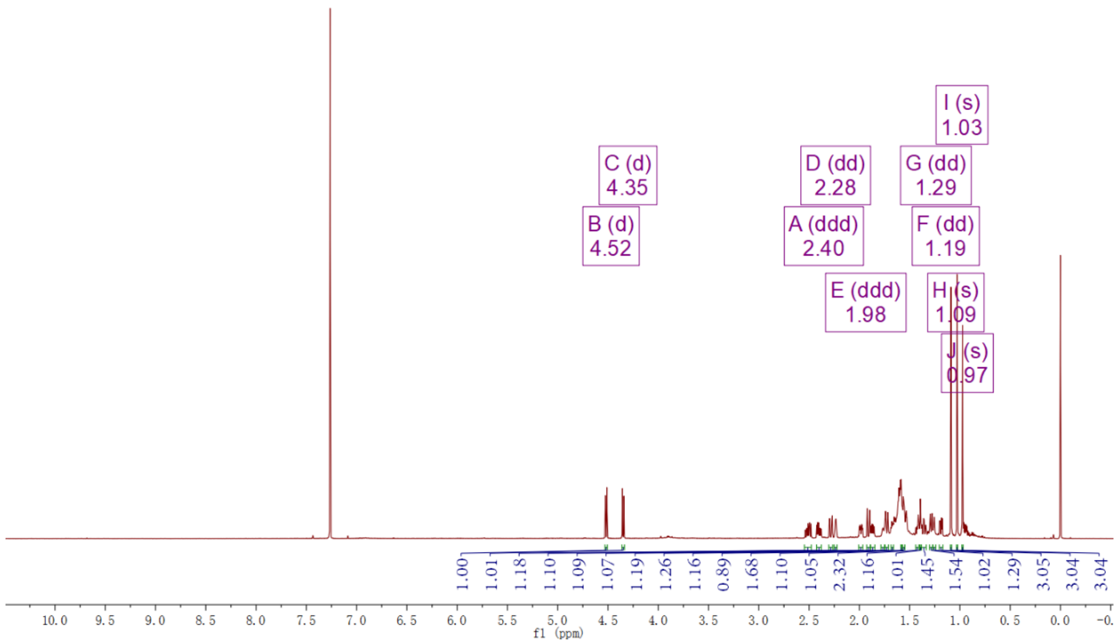


**Figure S37a.** $^1$H NMR spectrum of compound **11** in $CDCl_3$

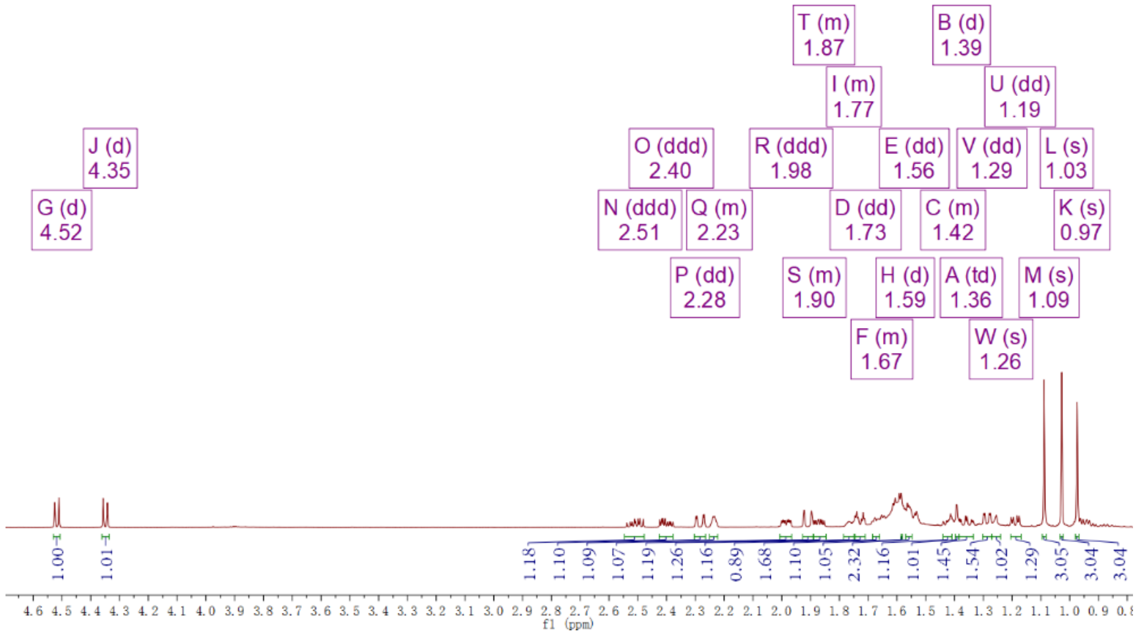


**Figure S37b.** Partial $^1$H NMR spectrum ($\delta$ 0.8-4.6 ppm) of compound **11** in $CDCl_3$

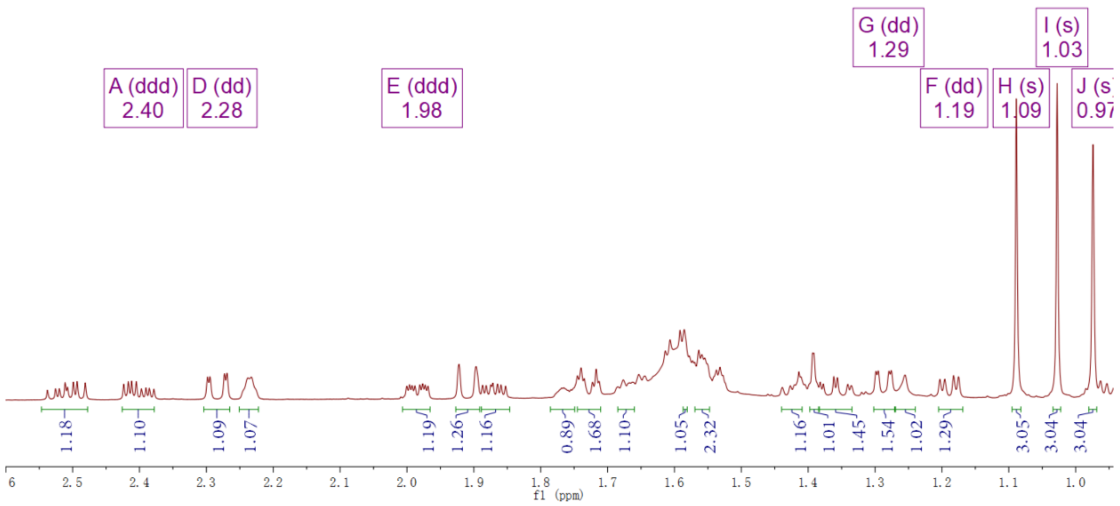


**Figure S37c.** Partial $^{1}H$ NMR spectrum ($\delta$ 0.9-2.6 ppm) of compound **11** in $CDCl_3$

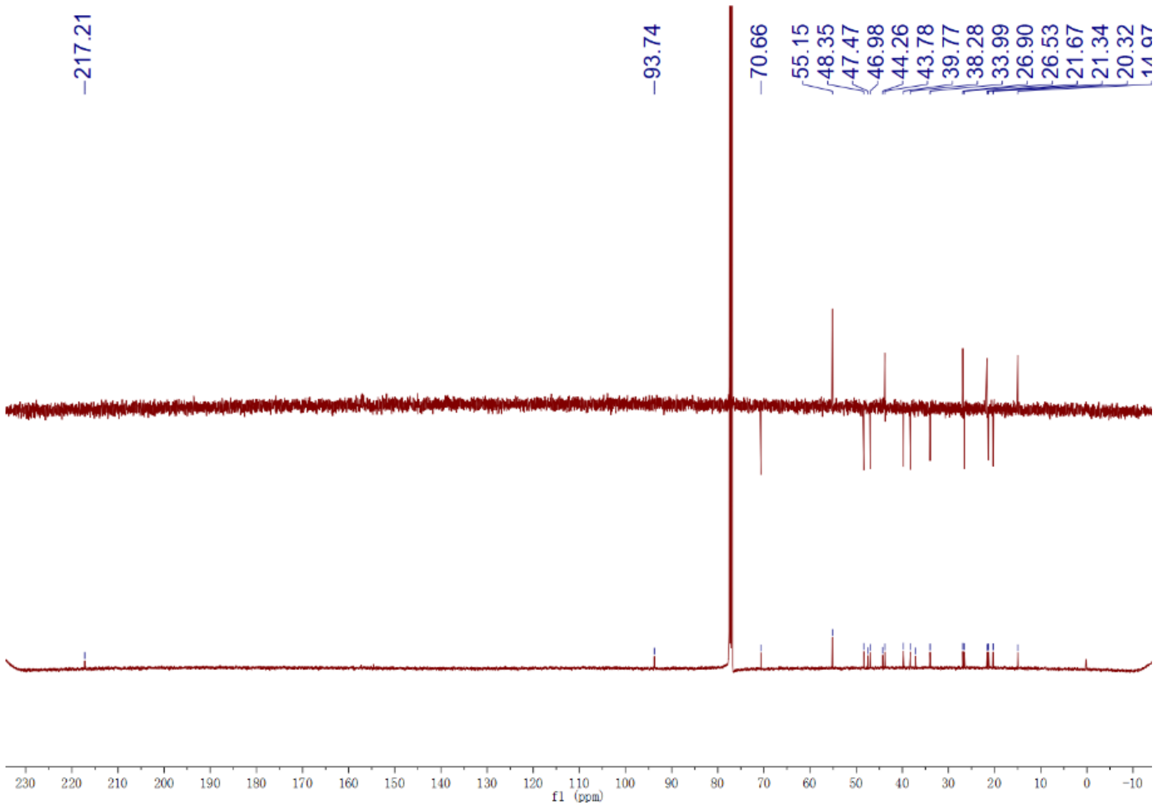


**Figure S38.** $^{13}C$ NMR and DEPT 135 spectrum of compound **11** in $CDCl_3$

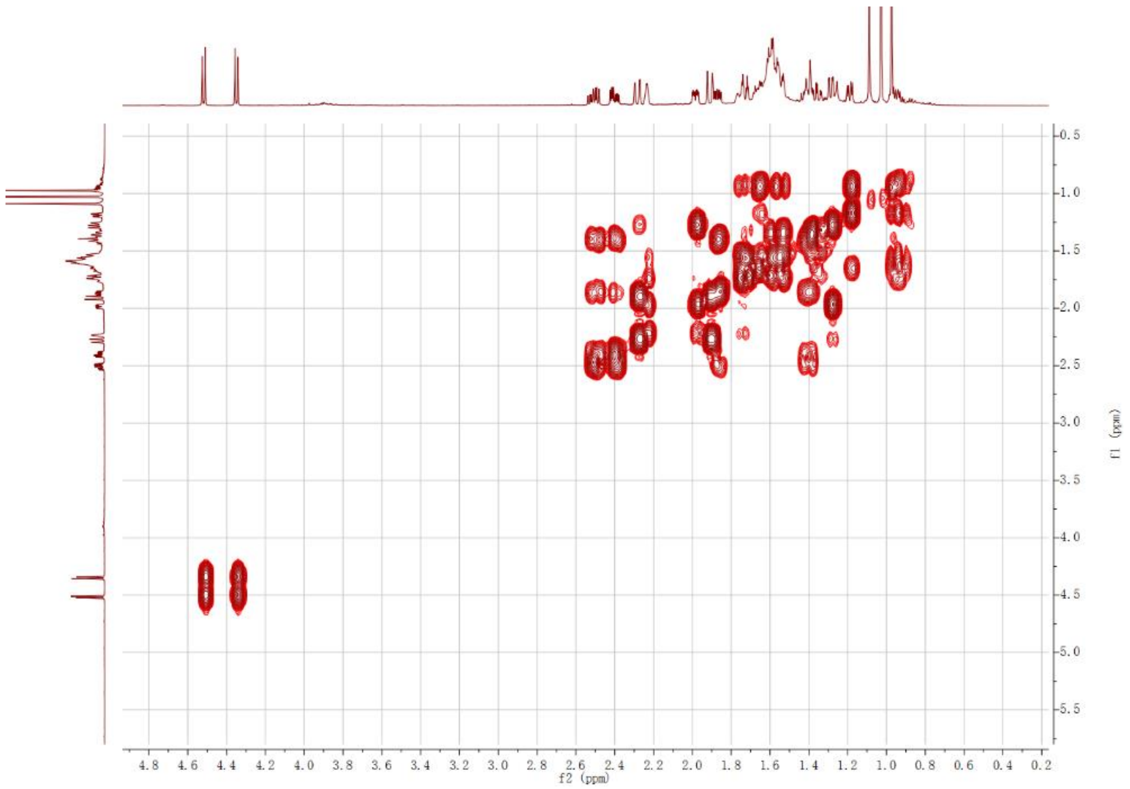


**Figure S39.** $^1H$-$^1H$ COSY spectrum of compound **11** in $CDCl_3$

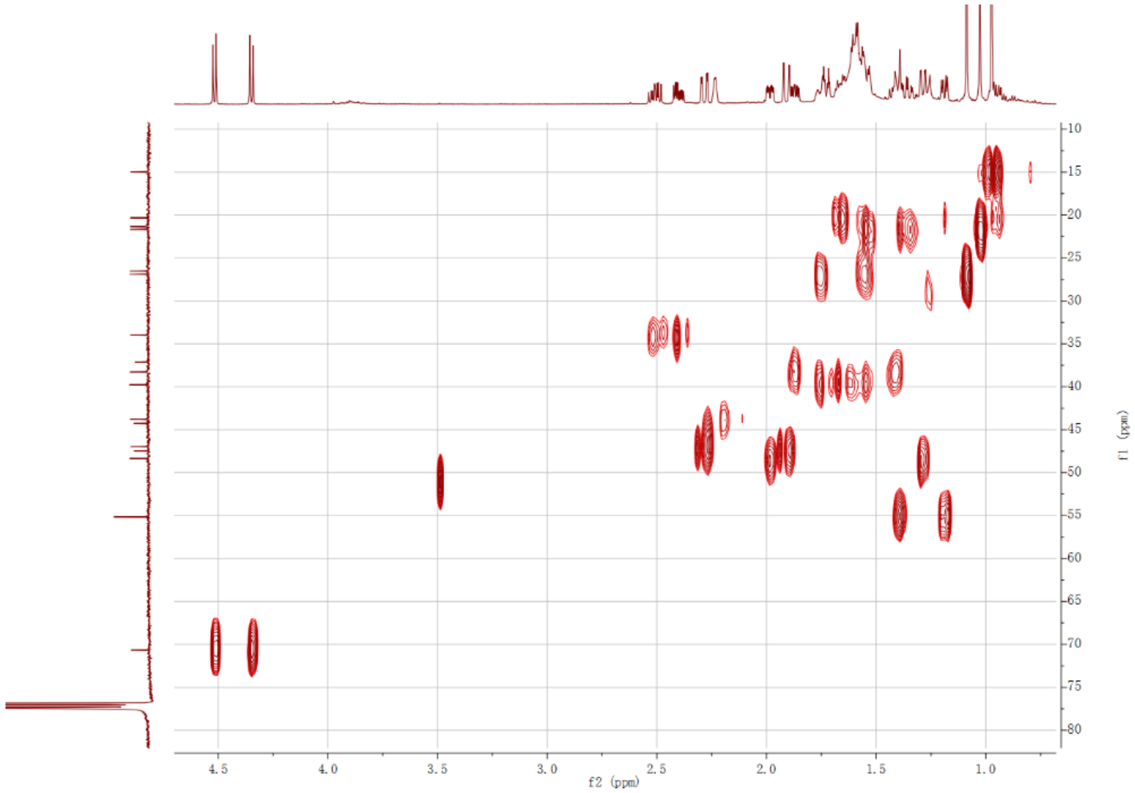


**Figure S40.** HSQC spectrum of compound **11** in $CDCl_3$

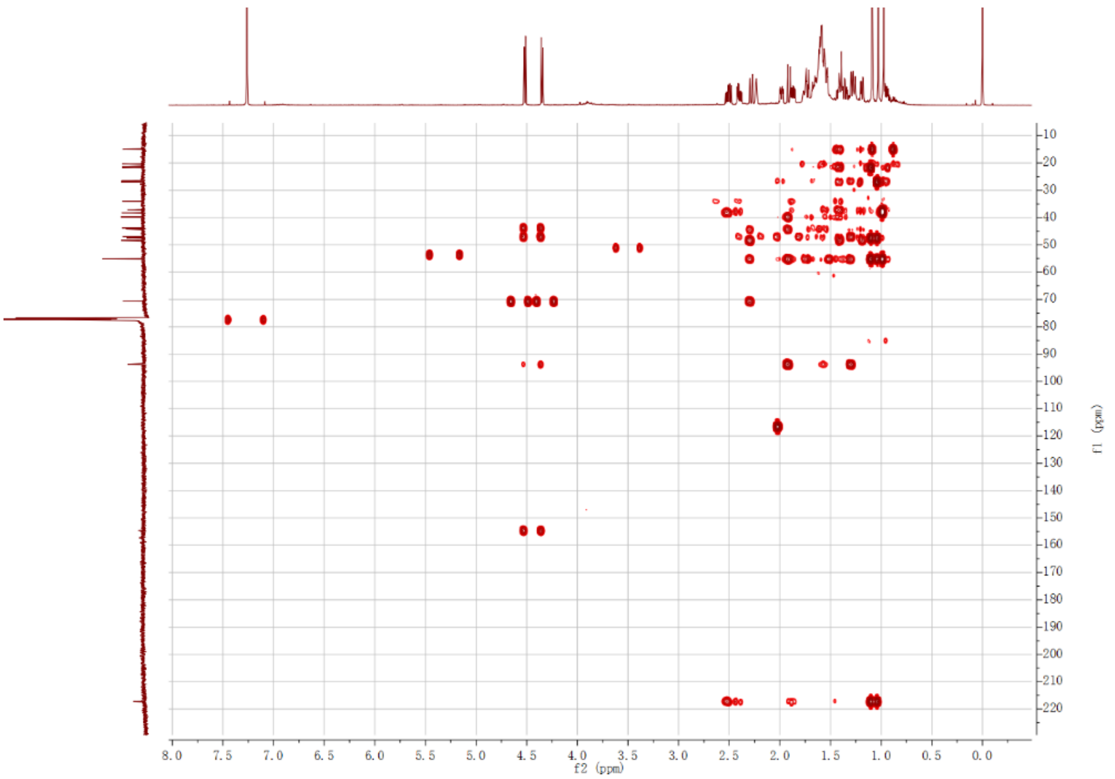


**Figure S41.** HMBC spectrum of compound **11** in $CDCl_3$

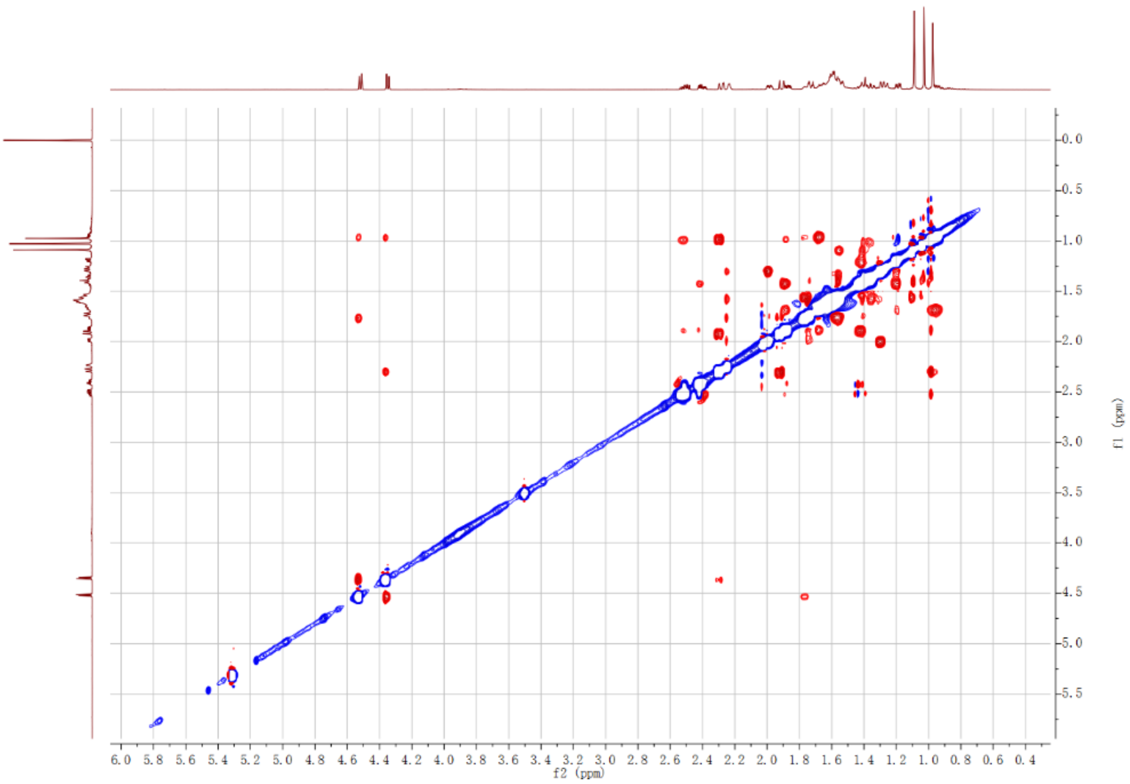


**Figure S42.** NOESY spectrum of compound **11** in $CDCl_3$

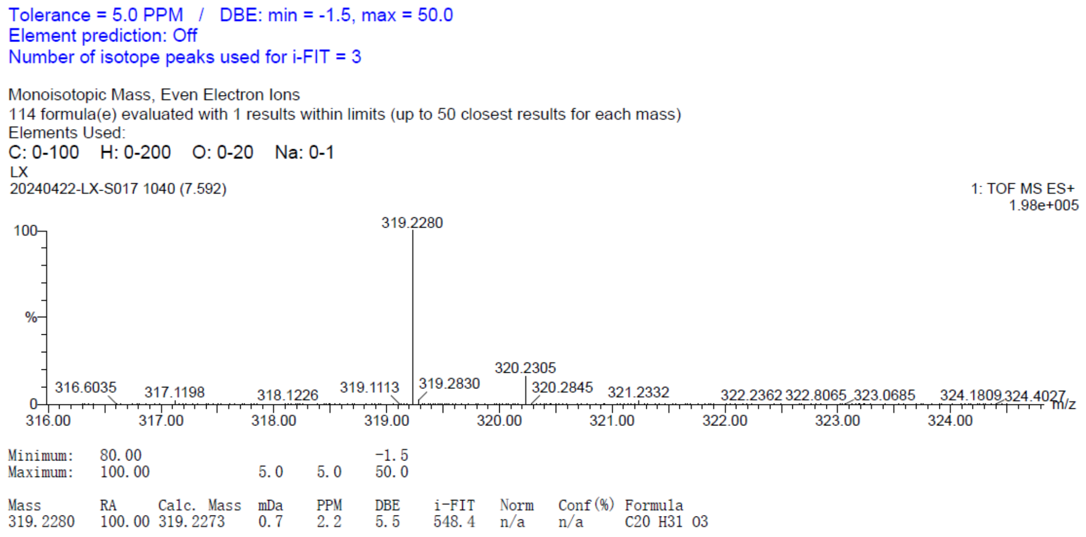


**Figure S43.** HRESIMS spectrum of compound **12**

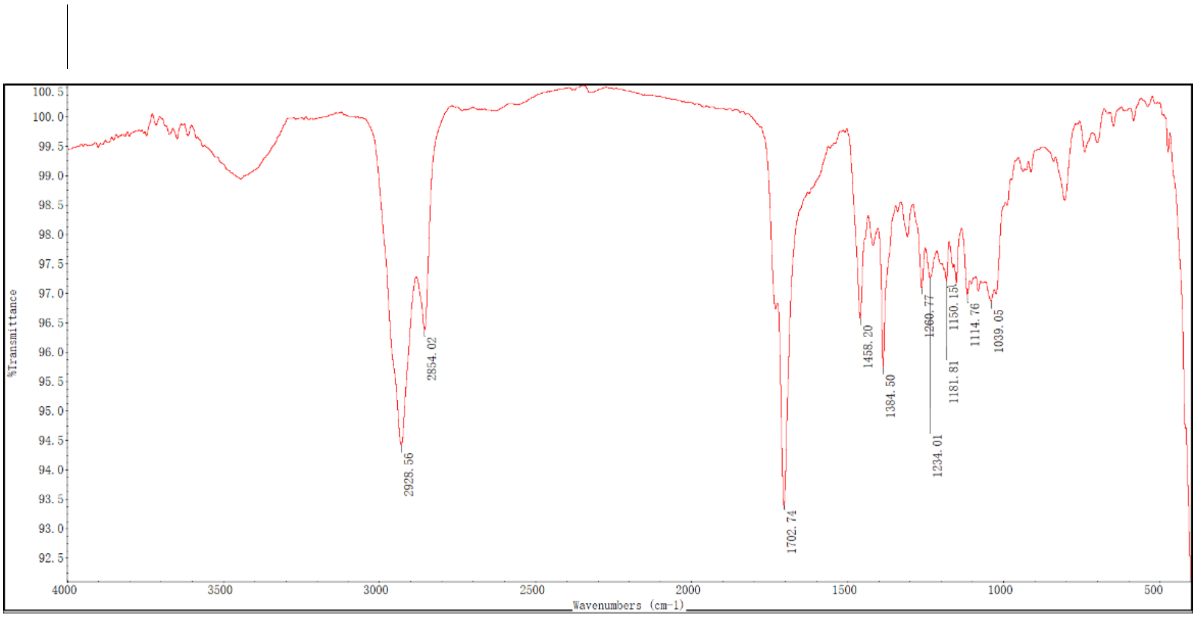


**Figure S44.** IR spectrum of compound **12**

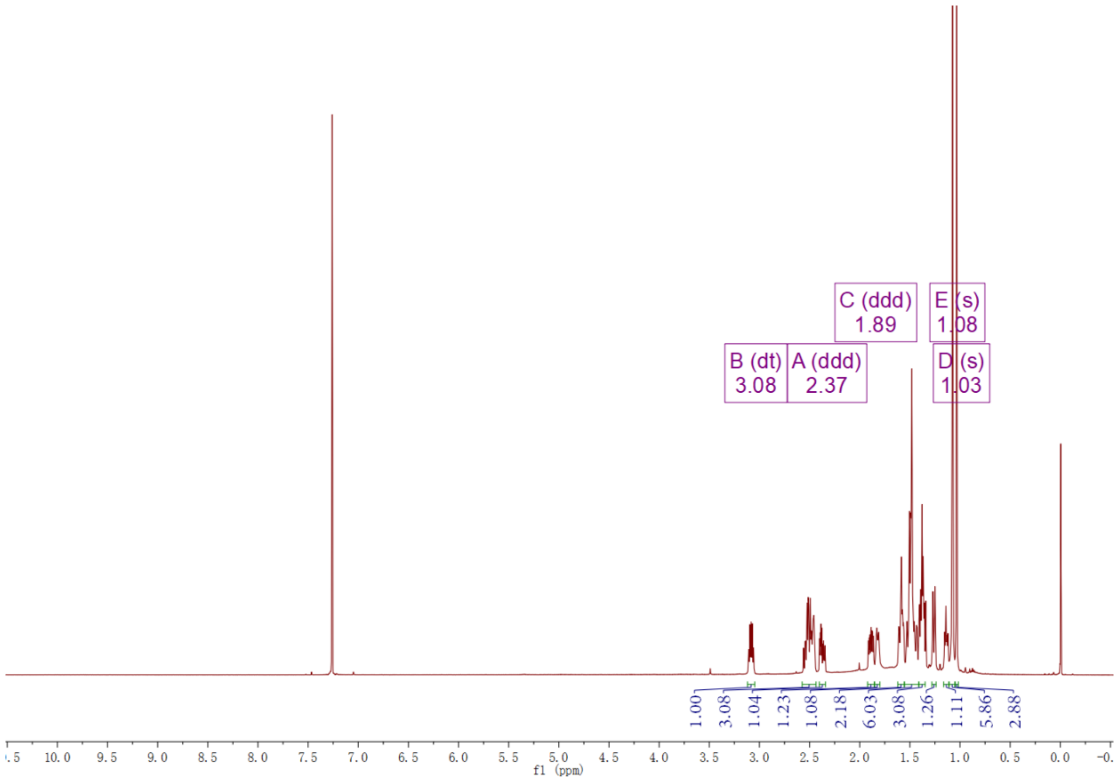


**Figure S45a.** $^1$H NMR spectrum of compound **12** in $CDCl_3$

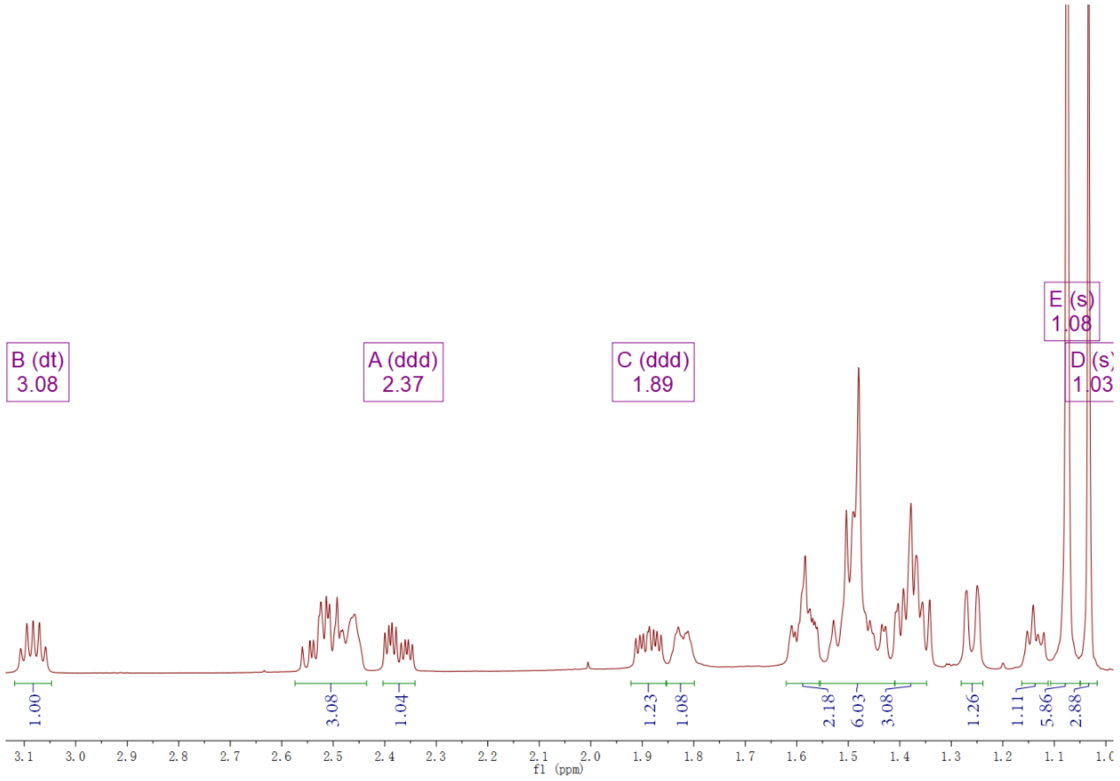


**Figure S45b.** Partial $^1$H NMR spectrum ($\delta$ 1.0-3.2 ppm) of compound **12** in $CDCl_3$

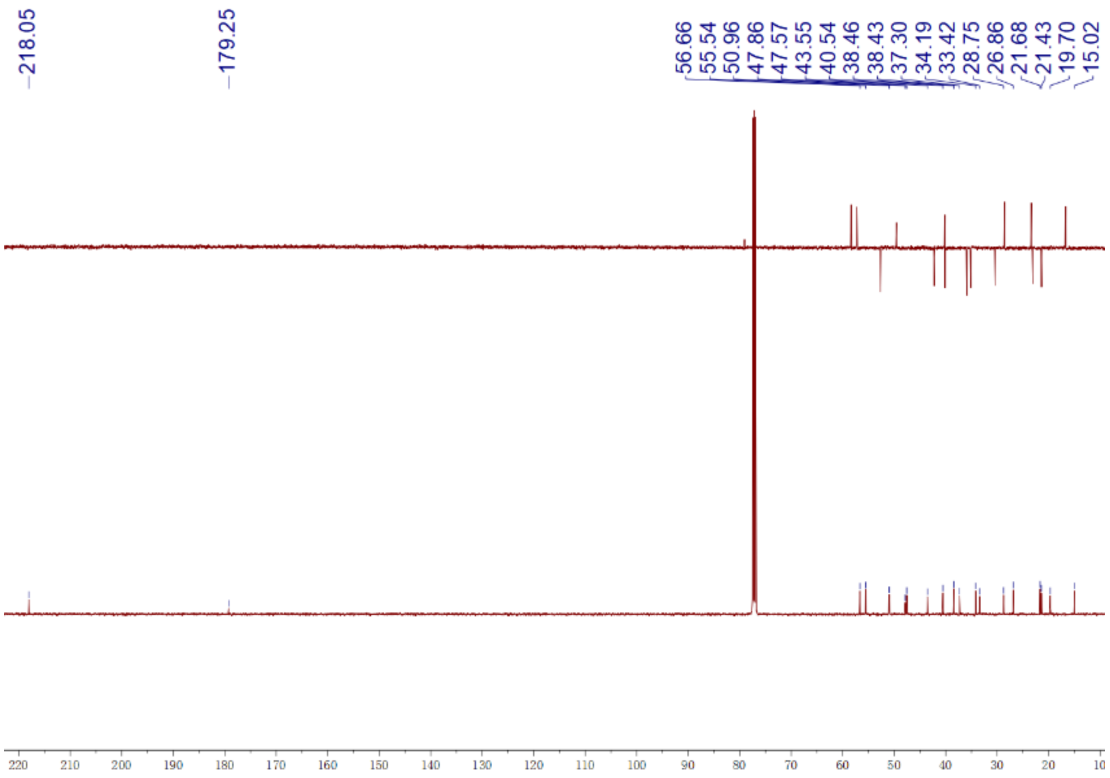



**Figure S46.** $^{13}C$ NMR and DEPT 135 spectrum of compound **12** in $CDCl_3$

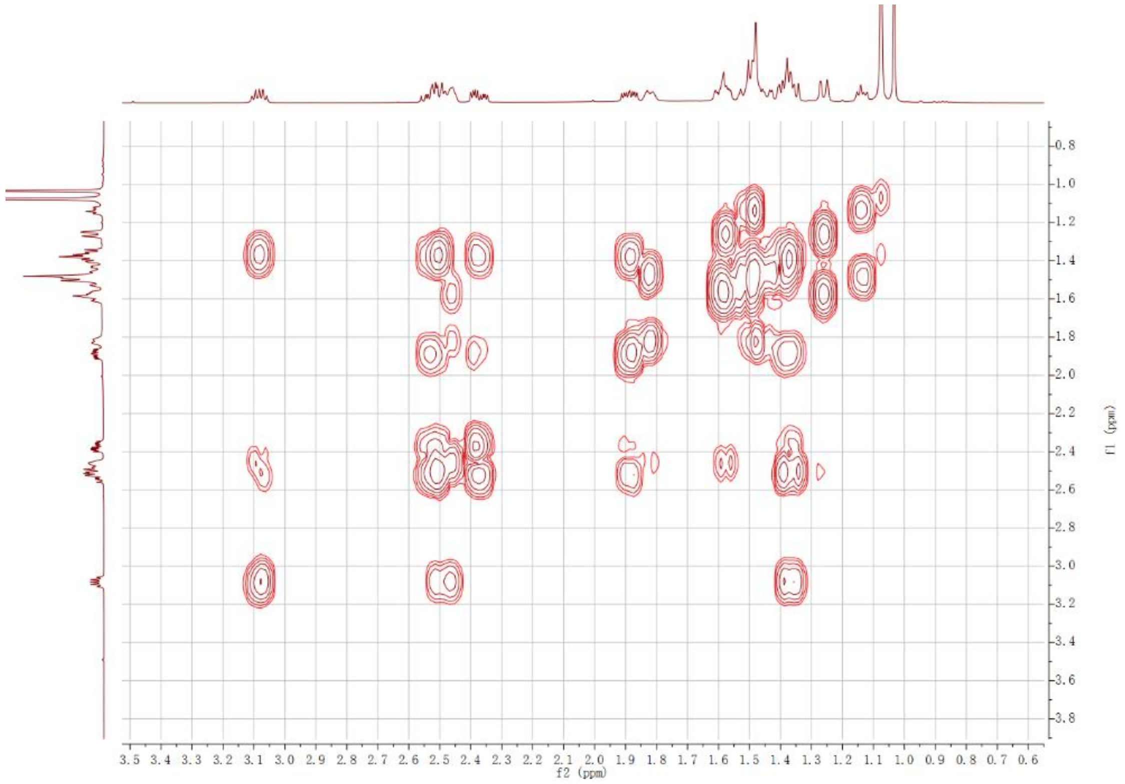



**Figure S47.** $^{1}H$-$^{1}H$ COSY spectrum of compound **12** in $CDCl_3$

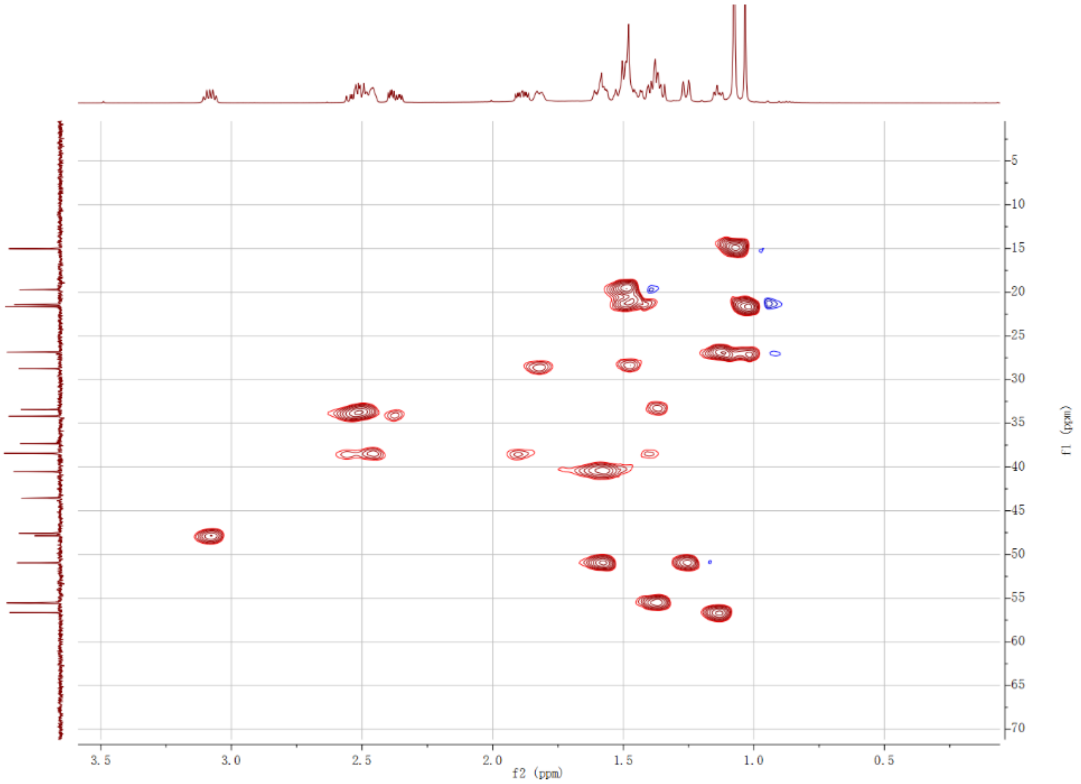


**Figure S48.** HSQC spectrum of compound **12** in $CDCl_3$

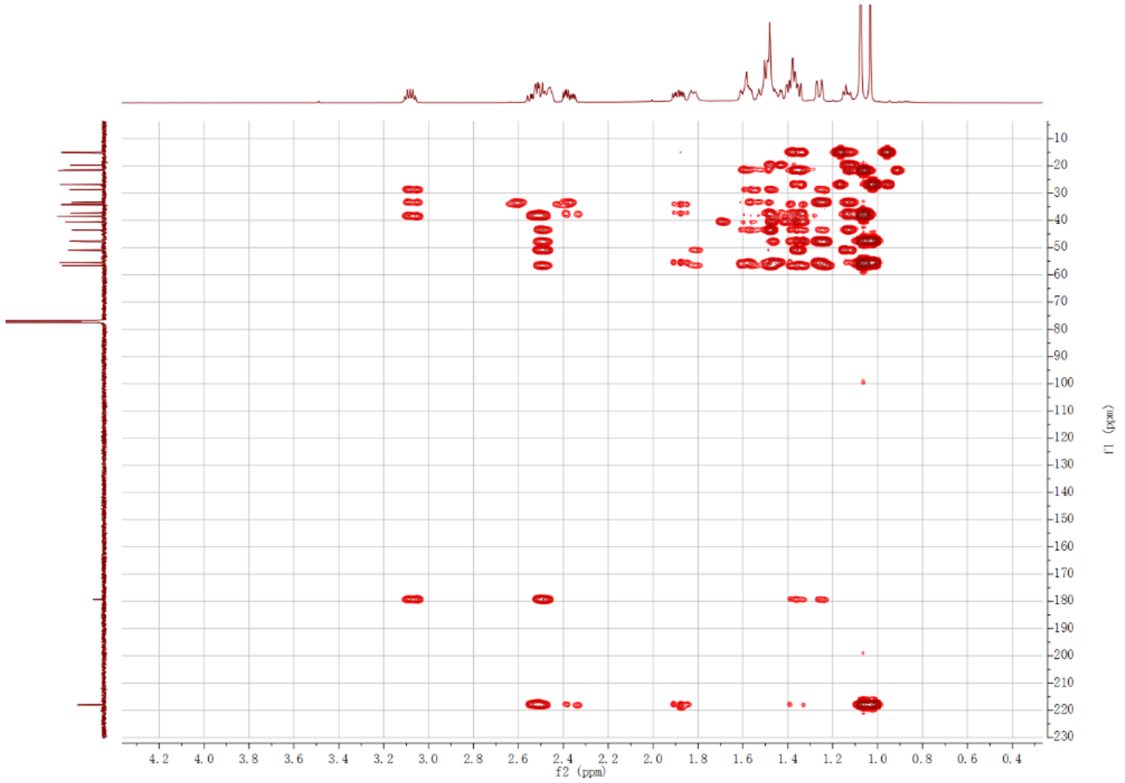


**Figure S49.** HMBC spectrum of compound **12** in $CDCl_3$

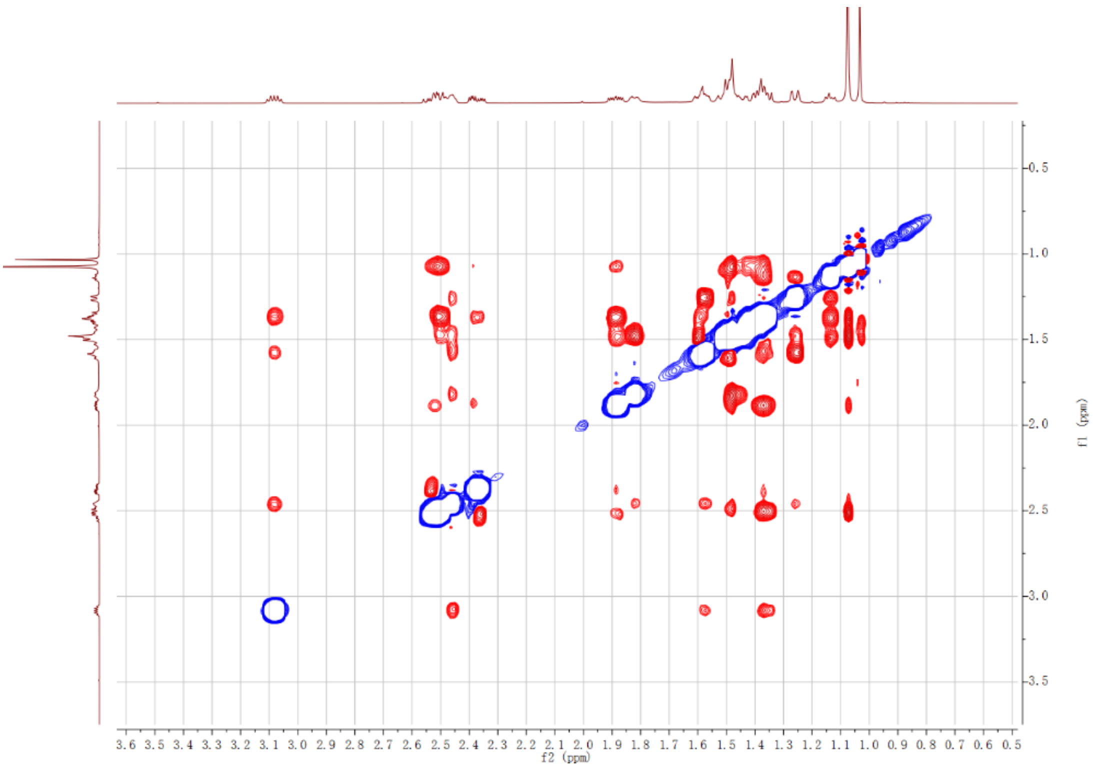


**Figure S50.** NOESY spectrum of compound **12** in $CDCl_3$

Tolerance = 5.0 PPM / DBE: min = -1.5, max = 50.0
Element prediction: Off
Number of isotope peaks used for i-FIT = 3

Monoisotopic Mass, Even Electron Ions
105 formula(e) evaluated with 1 results within limits (up to 50 closest results for each mass)
Elements Used:
C: 0-100 H: 0-200 O: 0-20 Na: 0-1
LX
20240422-LX-S015 1060 (7.745)

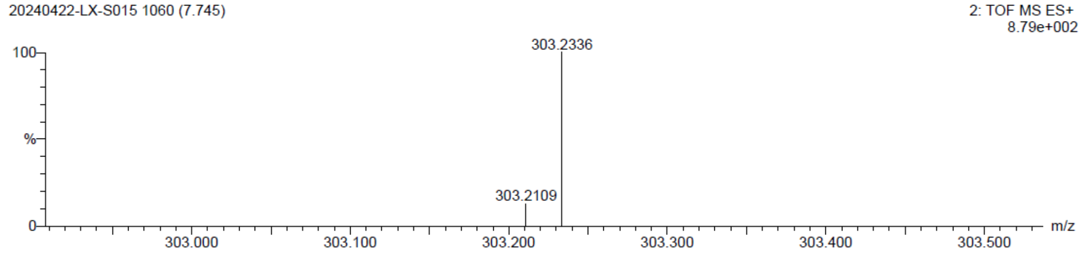


| | | | | | | | | | |
|---|---|---|---|---|---|---|---|---|---|
| Minimum: | 80.00 | | | | -1.5 | | | | |
| Maximum: | 100.00 | | 5.0 | 5.0 | 50.0 | | | | |
| Mass | RA | Calc. Mass | mDa | PPM | DBE | i-FIT | Norm | Conf(%) | Formula |
| 303.2336 | 100.00 | 303.2324 | 1.2 | 4.0 | 5.5 | 24.6 | n/a | n/a | C20 H31 O2 |

**Figure S51.** HRESIMS spectrum of compound **13**

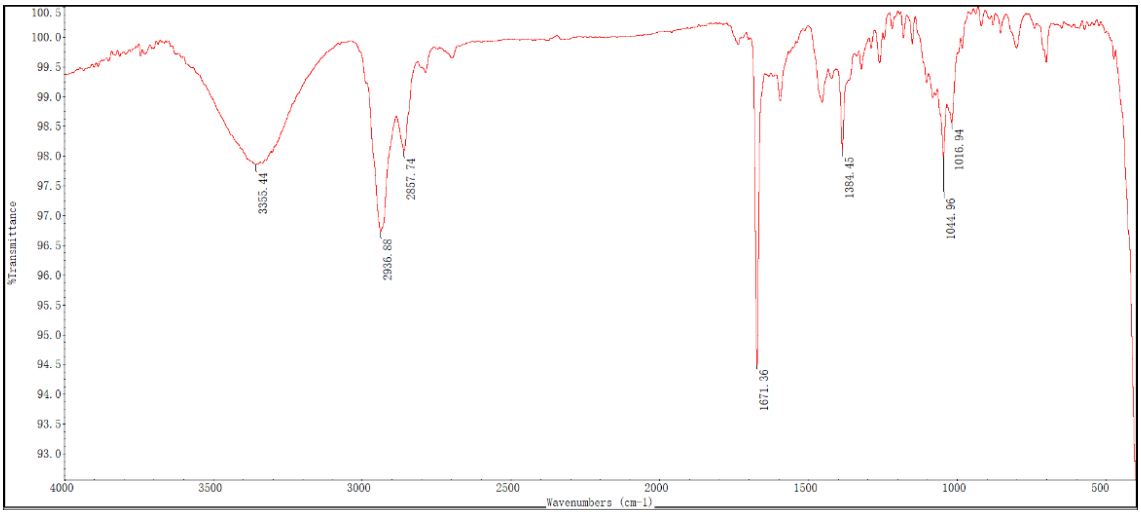


**Figure S52.** IR spectrum of compound **13**

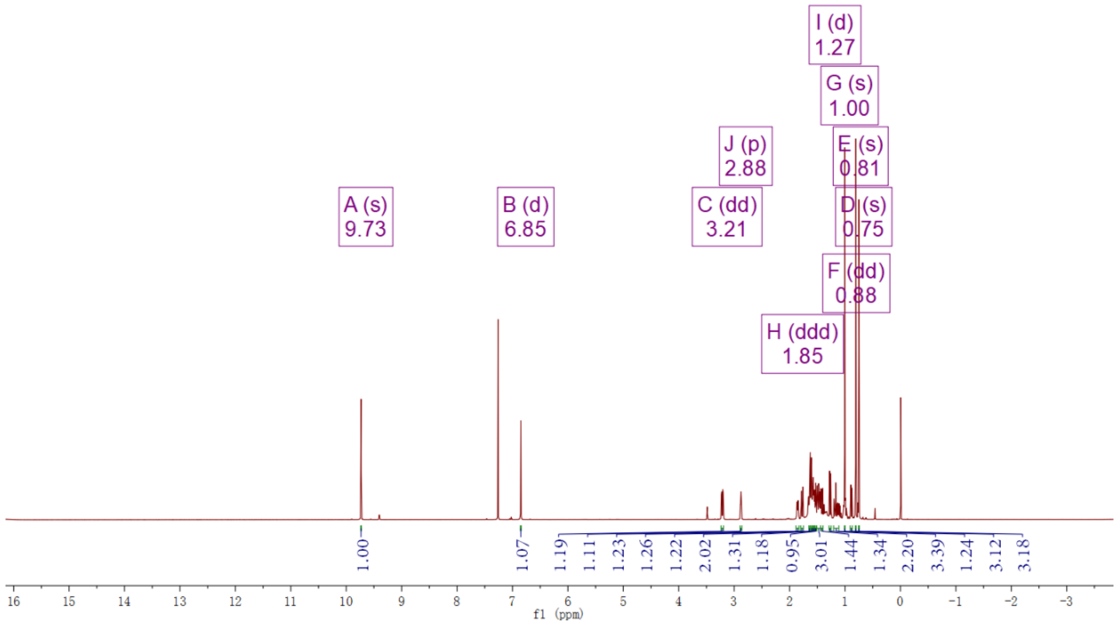


**Figure S53a.** $^1$H NMR spectrum of compound **13** in $CDCl_3$

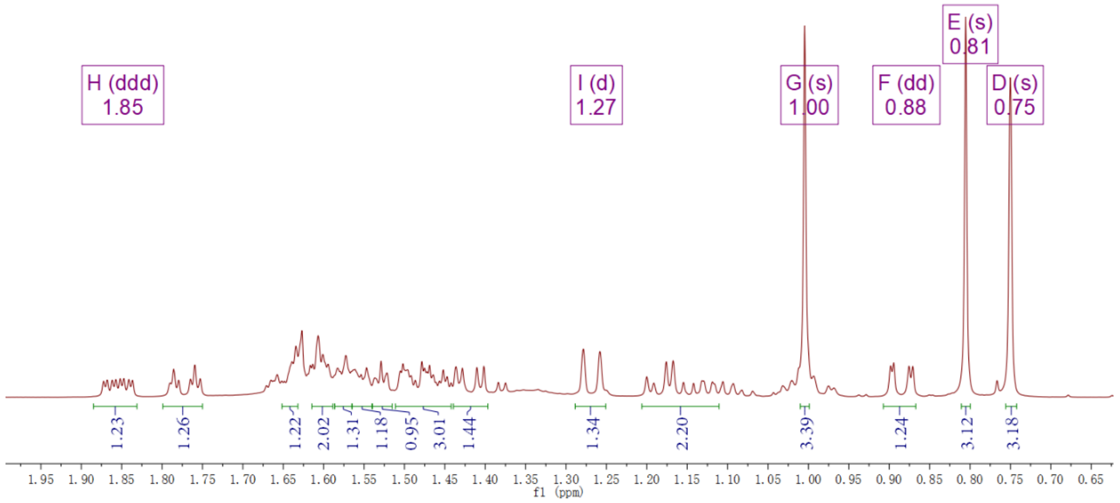


**Figure S53b.** $^{1}$H NMR spectrum ($\delta$ 0.7-1.9 ppm) of compound **13** in $CDCl_3$

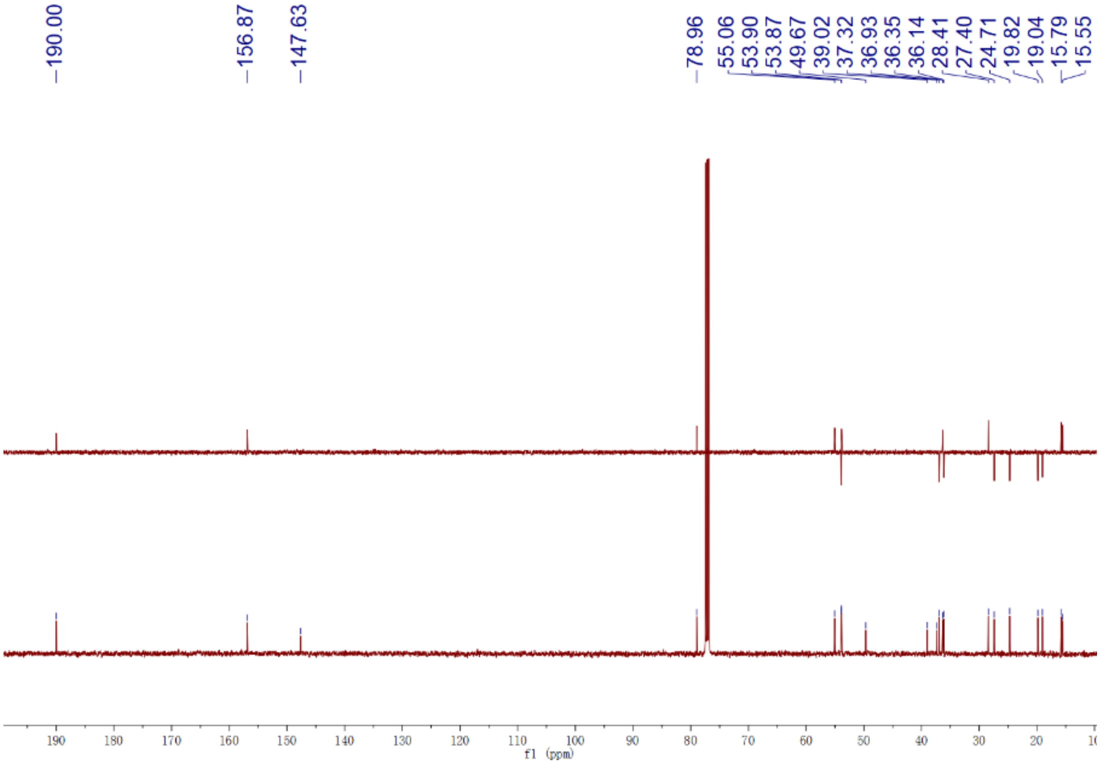


**Figure S54.** $^{13}$C NMR and DEPT 135 spectrum of compound **13** in $CDCl_3$

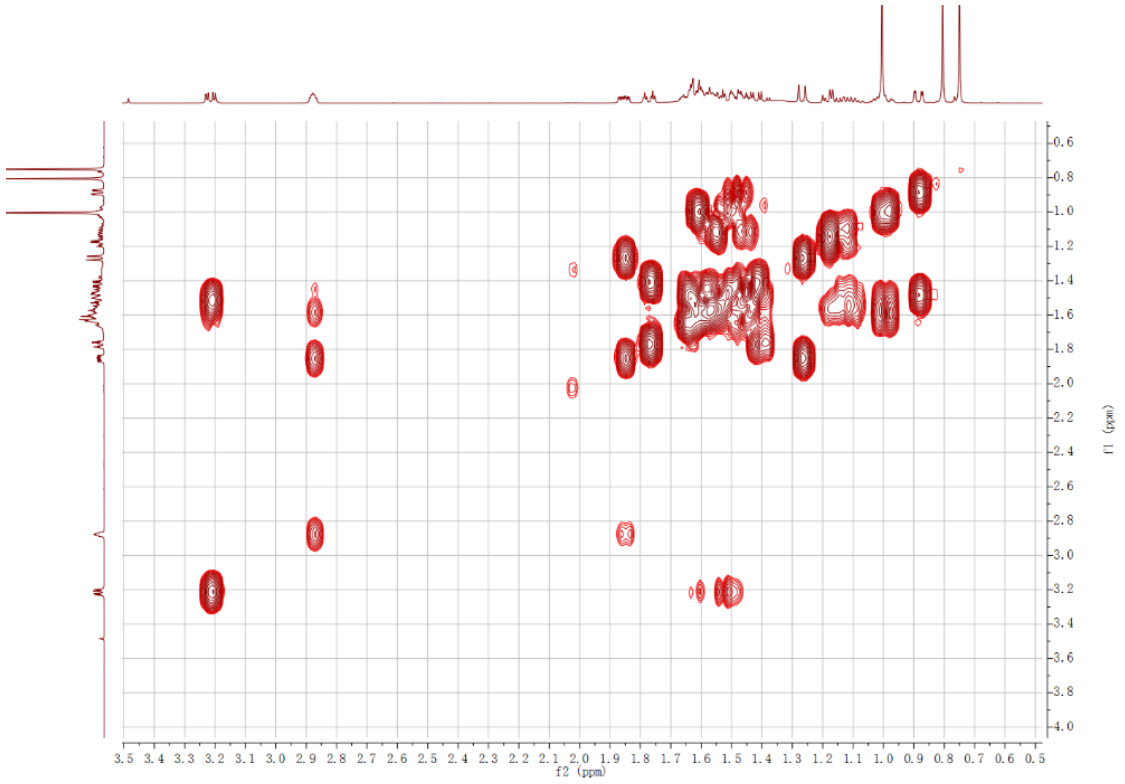


**Figure S55.** $^{1}H$-$^{1}H$ COSY spectrum of compound **13** in $CDCl_3$

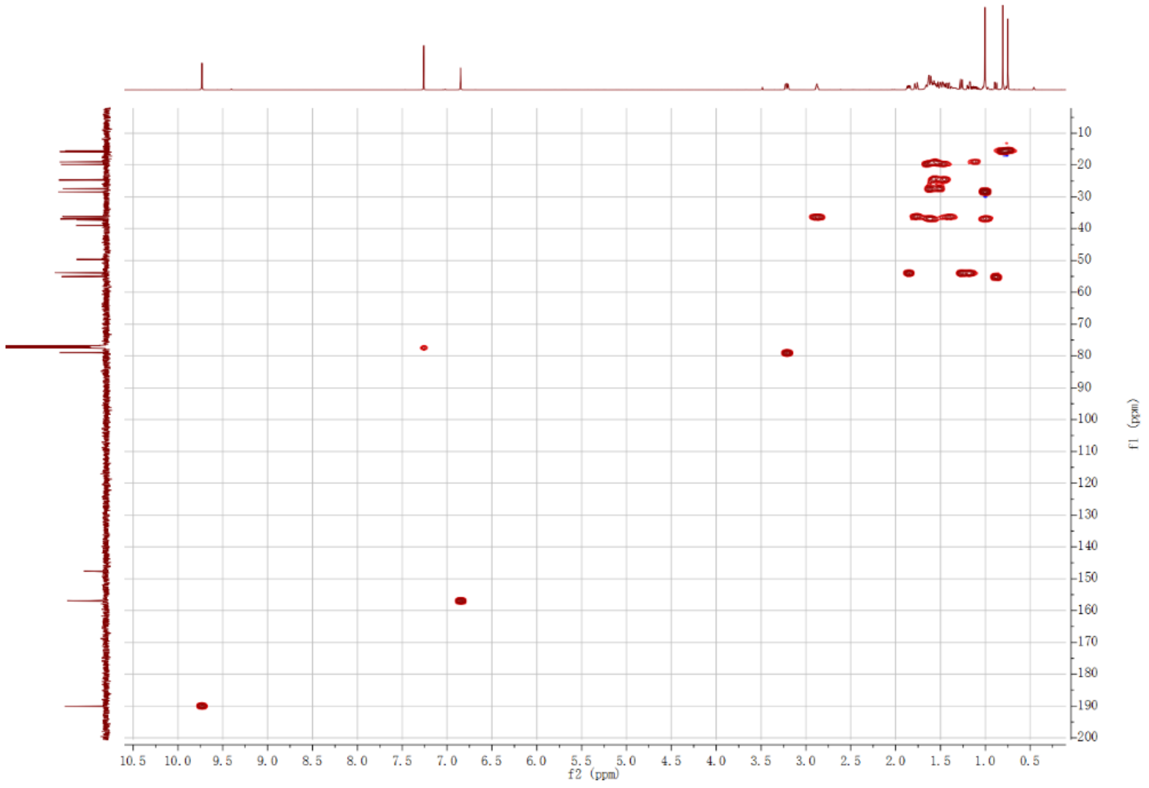


**Figure S56.** HSQC spectrum of compound **13** in $CDCl_3$

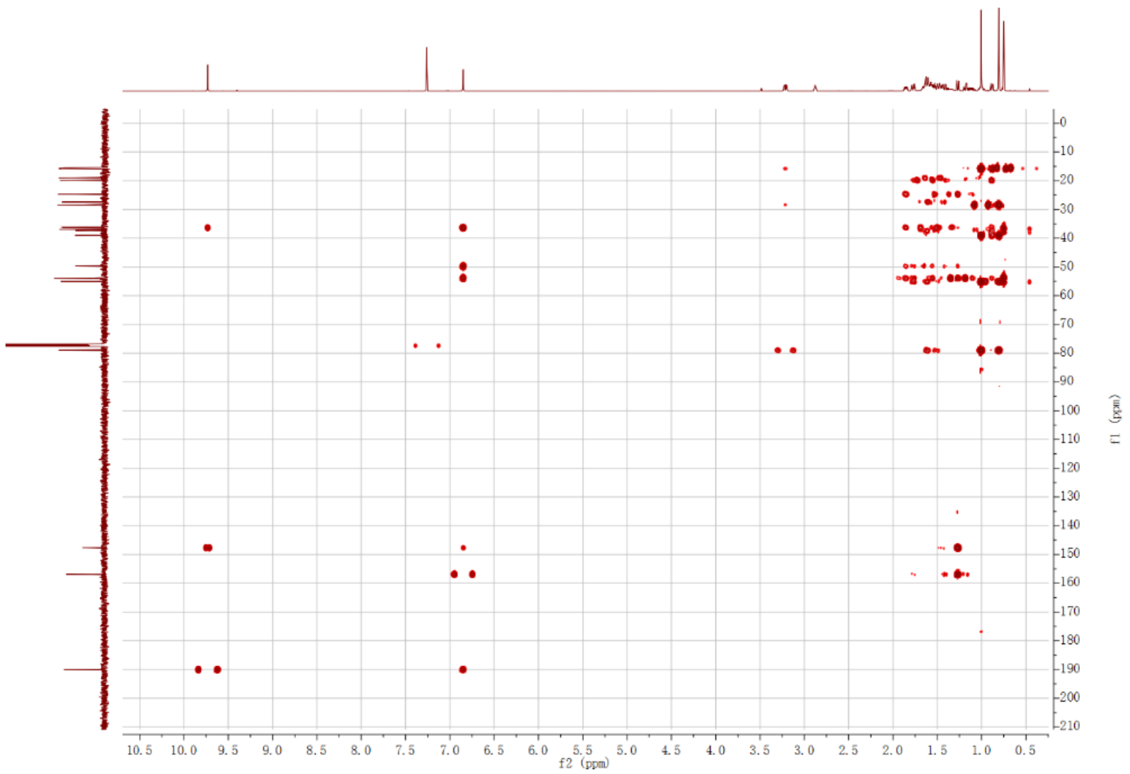


**Figure S57.** HMBC spectrum of compound **13** in $CDCl_3$

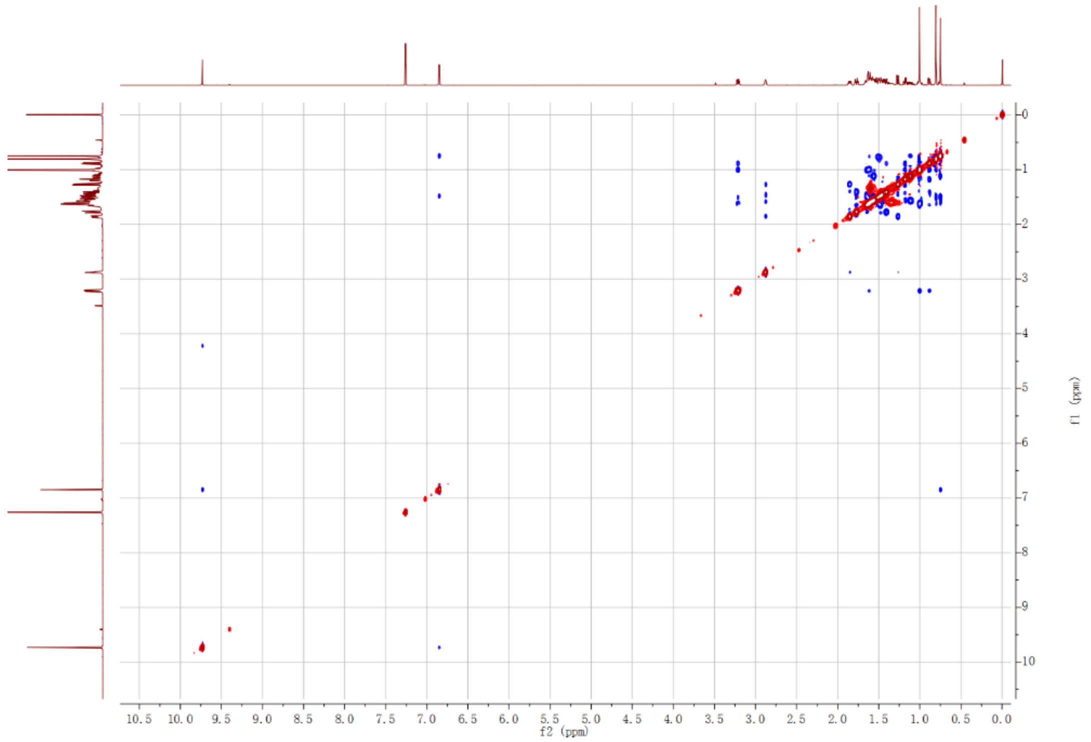


**Figure S58.** NOESY spectrum of compound **13** in $CDCl_3$

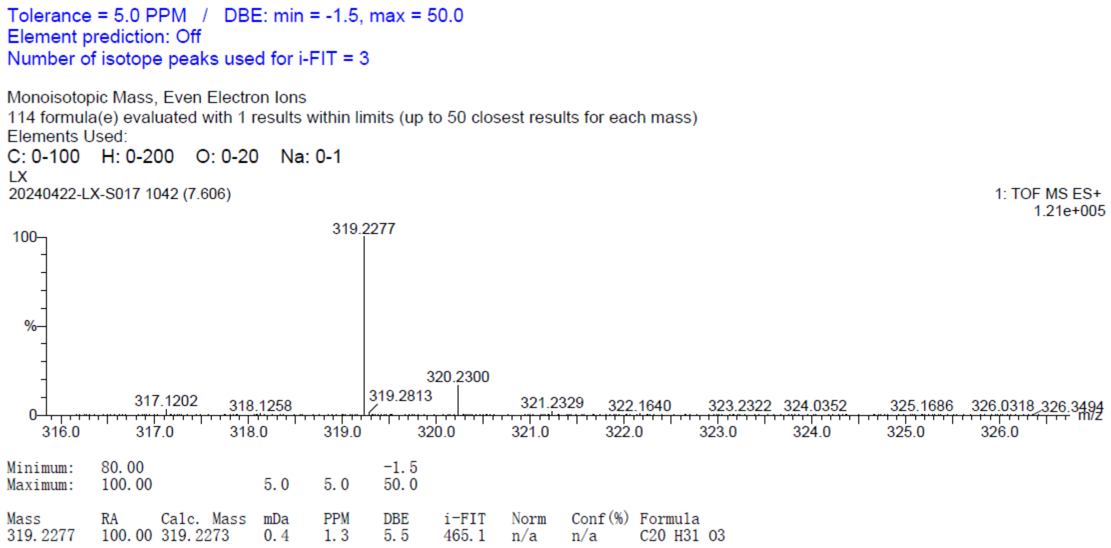


**Figure S59.** HRESIMS spectrum of compound **14**

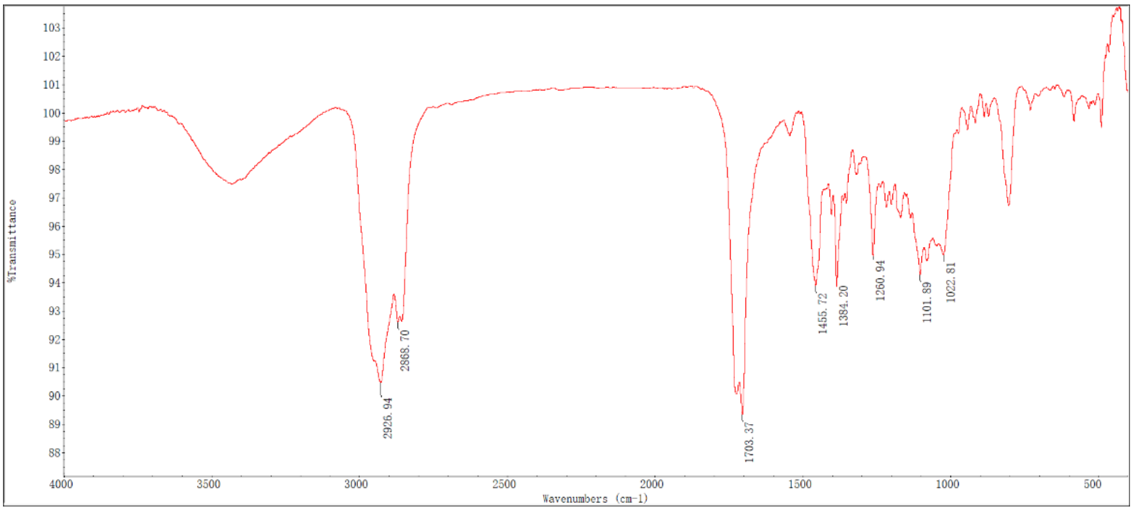


**Figure S60.** IR spectrum of compound **14**

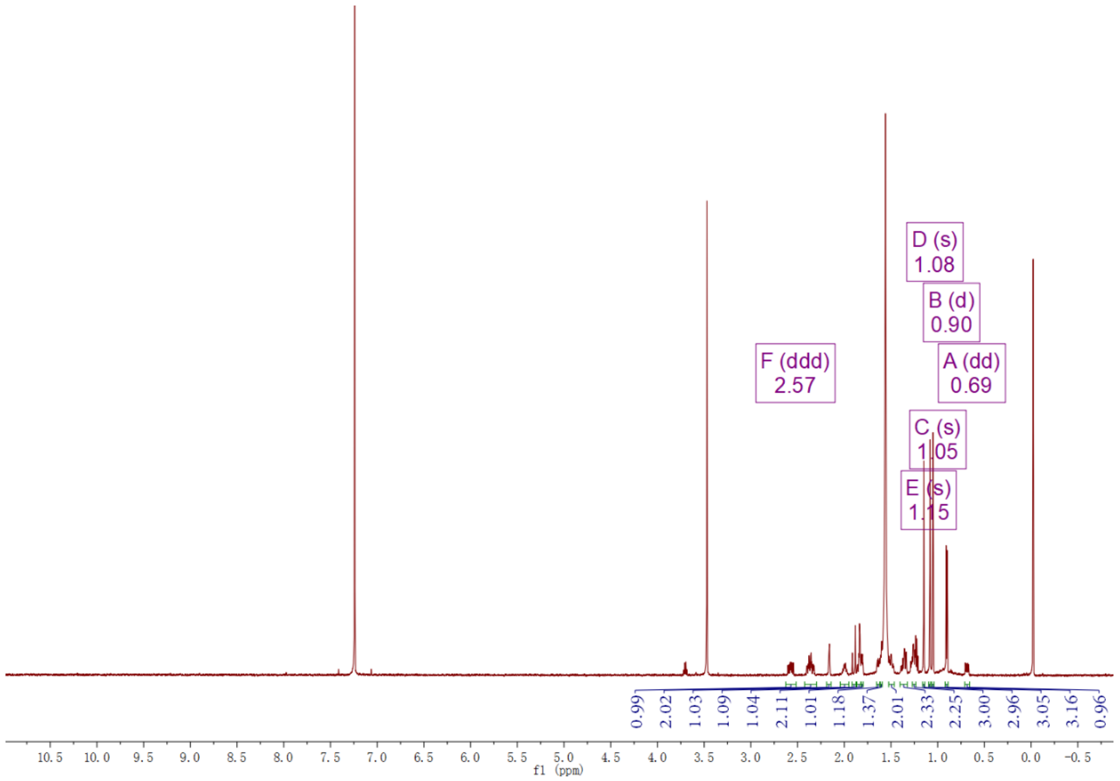


**Figure S61a.** $^{1}$H NMR spectrum of compound **14** in $CDCl_3$

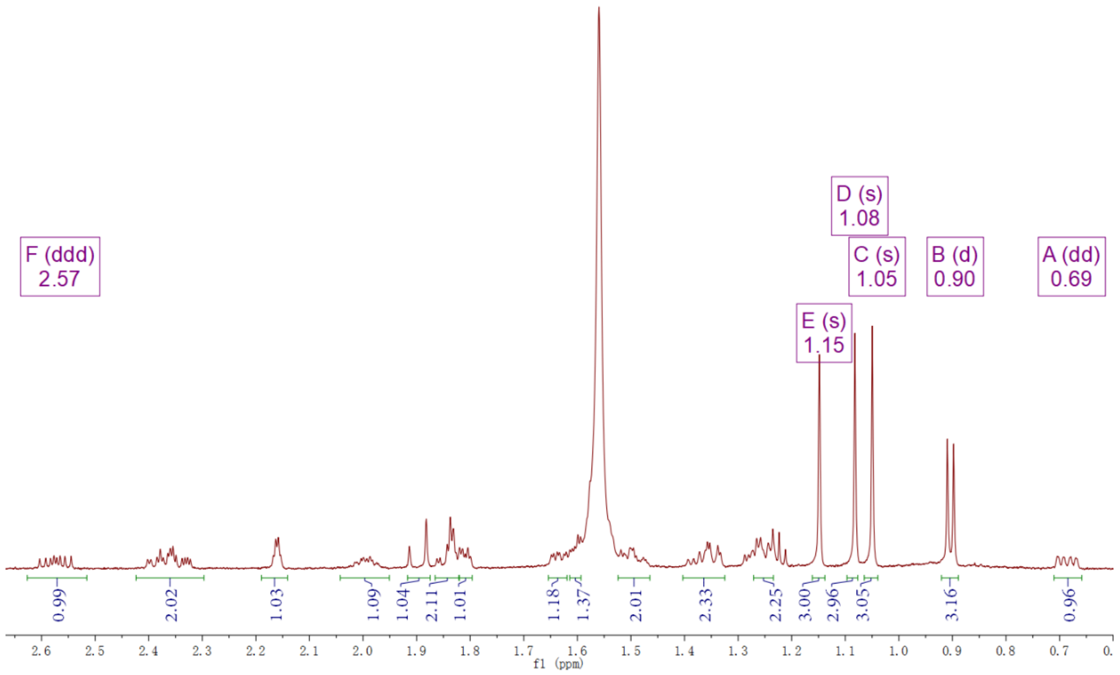


**Figure S61b.** Partial $^{1}$H NMR spectrum ($\delta$ 0.6-2.7 ppm) of compound **14** in $CDCl_3$

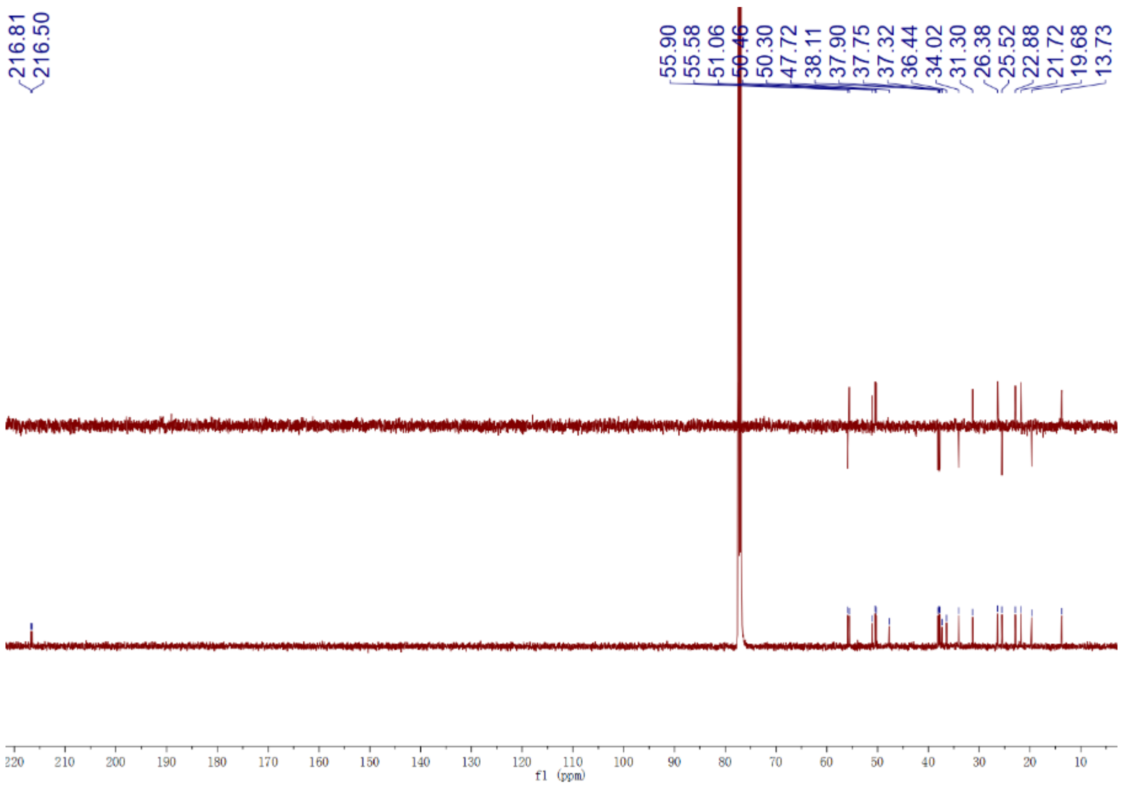


**Figure S62.** $^{13}$C NMR and DEPT 135 spectrum of compound **14** in $CDCl_3$

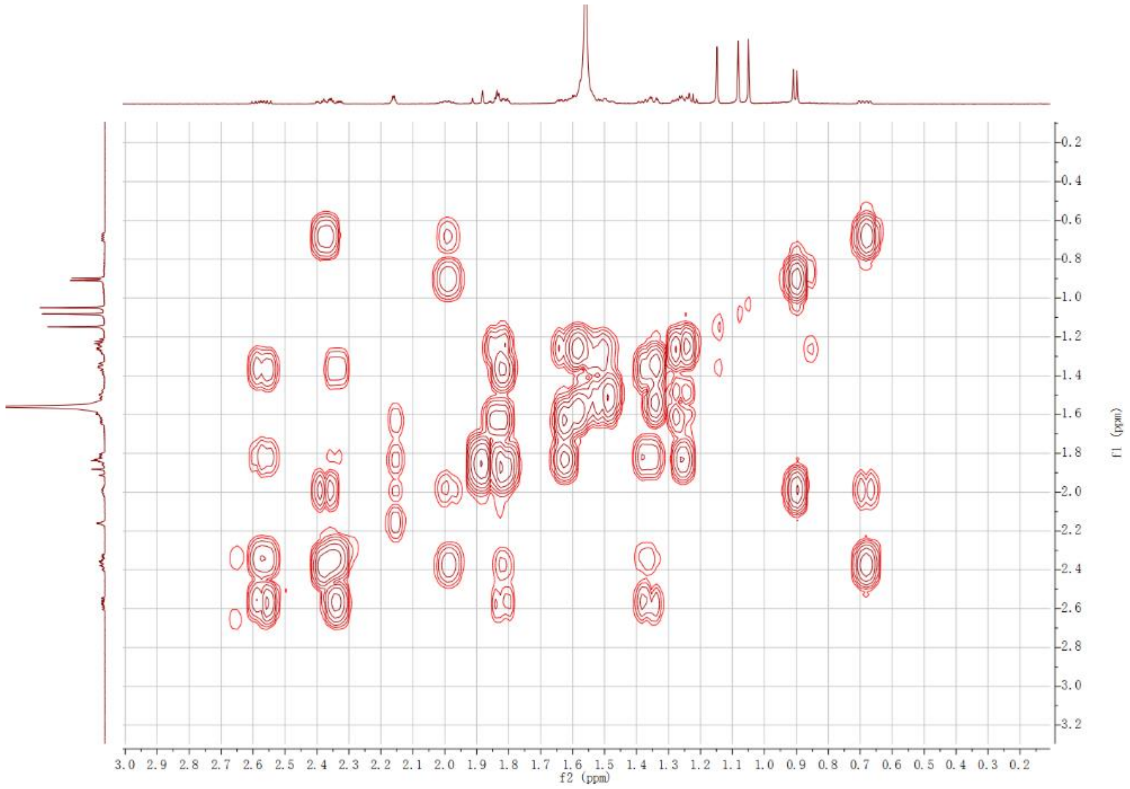


**Figure S63.** $^{1}$H-$^{1}$H COSY spectrum of compound **14** in $CDCl_3$

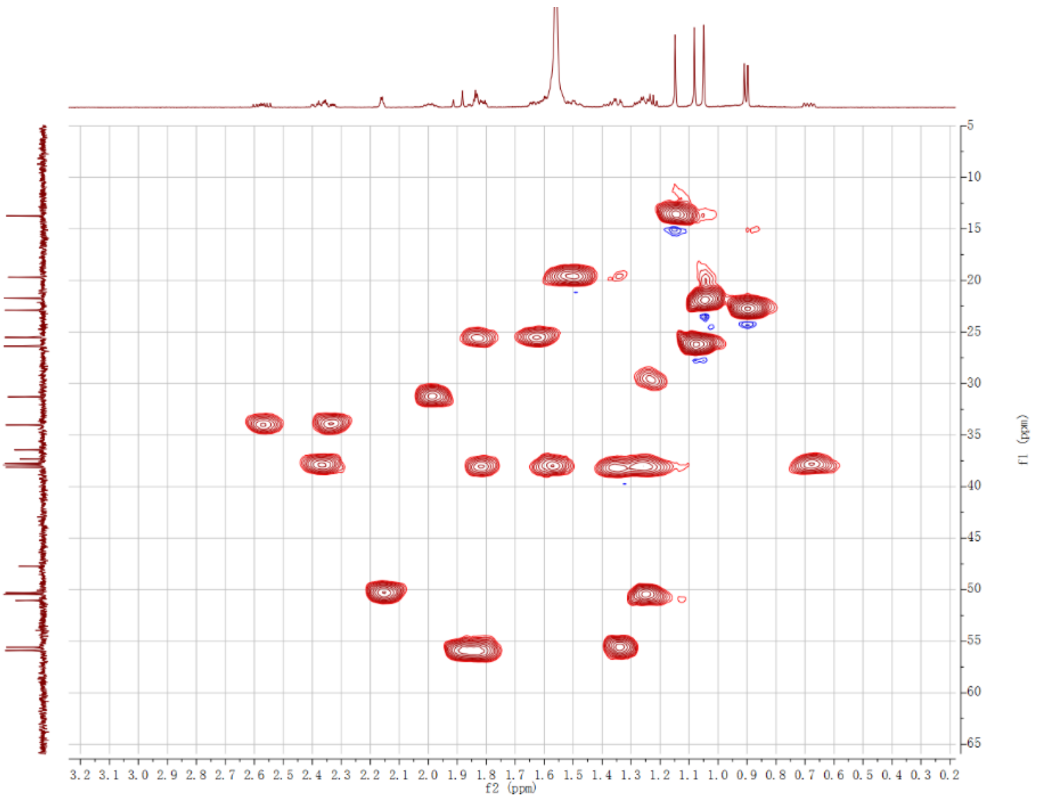


**Figure S64.** HSQC spectrum of compound **14** in $CDCl_3$

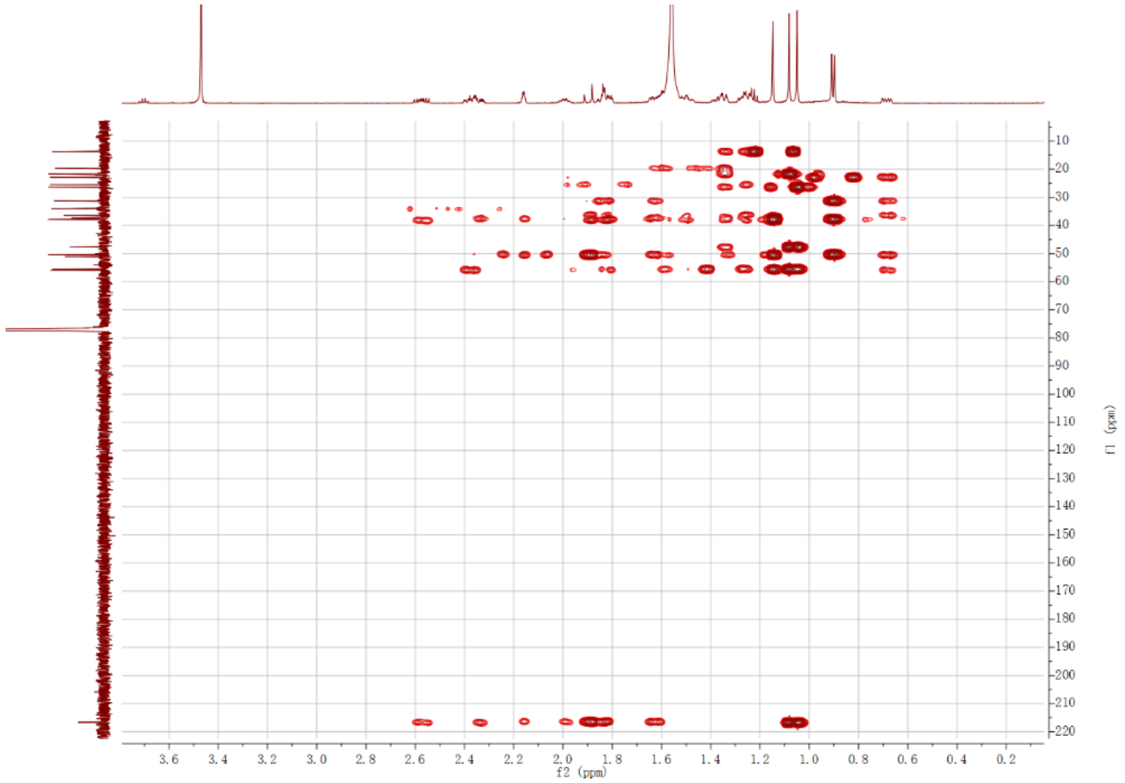


**Figure S65.** HMBC spectrum of compound **14** in $CDCl_3$

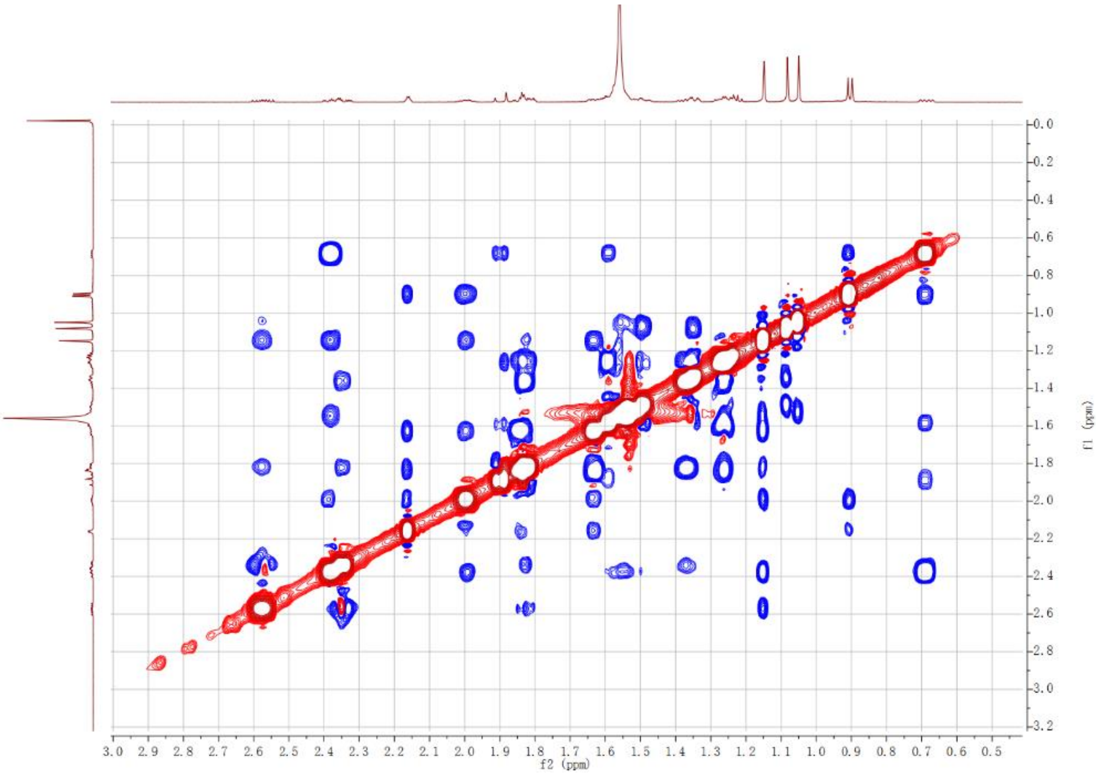


**Figure S66.** NOESY spectrum of compound **14** in $CDCl_3$

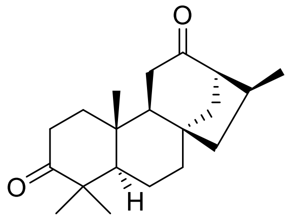


(5*R*, 8*R*, 9*S*, 10*R*, 13*R*, 16*S**)

14a

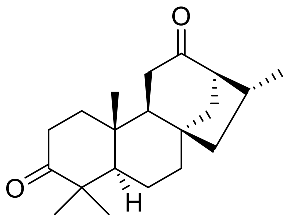


(5*R*, 8*R*, 9*S*, 10*R*, 13*R*, 16*R**)

14b

**Figure S67.** Two possible conformations of compound **14**

| Functional | Solvent? | Basis Set | Type of Data | | |
|---|---|---|---|---|---|
| mPW1PW91 | Gas Phase | 6-311G(d,p) | Shielding Tensors | | |

| | Isomer 1 | Isomer 2 | Isomer 3 | Isomer 4 | Isomer 5 | Isomer 6 |
|---|---|---|---|---|---|---|
| sDP4+ (H data) | 100.00% | 0.00% | – | – | – | – |
| sDP4+ (C data) | 14.78% | 85.22% | – | – | – | – |
| sDP4+ (all data) | 100.00% | 0.00% | – | – | – | – |
| uDP4+ (H data) | 97.02% | 2.98% | – | – | – | – |
| uDP4+ (C data) | 95.34% | 4.66% | – | – | – | – |
| uDP4+ (all data) | 99.85% | 0.15% | – | – | – | – |
| DP4+ (H data) | 100.00% | 0.00% | – | – | – | – |
| DP4+ (C data) | 78.01% | 21.99% | – | – | – | – |
| DP4+ (all data) | 100.00% | 0.00% | – | – | – | – |

**Figure S68.** DP4+ probability analysis result for compound **14** (14a : *rel*- 5*R*, 8*R*, 9*S*, 10*R*, 13*R*, 16*S*)

Tolerance = 5.0 PPM / DBE: min = -1.5, max = 50.0
Element prediction: Off
Number of isotope peaks used for i-FIT = 3

Monoisotopic Mass, Even Electron Ions
131 formula(e) evaluated with 1 results within limits (up to 50 closest results for each mass)
Elements Used:
C: 0-100 H: 0-200 O: 0-20 Na: 0-1
LX
20240422-LX-S005 781 (5.703) 1: TOF MS ES+
2.04e+004

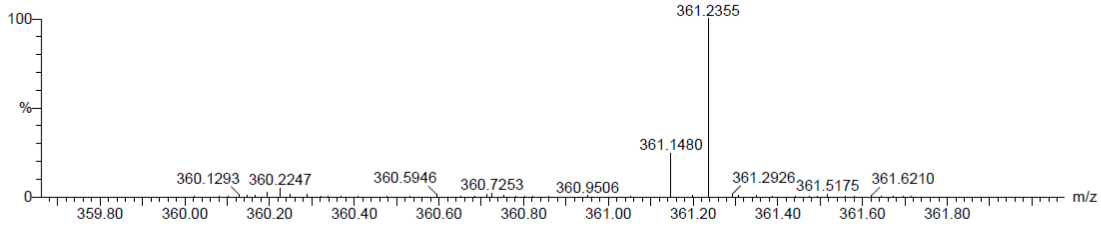


| | | | | | | | | | |
|---|---|---|---|---|---|---|---|---|---|
| Minimum: | 80.00 | | | | -1.5 | | | | |
| Maximum: | 100.00 | | 5.0 | 5.0 | 50.0 | | | | |
| Mass | RA | Calc. Mass | mDa | PPM | DBE | i-FIT | Norm | Conf(%) | Formula |
| 361.2355 | 100.00 | 361.2355 | 0.0 | 0.0 | 3.5 | 154.4 | n/a | n/a | C20 H34 O4 Na |

**Figure S69.** HRESIMS spectrum of compound **15**

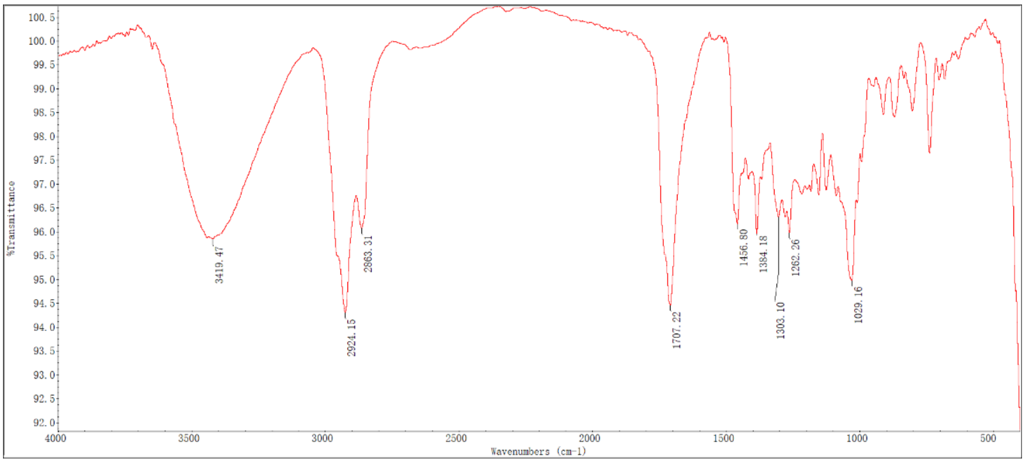


**Figure S70.** IR spectrum of compound **15**

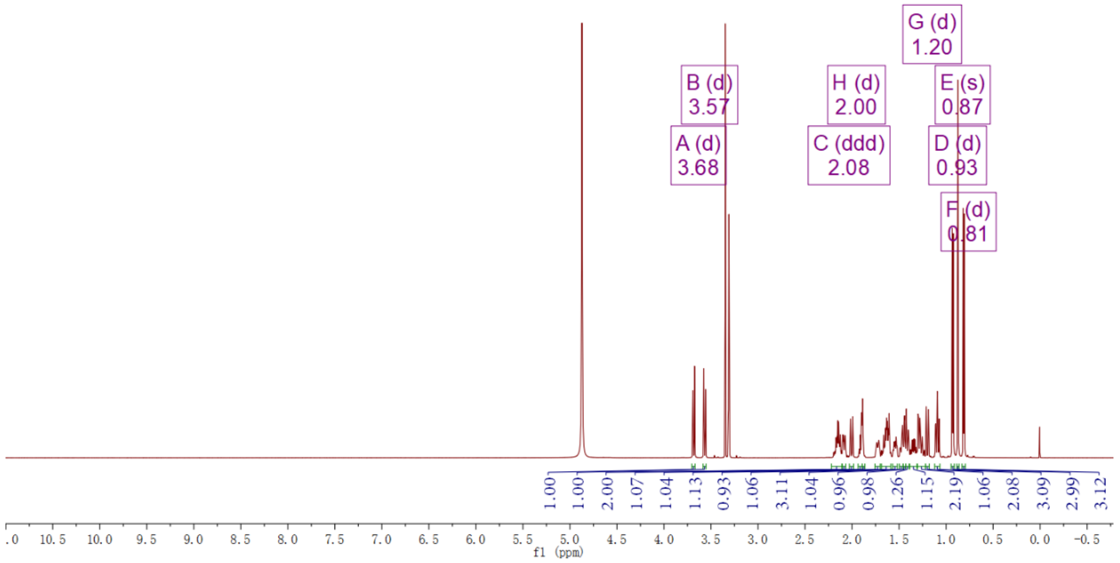


**Figure S71a.** $^{1}H$ NMR spectrum of compound **15** in $CD_3OD$

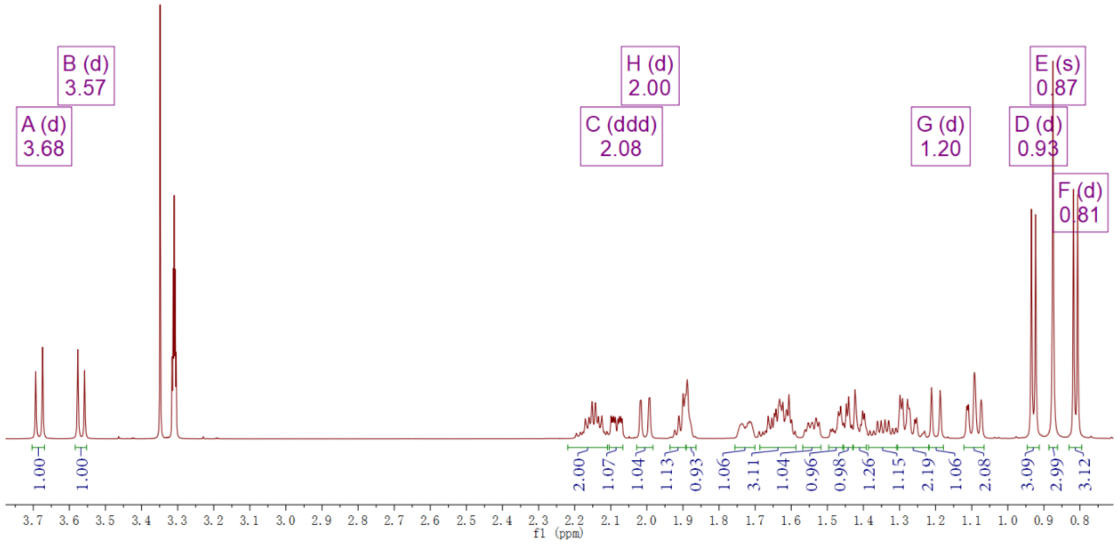


**Figure S71b.** Partial $^{1}H$ NMR spectrum ($\delta$ 0.7-3.8 ppm) of compound **15** in $CD_3OD$

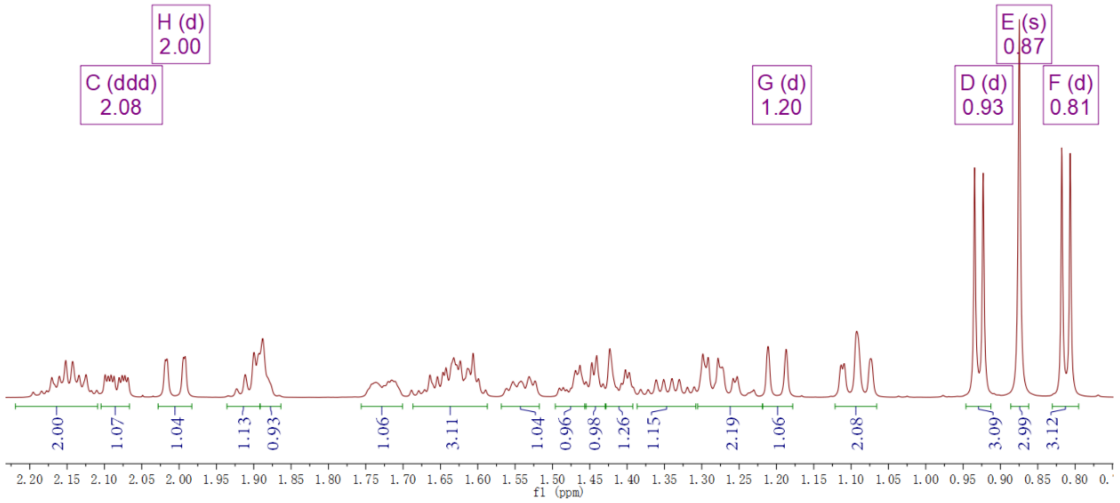


**Figure S71c.** Partial $^{1}$H NMR spectrum ($\delta$ 0.7-2.3 ppm) of compound **15** in $CD_3OD$

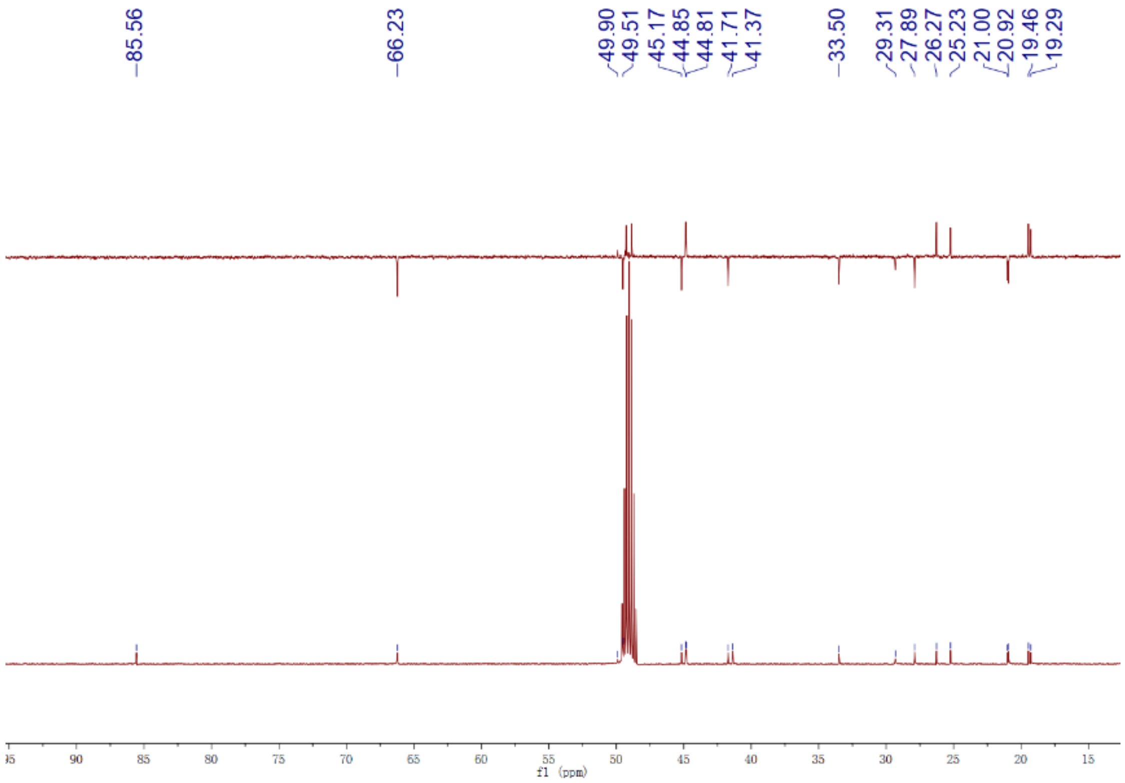


**Figure S72.** $^{13}$C NMR and DEPT 135 spectrum of compound **15** in $CD_3OD$

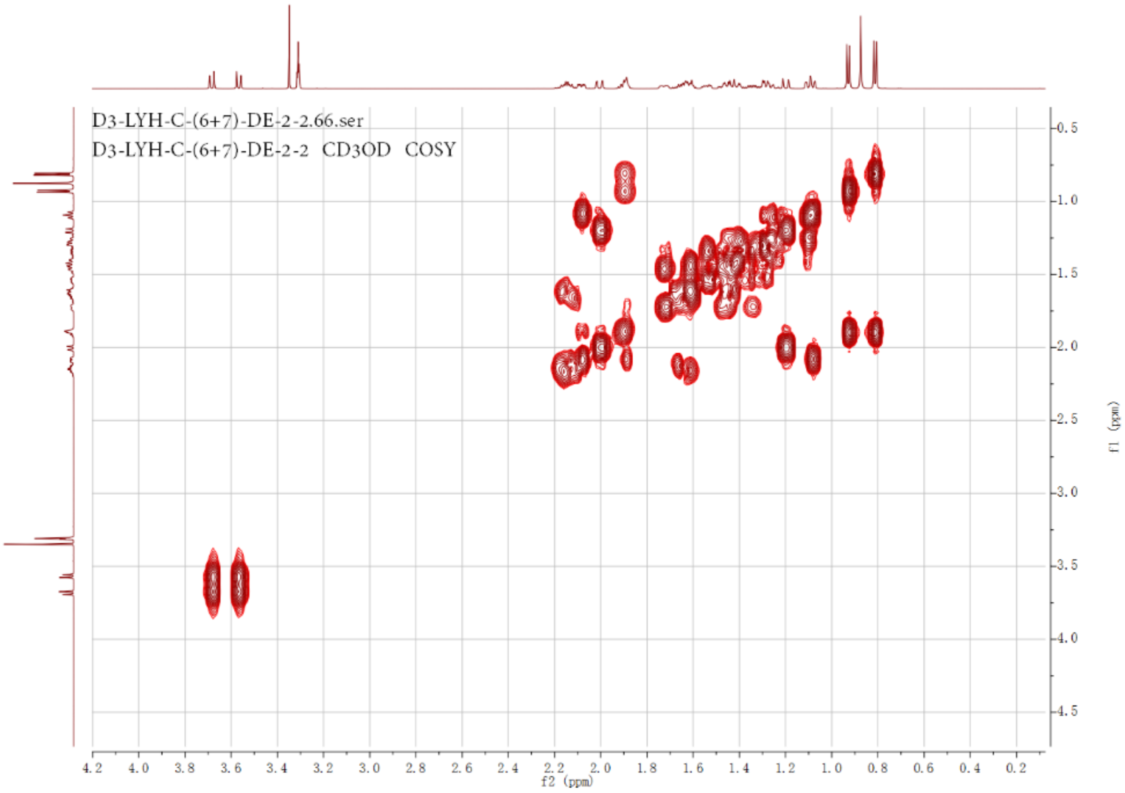


**Figure S73.** $^{1}H$-$^{1}H$ COSY spectrum of compound **15**in $CD_3OD$

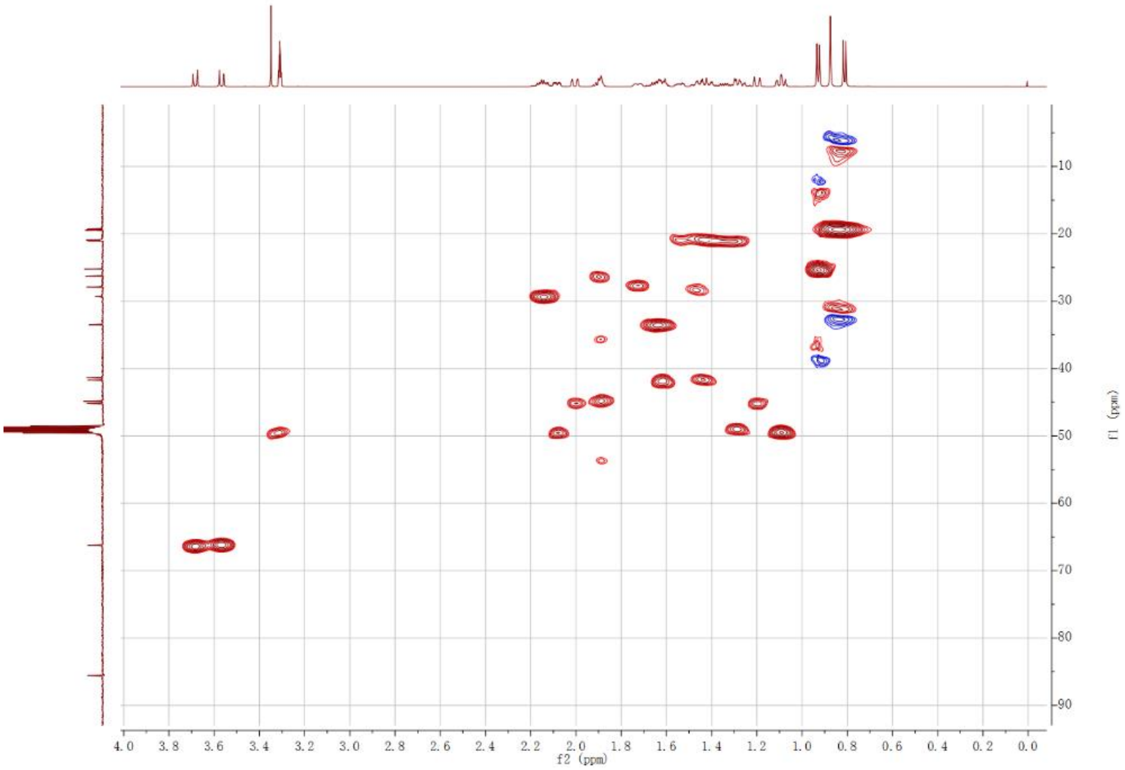


**Figure S74.** HSQC spectrum of compound **15** in $CD_3OD$

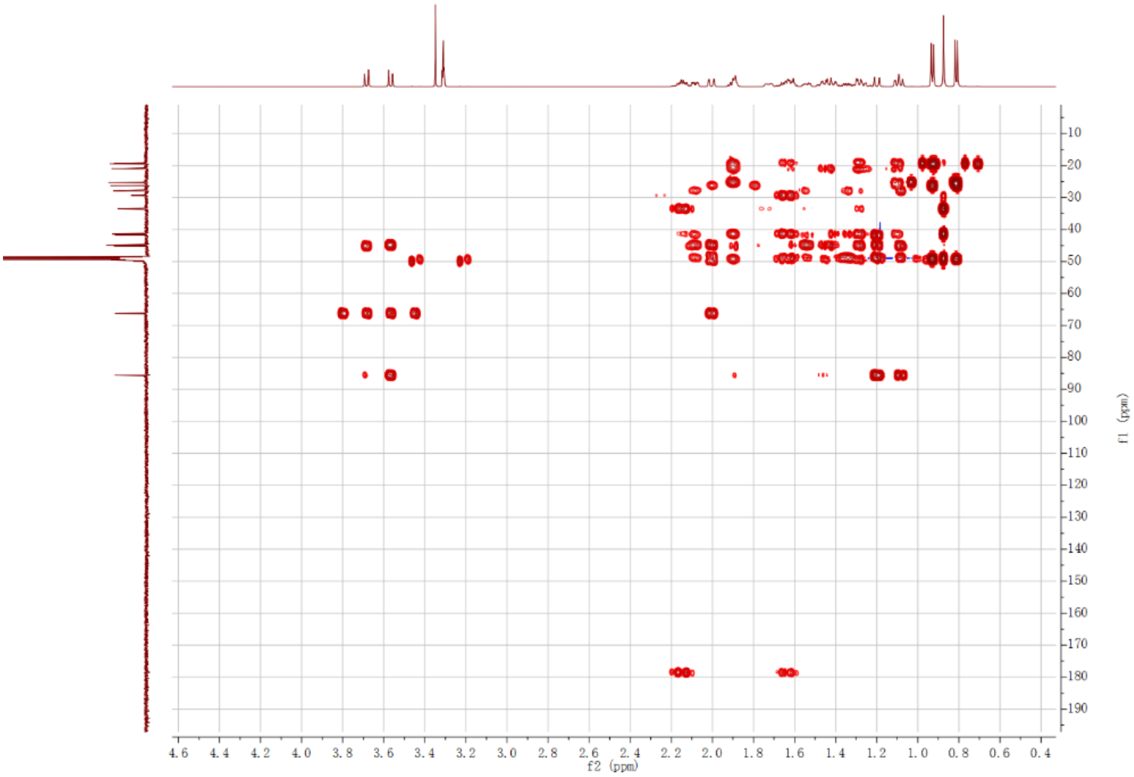


**Figure S75.** HMBC spectrum of compound **15** in $CD_3OD$

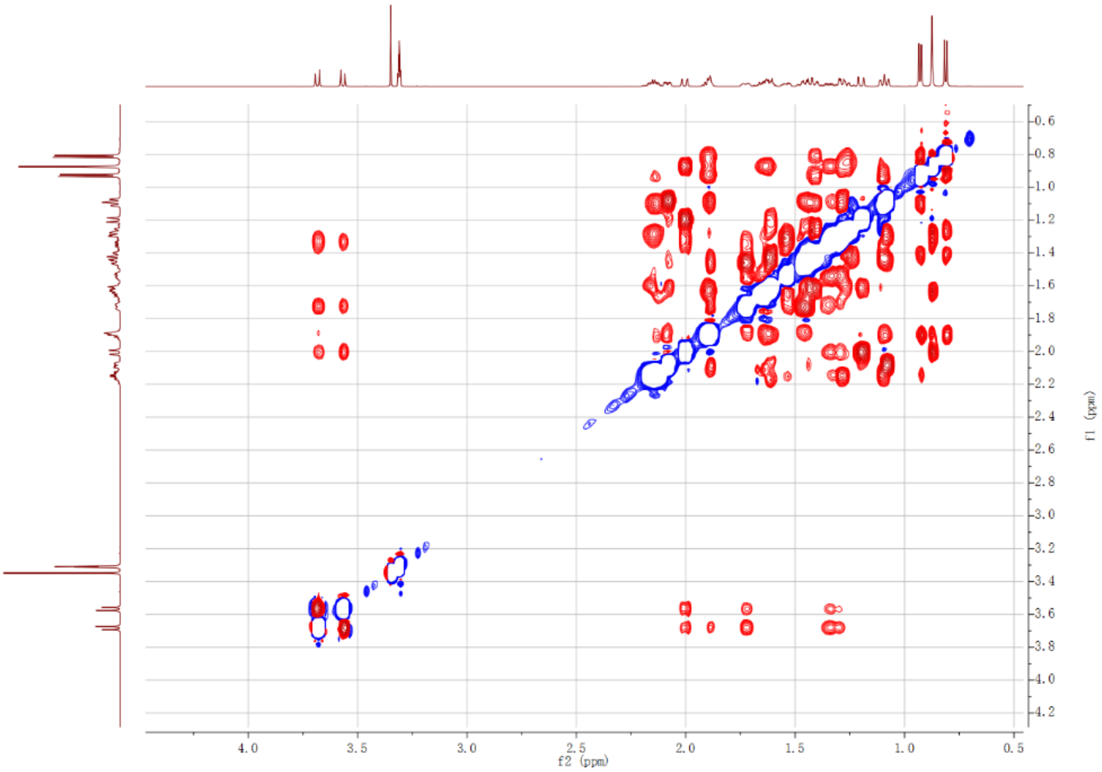


**Figure S76.** NOESY spectrum of compound **15** in $CD_3OD$

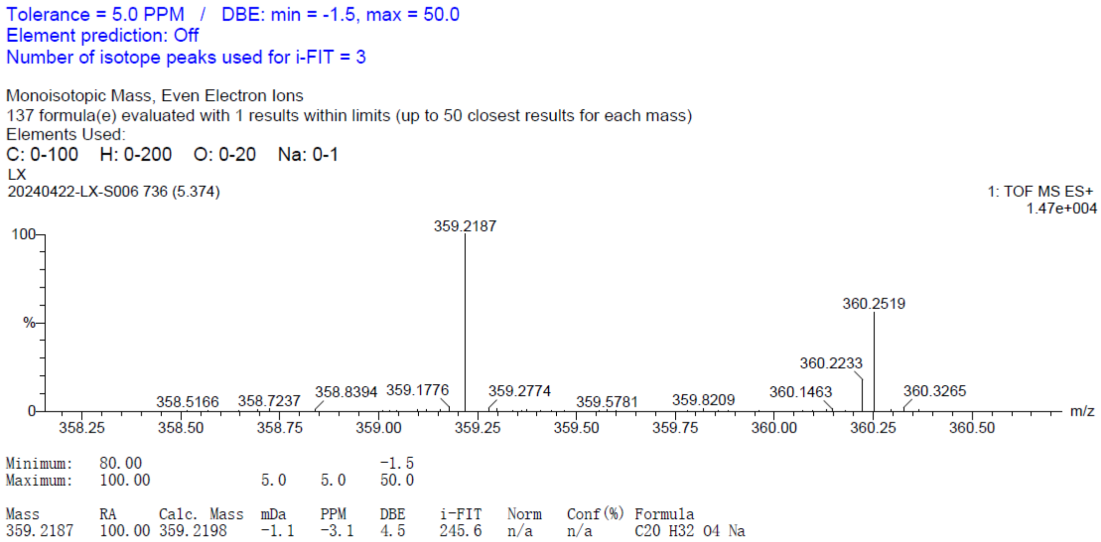


**Figure S77.** HRESIMS spectrum of compound **16**

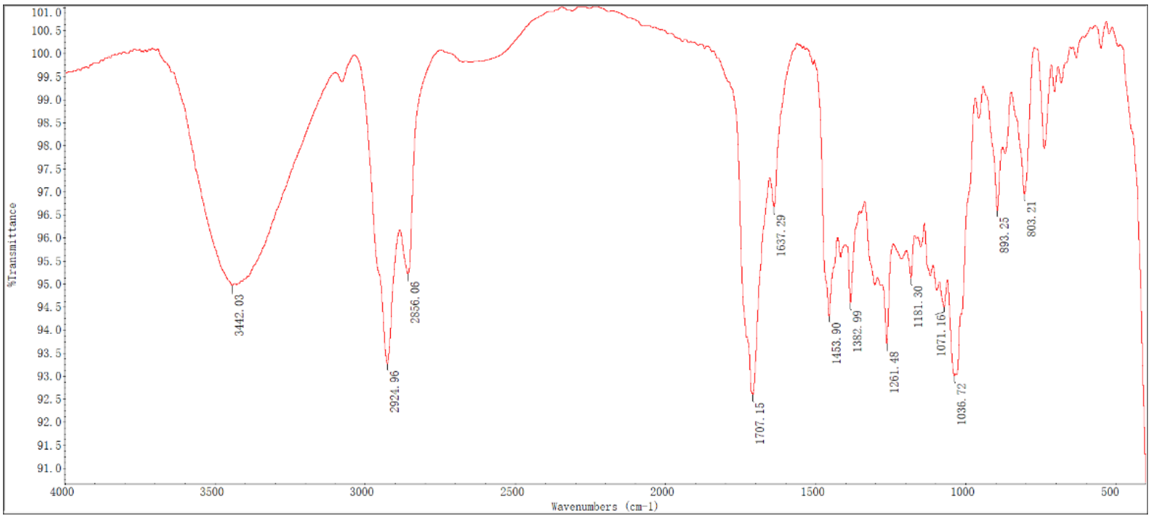


**Figure S78.** IR spectrum of compound **16**

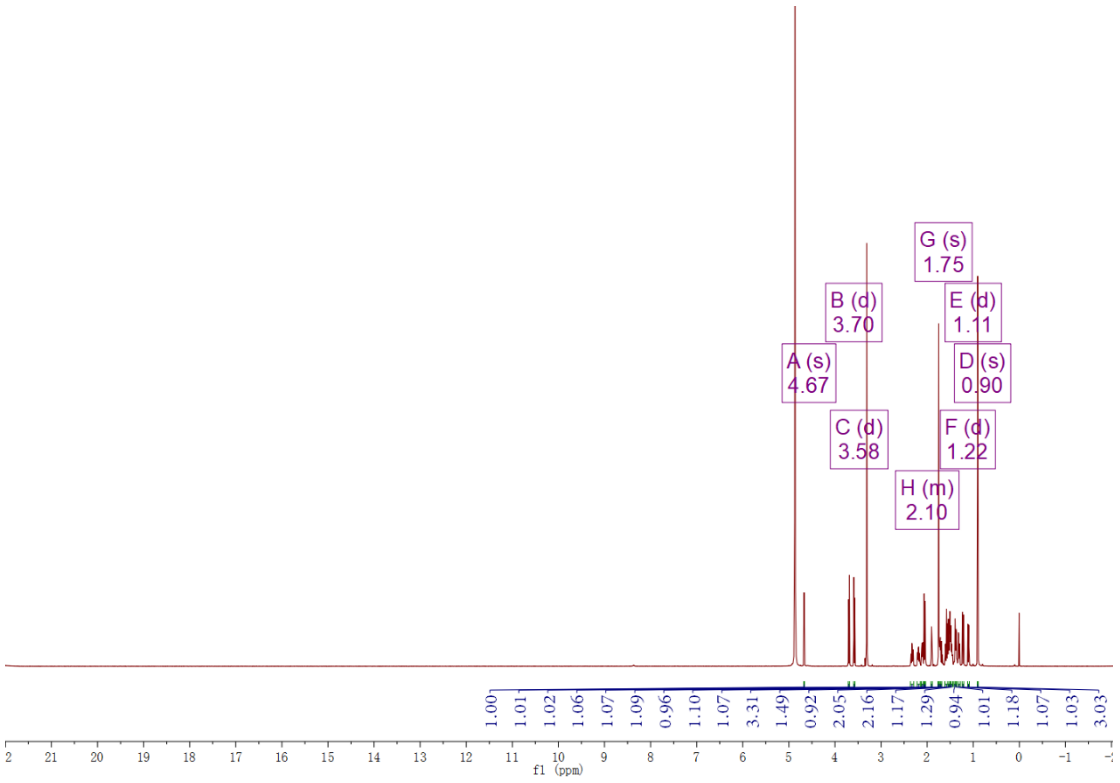


**Figure S79a.** $^{1}$H NMR spectrum of compound **16** in $CD_3OD$

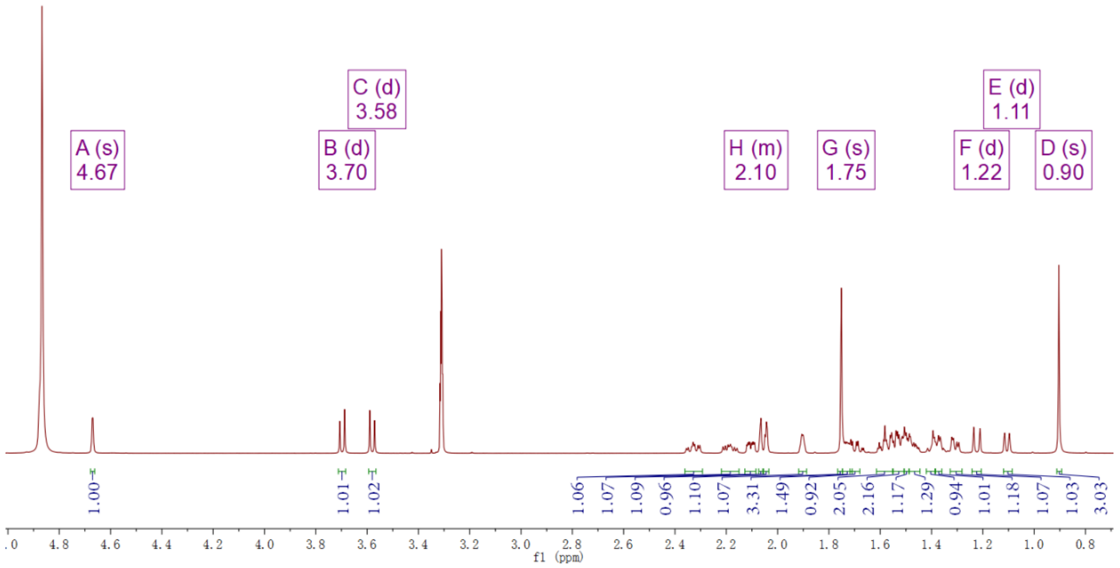


**Figure S79b.** Partial $^{1}$H NMR spectrum ($\delta$ 0.8-4.9 ppm) of compound **16** in $CD_3OD$

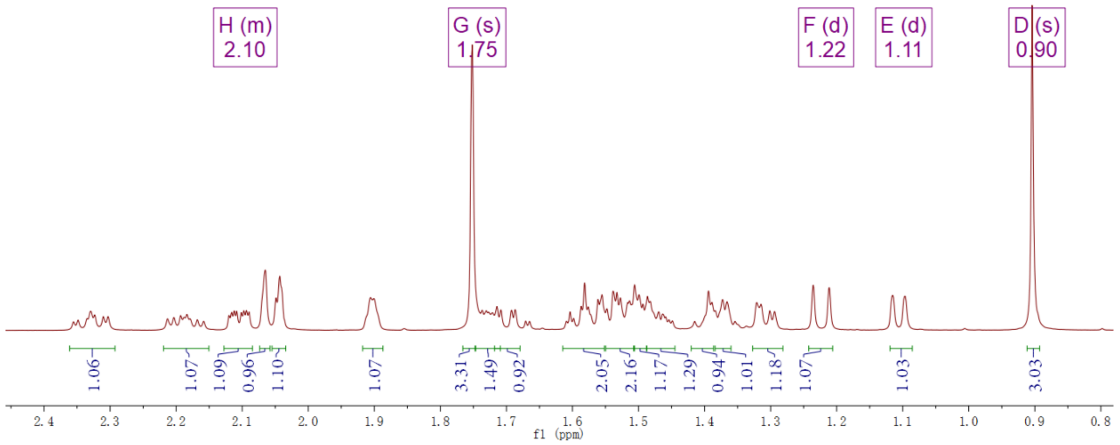


**Figure S79c.** Partial $^{1}$H NMR spectrum ($\delta$ 0.8-2.4 ppm) of compound **16** in $CD_3OD$

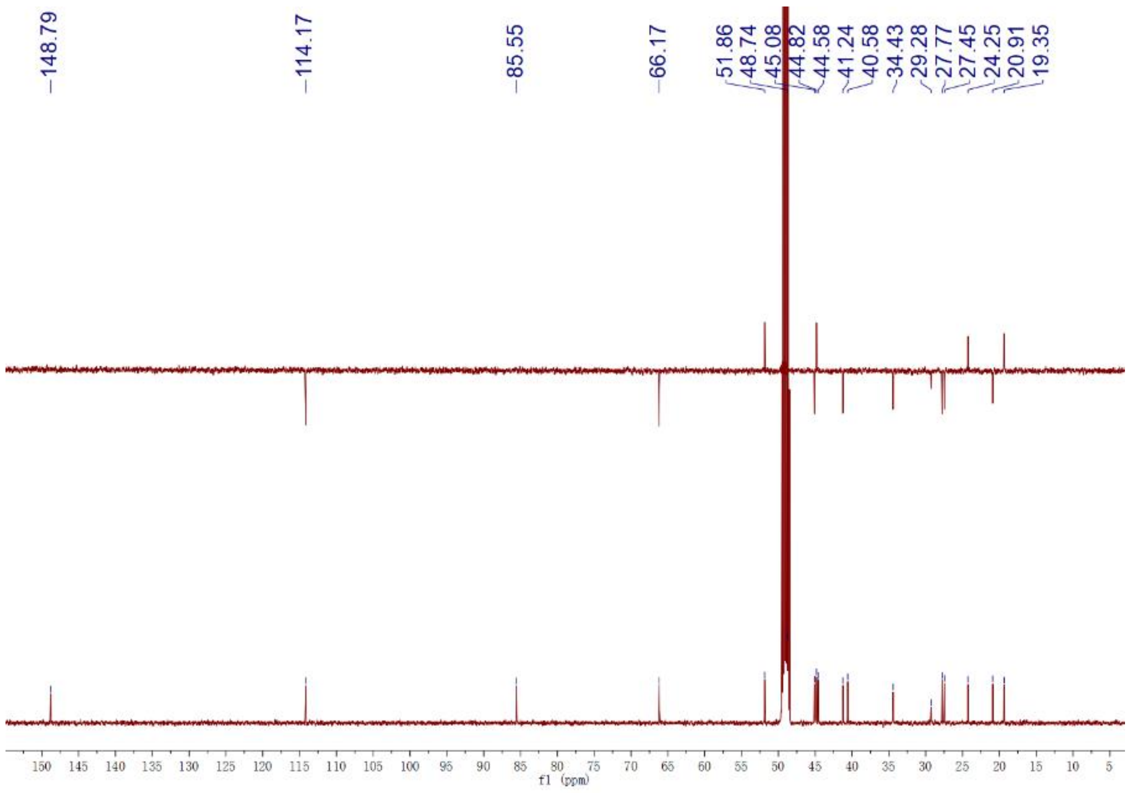


**Figure S80.** $^{13}$C NMR and DEPT 135 spectrum of compound **16** in $CD_3OD$

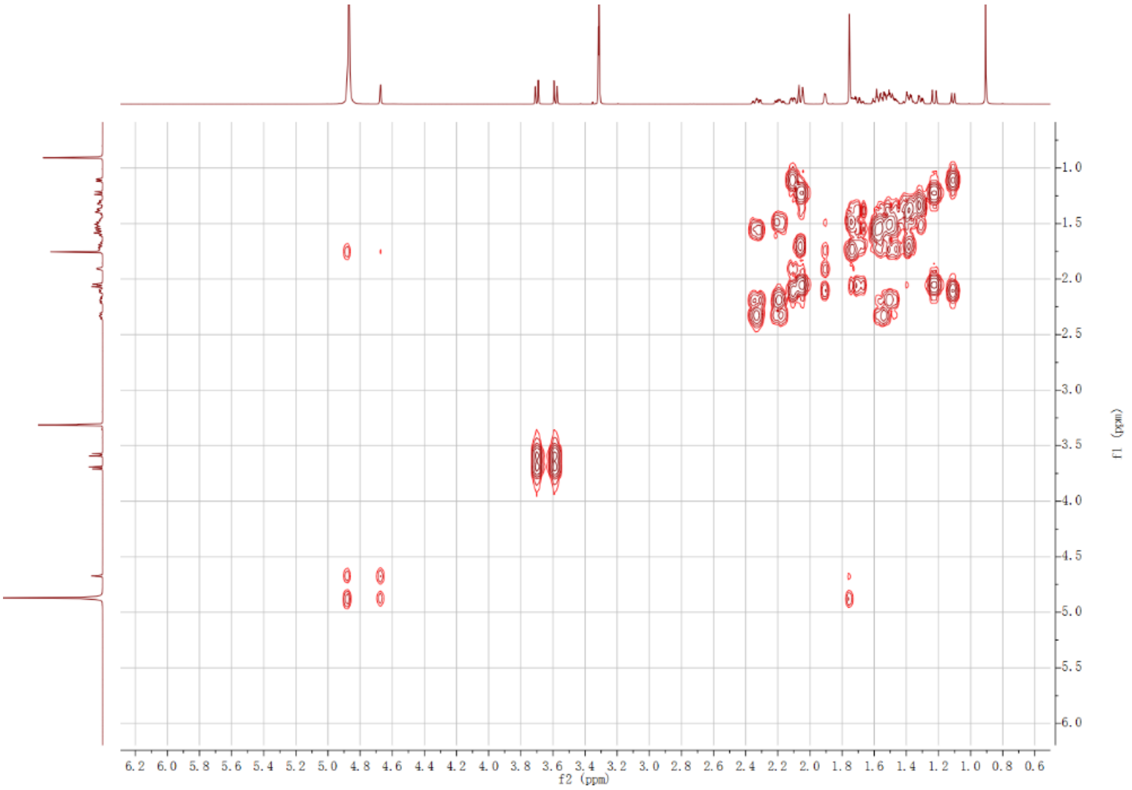


**Figure S81.** $^{1}H$-$^{1}H$ COSY spectrum of compound **16** in $CD_3OD$

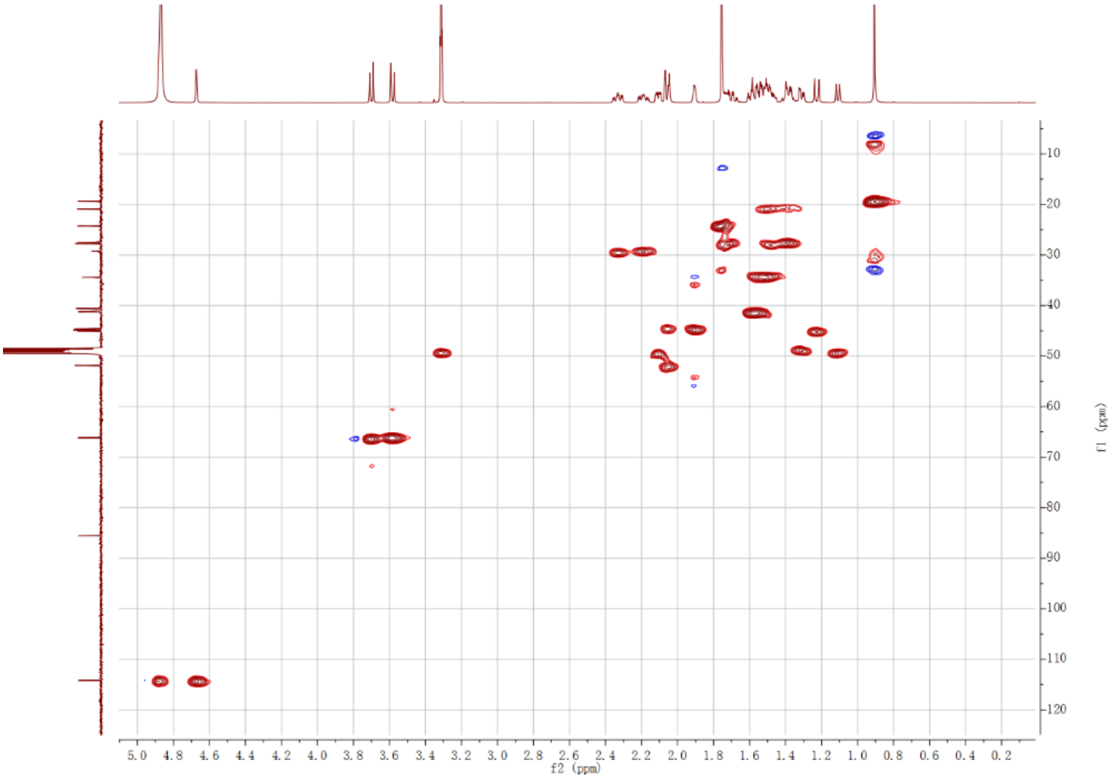


**Figure S82.** HSQC spectrum of compound **16** in $CD_3OD$

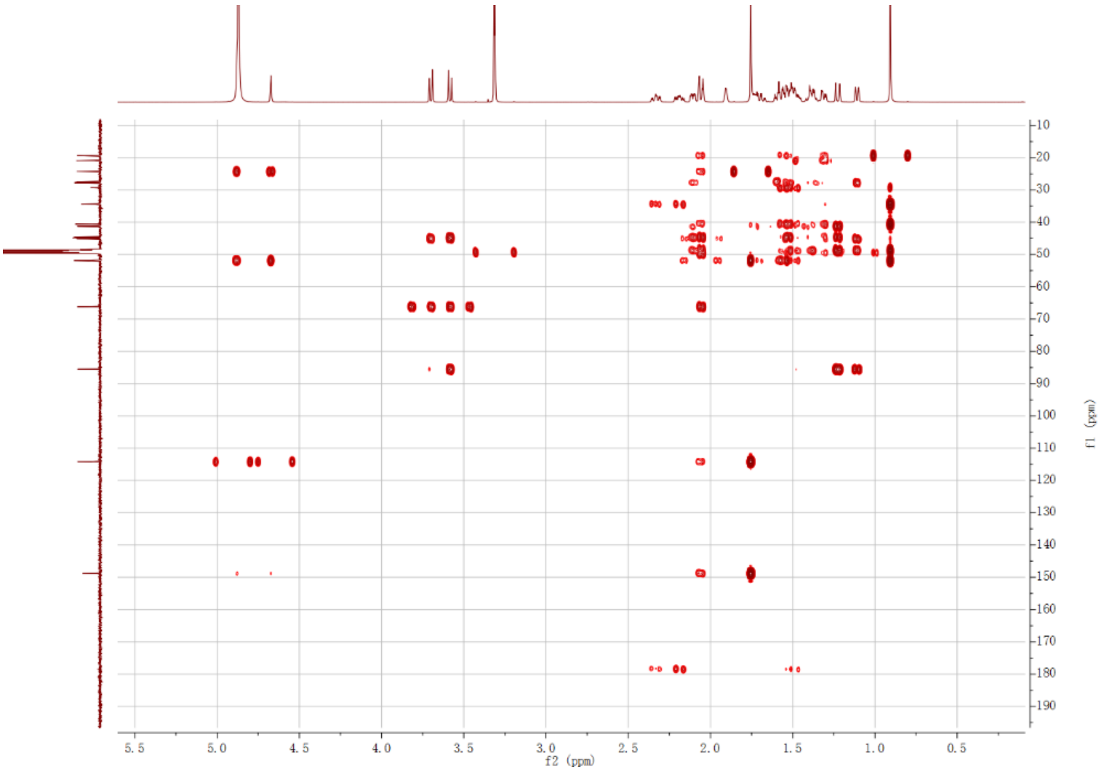


**Figure S83.** HMBC spectrum of compound **16** in $CD_3OD$

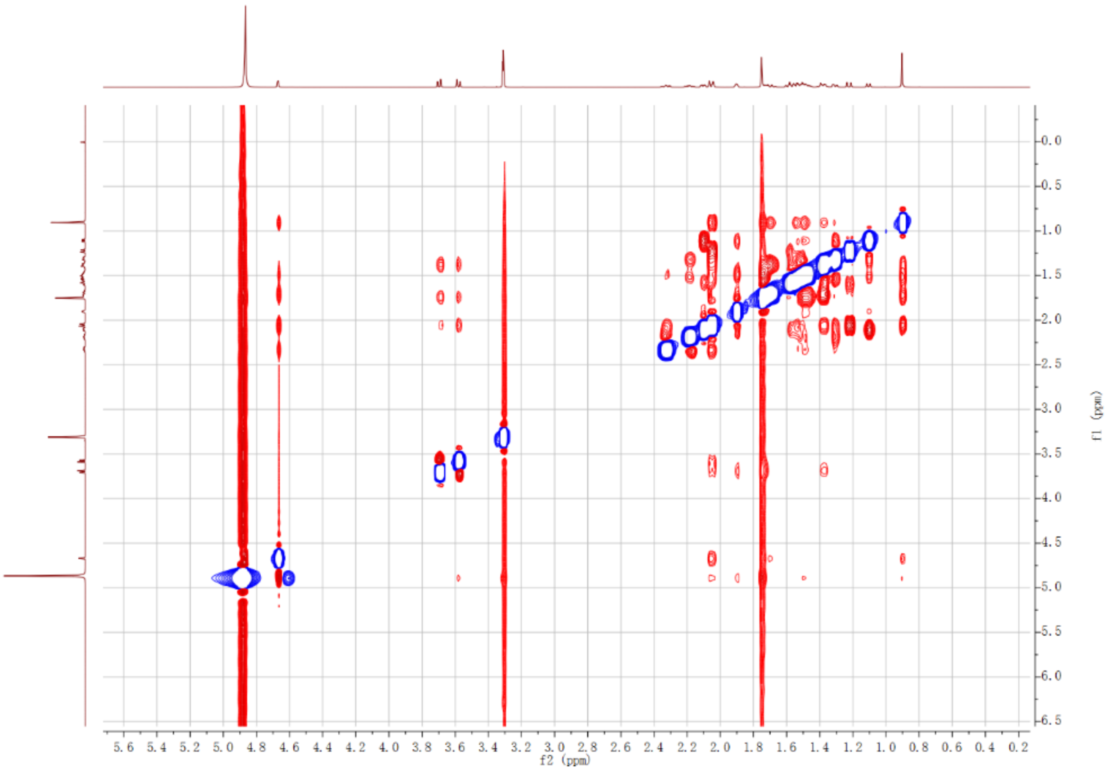


**Figure S84.** NOESY spectrum of compound **16** in $CD_3OD$

Tolerance = 5.0 PPM / DBE: min = -1.5, max = 50.0
Element prediction: Off
Number of isotope peaks used for i-FIT = 3

Monoisotopic Mass, Even Electron Ions
151 formula(e) evaluated with 1 results within limits (up to 50 closest results for each mass)
Elements Used:
C: 0-100 H: 0-200 O: 0-20 Na: 0-1
LX
20240422-LX-S010 853 (6.230) Cm (851:853)

1: TOF MS ES+
3.31e+004

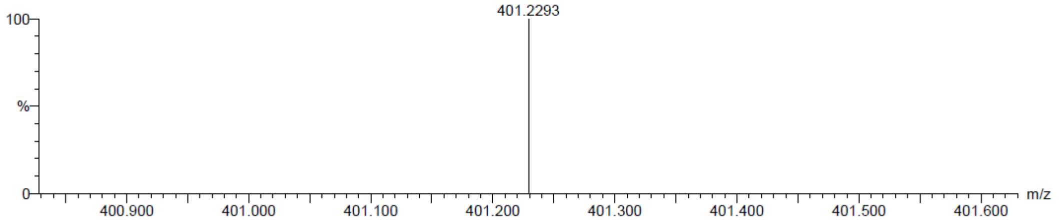


| | | | | | | | | |
|---|---|---|---|---|---|---|---|---|
| Minimum: | | | | -1.5 | | | | |
| Maximum: | | 5.0 | 5.0 | 50.0 | | | | |
| Mass | Calc. Mass | mDa | PPM | DBE | i-FIT | Norm | Conf(%) | Formula |
| 401.2293 | 401.2304 | -1.1 | -2.7 | 5.5 | 33.6 | n/a | n/a | C22 H34 O5 Na |

**Figure S85.** HRESIMS spectrum of compound **17**

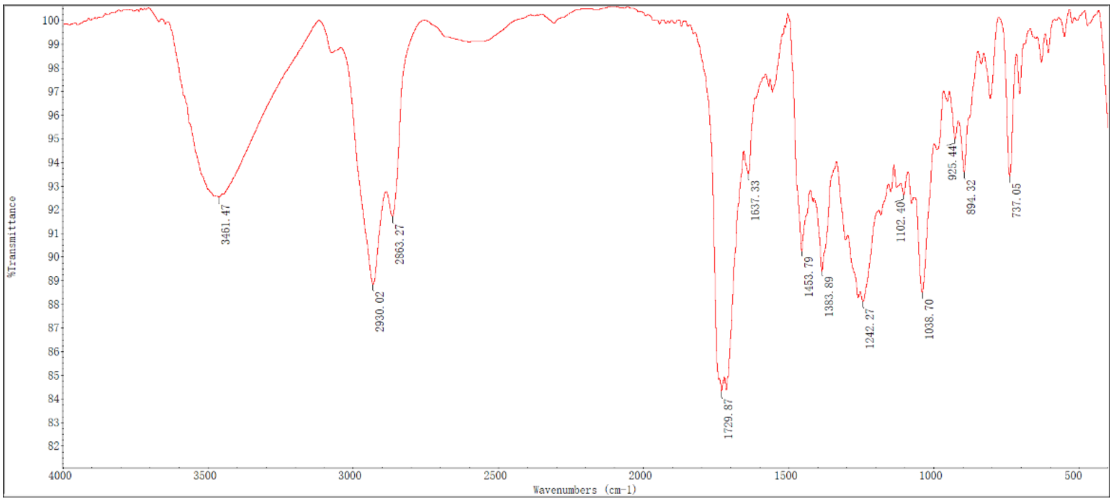


**Figure S86.** IR spectrum of compound **17**

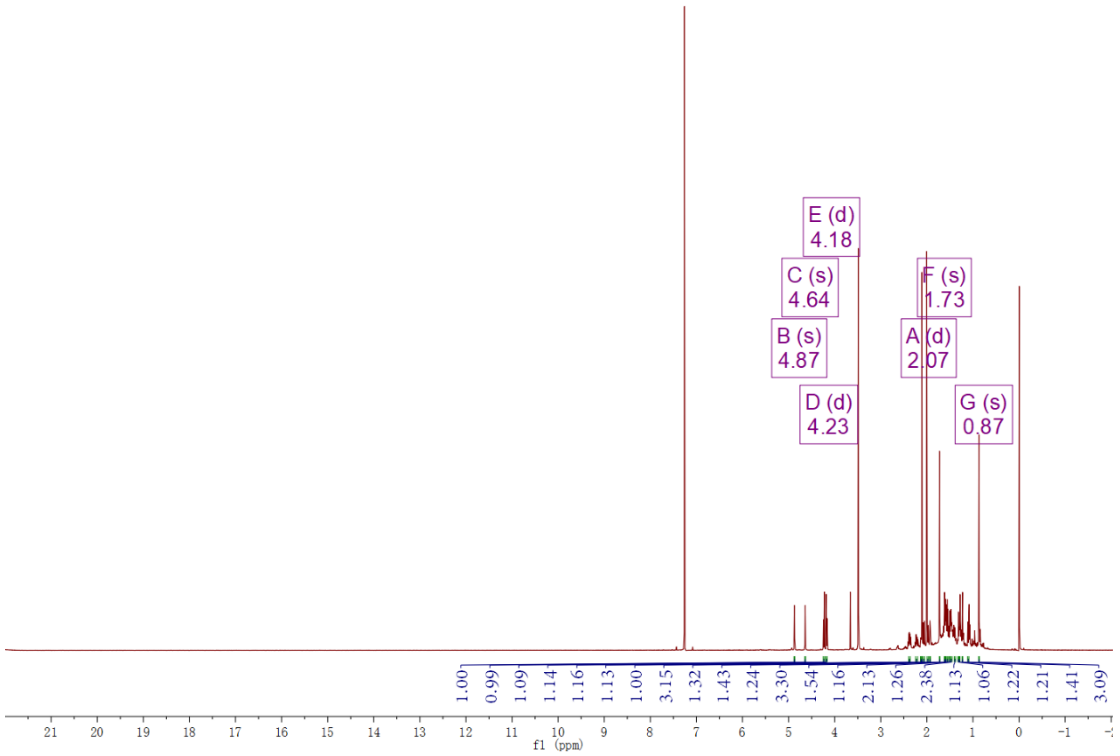


**Figure S87a.** $^{1}H$ NMR spectrum ($\delta$ 0.7-4.3 ppm)of compound **17** in $CDCl_3$

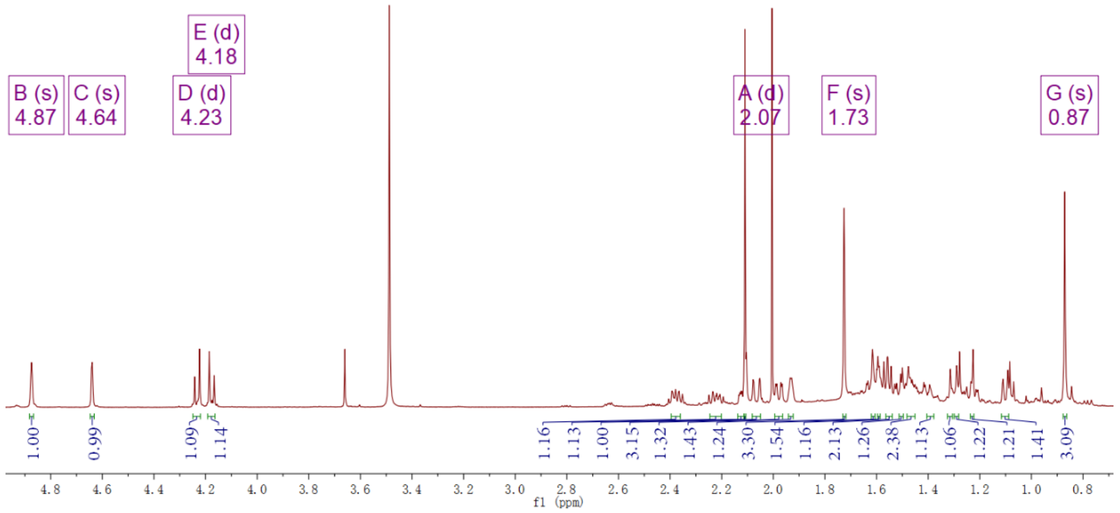


**Figure S87b.** Partial $^{1}H$ NMR spectrum ($\delta$ 0.7-4.9 ppm)of compound **17** in $CDCl_3$

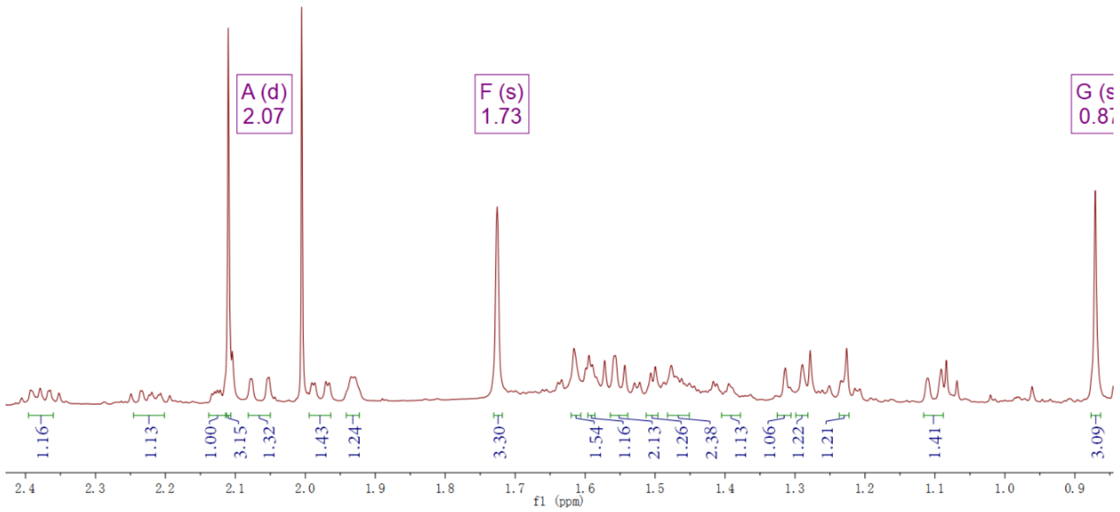


**Figure S87b.** Partial $^{1}H$ NMR spectrum ($\delta$ 0.8-2.5 ppm) of compound **17** in $CDCl_3$

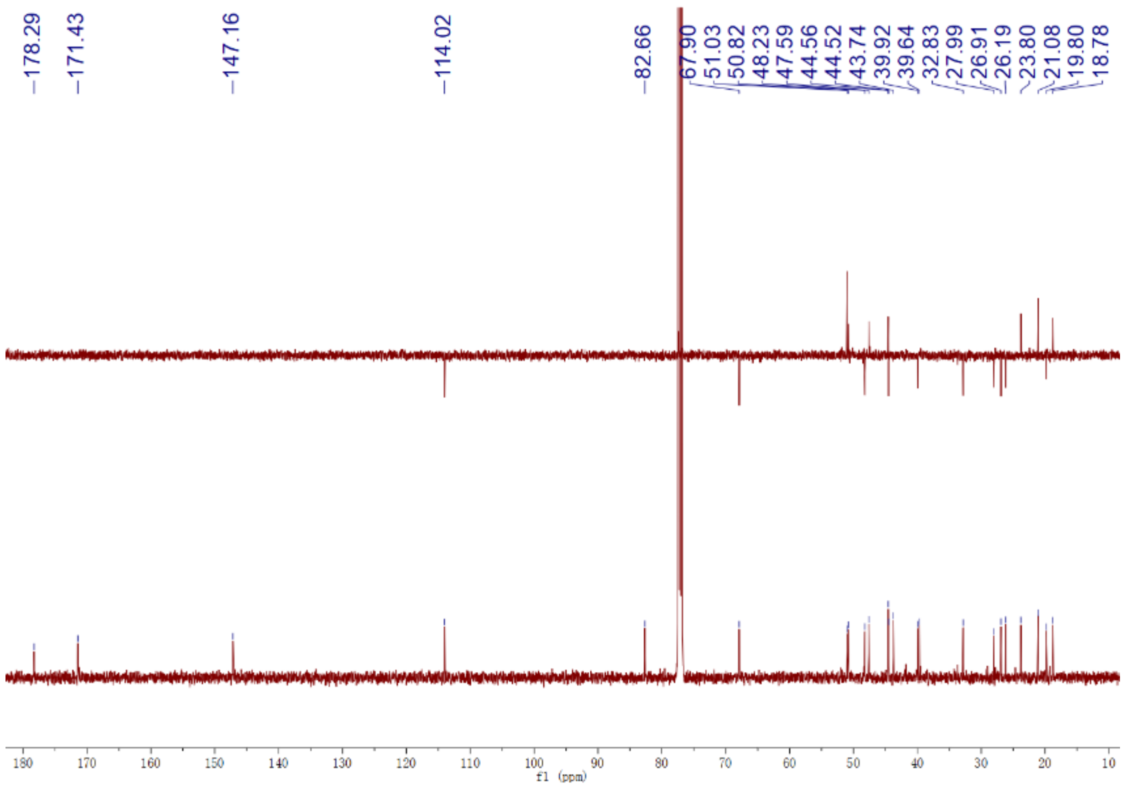


**Figure S88.** $^{13}C$ NMR and DEPT 135 spectrum of compound **17** in $CDCl_3$

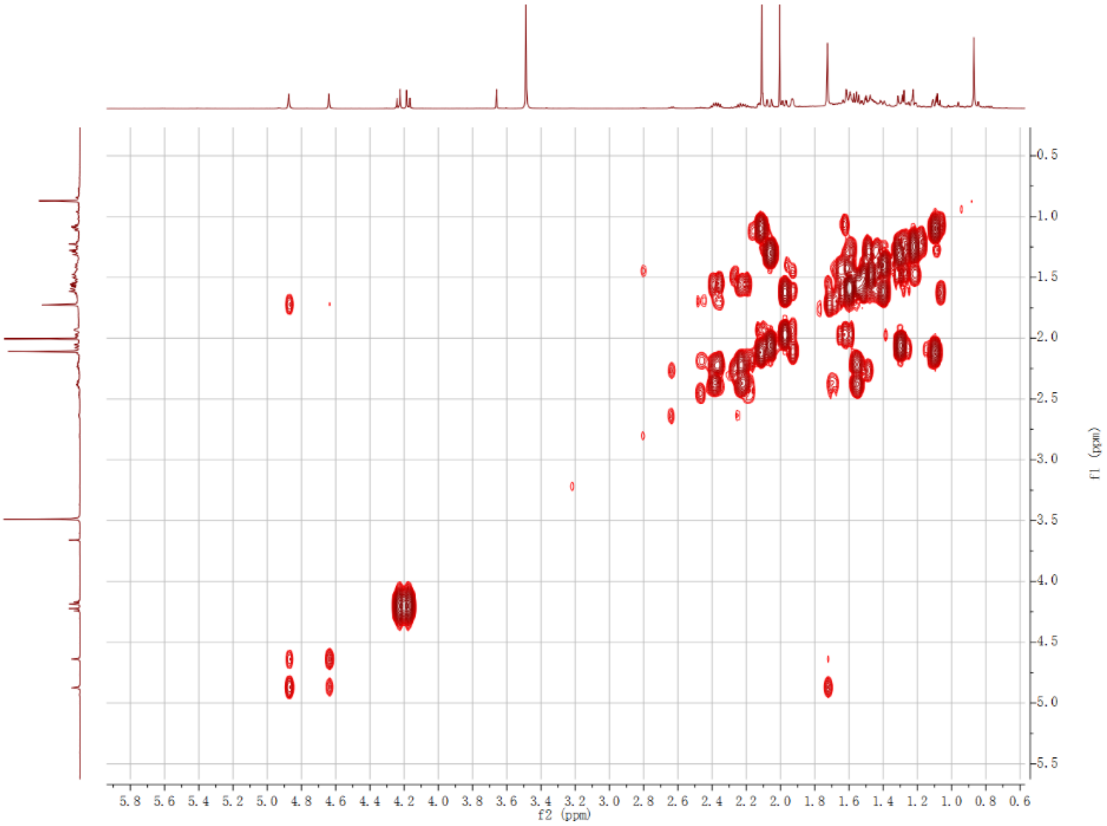


**Figure S89.** $^{1}H$-$^{1}H$ COSY spectrum of compound **17** in $CDCl_3$

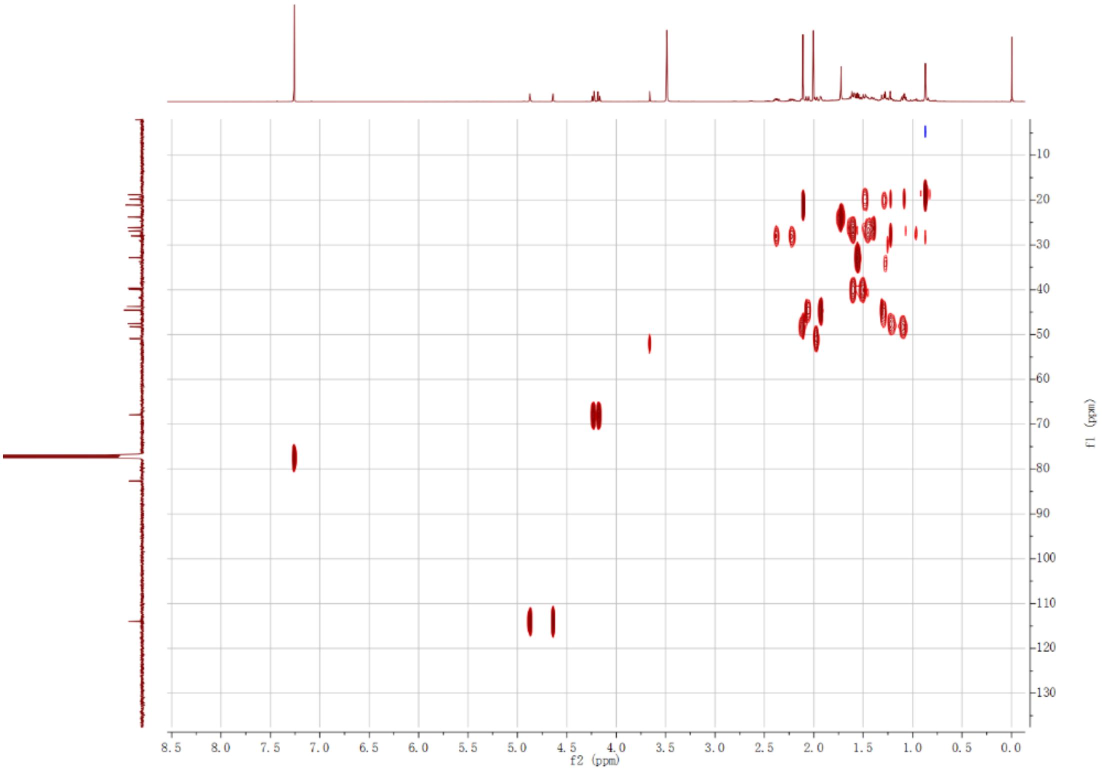


**Figure S90.** HSQC spectrum of compound **17** in $CDCl_3$

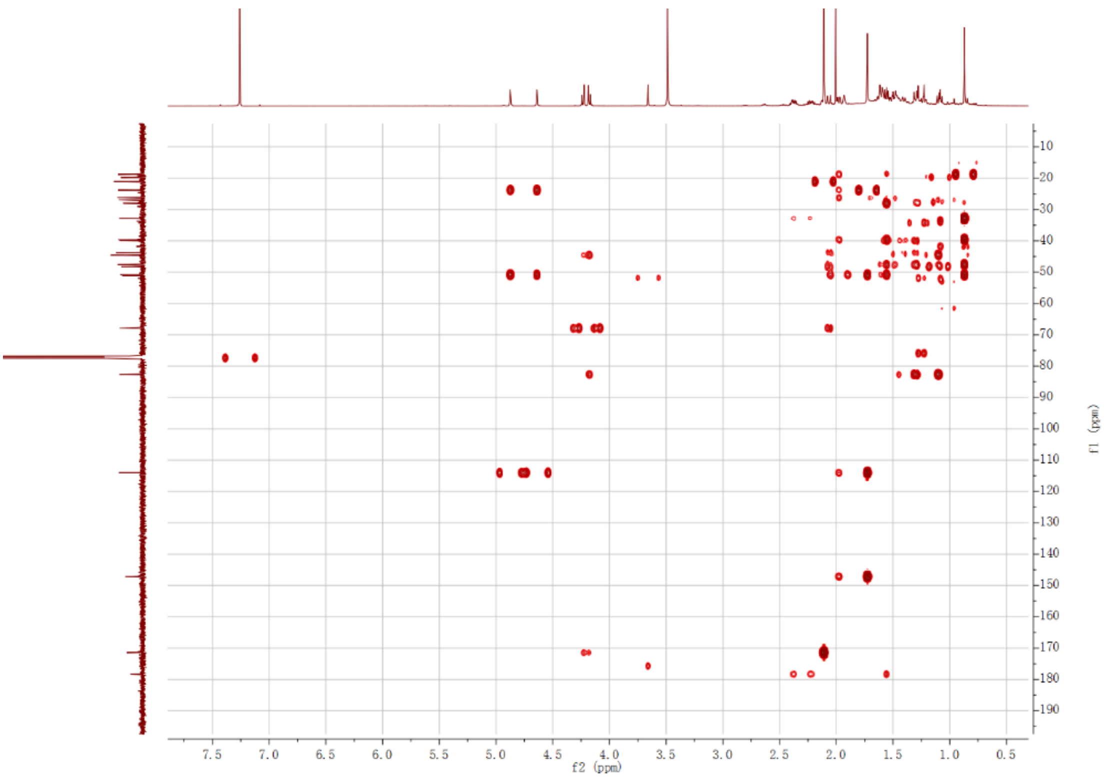


**Figure S91.** HMBC spectrum of compound **17** in $CDCl_3$

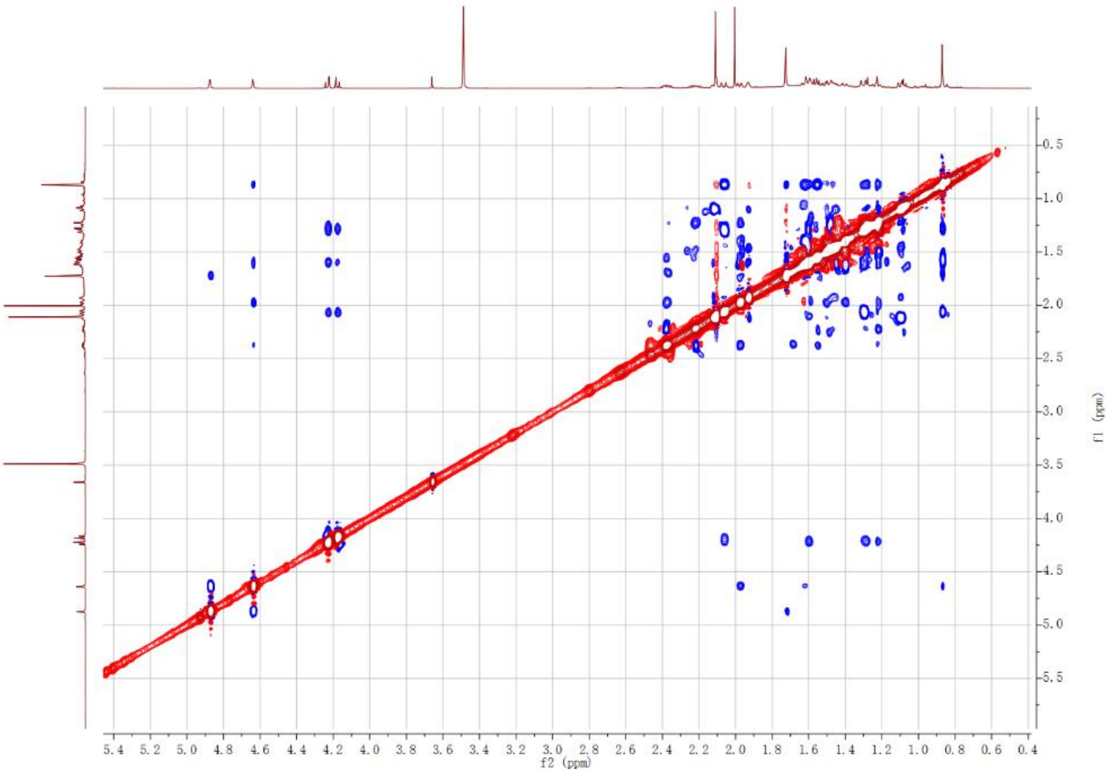


**Figure S92.** NOESY spectrum of compound **17** in $CDCl_3$

Tolerance = 5.0 PPM / DBE: min = -1.5, max = 50.0
Element prediction: Off
Number of isotope peaks used for i-FIT = 3

Monoisotopic Mass, Even Electron Ions
151 formula(e) evaluated with 1 results within limits (up to 50 closest results for each mass)
Elements Used:
C: 0-100 H: 0-200 O: 0-20 Na: 0-1
LX
20240422-LX-S007 1012 (7.384)

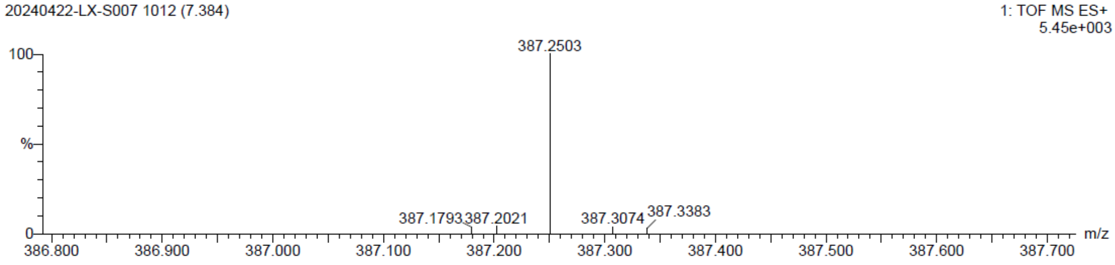


| | | | | | | | | | |
|---|---|---|---|---|---|---|---|---|---|
| Minimum: | 80.00 | | | | -1.5 | | | | |
| Maximum: | 100.00 | | 5.0 | 5.0 | 50.0 | | | | |
| Mass | RA | Calc. Mass | mDa | PPM | DBE | i-FIT | Norm | Conf(%) | Formula |
| 387.2503 | 100.00 | 387.2511 | -0.8 | -2.1 | 4.5 | 49.3 | n/a | n/a | C22 H36 O4 Na |

**Figure S93.** HRESIMS spectrum of compound **18**

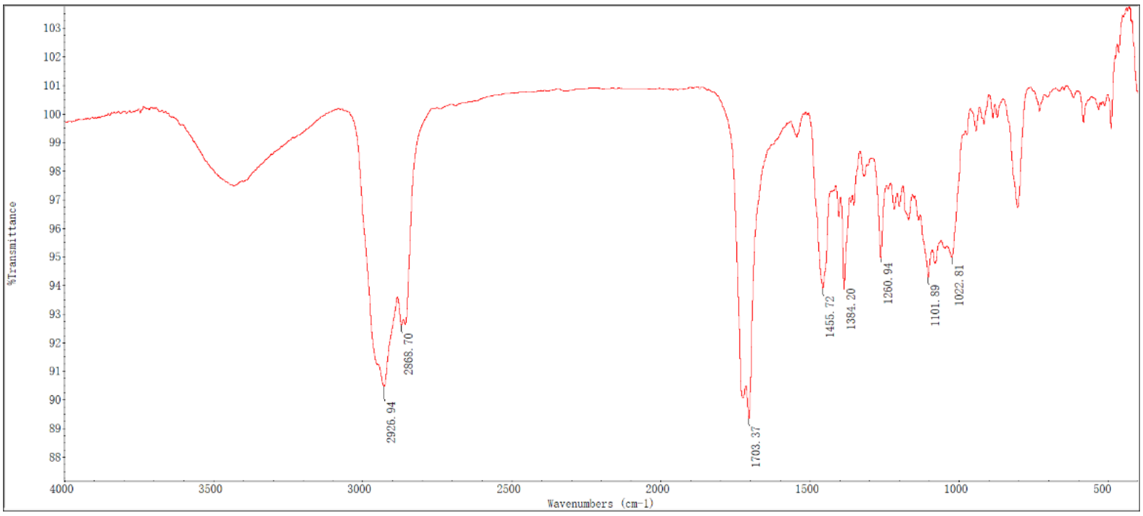


**Figure S94.** IR spectrum of compound **18**

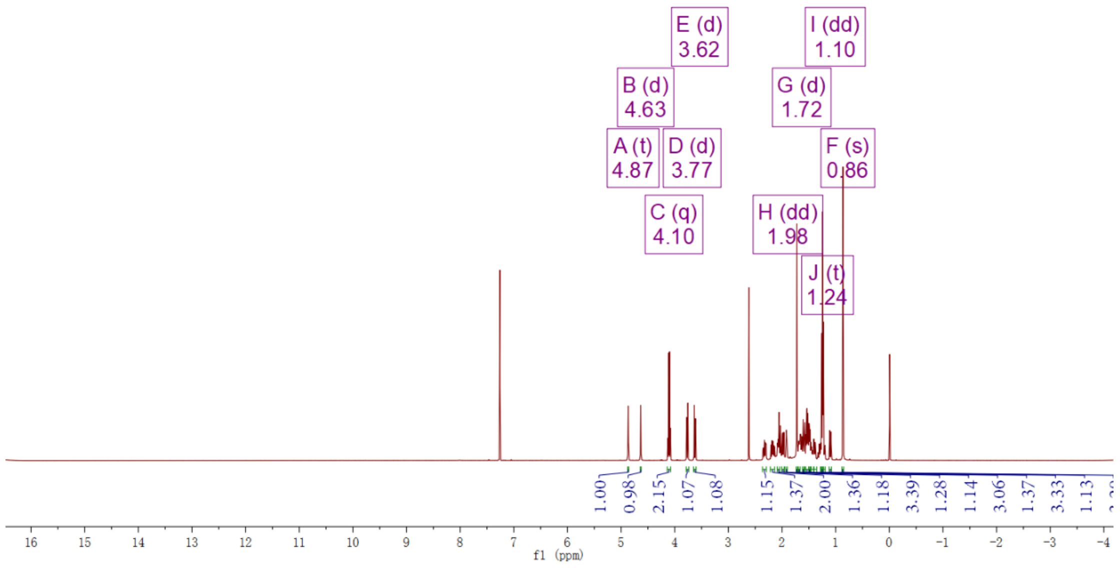


**Figure S95a.** $^1$H NMR spectrum of compound **18** in $CDCl_3$

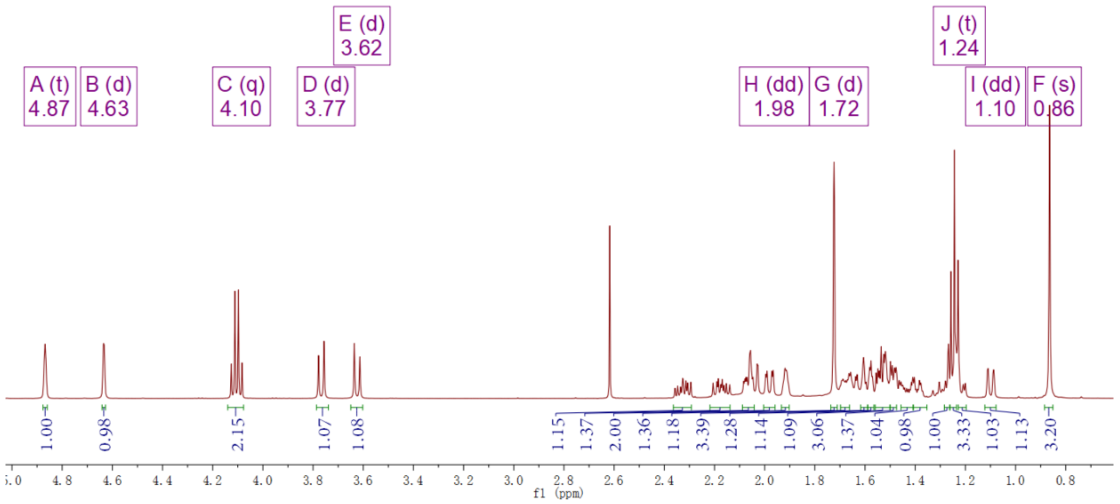


**Figure S95b.** Partial $^1$H NMR spectrum ($\delta$ 0.7-5.0 ppm) of compound **18** in $CDCl_3$

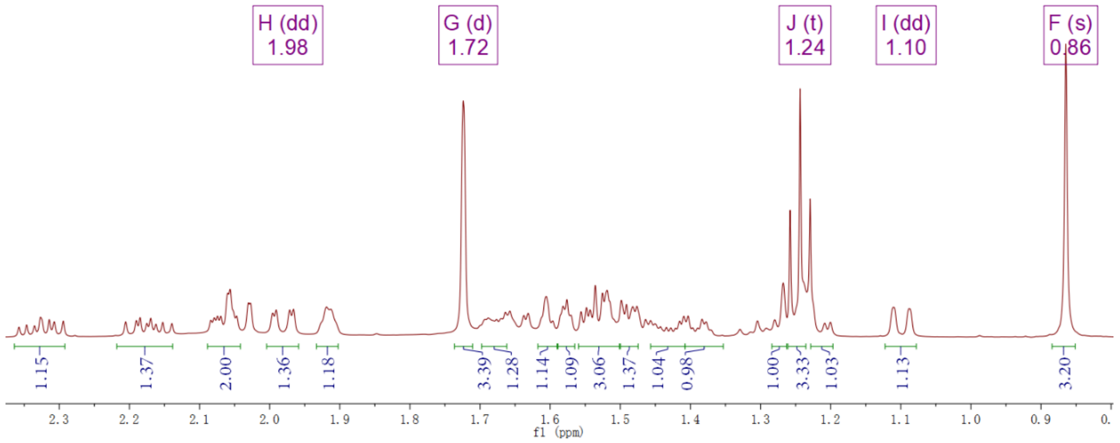


**Figure S95c.** Partial $^{1}H$ NMR spectrum ($\delta$ 0.8-2.4 ppm) of compound **18** in $CDCl_3$

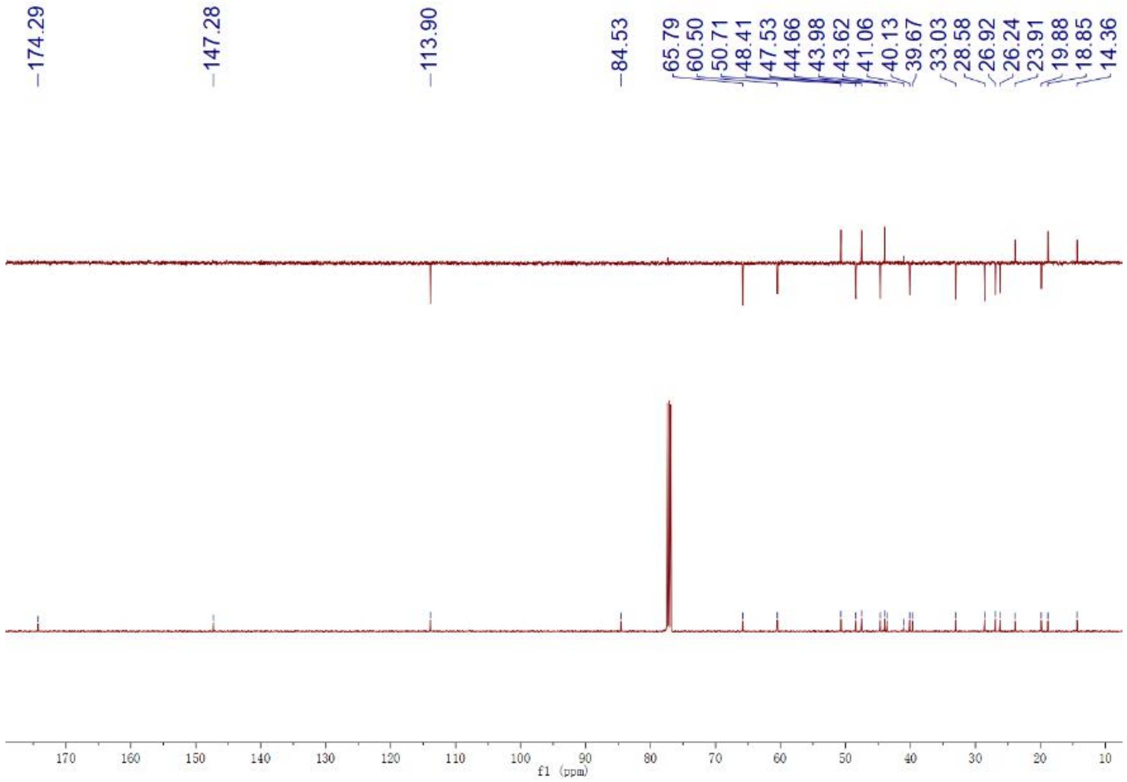


**Figure S96.** $^{13}C$ NMR and DEPT 135 spectrum of compound **18** in $CDCl_3$

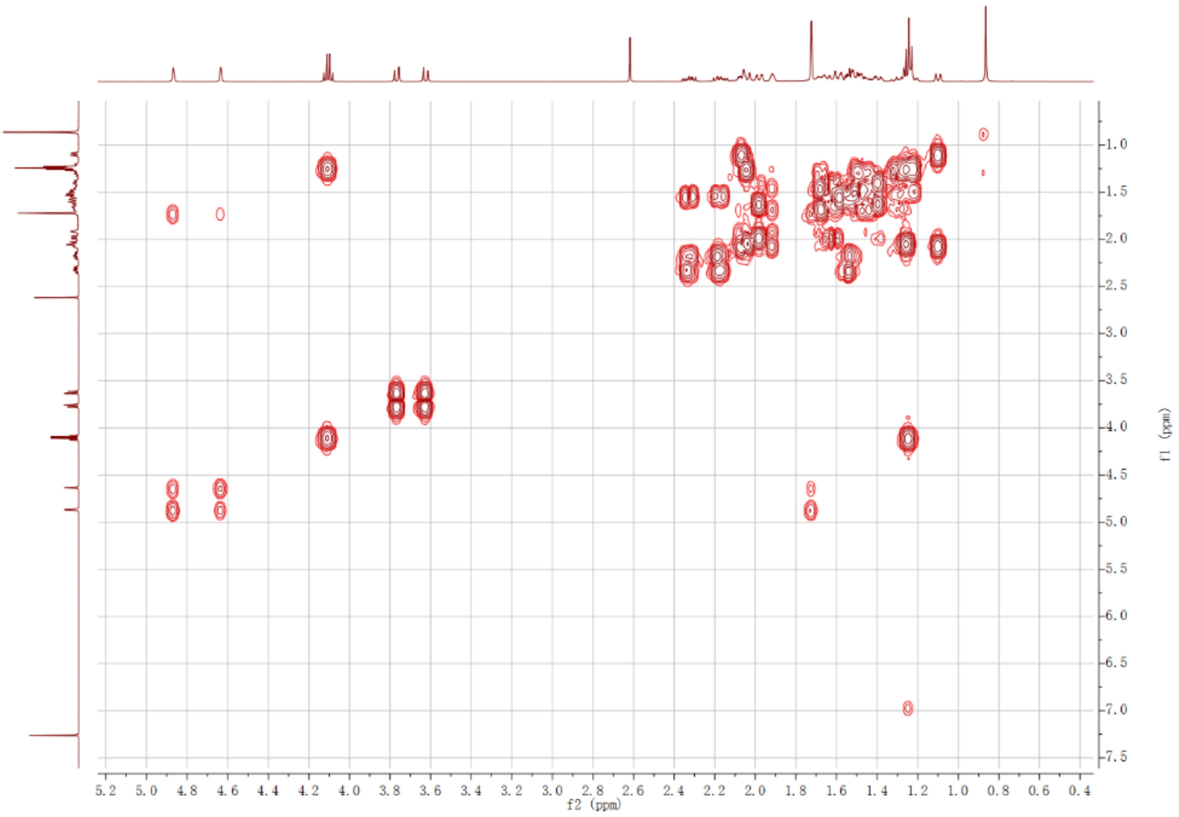


**Figure S97.** $^{1}H$-$^{1}H$ COSY spectrum of compound **18** in $CDCl_3$

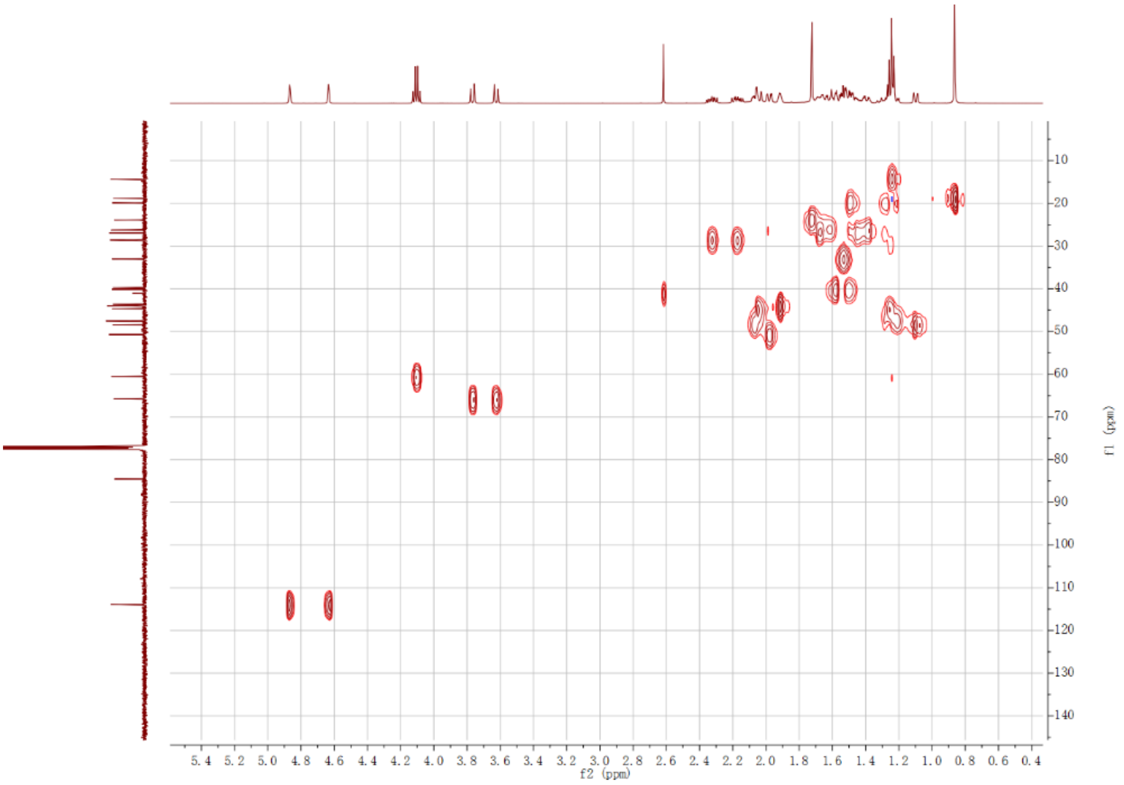


**Figure S98.** HSQC spectrum of compound **18** in $CDCl_3$

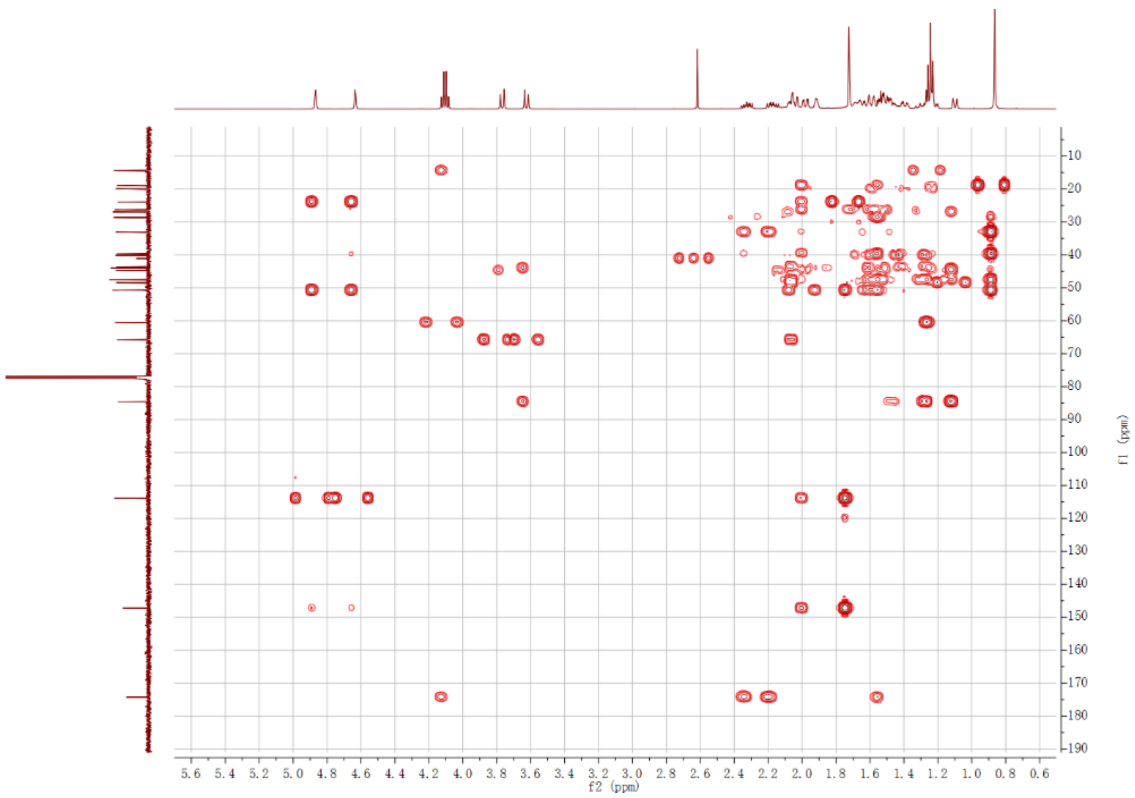


**Figure S99.** HMBC spectrum of compound **18** in $CDCl_3$

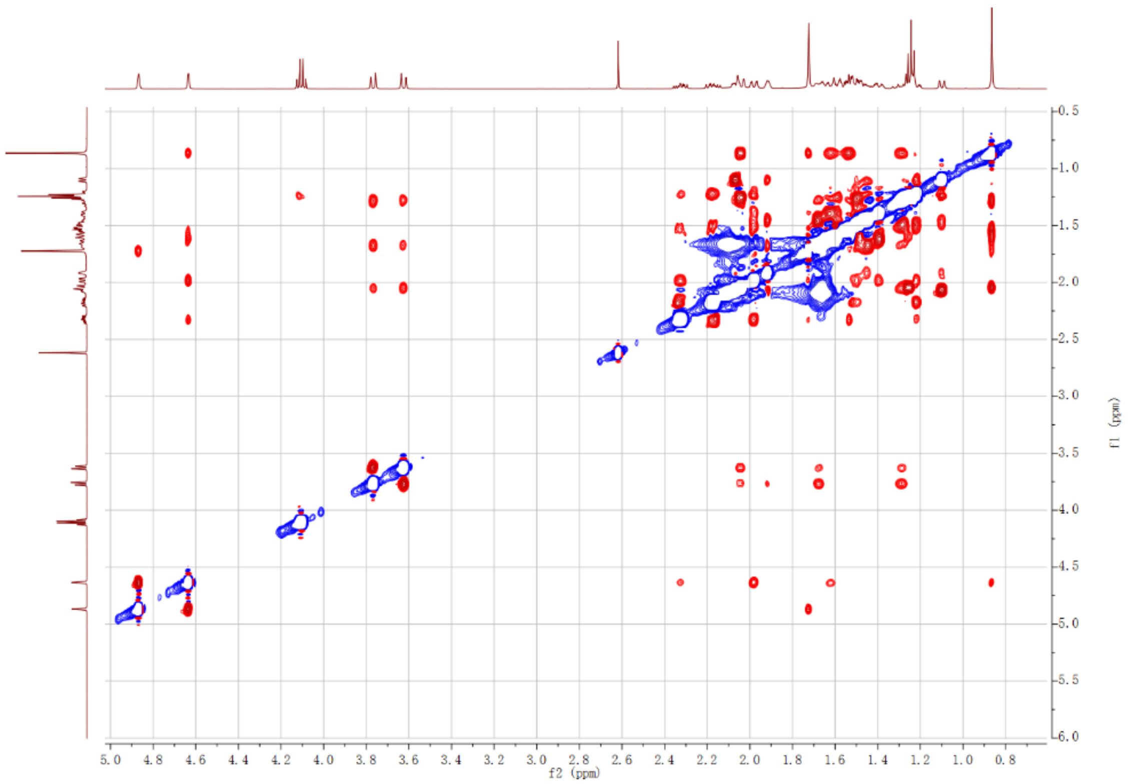


**Figure S100.** NOESY spectrum of compound **18** in $CDCl_3$

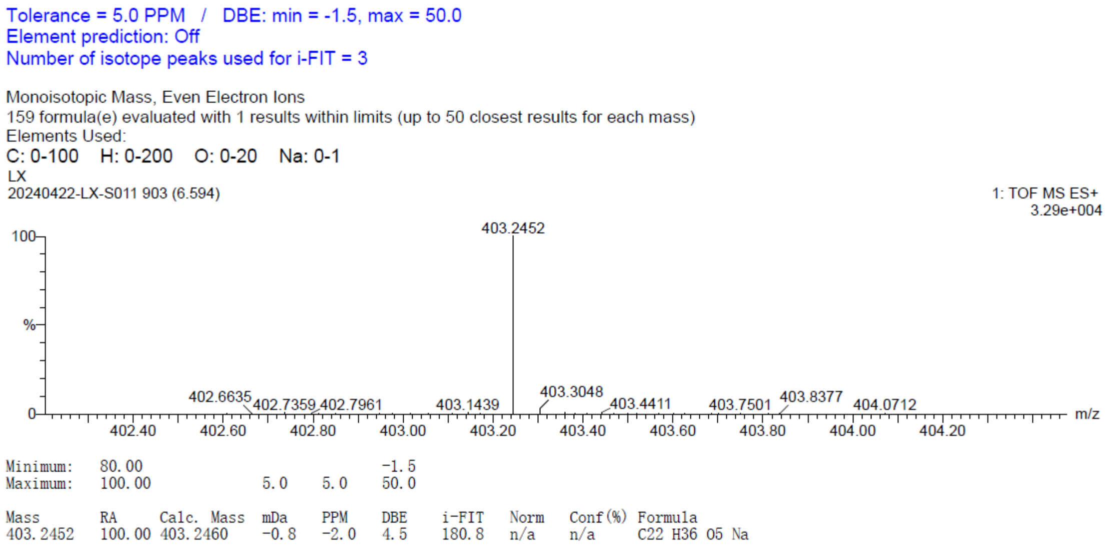


**Figure S101.** HRESIMS spectrum of compound **19**

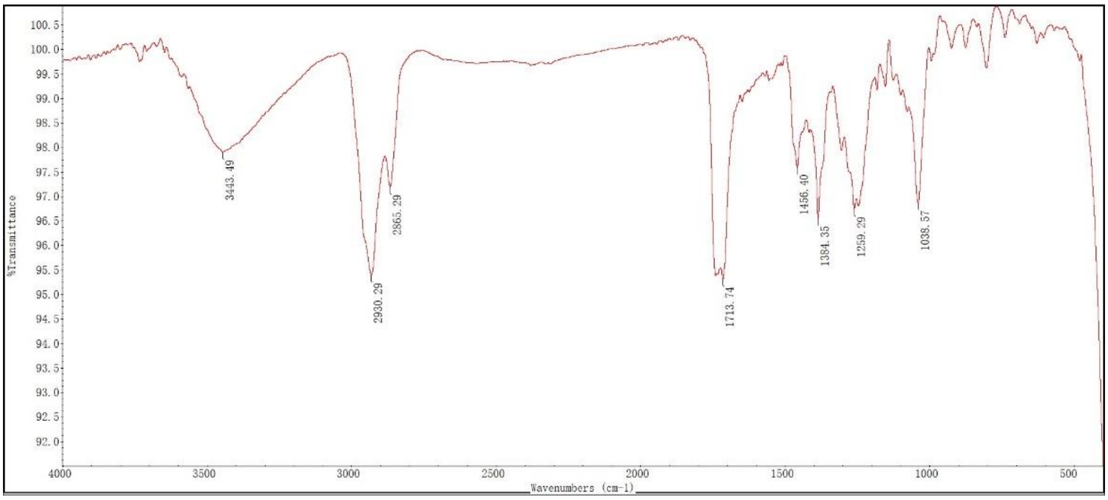


**Figure S102.** IR spectrum of compound **19**

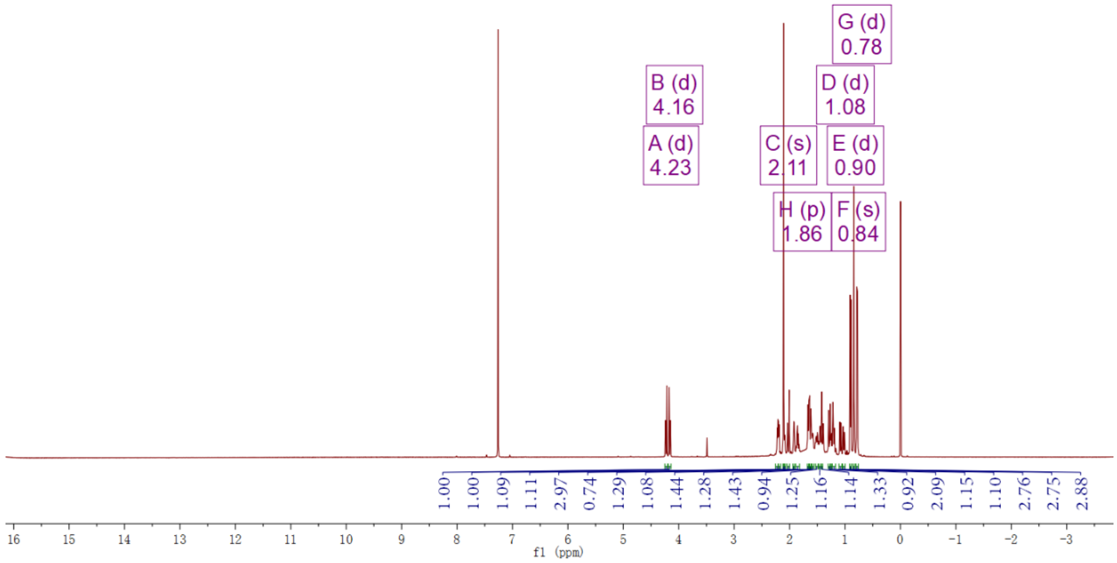


**Figure S103a.** $^{1}H$ NMR spectrum of compound **19** in $CDCl_3$

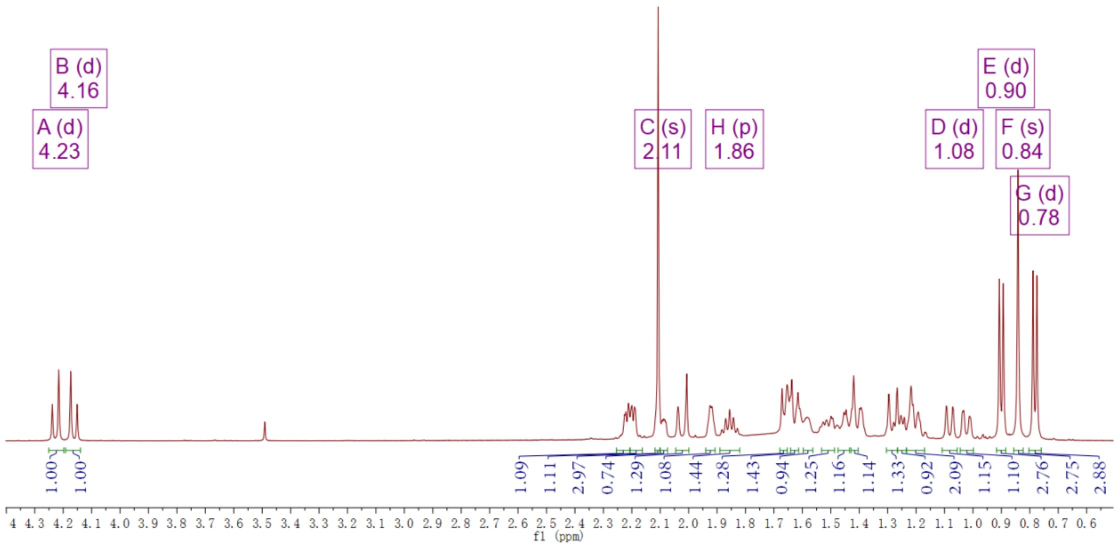


**Figure S103b.** Partial $^{1}H$ NMR spectrum ($\delta$ 0.6-4.4 ppm) of compound **19** in $CDCl_3$

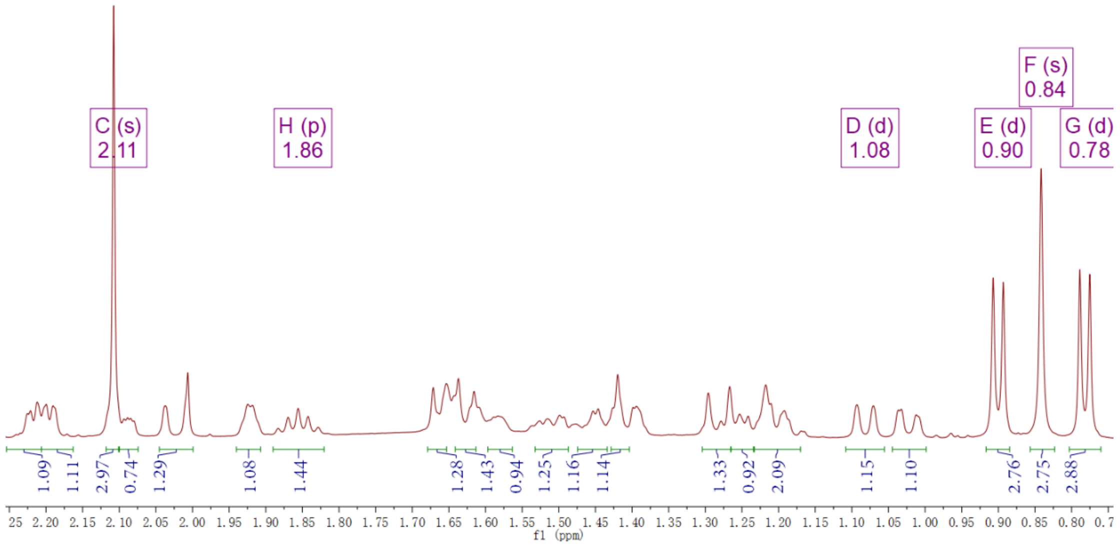


**Figure S103c.** Partial $^{1}H$ NMR spectrum ($\delta$ 0.7-2.3 ppm) of compound **19** in $CDCl_3$

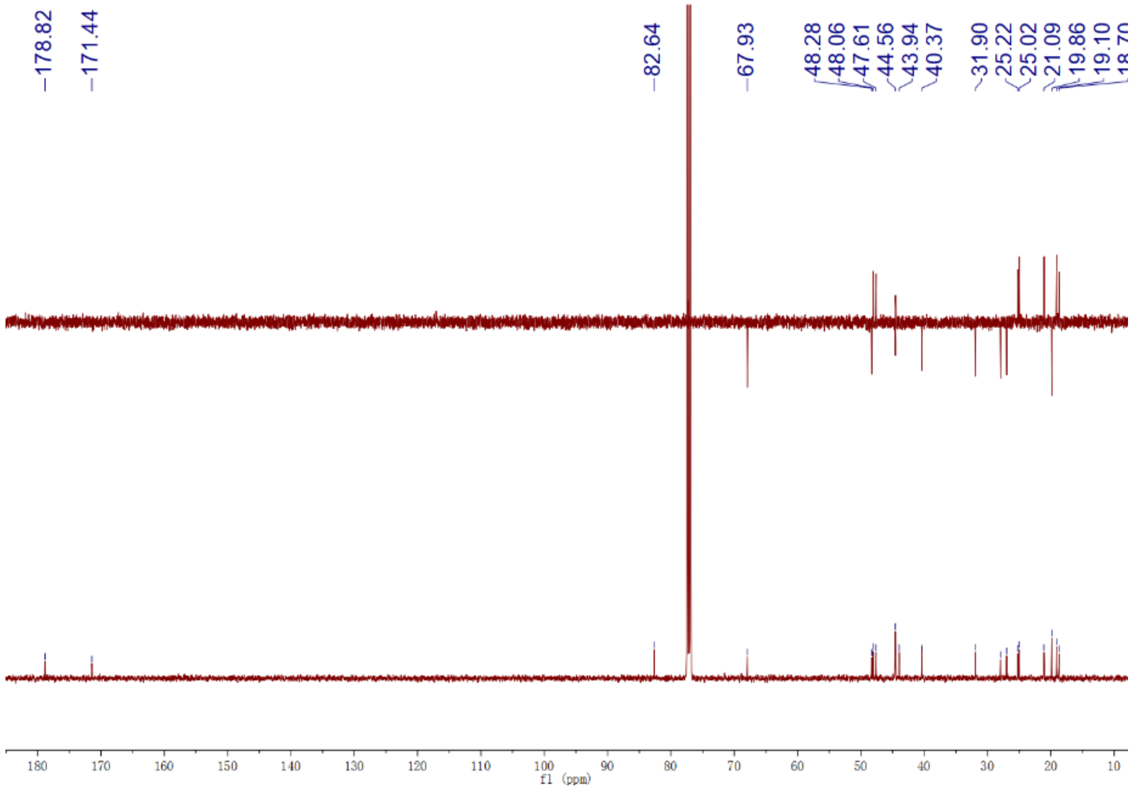


**Figure S104.** $^{13}C$ NMR and DEPT 135 spectrum of compound **19** in $CDCl_3$

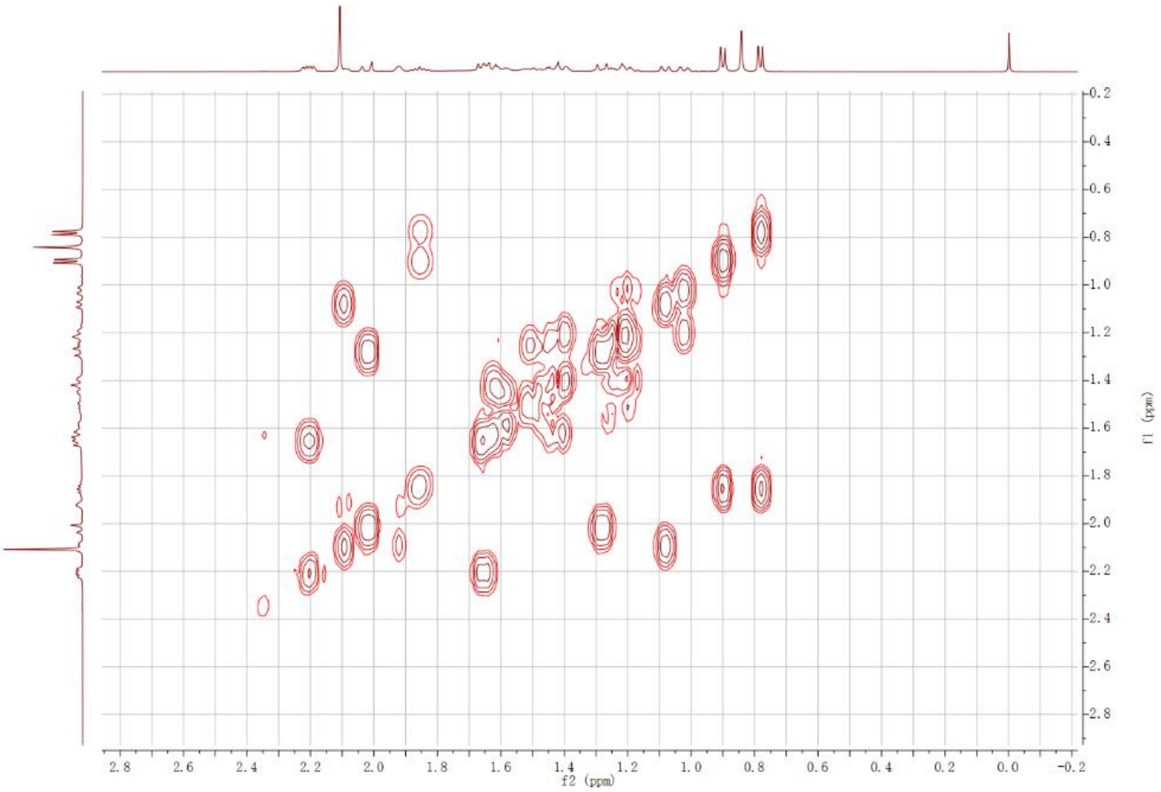


**Figure S105.** $^{1}H$-$^{1}H$ COSY spectrum of compound **19** in $CDCl_3$

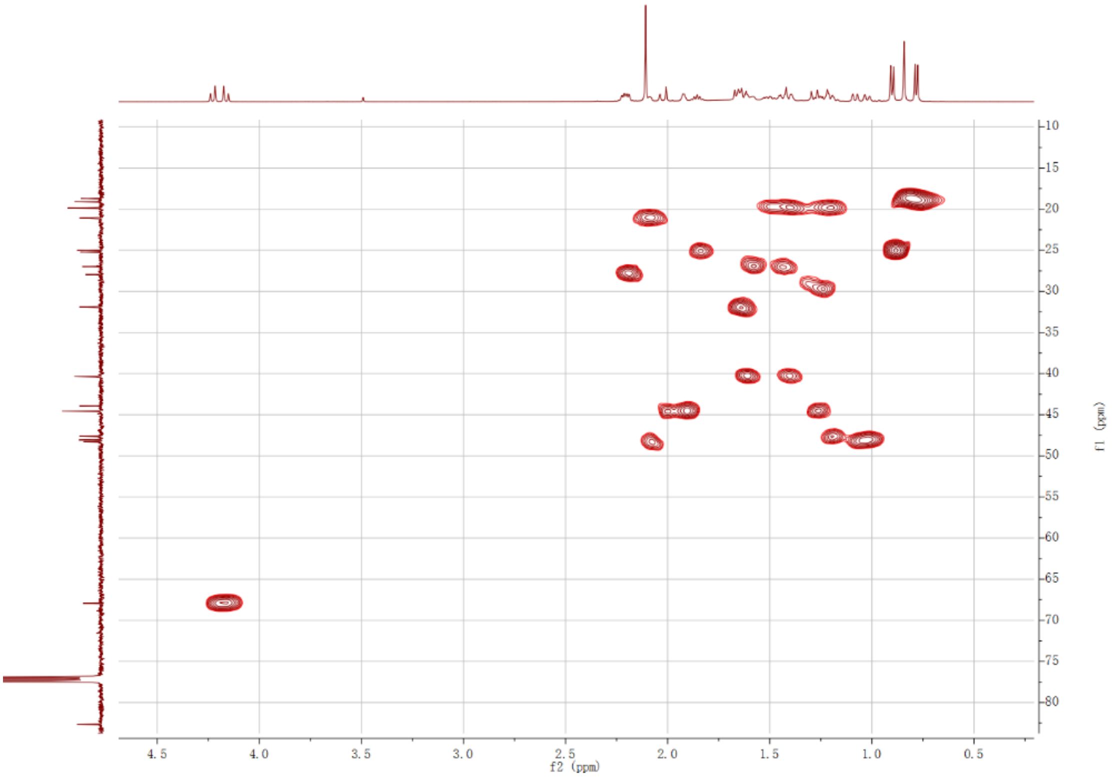


**Figure S106.** HSQC spectrum of compound **19** in $CDCl_3$

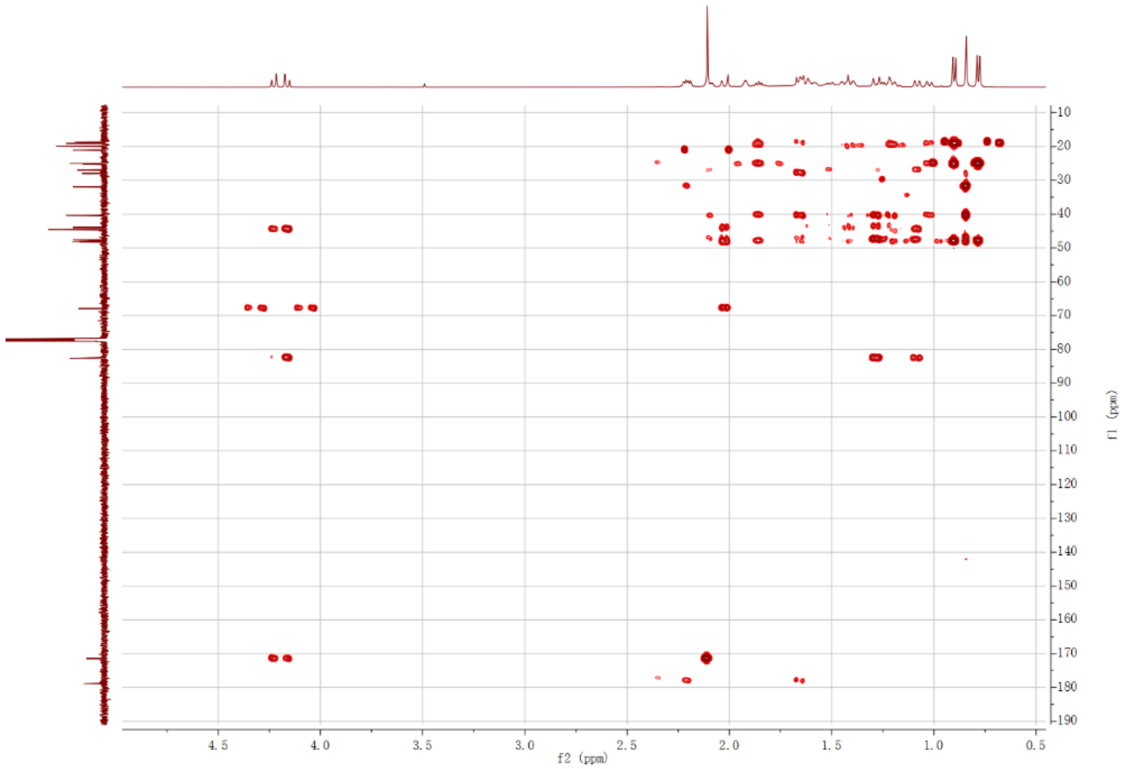


**Figure S107.** HMBC spectrum of compound **19** in $CDCl_3$

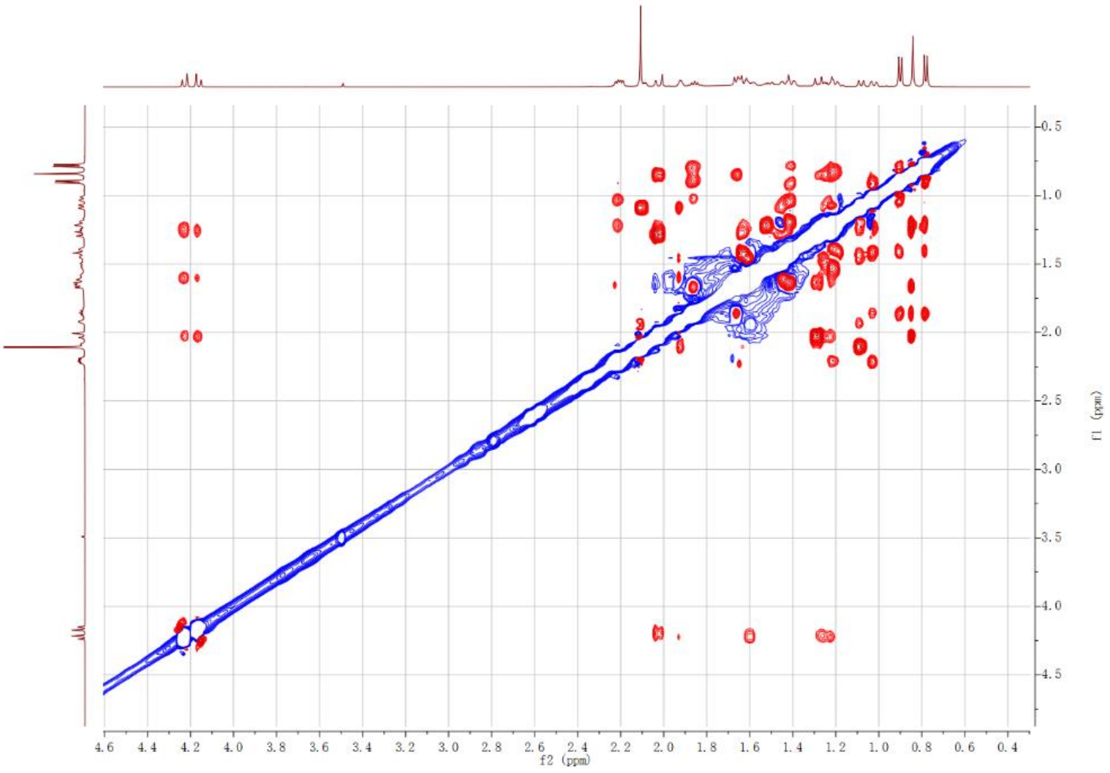


**Figure S108.** NOESY spectrum of compound **19** in $CDCl_3$

Tolerance = 5.0 PPM / DBE: min = -1.5, max = 50.0
Element prediction: Off
Number of isotope peaks used for i-FIT = 3

Monoisotopic Mass, Even Electron Ions
145 formula(e) evaluated with 1 results within limits (up to 50 closest results for each mass)
Elements Used:
C: 0-100 H: 0-200 O: 0-20 Na: 0-1
LX
20240422-LX-S009 1068 (7.797) 1: TOF MS ES+ 2.32e+004

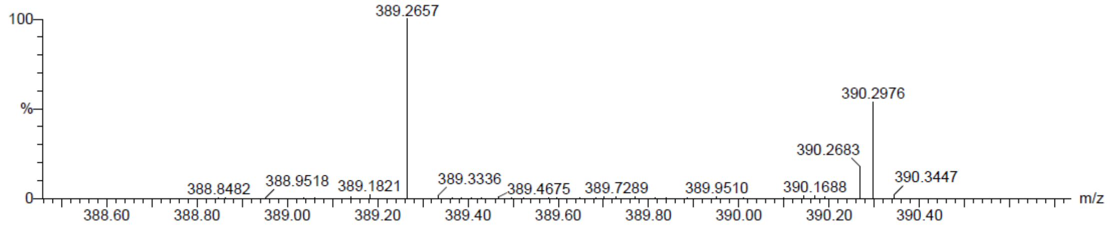


| | | | | | | | | | |
|---|---|---|---|---|---|---|---|---|---|
| Minimum: | 80.00 | | | | -1.5 | | | | |
| Maximum: | 100.00 | | 5.0 | 5.0 | 50.0 | | | | |
| Mass | RA | Calc. Mass | mDa | PPM | DBE | i-FIT | Norm | Conf(%) | Formula |
| 389.2657 | 100.00 | 389.2668 | -1.1 | -2.8 | 3.5 | 269.6 | n/a | n/a | C22 H38 O4 Na |

**Figure S109.** HRESIMS spectrum of compound **20**

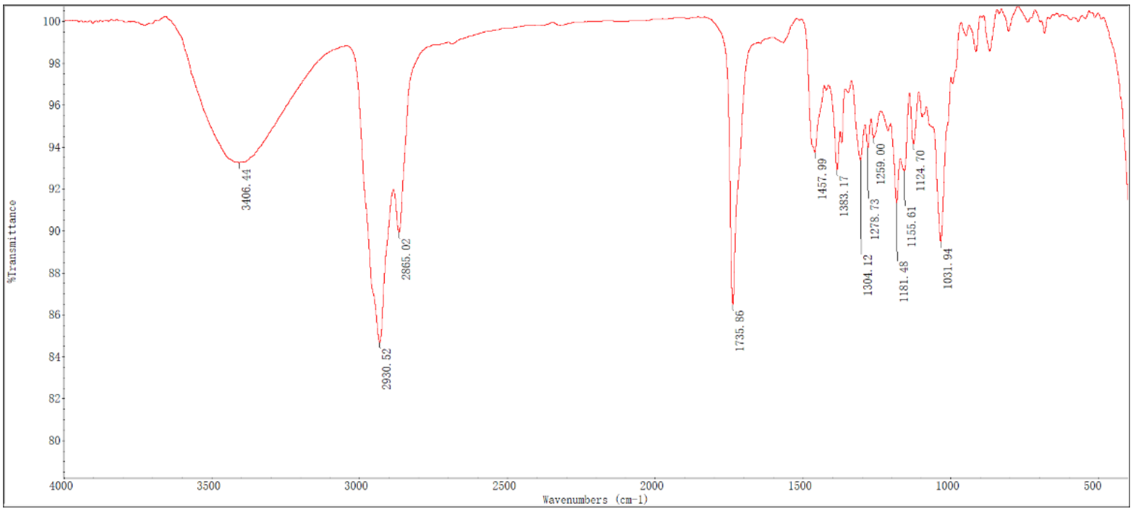


**Figure S110.** IR spectrum of compound **20**

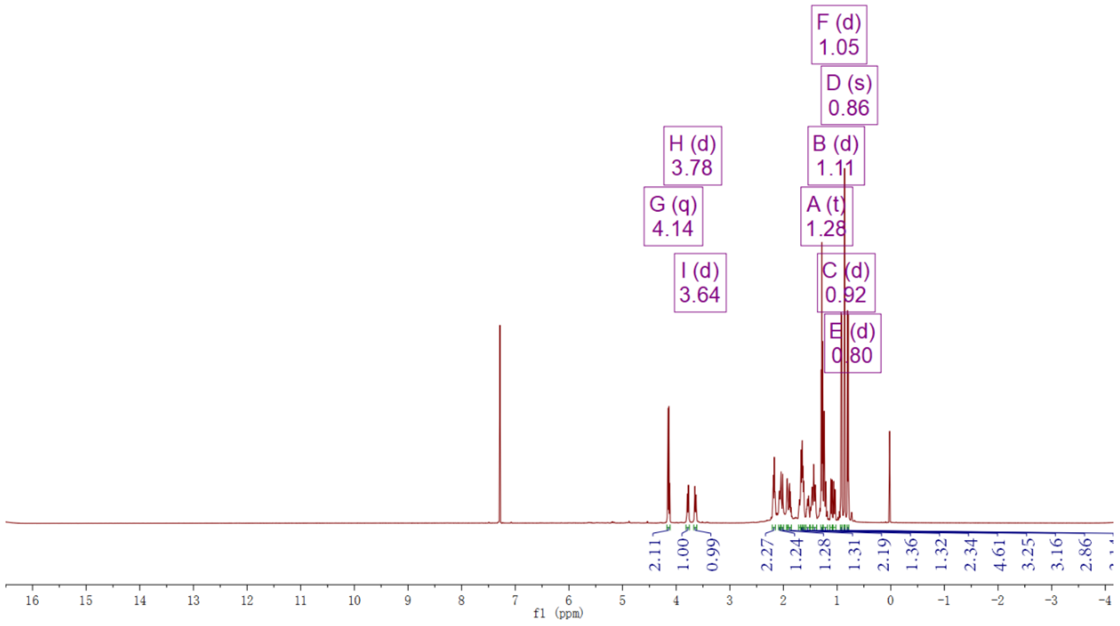


**Figure S111a.** $^{1}$H NMR spectrum of compound **20** in $CDCl_3$

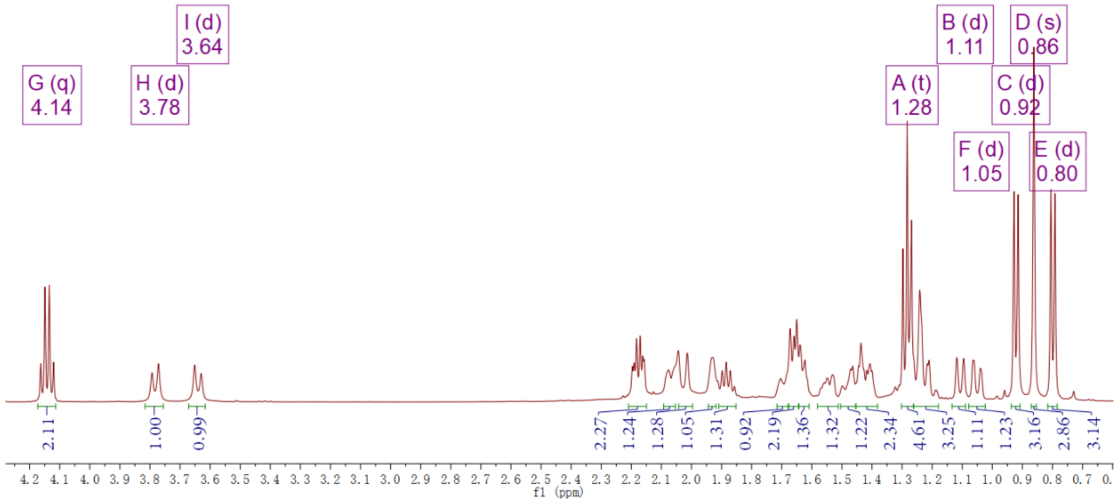


**Figure S111b.** Partial $^{1}$H NMR spectrum ($\delta$ 0.6-4.3 ppm) of compound **20** in $CDCl_3$

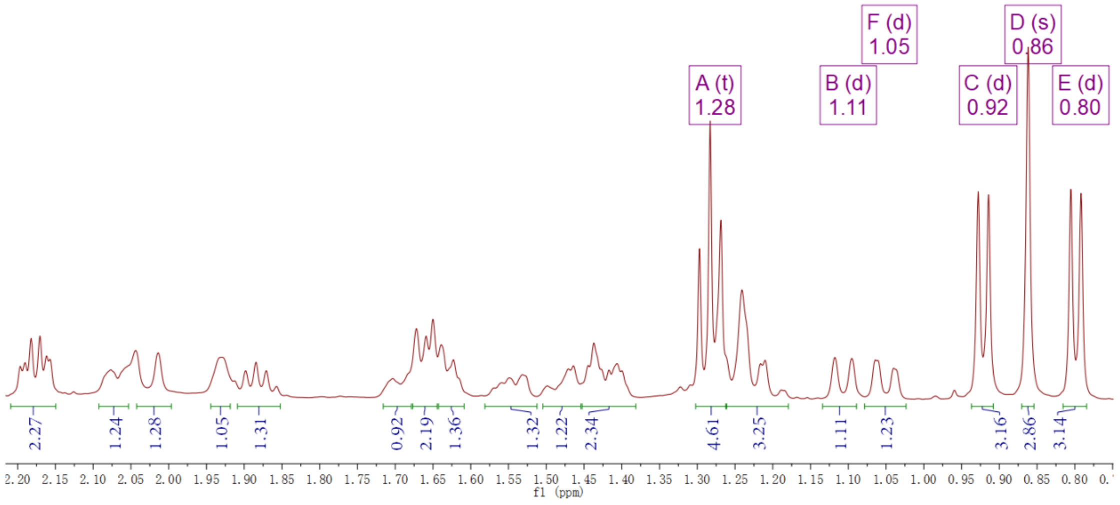


**Figure S111c.** Partial $^{1}H$ NMR spectrum ($\delta$ 0.7-2.3 ppm) of compound **20** in $CDCl_3$

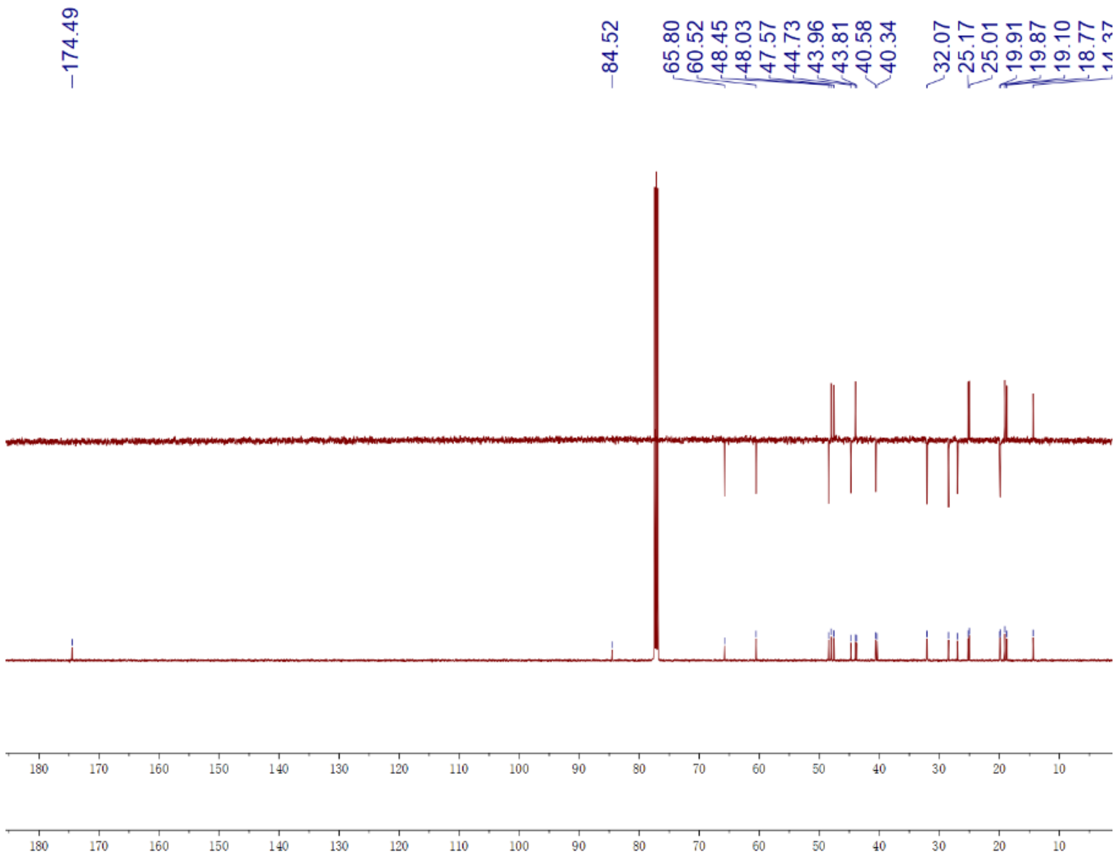


**Figure S112** $^{13}C$ NMR and DEPT 135 spectrum of compound **20** in $CDCl_3$

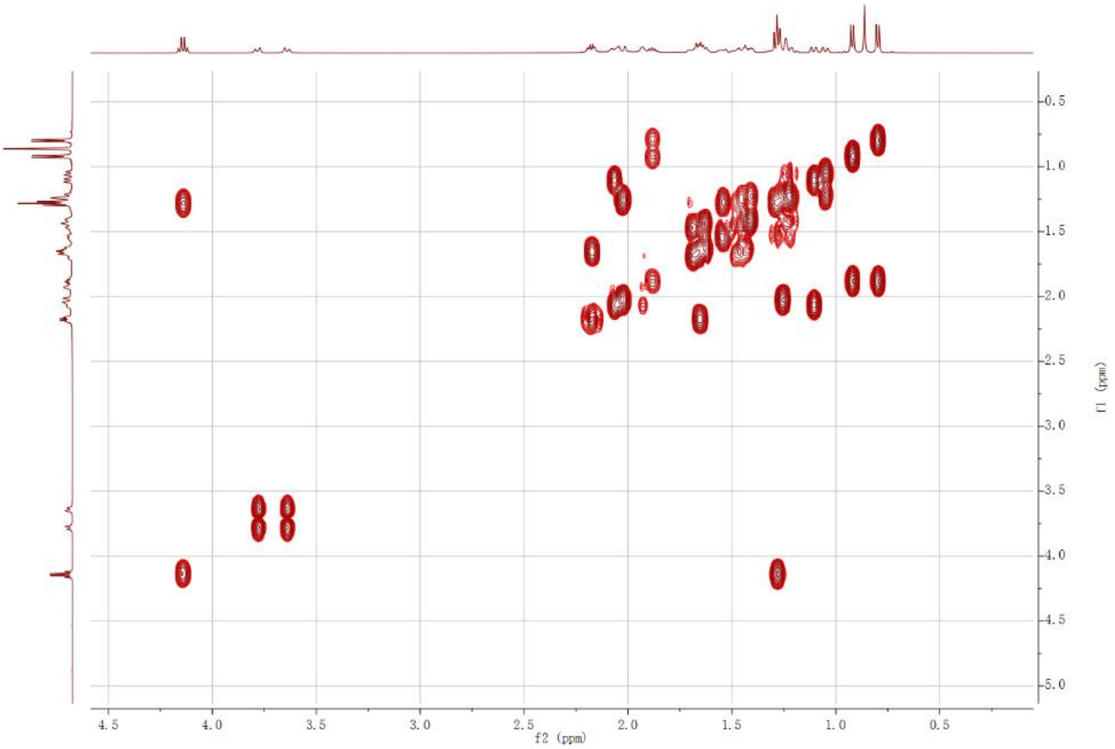


**Figure S113.** $^{1}H$-$^{1}H$ COSY spectrum of compound **20** in $CDCl_3$

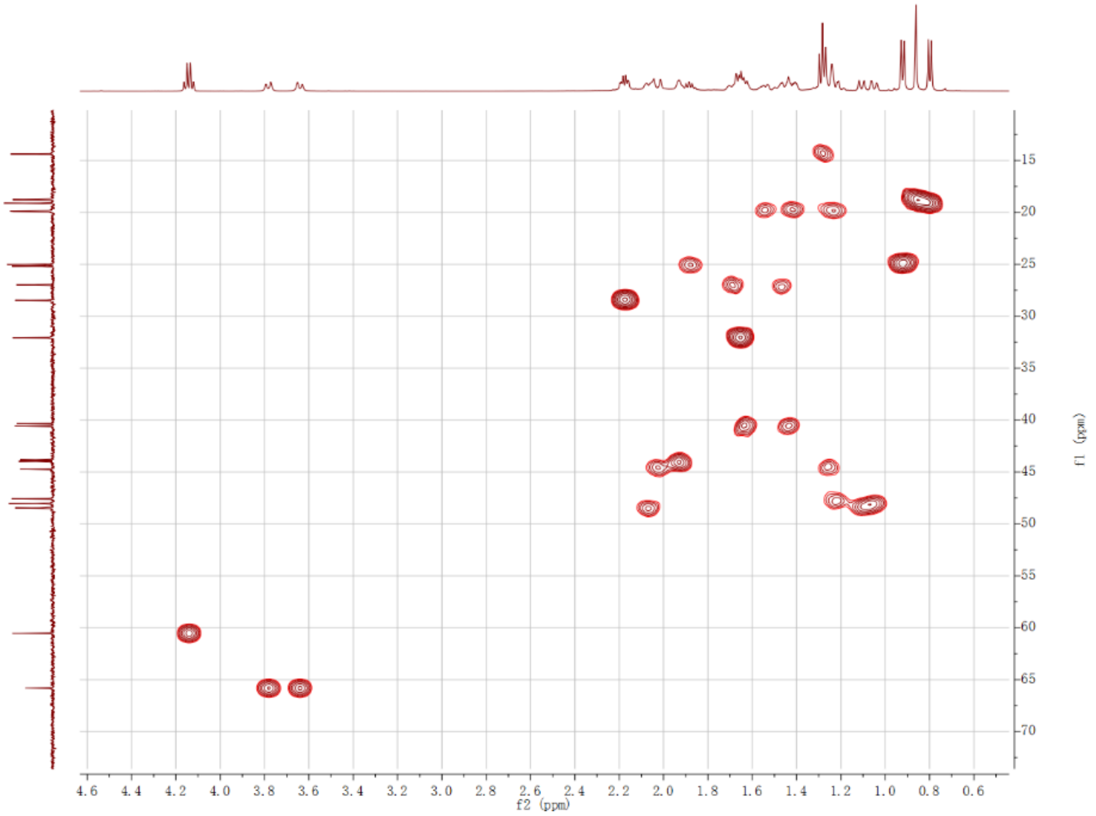


**Figure S114.** HSQC spectrum of compound **20** in $CDCl_3$

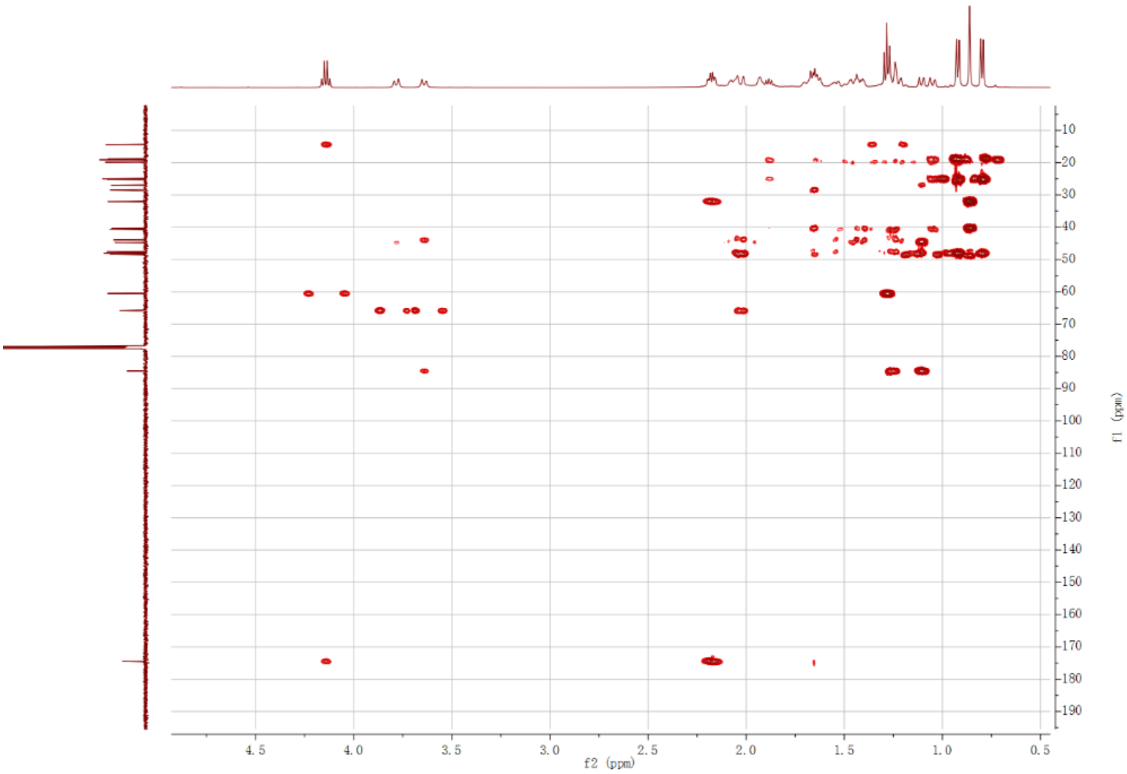


**Figure S115.** HMBC spectrum of compound **20** in $CDCl_3$

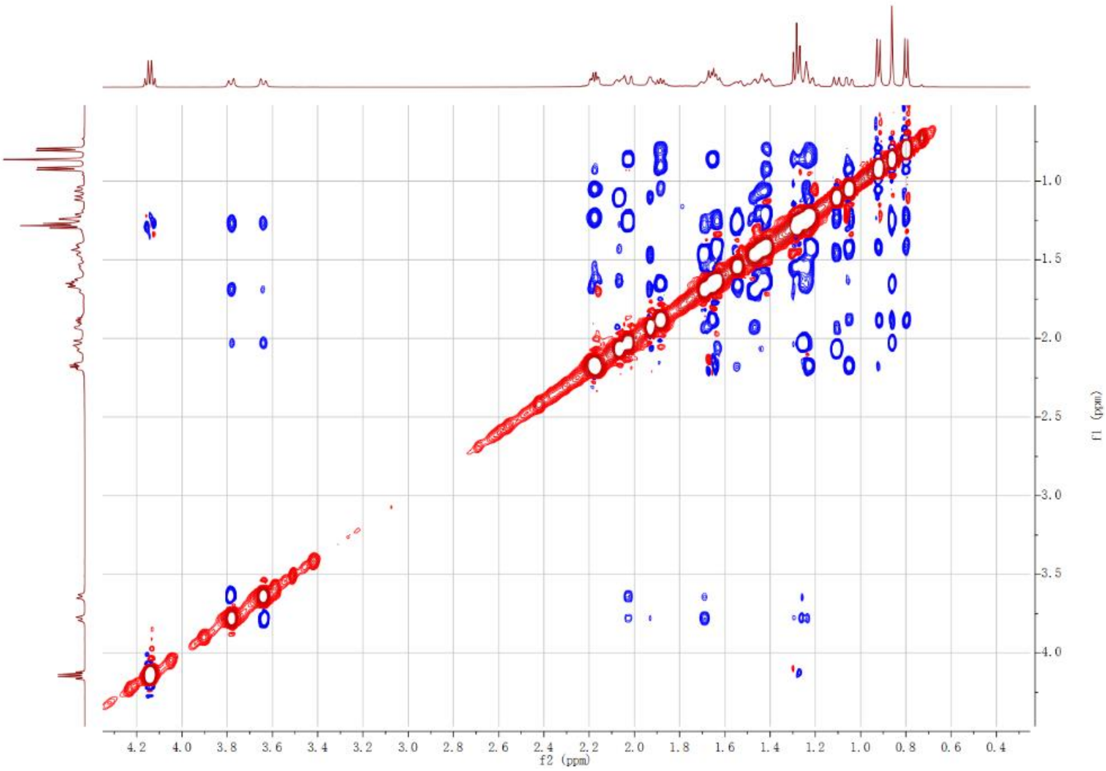


**Figure S116.** NOESY spectrum of compound **20** in $CDCl_3$

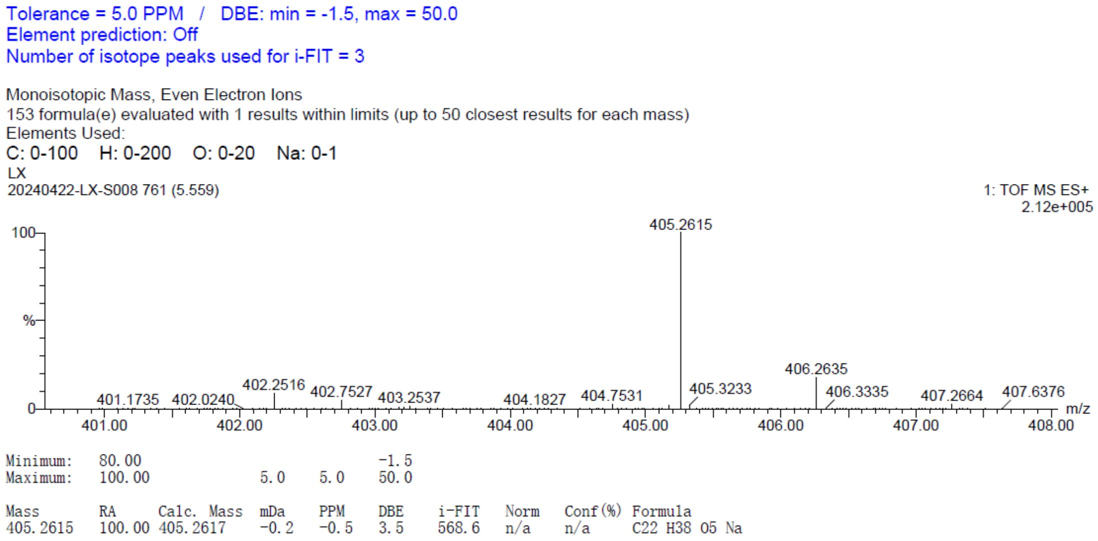


**Figure S117.** HRESIMS spectrum of compound **21**

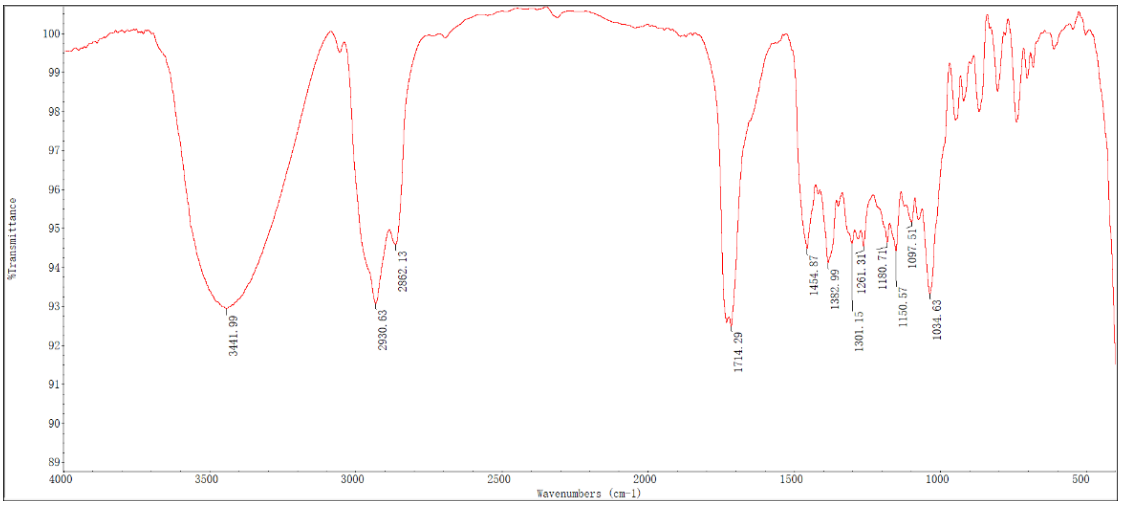


**Figure S118.** IR spectrum of compound **21**

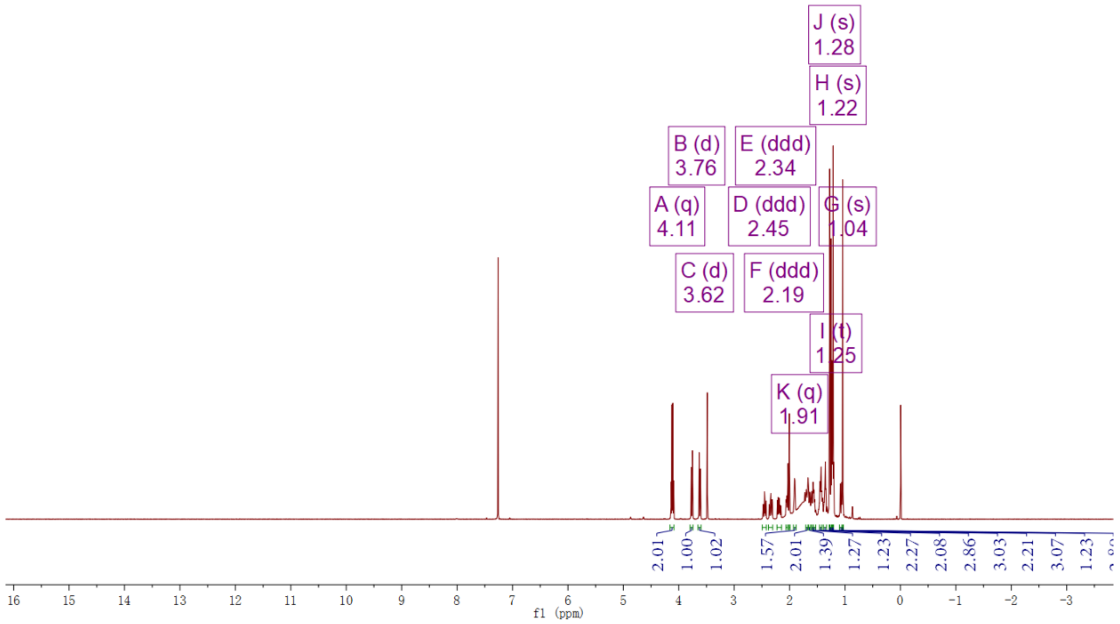


**Figure S119a.** $^{1}$H NMR spectrum of compound **21** in $CDCl_3$

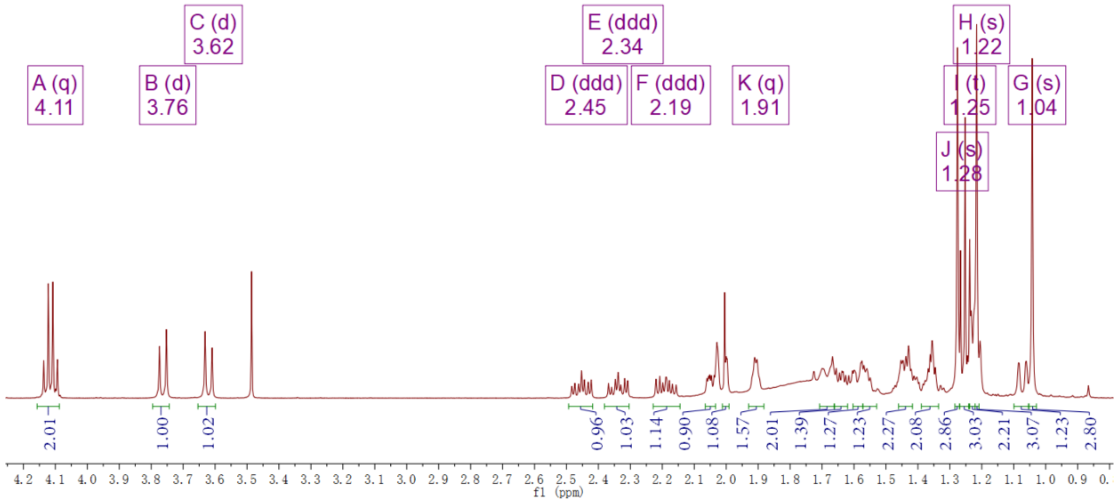


**Figure S119b.** Partial $^{1}$H NMR spectrum ($\delta$ 0.8-4.3 ppm) of compound **21** in $CDCl_3$

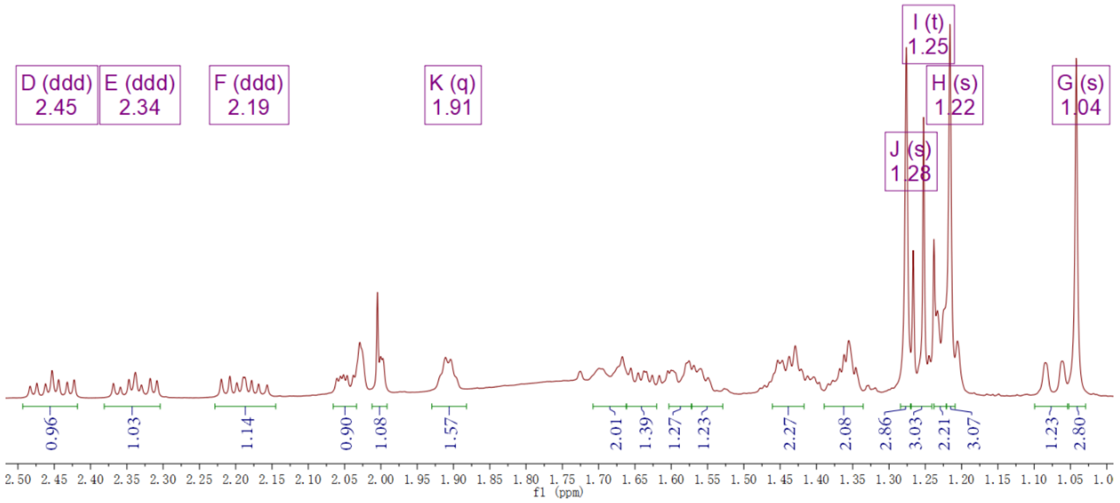


**Figure S119c.** Partial $^{1}$H NMR spectrum ($\delta$ 1.0-2.5 ppm) of compound **21** in $CDCl_3$

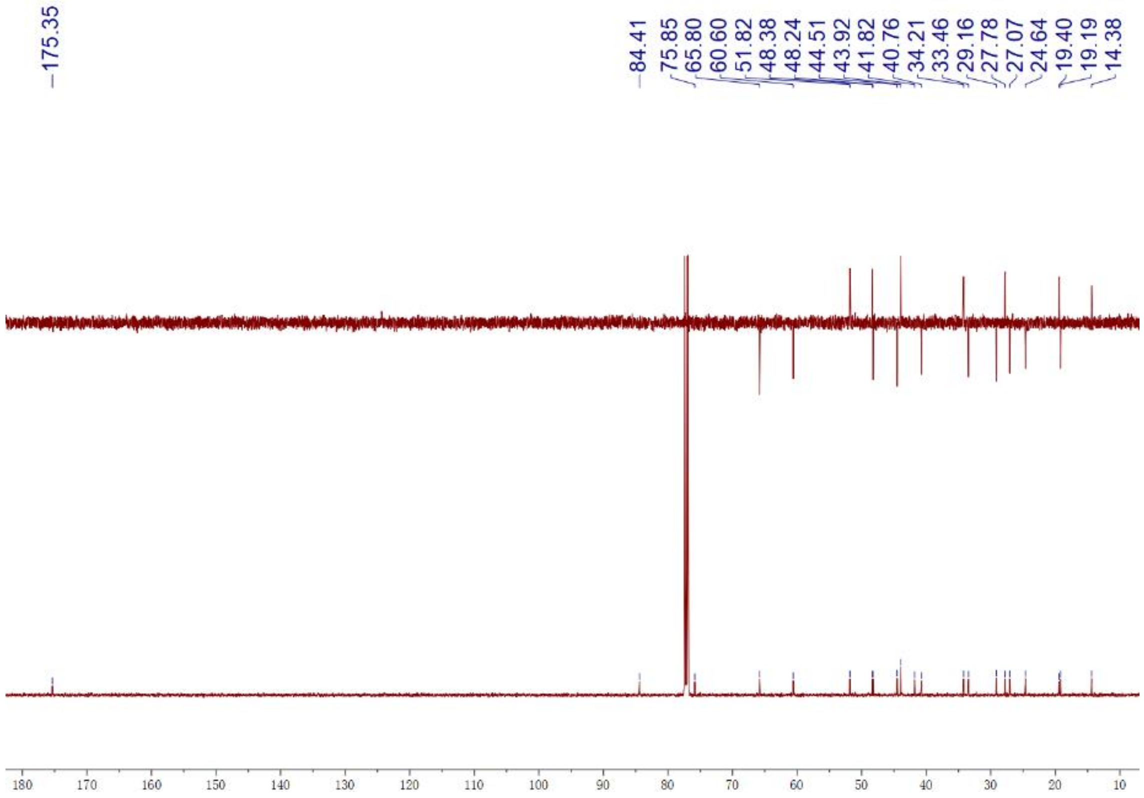


**Figure S120** $^{13}$C NMR and DEPT 135 spectrum of compound **21** in $CDCl_3$

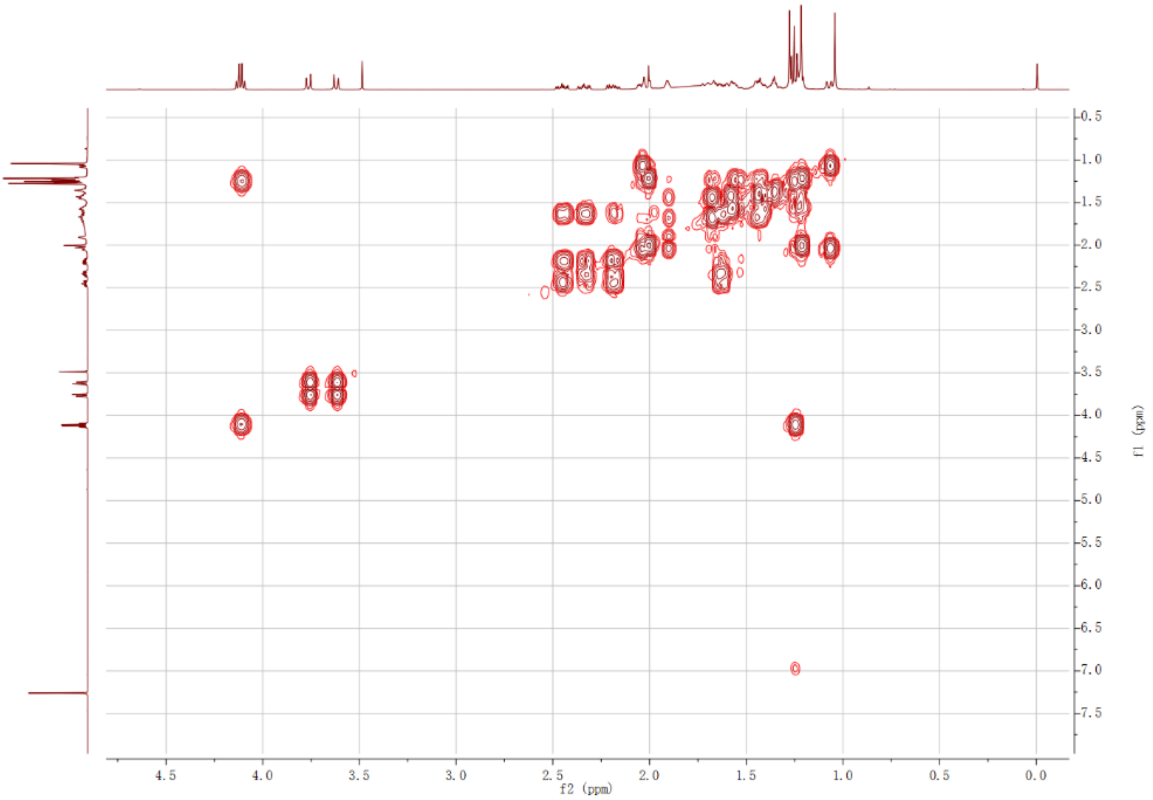


**Figure S121.** $^{1}H$-$^{1}H$ COSY spectrum of compound **21** in $CDCl_3$

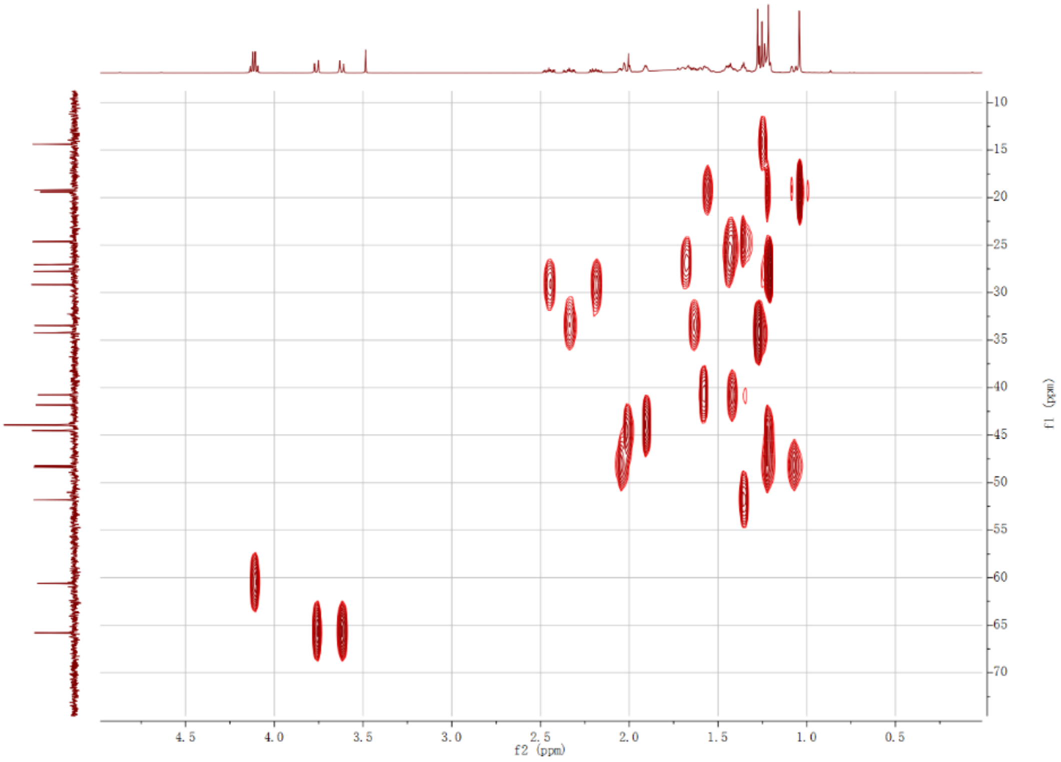


**Figure S122.** HSQC spectrum of compound **21** in $CDCl_3$

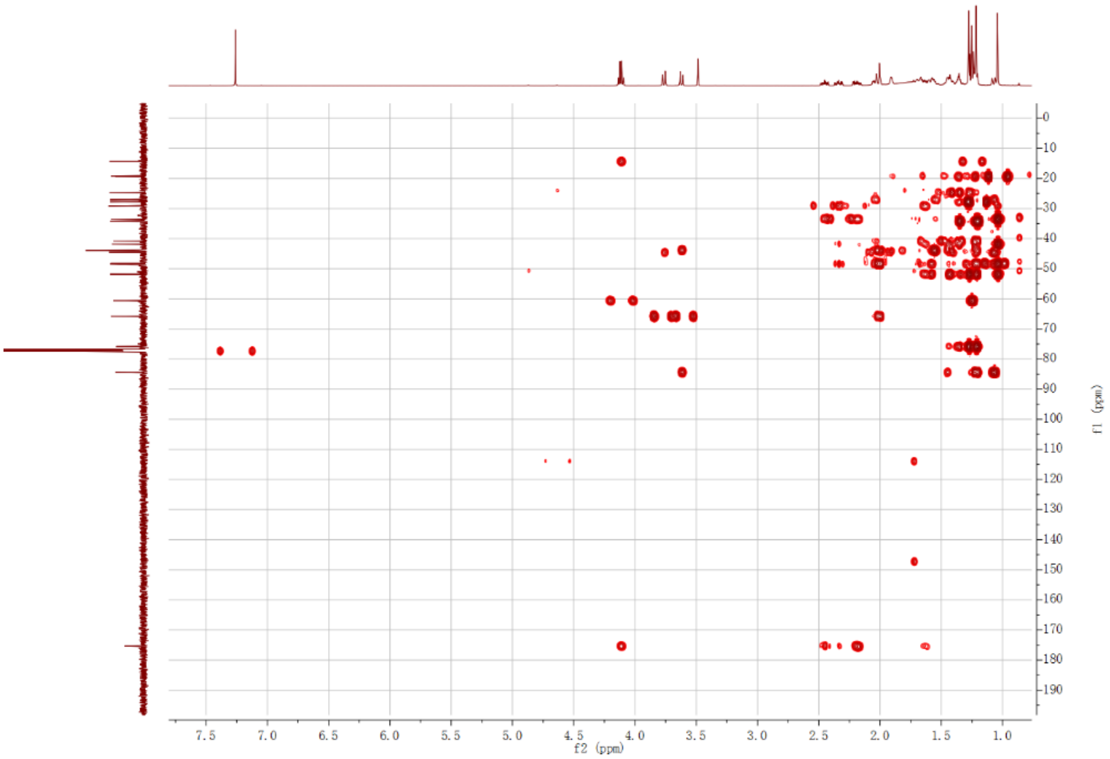


**Figure S123.** HMBC spectrum of compound **21** in $CDCl_3$

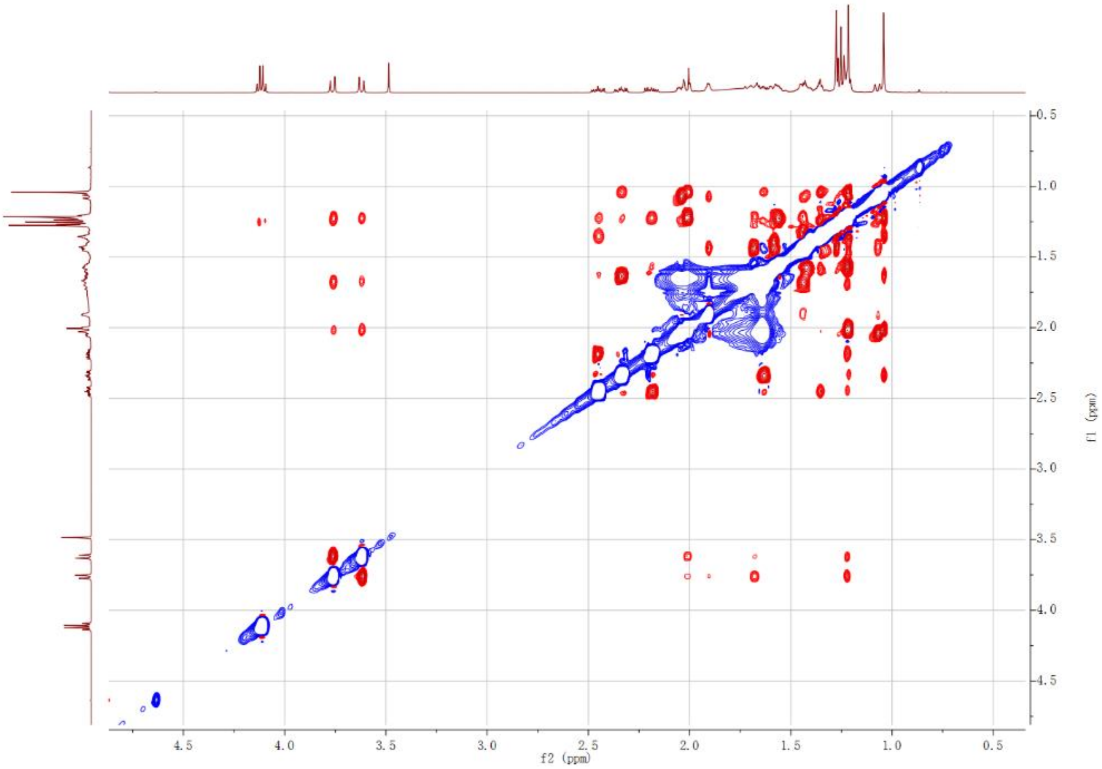


**Figure S124.** NOESY spectrum of compound **21** in $CDCl_3$

Tolerance = 5.0 PPM / DBE: min = -1.5, max = 50.0
Element prediction: Off
Number of isotope peaks used for i-FIT = 3

Monoisotopic Mass, Even Electron Ions
181 formula(e) evaluated with 1 results within limits (up to 50 closest results for each mass)
Elements Used:
C: 0-100 H: 0-200 O: 0-20 Na: 0-1
LX
20240422-LX-S012 905 (6.607) 1: TOF MS ES+ 2.23e+005

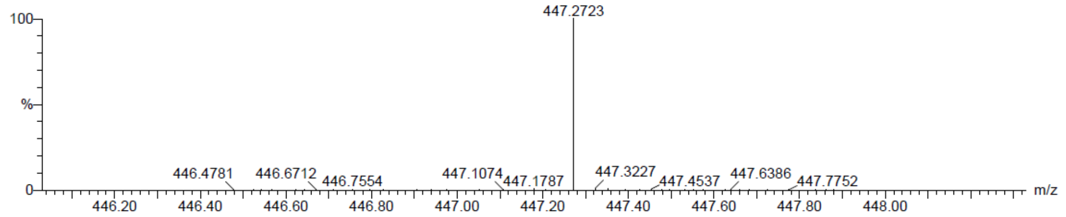


| | | | | | | | | | |
|---|---|---|---|---|---|---|---|---|---|
| Minimum: | 80.00 | | | | -1.5 | | | | |
| Maximum: | 100.00 | | 5.0 | 5.0 | 50.0 | | | | |
| Mass | RA | Calc. Mass | mDa | PPM | DBE | i-FIT | Norm | Conf(%) | Formula |
| 447.2723 | 100.00 | 447.2723 | 0.0 | 0.0 | 4.5 | 239.4 | n/a | n/a | C24 H40 O6 Na |

**Figure S125.** HRESIMS spectrum of compound **22**

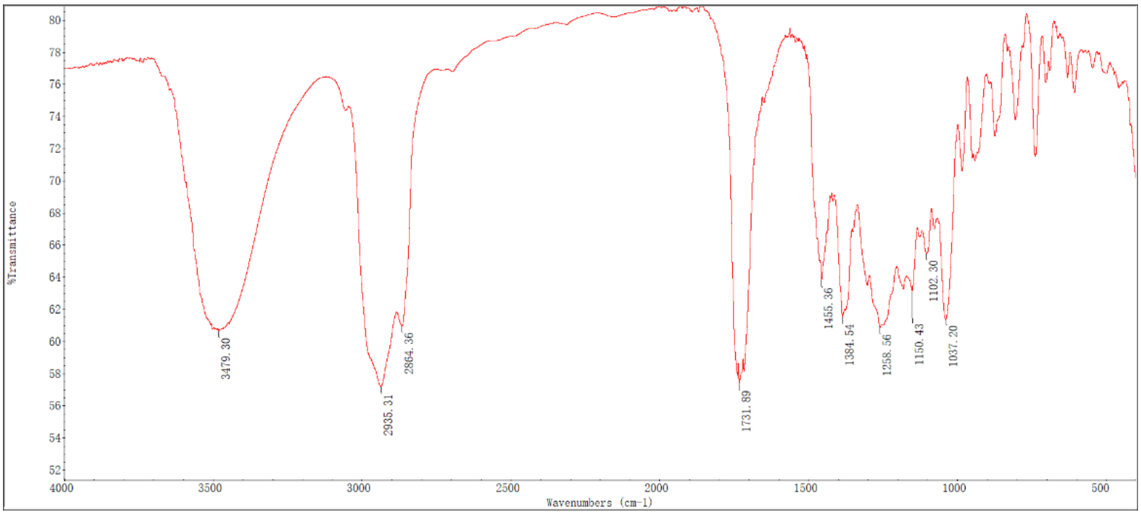


**Figure S126.** IR spectrum of compound **22**

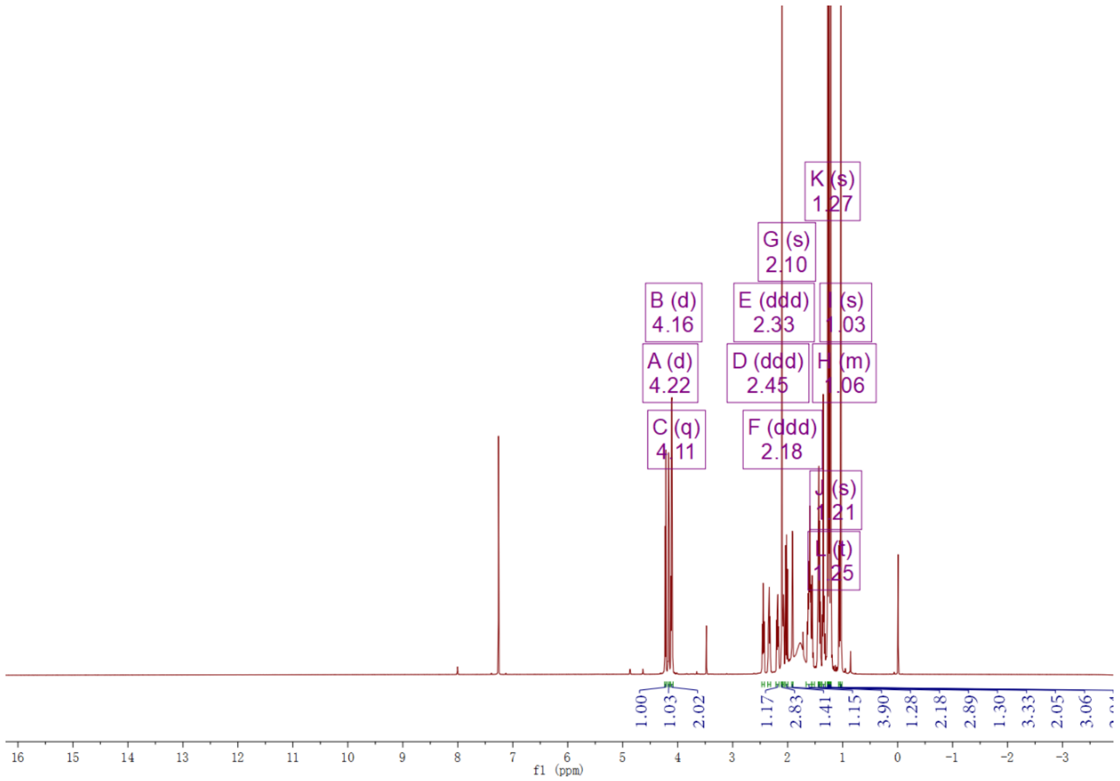


**Figure S127a.** $^{1}$H NMR spectrum of compound **22** in $CDCl_3$

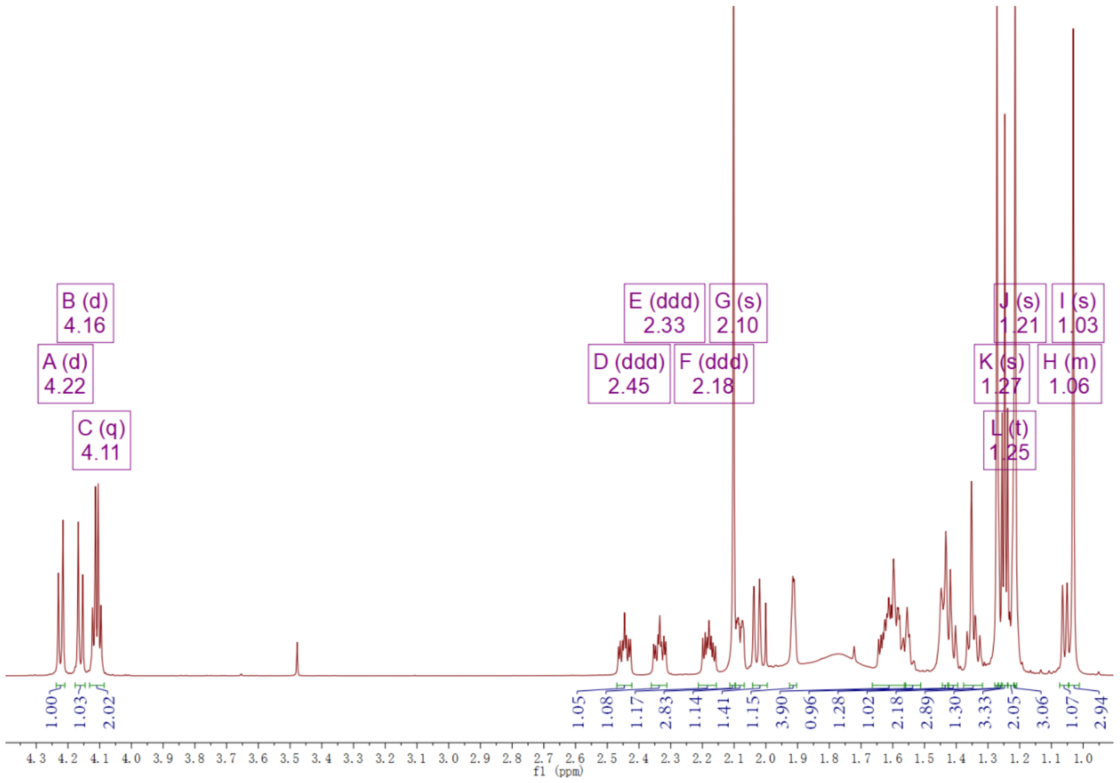


**Figure S127b.** Partial $^{1}$H NMR spectrum ($\delta$ 0.9-4.3 ppm) of compound **22** in $CDCl_3$

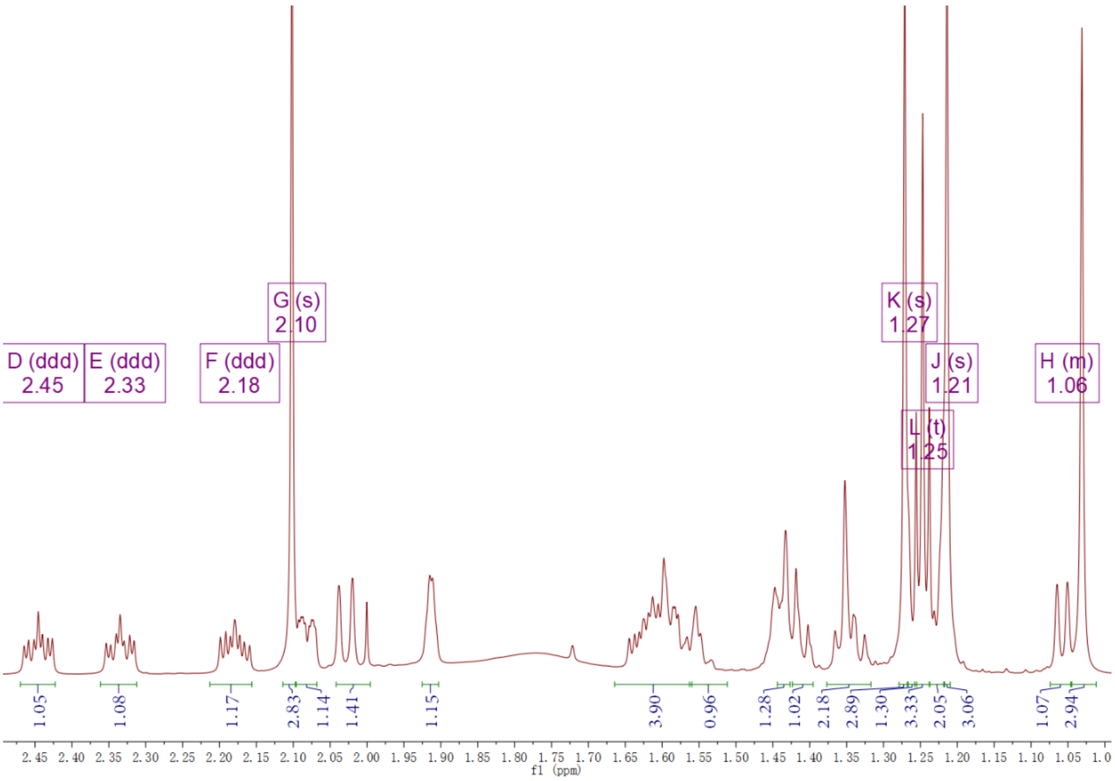


**Figure S127c.** Partial $^{1}$H NMR spectrum($\delta$ 1.0-2.5 ppm) of compound **22** in $CDCl_3$

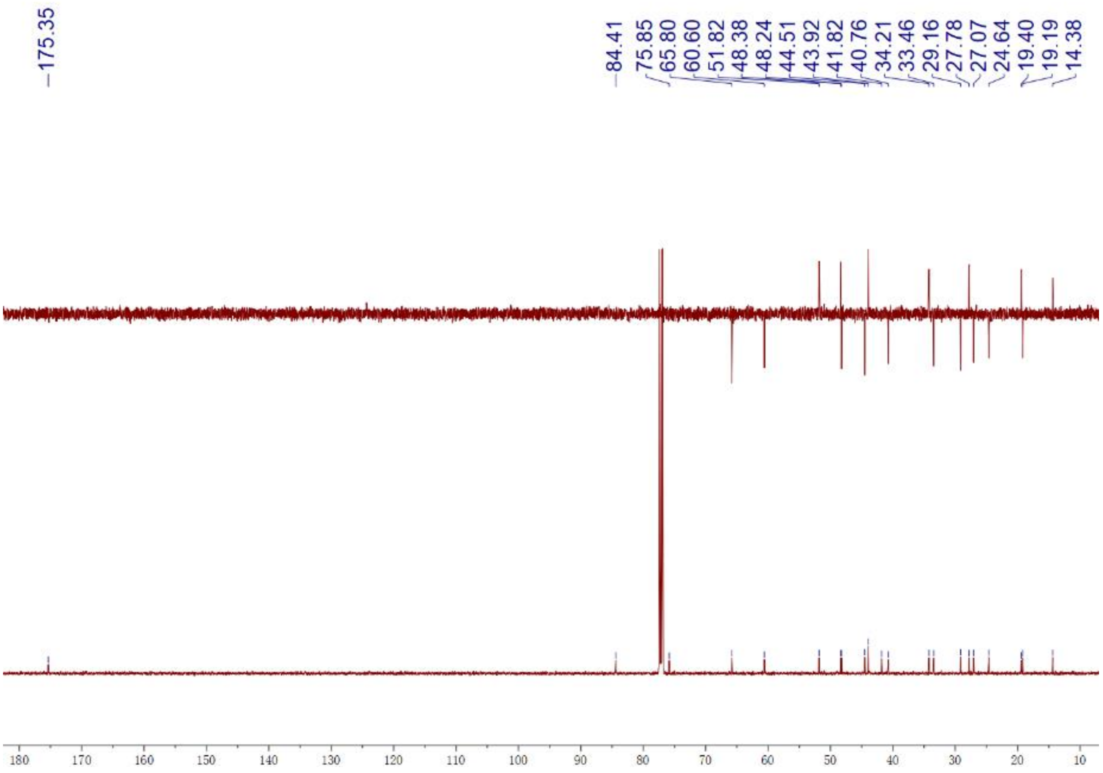


**Figure S128** $^{13}$C NMR and DEPT 135 spectrum of compound **22** in $CDCl_3$

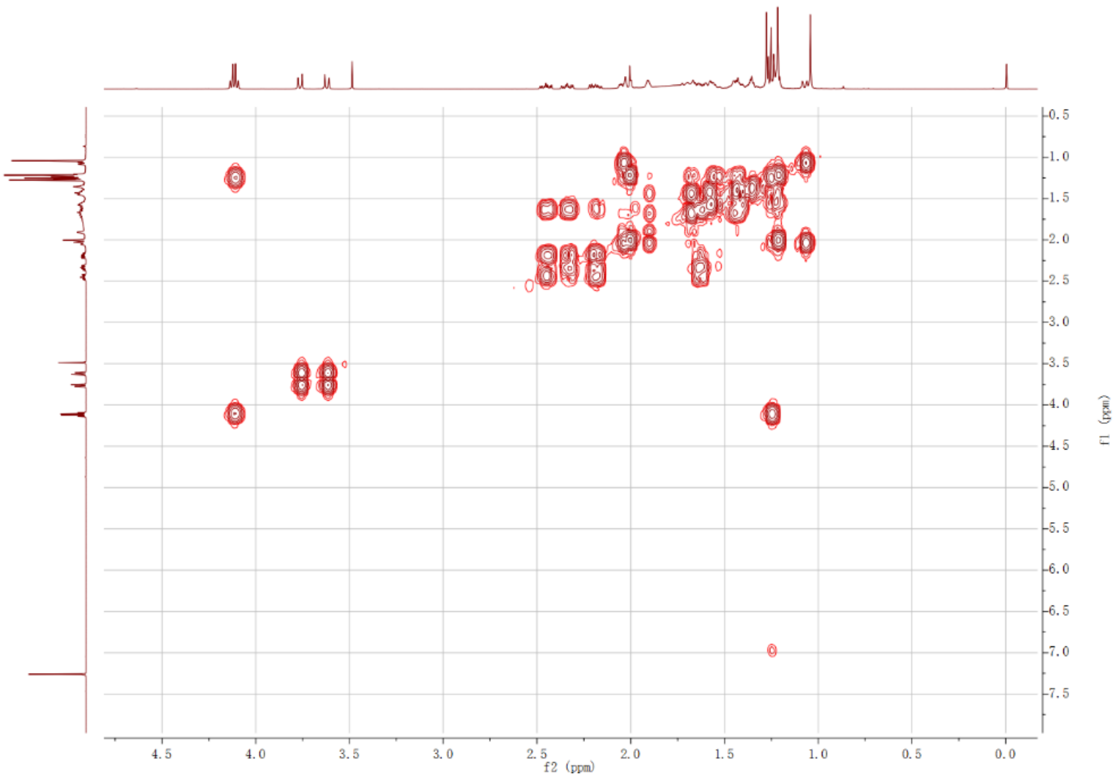


**Figure S129.** $^{1}$H-$^{1}$H COSY spectrum of compound **22** in $CDCl_3$

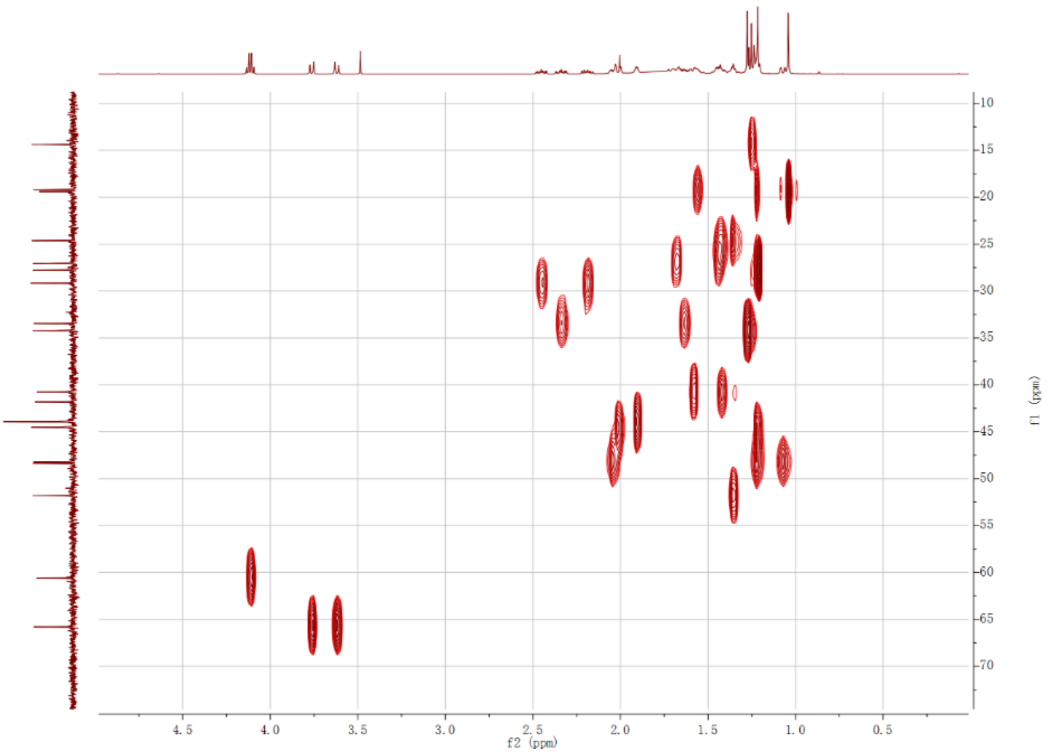


**Figure S130.** HSQC spectrum of compound **22** in $CDCl_3$

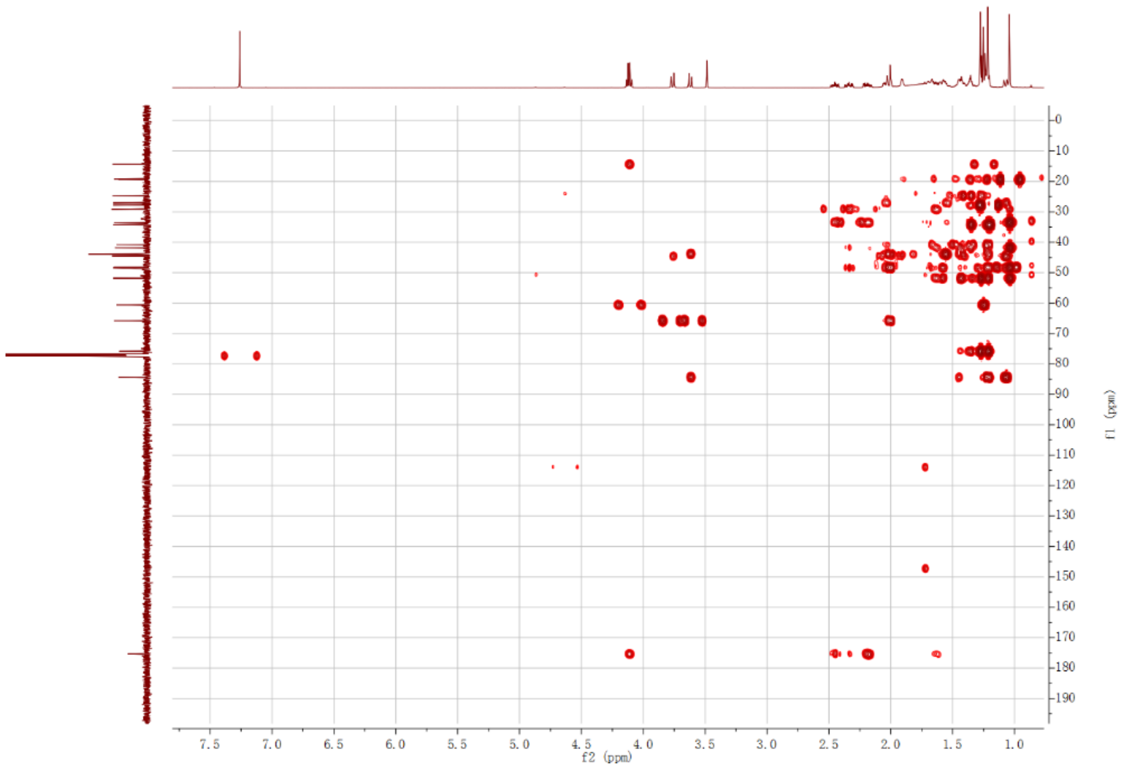


**Figure S131.** HMBC spectrum of compound **22** in $CDCl_3$

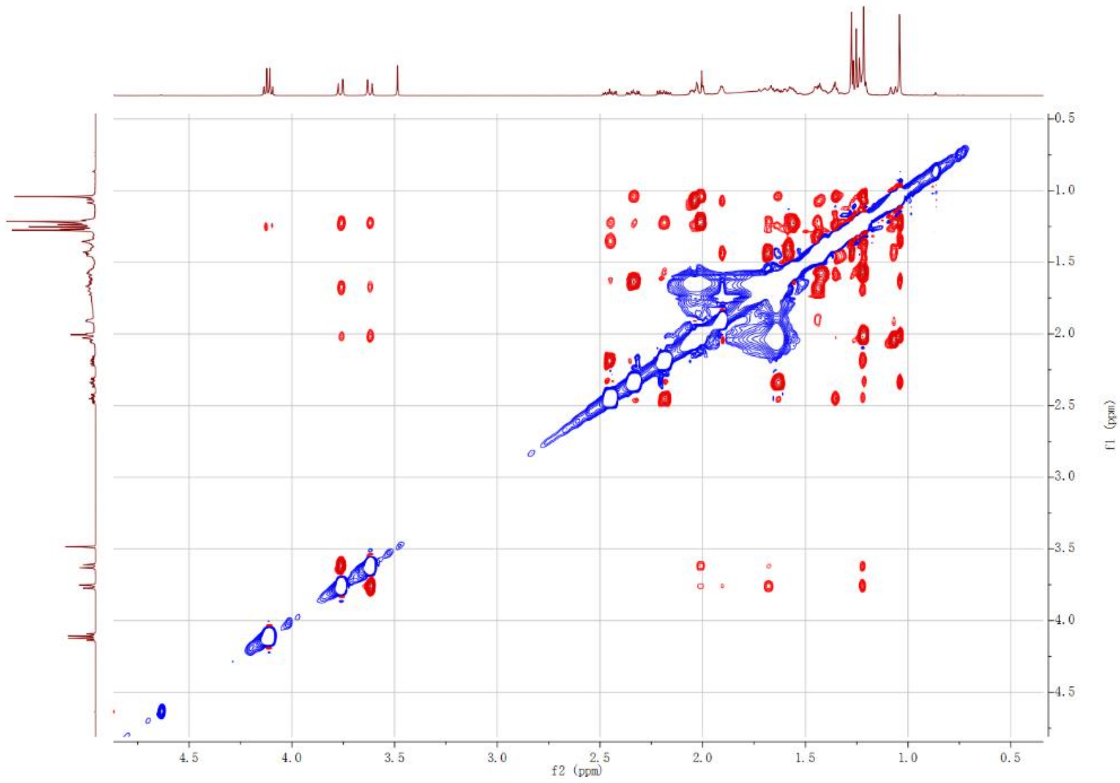


**Figure S132.** NOESY spectrum of compound **22** in $CDCl_3$

Tolerance = 5.0 PPM / DBE: min = -1.5, max = 50.0
Element prediction: Off
Number of isotope peaks used for i-FIT = 3

Monoisotopic Mass, Even Electron Ions
297 formula(e) evaluated with 3 results within limits (up to 50 closest results for each mass)
Elements Used:
C: 0-100 H: 0-200 O: 0-20 Na: 0-1
LX
20240422-LX-S018 1030 (7.520)

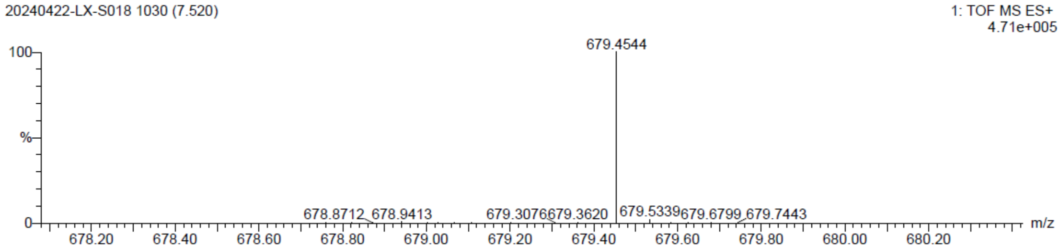


| | | | | | | | | | |
|---|---|---|---|---|---|---|---|---|---|
| Minimum: | 80.00 | | | | -1.5 | | | | |
| Maximum: | 100.00 | | 5.0 | 5.0 | 50.0 | | | | |
| Mass | RA | Calc. Mass | mDa | PPM | DBE | i-FIT | Norm | Conf(%) | Formula |
| 679.4544 | 100.00 | 679.4550 | -0.6 | -0.9 | 8.5 | 115.4 | 0.349 | 70.52 | C40 H64 O7 Na |
| | | 679.4515 | 2.9 | 4.3 | 20.5 | 116.9 | 1.940 | 14.37 | C49 H59 O2 |
| | | 679.4574 | -3.0 | -4.4 | 11.5 | 116.9 | 1.890 | 15.10 | C42 H63 O7 |

**Figure S133.** HRESIMS spectrum of compound **23**

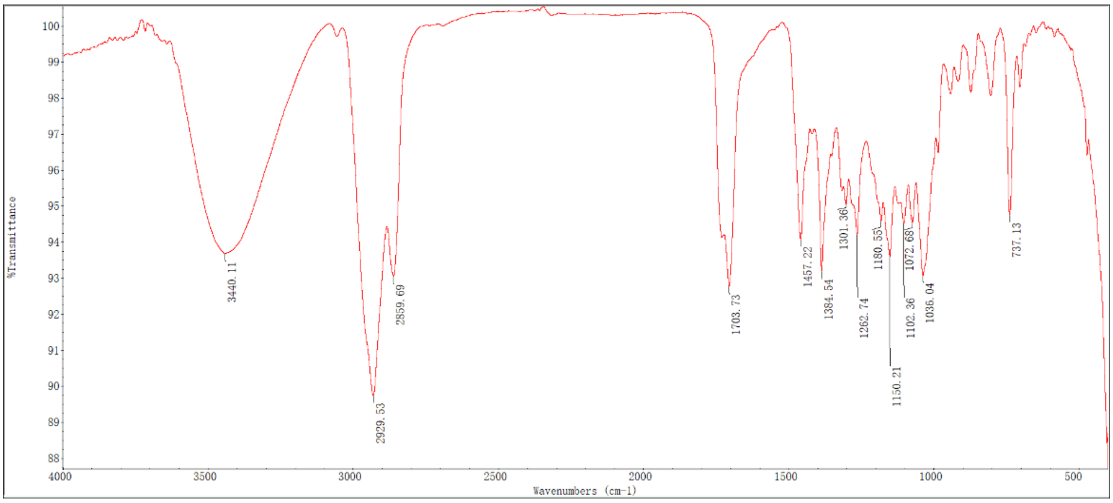


**Figure S134.** IR spectrum of compound **23**

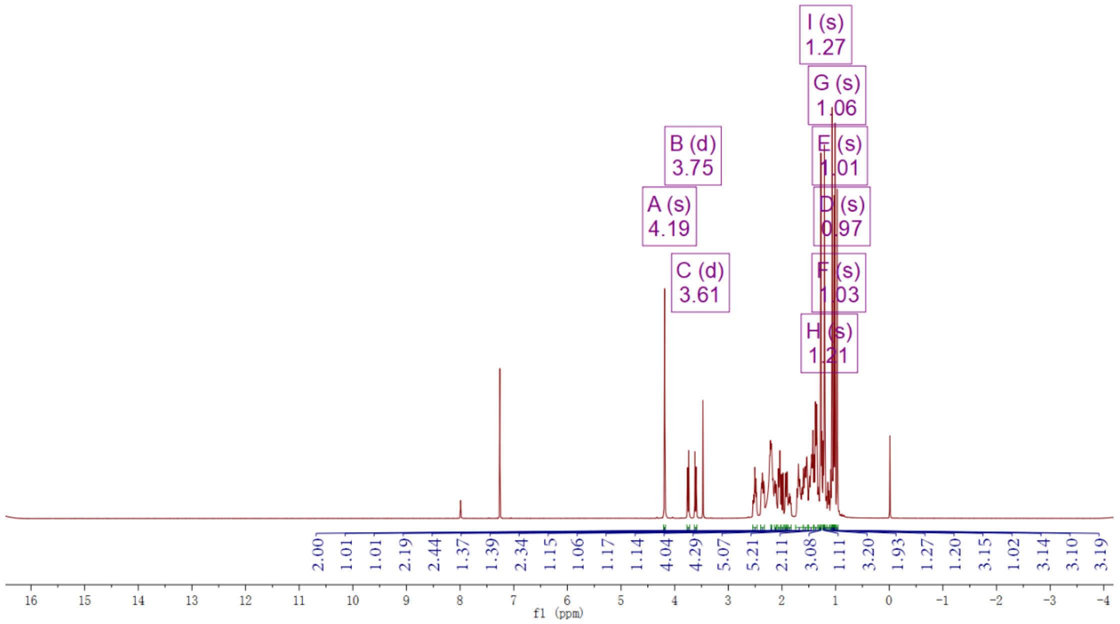


**Figure S135a.** $^{1}$H NMR spectrum of compound **23** in $CDCl_3$

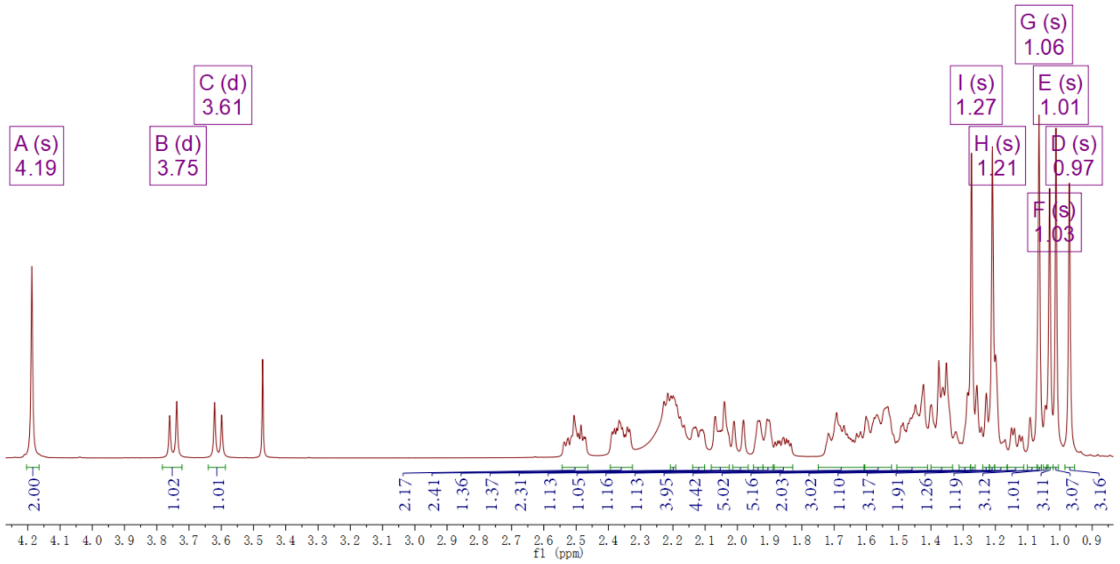


**Figure S135b.** Partial $^{1}$H NMR spectrum ($\delta$ 0.8-4.3 ppm) of compound **23** in $CDCl_3$

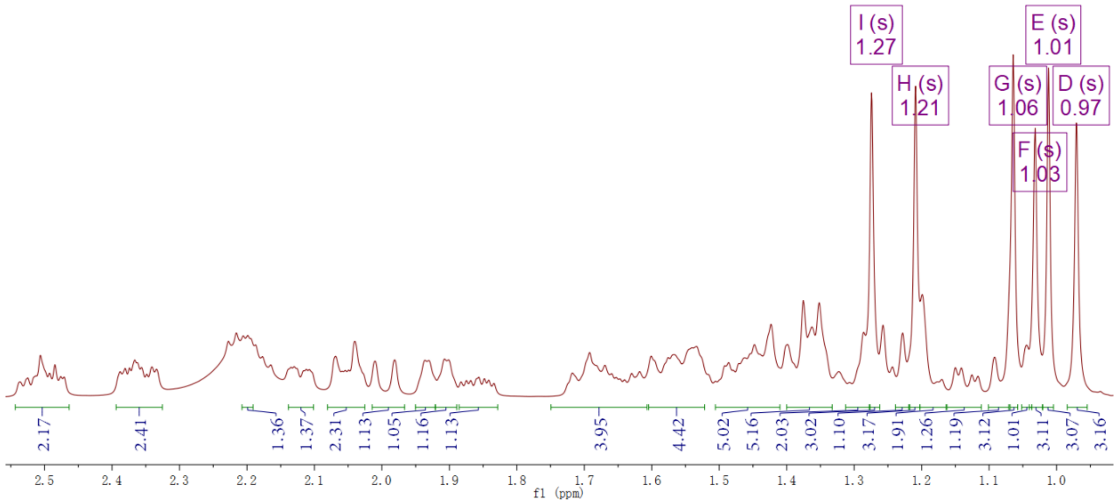


**Figure S135c.** Partial $^{1}H$ NMR spectrum ($\delta$ 0.9-2.6 ppm) of compound **23** in $CDCl_3$

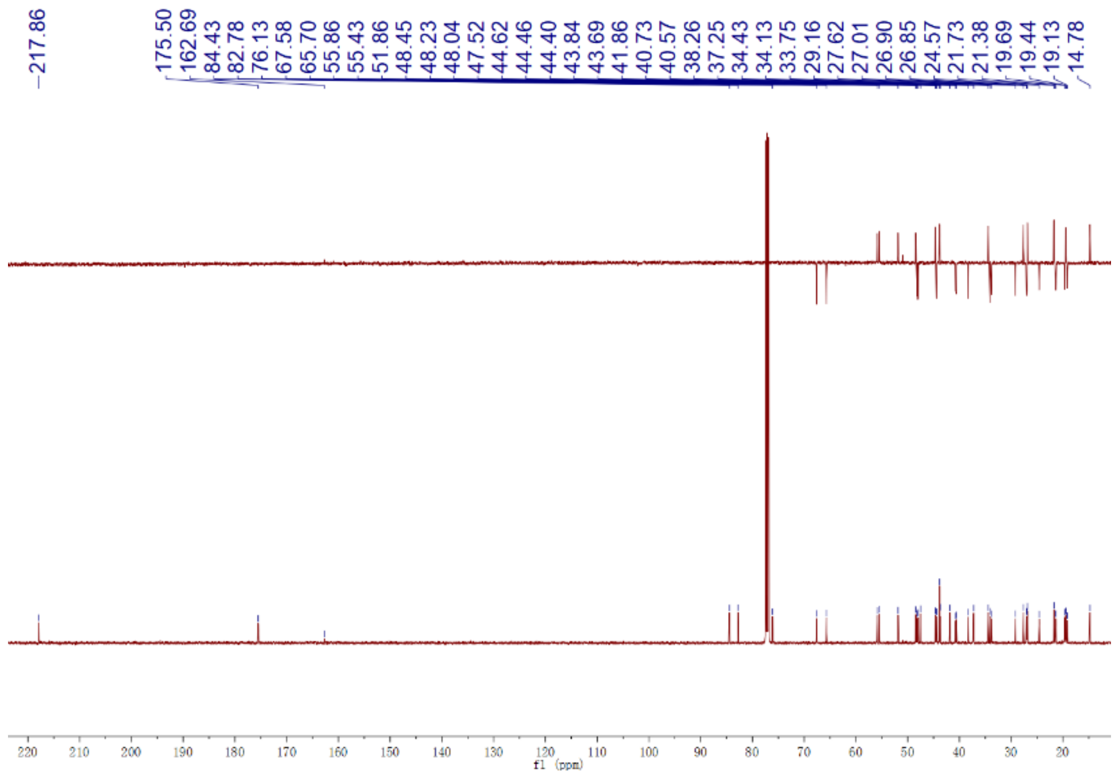


**Figure S136.** $^{13}C$ NMR and DEPT 135 spectrum of compound **23** in $CDCl_3$

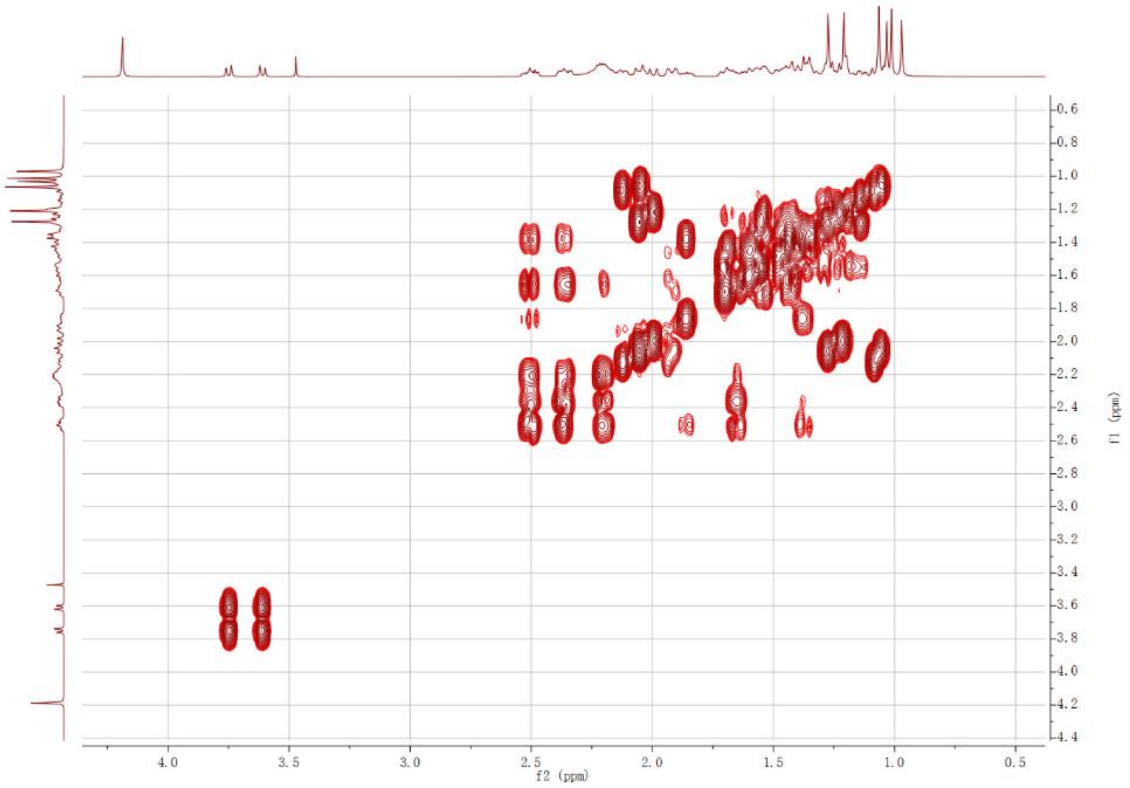


**Figure S137.** $^1$H-$^1$H COSY spectrum of compound **23** in $CDCl_3$

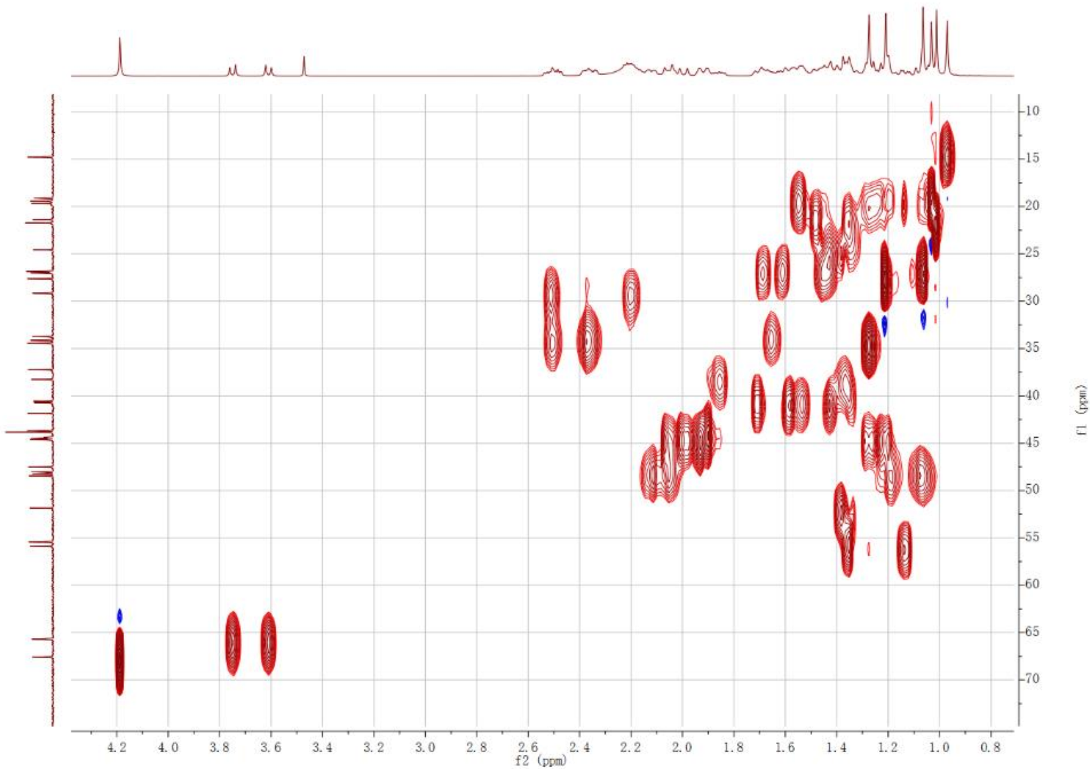


**Figure S138.** HSQC spectrum of compound **23** in $CDCl_3$

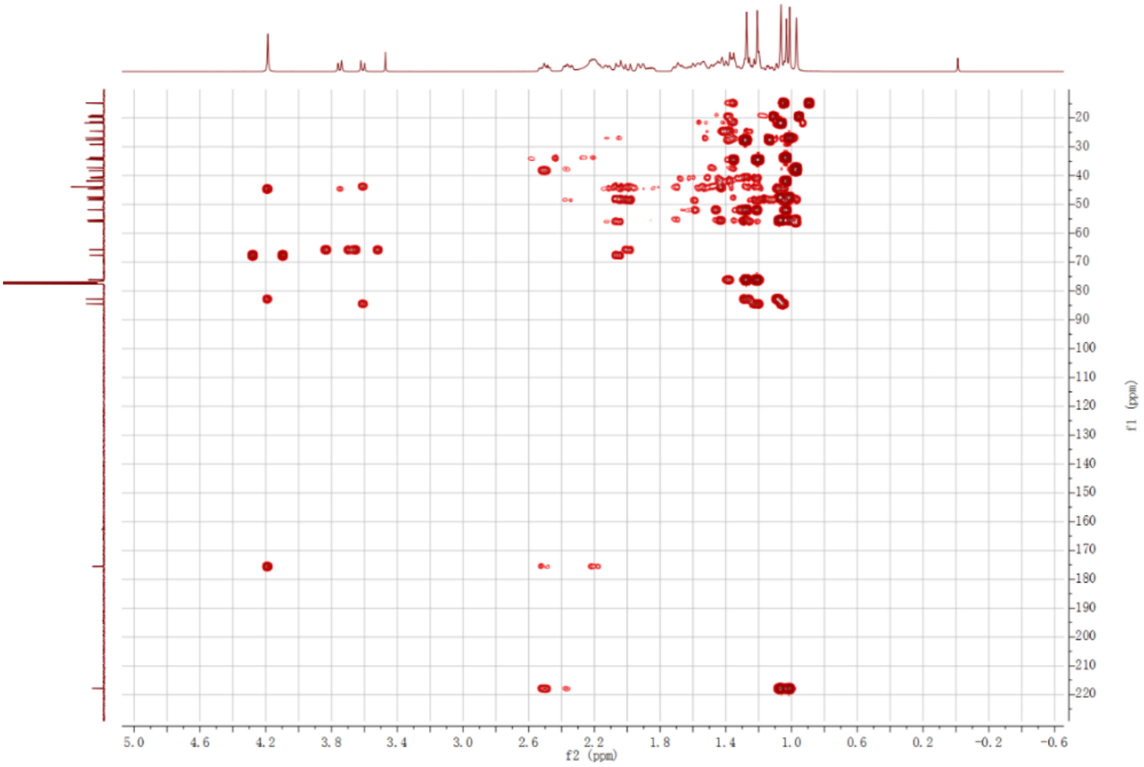


**Figure S139.** HMBC spectrum of compound **23** in $CDCl_3$

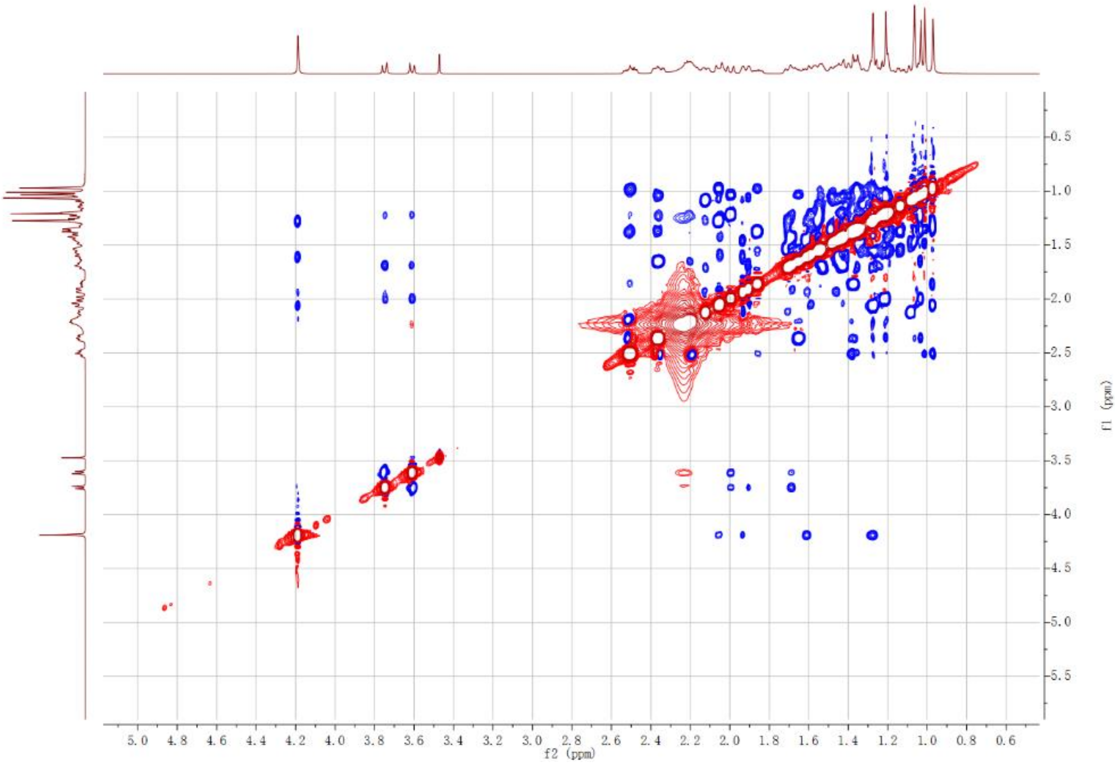


**Figure S140.** NOESY spectrum of compound **23** in $CDCl_3$

Tolerance = 5.0 PPM / DBE: min = -1.5, max = 50.0
Element prediction: Off
Number of isotope peaks used for i-FIT = 3

Monoisotopic Mass, Even Electron Ions
300 formula(e) evaluated with 3 results within limits (up to 50 closest results for each mass)
Elements Used:
C: 0-100 H: 0-200 O: 0-20 Na: 0-1
LX
20240422-LX-S019 1003 (7.324)

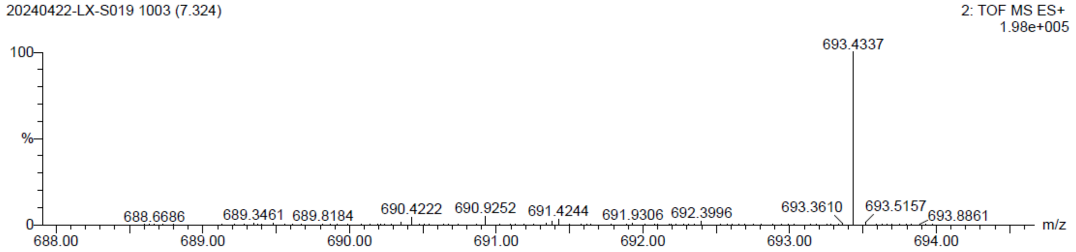


| | | | | | | | | | |
|---|---|---|---|---|---|---|---|---|---|
| Minimum: | 80.00 | | | | -1.5 | | | | |
| Maximum: | 100.00 | | 5.0 | 5.0 | 50.0 | | | | |
| Mass | RA | Calc. Mass | mDa | PPM | DBE | i-FIT | Norm | Conf(%) | Formula |
| 693.4337 | 100.00 | 693.4342 | -0.5 | -0.7 | 9.5 | 169.0 | 0.366 | 69.33 | C40 H62 O8 Na |
| | | 693.4308 | 2.9 | 4.2 | 21.5 | 170.6 | 1.921 | 14.65 | C49 H57 O3 |
| | | 693.4366 | -2.9 | -4.2 | 12.5 | 170.5 | 1.831 | 16.02 | C42 H61 O8 |

**Figure S141.** HRESIMS spectrum of compound **24**

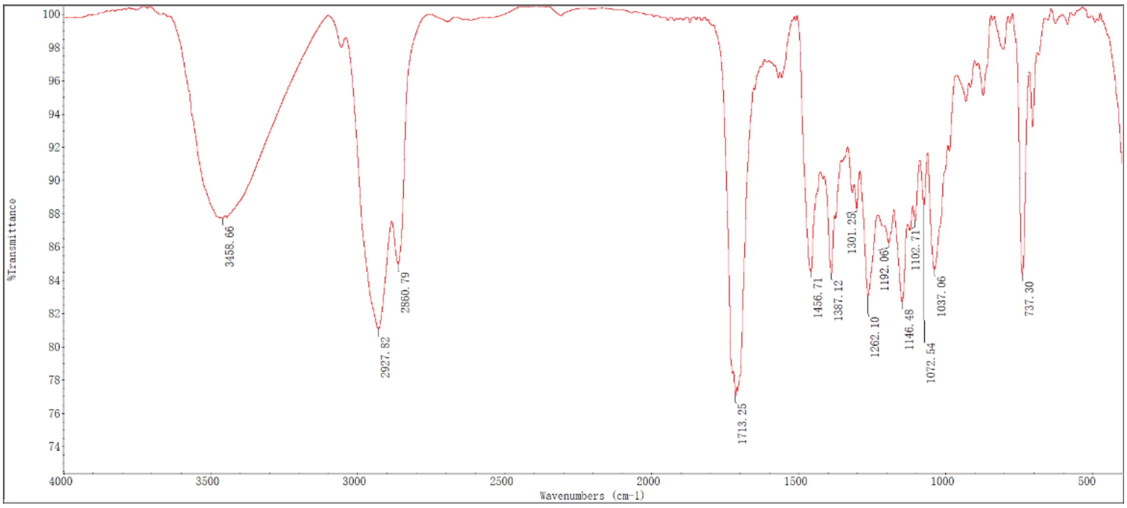


**Figure S142.** IR spectrum of compound **24**

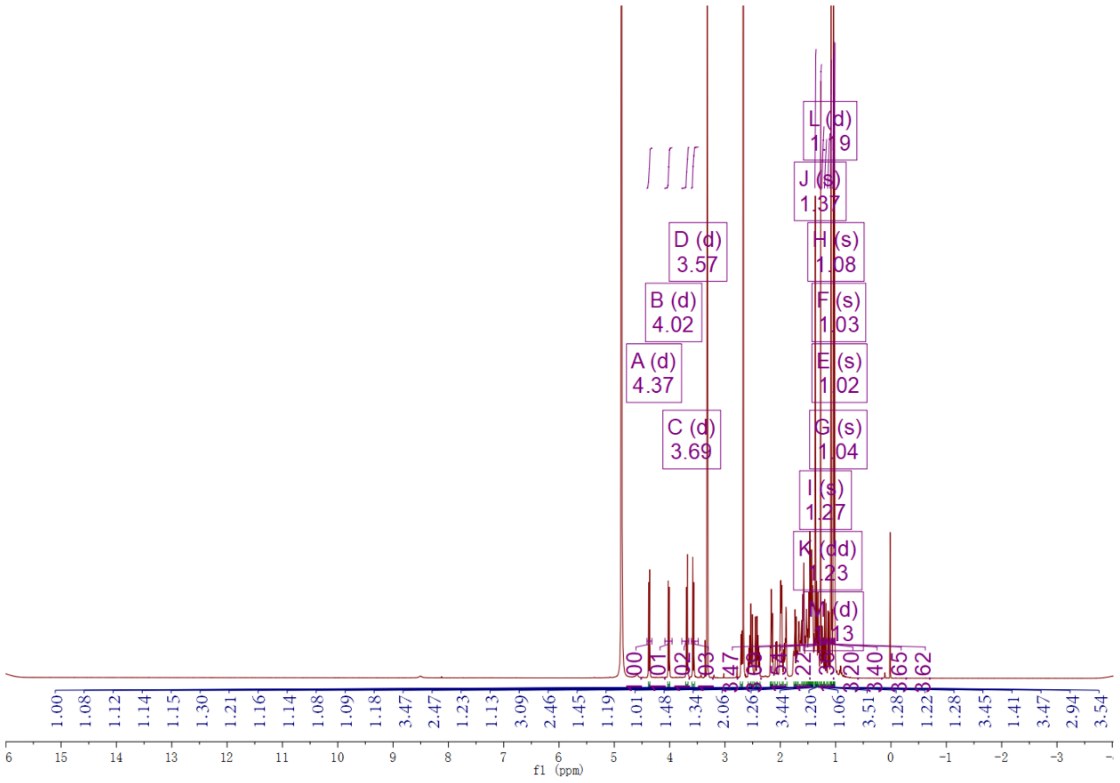


**Figure S143a.** $^{1}$H NMR spectrum of compound **24** in $CD_3OD$

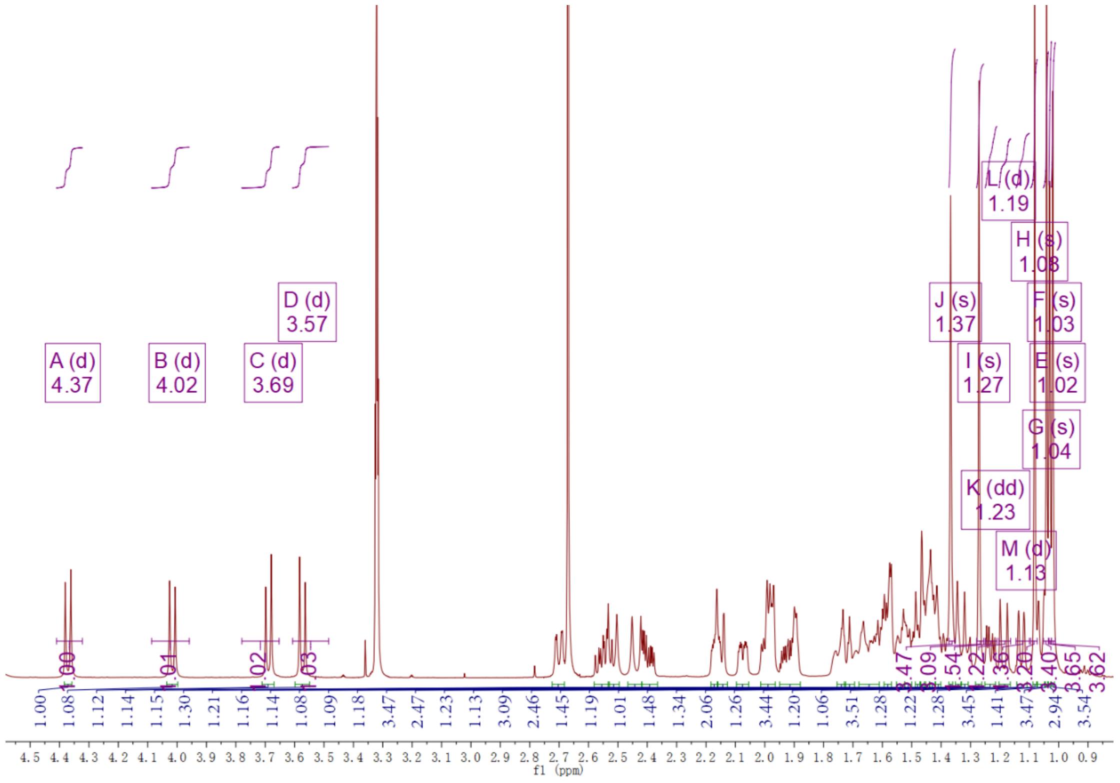


**Figure S143b.** Partial $^{1}$H NMR spectrum ($\delta$ 0.8-4.5 ppm) of compound **24** in $CD_3OD$

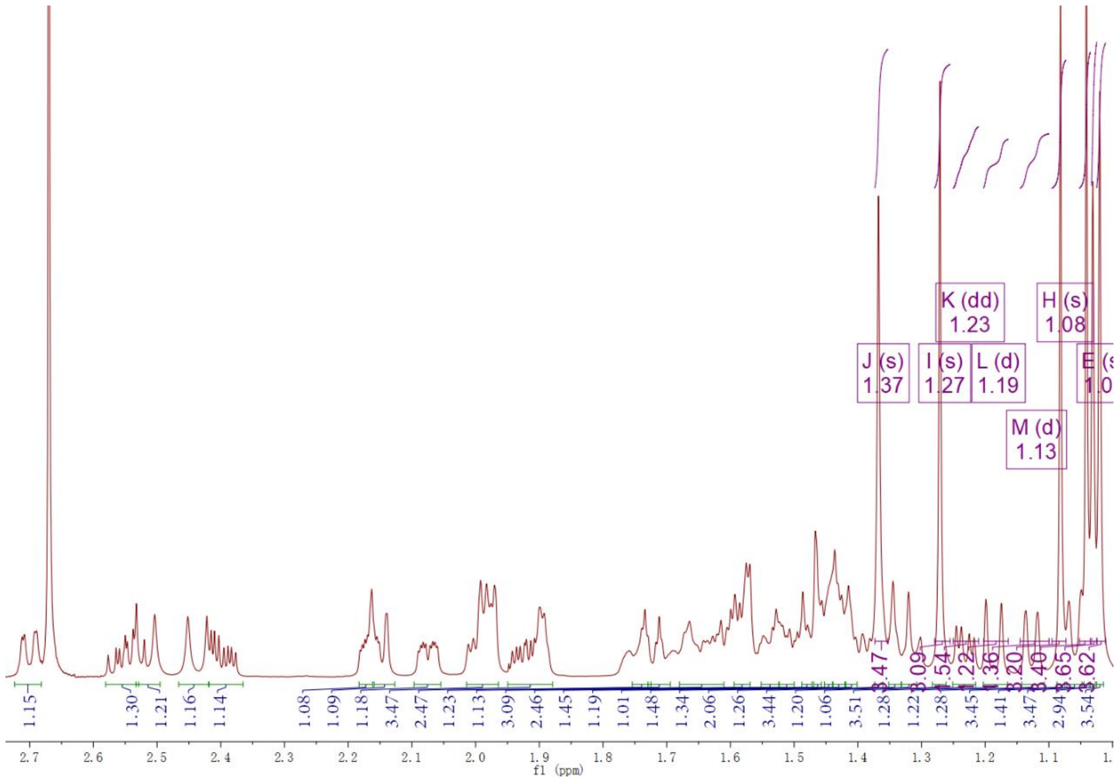

**Figure S143c.** Partial $^{1}$H NMR spectrum ($\delta$ 1.0-2.8 ppm) of compound **24** in $CD_3OD$

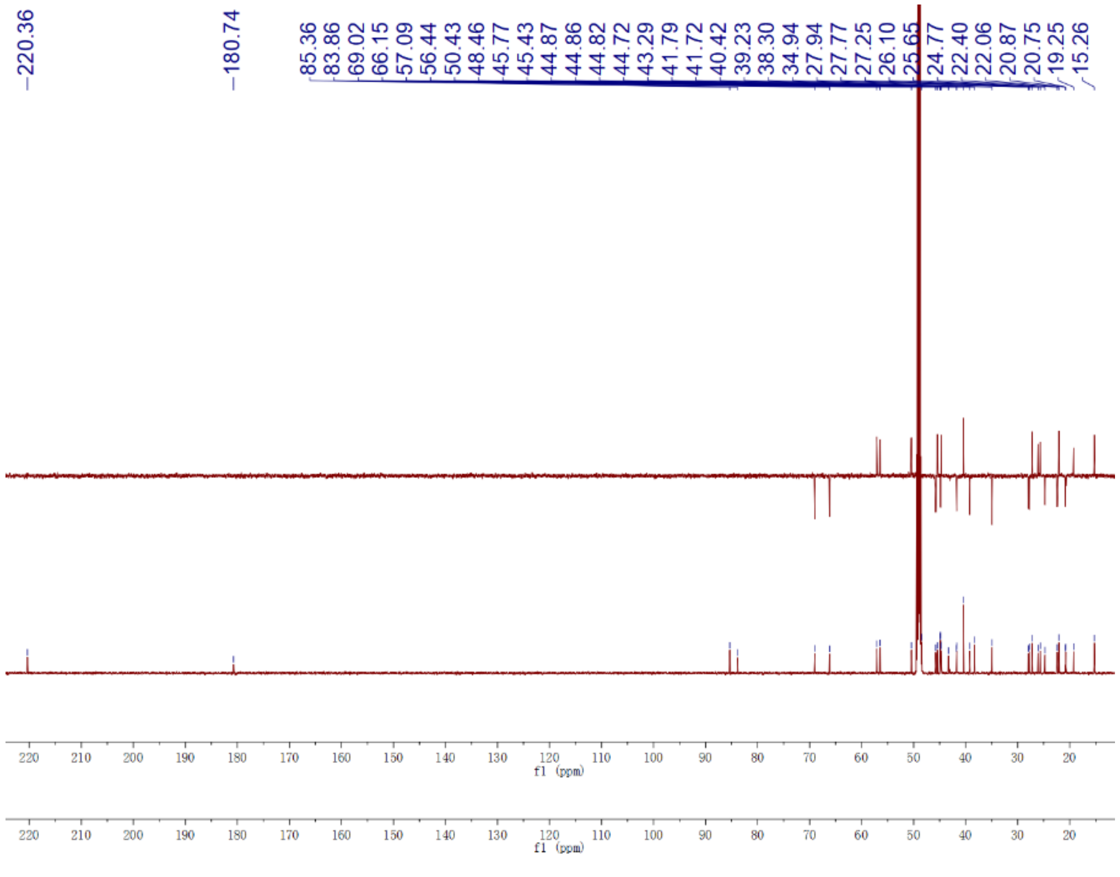

**Figure S144.** $^{13}$C NMR and DEPT 135 spectrum of compound **24** in $CD_3OD$

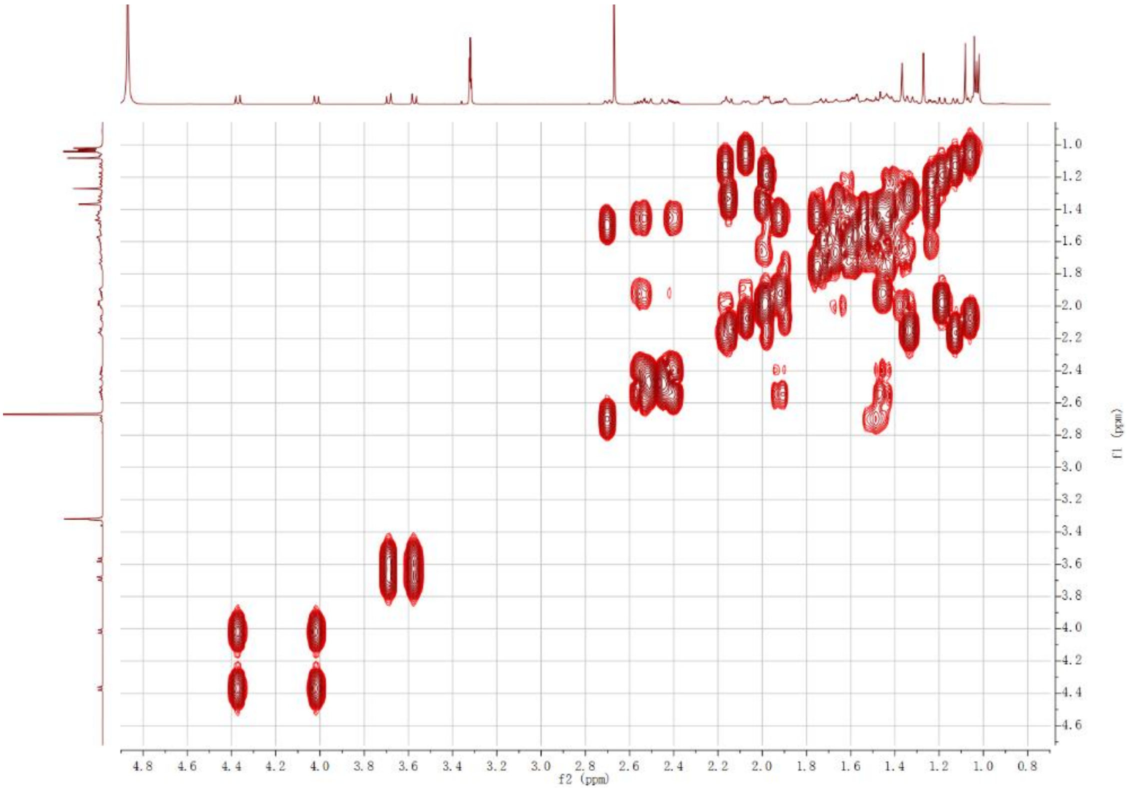


**Figure S145.** $^{1}H$-$^{1}H$ COSY spectrum of compound **24** in $CD_3OD$

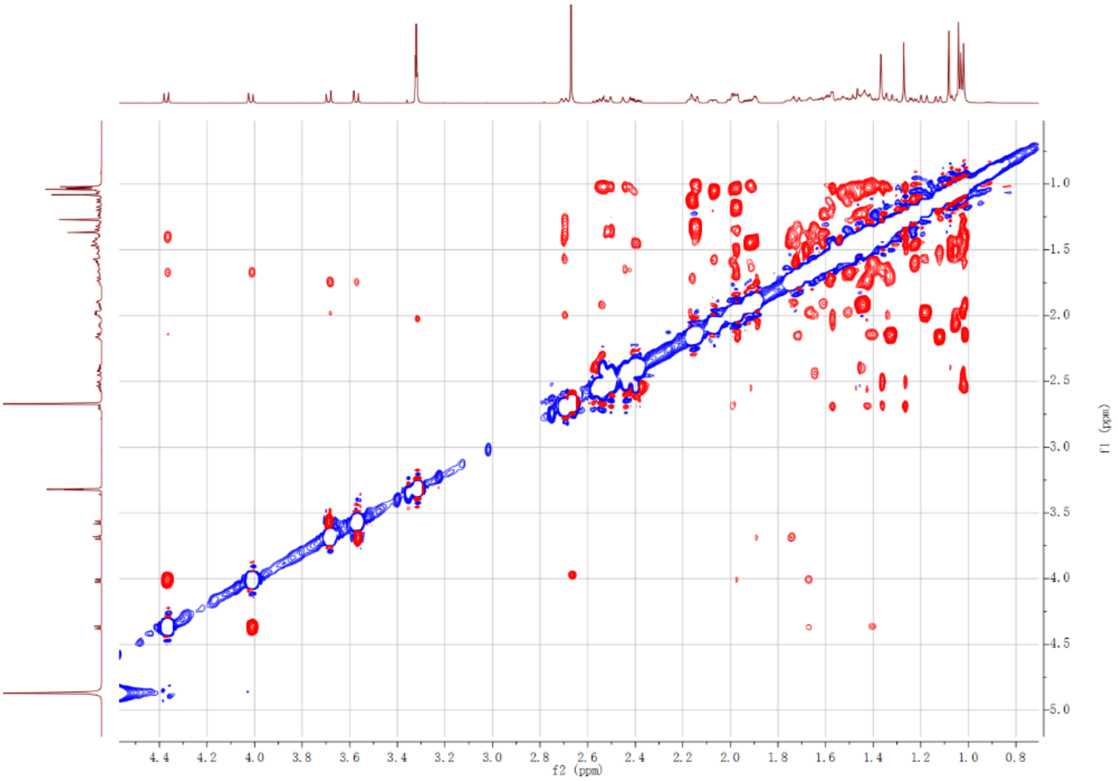


**Figure S146.** HSQC spectrum of compound **24** in $CD_3OD$

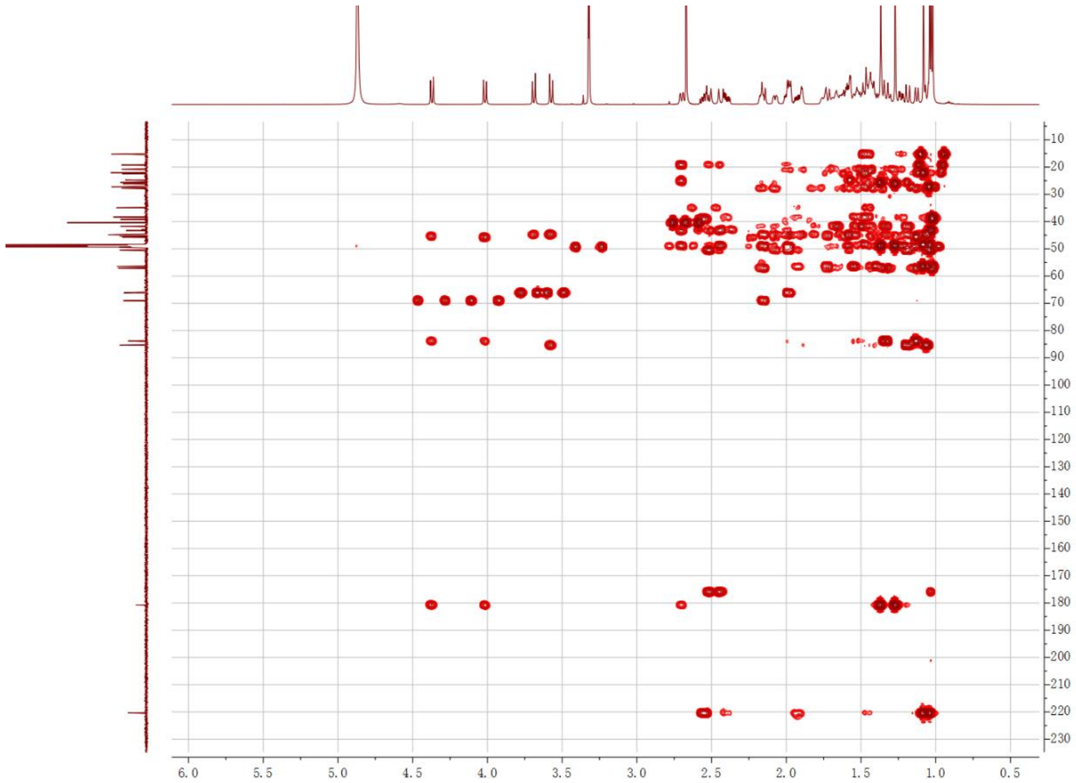


**Figure S147.** HMBC spectrum of compound **24** in $CD_3OD$

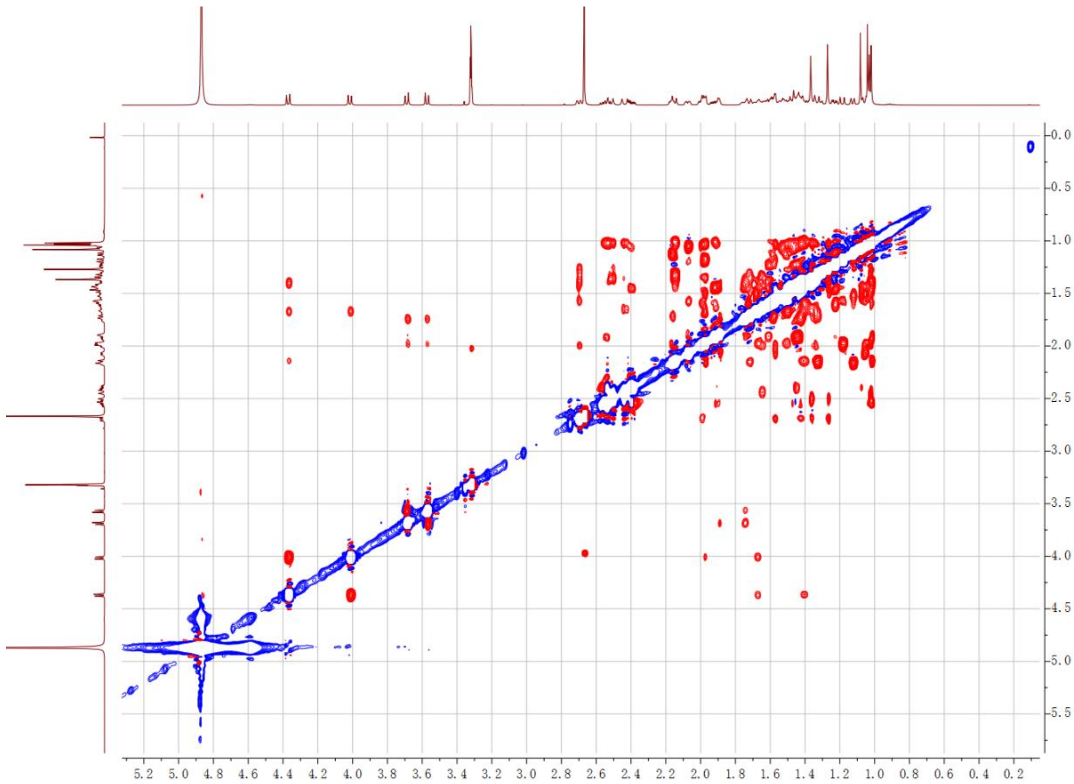


**Figure S148.** NOESY spectrum of compound **24** in $CD_3OD$
